# Fatigue, interoplastic and nociplastic distress in myalgic encephalomyelitis/chronic fatigue syndrome, Gulf War Illness, and chronic idiopathic fatigue

**DOI:** 10.3389/fnins.2025.1530652

**Published:** 2025-08-25

**Authors:** Emily Chen, Tamera Rudder, Charles Nwankwere, James N. Baraniuk

**Affiliations:** Department of Medicine, Georgetown University Medical Center, Washington, DC, United States

**Keywords:** fatigue, postexertional malaise, interoception, tenderness, dolorimetry, pain, nociception, disability

## Abstract

**Introduction:**

Myalgic encephalomyelitis/chronic fatigue syndrome (ME/CFS) and Gulf War Illness (GWI) have similar profiles of pain (nociception), visceral interoception, and tenderness (central sensitization) that may be due to dysfunction of midbrain and medulla descending antinociceptive and antiinteroceptive mechanisms. If so, then dolorimetry, a proxy for tenderness, may be correlated with subjective symptoms. The relationship with fatigue was assessed in Chronic Idiopathic Fatigue (CIF).

**Methods:**

Cohorts of ME/CFS, GWI, and sedentary control subjects completed questionnaires and had dolorimetry. Spearman correlations were calculated between central sensitization (dolorimetry), fatigue (Chalder Fatigue), pain (McGill Pain), interoception (Chronic Multisymptom Inventory), disability (SF36), psychological constructs, and other symptoms. Females were more tender than males and were thus analyzed separately.

**Results:**

GWI and ME/CFS groups were more tender than controls for females (*p* < 0.0045) and males (*p* < 10^−6^). Receiver operating characteristics area under the curve for female ME/CFS (0.730) and GWI (0.792) and male ME/CFS (0.816) and GWI (0.831) were not optimal for diagnostic purposes. Pain and interoception were highly correlated. Dolorimetry correlated better with pain (Spearman *R* = −0.574 to −0.629) than interoception (*R* = −0.417 to −0.545) questionnaires. Dolorimetry correlated weakly with fatigue and disability (|R| < 0.42). CIF was defined by receiver operating characteristics with elevated fatigue, postexertional malaise, and reduced vitality. CIF had intermediate tenderness.

**Discussion:**

The outcomes generate several hypotheses about ME/CFS and GWI pathophysiology. Disease pathologies may involve injury to midbrain and medulla regulatory pathways causing central sensitization with the loss of descending antiinteroceptive and antinociceptive inhibitory mechanisms and increased perceptions of widespread visceral complaints and pain. The diseases can be re-conceptualized as chronic disabling fatigue with heightened interoceptive and nociceptive symptoms. Variations in antiinteroceptive control may provoke unpredictable shifts in symptom spectrum and severity that contribute to exertional exhaustion and symptom exacerbation. Subjective criteria were found to define CIF prospectively.

## Introduction

Myalgic encephalomyelitis/chronic fatigue syndrome (ME/CFS; Naranch et al., 2002) and Gulf War Illness (GWI; Surian and Baraniuk, 2020) are characterized by fatigue, postexertional malaise (PEM), sleep disruption, neurocognitive disruption, widespread pain, orthostatic intolerance, and a wide range of interoceptive or “inner body” complaints, such as sensitivity to irritants, dyspnea, and gastrointestinal distress. We propose that heightened awareness of pain and interoceptive sensations is due to lapses in midbrain and brainstem antinociceptive and antiinteroceptive systems that ordinarily filter and suppress the transmission of these sensations to centers of conscious perception in the cerebrum. Dysfunction of midbrain, brainstem, and descending antinociceptive pathways is termed central sensitization and leads to central or neuroplastic pain.

We propose that dysfunction of midbrain and brainstem mechanisms and descending antiinteroceptive and antinociceptive pathways contribute to the pathologies of ME/CFS and GWI (Baraniuk, 2022). Dysfunctional descending inhibitory processes lead to tenderness that can be quantified by pressure-induced pain thresholds (kg) measured by dolorimetry (algometry) and tender point counts. These measures are predicted to correlate with generalized pain sensations. With regard to interoceptive discomfort, we predict that identical or separate analogous interoplastic mechanisms will contribute to interoceptive discomfort. If nociplastic and interoplastic outcomes have shared mechanisms, then pain and pressure thresholds should correlate with interoceptive complaints. ME/CFS and GWI are disabling conditions, and so measures of quality of life are anticipated to correlate with pain and tenderness. Fatigue is a difficult sensation or emotion to define, but insights may be gained by contrasting its severity with tenderness, interoception, nociception, and disability. Fatigue without other symptoms was examined by studying chronic idiopathic fatigue (CIF) subjects.

Tenderness is an objective measure of nociception that is demonstrated by applying a stimulus such as pressure to the skin in order to elicit a painful response. Tenderness has been demonstrated in ME/CFS (Naranch et al., 2002) and GWI (Surian and Baraniuk, 2020) and is a pillar of the concept of fibrositis (Bennett, 1981), central sensitization syndrome (Cuesta-Vargas et al., 2018), and the diagnosis of fibromyalgia (FM) using the 1990 criteria (Wolfe et al., 2010; Clauw, 2024). Tenderness is proposed to be the consequence of disrupted central “nociplastic” regulation of pain signaling (Nijs et al., 2021). Unmyelinated nociceptive and multimodal afferent nerves synapse with secondary interneurons in the spinal dorsal horn that ascend in the spinal cord to the brain. Pain transmission is regulated by descending antinociceptive pathways that originate in the periaqueductal gray matter and medulla and project to the dorsal horn. Interruption of the pathway(s) leads to increased perception of pain and mechanically induced tenderness. The central mechanism has been classified as nociplastic to distinguish it from nociceptive pain caused by peripheral inflammation with excessive stimulation of nerve endings and nocipathic pain resulting from injury to conducting nerves or ganglia. The absence of the descending inhibitory tone permits increased conduction of painful sensations so that a low level of peripheral stimulation that would ordinarily be insufficient to trigger signal transmission can now convey ascending perceptions of pain via spinothalamic routes. This is termed systemic hyperalgesia. In addition, dysregulation in the dorsal horn allows innocuous peripheral stimuli such as light touch, proprioception, or vibration to incorrectly generate nociceptive signals (allodynia; Jensen and Finnerup, 2014). Collectively, these processes are called central sensitization. The peripheral triggering of pain sensations, i.e., tenderness, can be measured by dolorimetry (algometry), where a pressure gauge is pressed against the skin and the pressure causing pain is recorded (kg). The nociceptive and interoceptive pathways are distinct from the highly myelinated peripheral somatosensory, proprioceptive, dermatomal, muscular, and exertioceptive nerves that synapse in the dorsal horn and ascend to the thalamus and thence through the internal capsule to the primary somatosensory cortex.

The relevance of nociplastic and presumed interoplastic mechanisms can be appreciated by examining the many symptoms that are attributed to internal organs and mucosal surfaces in ME/CFS, GWI, and FM. The evolution of diagnostic criteria for ME/CFS, GWI, FM, and CIF provides the context for quantifying the relationships between these core dimensions of disease severity and mechanisms of disease.

ME/CFS is defined by prolonged debilitating fatigue, postexertional malaise (PEM), cognitive and other physical symptoms, plus the exclusion of known chronic medical and psychiatric diseases (Fukuda et al., 1994; Carruthers et al., 2003). Prevalence is 0.1–2%, with a wide range in part because of differences in diagnostic criteria in different studies (Lim et al., 2020). The etiology has been presumed to be a post-infectious disorder (Holmes, 1988), as inferred from polio, enterovirus (Dowsett et al., 1990), Epstein–Barr (Pedersen et al., 2019), and other infectious diseases (Bannister, 1988) that may occur in sporadic or epidemic fashion, and as suggested for post-COVID-19 (coronavirus disease 2019) fatigue (Carfì et al., 2020; Thaweethai et al., 2023; Fineberg et al., 2024). Evidence of autoimmunity (Sotzny et al., 2018), as well as immune, metabolome (Tomas and Newton, 2018), and autonomic (Nelson et al., 2019) dysfunction, makes the spectrum of molecular mechanism(s) more complex. Because no biomarkers have been validated, the diagnosis relies on patient history, physical examination, and the exclusion of chronic medical and psychiatric conditions based on clinical practice and routine blood work.

**Figure 1:**
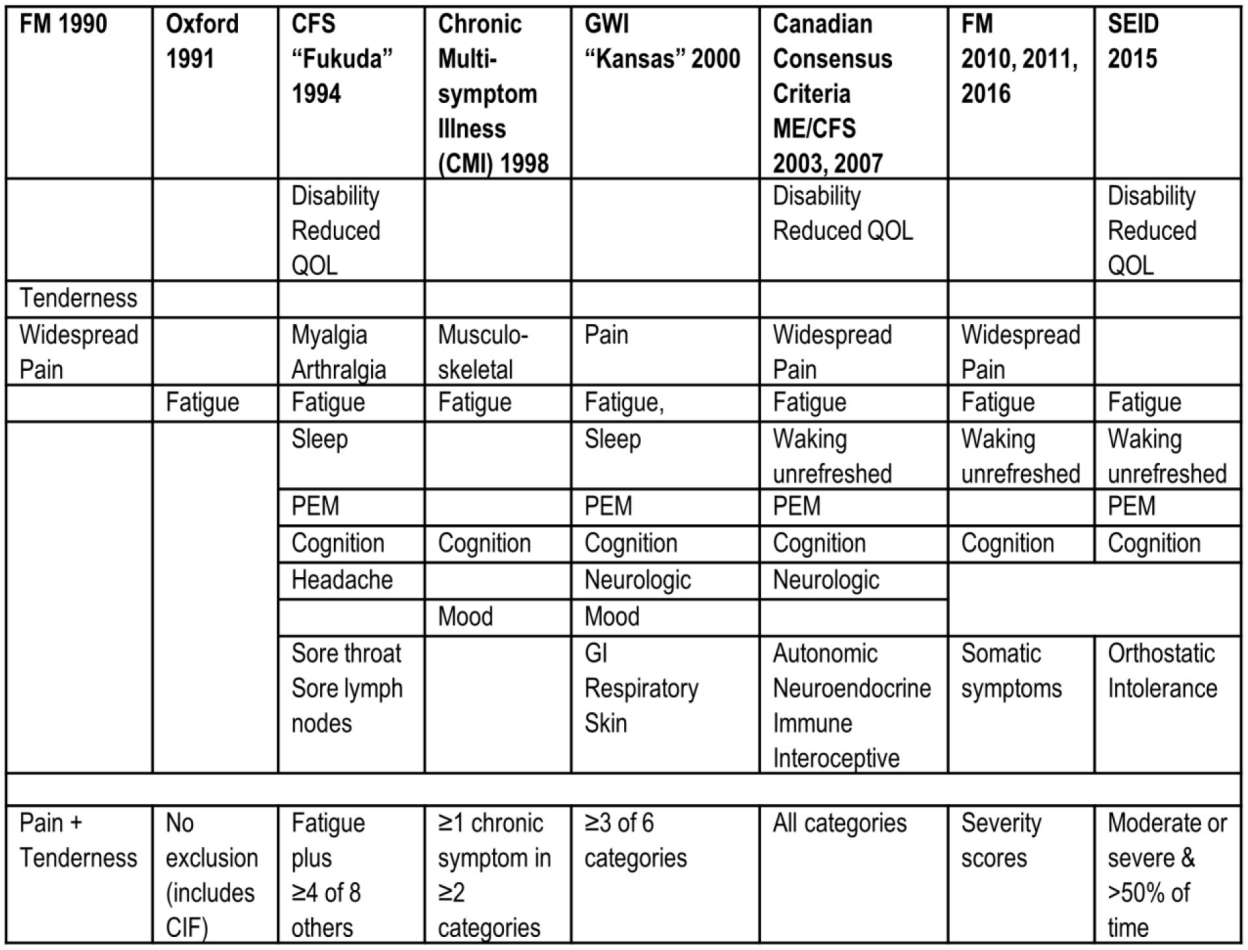
Diagnostic criteria. Changes in criteria for ME/CFS, GWI, and fibromyalgia (FM) over time are indicated from left to right. The 1990 FM criteria were the only ones to require an objective finding (tenderness to pressure or systemic hyperalgesia). Postexertional malaise (PEM) is proposed to be the most discriminating symptom in ME/CFS and SEID criteria. Disability and reduced quality of life (QOL) are often not quantified in research manuscripts.

The diagnostic criteria for ME/CFS have evolved from lists of symptoms experienced in patient cohorts to more focused consensus approaches (Figure 1; Jason et al., 2016, 2015, 2012). Benign myalgic encephalomyelitis was introduced by Ramsay in 1973 (Ramsay, 1973) and post-infectious chronic fatigue by Holmes in 1988 (Holmes, 1988). The 1994 Centers for Disease Control and Prevention (CDC) criteria (“Fukuda”; Fukuda et al., 1994) expanded criteria by requiring more than 6 months of disabling, prolonged, or relapsing fatigue with at least four of eight ancillary symptoms: post-exertional malaise, cognitive problems with memory or concentration, unrefreshing sleep, muscle pain, joint pain, headaches, sore throat, and tender lymph nodes. Specificity is improved by requiring moderate or severe symptoms for case designation, as in the ME/CFS severity questionnaire (CFSQ) used here (Baraniuk et al., 2013a). More recent criteria have increased the emphasis on postexertional symptom exacerbations or postexertional malaise (PEM) in the 2003 Carruthers Canadian Consensus Criteria for Myalgic Encephalomyelitis/Chronic Fatigue Syndrome (ME/CFS; CCC; Carruthers et al., 2003; Carruthers, 2007), the 2011 Myalgic Encephalomyelitis International Consensus Criteria (International; Carruthers et al., 2011) and Systemic Exertion Intolerance Disease (SEID) defined by the Institute of Medicine in 2015 (Committee on the Diagnostic Criteria for Myalgic Encephalomyelitis/Chronic Fatigue Syndrome, Board on the Health of Select Populations, and Institute of Medicine, 2015). Symptom severity should be moderate or severe and present at least half of the time (Jason et al., 2013; Sunnquist et al., 2019; Bedree et al., 2019). In 2024, the National Academy of Sciences, Engineering, and Medicine recommended considering ME/CFS and Long COVID as post-infectious diseases with potentially shared pathophysiologies (Fineberg et al., 2024). PEM refers to debilitating symptom exacerbation that impairs activities of daily living following mild physical, cognitive, or emotional stressors. Onset is often delayed 24 h, and dysfunction may last for 72 h or more (Chu et al., 2018; Stussman et al., 2020). Sleep is not refreshing, which distinguishes PEM from the temporary exertional tiredness experienced by healthy people and those with chronic illnesses who return to normal after rest and refreshing sleep. Note that postexertional malaise was not required for the 1994 Fukuda criteria (Fukuda et al., 1994), but both postexertional malaise and fatigue are required by the CCC (Carruthers et al., 2003; Carruthers, 2007), International (Carruthers et al., 2011), and SEID (Committee on the Diagnostic Criteria for Myalgic Encephalomyelitis/Chronic Fatigue Syndrome, Board on the Health of Select Populations, and Institute of Medicine, 2015) criteria. Also, the SEID criteria removed pain as a requirement in order to avoid overlap with chronic nociceptive illnesses such as fibromyalgia. The shifting criteria emphasize the need to reframe the role of pain in ME/CFS and to understand patients who have chronic fatigue, pain, and interoceptive discomfort.

Fibromyalgia (FM) is the prototypical nociplastic disease with pain and tenderness due to central sensitization and systemic hyperalgesia (Nijs et al., 2021; Fitzcharles et al., 2021; Kaplan et al., 2024). Clinical criteria have evolved over the years (Clauw, 2024). The 1990 American College of Rheumatology criteria for FM required widespread pain plus tenderness to thumb pressure at ≥11 of 18 traditional tender points (Wolfe et al., 2010; Figure 1). Pressure should be sufficient to blanch the thumbnail bed or ~4 kg (Bennett, 1981). However, tender point counts correlate with catastrophizing, general distress, fatigue, depression, sleep, and orthostasis, and may be independent of pain (Giesecke et al., 2003; Petzke et al., 2003; Geisser et al., 2007; Croft et al., 1994; Geisser et al., 2003; Davydov et al., 2024). The technique is challenging to calibrate and standardize between investigators. Tenderness in FM is present diffusely throughout the body and is not localized to the 18 traditionally specified sites (Petzke et al., 2001). Therefore, tender point counts were removed from the 2010 revision of the FM criteria (Wolfe et al., 2011), even though the concept of tenderness is still considered important for FM diagnosis in clinical practice (Bettini and Moore, 2016). The 2010 revision tabulates painful body regions (Pain and Tenderness in the Past Week; Wolfe et al., 2011) and was expanded to include graded assessments of the severity of fatigue, cognitive difficulties, problems upon waking up, and somatic complaints (Figure 1; Gracely and Schweinhardt, 2015; Yunus, 2008). A modification in 2011 maintained widespread pain, fatigue, cognition, and sleep, but changed somatic complaints to nominal confirmation of headache, lower abdominal pain, and feeling depressed (Wolfe et al., 2011). The 2016 amendment excluded chronic regional pain syndrome (Wolfe et al., 2016). The criteria for fibromyalgia were reduced in 2019 to chronic widespread pain with either fatigue or sleep disturbances lasting more than 3 months (Arnold et al., 2019). Widespread sensory sensitivity is appreciated (Rafferty and Ward, 2024). These modifications increased the overlap between the criteria for FM, ME/CFS, and GWI, and blurred distinctions between these clinical entities (Ramírez-Morales et al., 2022).

Gulf War Illness (GWI) criteria overlap with ME/CFS (Figure 1). GWI was defined by meeting both the 1998 Centers for Disease Control Chronic Multisymptom Illness (CMI; Fukuda et al., 1998) and the 2000 Kansas (Steele, 2000) criteria. CMI requires two of three complaints: fatigue, mood and cognition, or musculoskeletal pain (Fukuda et al., 1998). The CMI criteria follow a tridimensional approach that probes tiredness as a psychosocial dimension, with restrained social activity due to fatigue or mood impairment, cognitive complaints with a decreased ability to maintain attention and memory impairment, and somatic musculoskeletal pain (Brurberg et al., 2014; Onozuka and Yen, 2008). A limitation is that the original criteria did not have to be graded for severity.

The Kansas criteria were defined from a population study of Kansas veterans that identified 28 symptoms organized into six domains, which were significantly more prevalent in deployed compared to non-deployed veterans of the 1990–1991 Persian Gulf War (Steele, 2000). GWI is diagnosed by having at least three of six domains with moderate or severe complaints. Although there is extensive overlap of ME/CFS and GWI symptoms, these are different diseases because ME/CFS has a sporadic onset and affects predominantly women, while GWI occurs in 25–32% of the cohort of predominantly young adult male military personnel deployed to the Persian Gulf theater of operations in 1990–1991 who were exposed to nerve agents (Sarin), pyridostigmine bromide pills, cholinesterase inhibitors, pesticides, oil well fire smoke, and other potentially pathological neurotoxic conditions (White et al., 2016; Maule et al., 2018). One quarter to one third of Gulf War veterans have developed chronically unremitting disease. GWI and ME/CFS differ in cognitive responses to exercise, with different patterns of change in functional connectivity involving dorsal midbrain and cortical regions during a difficult cognitive working memory task (Baraniuk et al., 2022; Provenzano et al., 2020). Note that pain, persistent fatigue, and postexertional malaise are not absolutely required for GWI diagnosis.

Chronic idiopathic fatigue (CIF) has been defined by significant unremitting fatigue lasting longer than 6 months (Vincent et al., 2012; Palacios et al., 2023). CIF and ME/CFS are different because the absence of significant ancillary nociceptive and interoceptive symptoms precludes the diagnosis of ME/CFS (Fukuda et al., 1994). Incidence and prevalence are comparable to ME/CFS (Van Amberg, 1990; Steele et al., 1998; Reeves et al., 2007). Almost half have comorbid insomnia, somatic disorders, anxiety, or depression (Vincent et al., 2012; Van Amberg, 1990; Reeves et al., 2007; Son, 2019). Subjects with CIF would meet the inappropriately lenient Oxford criteria and may have been included in the ill-conceived PACE study (Baraniuk, 2017). One hypothesis is that CIF represents an early expression of ME/CFS. However, CIF does not show cardiac or respiratory deterioration on 2-day maximal cardiopulmonary exercise testing in females (van Campen and Visser, 2021a,b) or males (van Campen and Visser, 2021a). CIF must be distinguished from frailty and fatigue that develop with advancing age (Wawrzyniak et al., 2016; Alexander et al., 2010). The CFSQ measurements and the rationale of the Fukuda criteria (Fukuda et al., 1994) were utilized to define a CIF subgroup that had significant fatigue but ≤ 3 other ancillary criteria (“quadrant analysis”; Baraniuk et al., 2013a; Baraniuk, 2017). The current data set provided insights into tenderness, fatigue, interoceptive, and affective elements of CIF.

For this study, dolorimetry pressure thresholds were compared between control, ME/CFS, and GWI groups. In secondary analysis, CIF subjects were selected from the pool and compared to the residual control, ME/CFS, and GWI groups. Pressure thresholds have a sexual dimorphism, so female and male subjects were examined separately for correlations with pain, interoception, fatigue, quality of life, and other measures. In contrast, symptoms were comparable across genders, and so the subjective correlations were recalculated for the entire cohort. Significant correlations were stratified to infer possible effects of nociplastic and interoplastic mechanisms.

## Methods and materials

A long-term prospective plan was put in place to collect standardized data to compare between disease groups. Data were collated from a series of protocols that were approved by the Georgetown University Institutional Review Board and Department of Defense Congressionally Directed Medical Research Program (CDMRP) Human Research Program Office (HRPO; A-15547 and A-18479) in accordance with the Declaration of Helsinki and listed in http://clinicaltrials.gov (NCT01291758 and NCT00810225). Subjects gave virtual informed consent to complete online questionnaires, followed by written informed consent for in-person evaluations to confirm their diagnoses.

**Figure 2:**
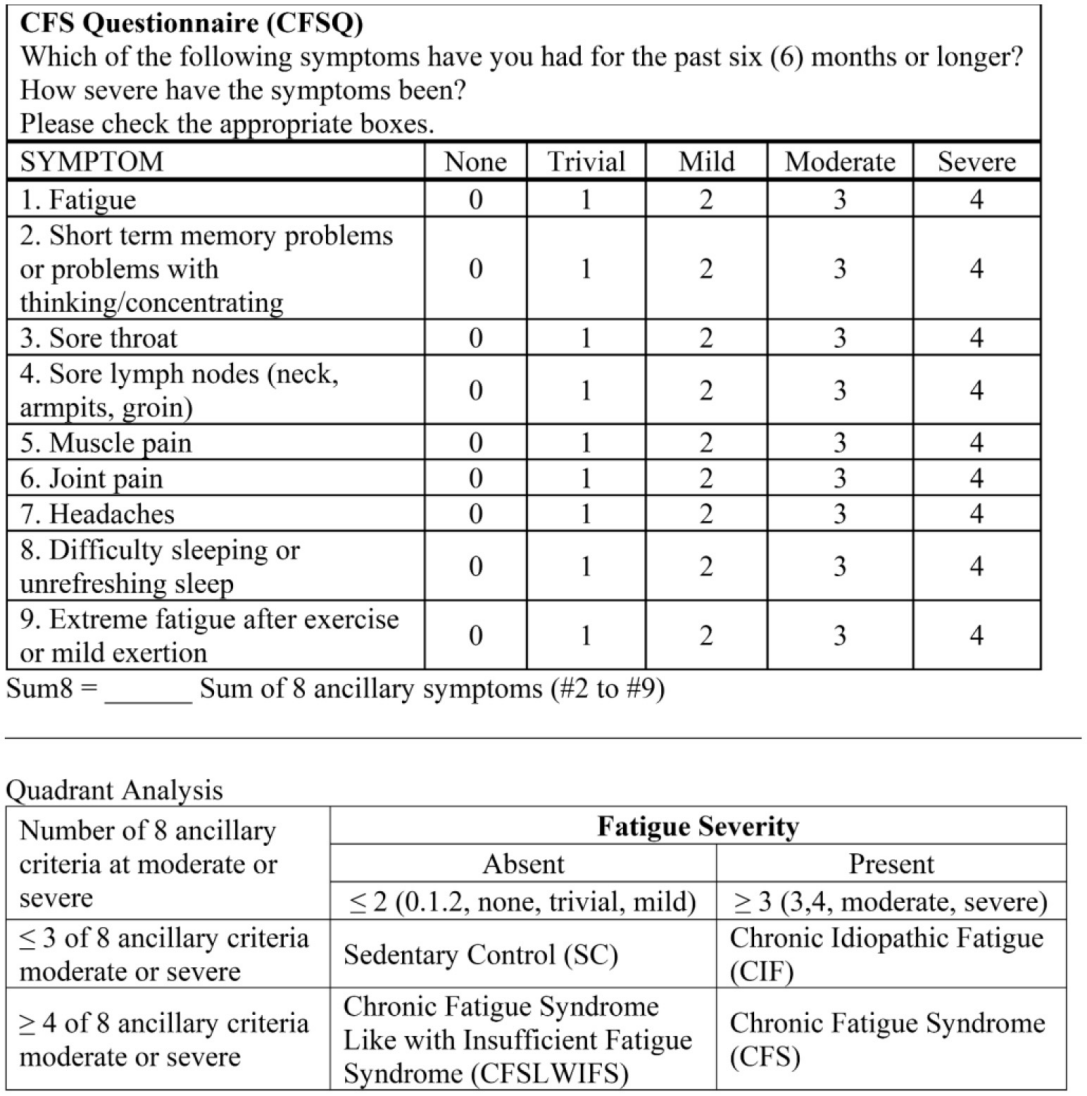
CFS questionnaire (CFSQ) and quadrant system. Severity of each fatigue and the ancillary criteria of the 1994 CDC (Fukuda) CFS definition were scored. The numbers of symptoms with moderate and severe levels were cross-referenced to identify Sedentary Controls (SC), CFS Like With Insufficient Fatigue Syndrome (CFSLWIFS), Chronic Idiopathic Fatigue (CIF), and CFS.

**Figure 3:**
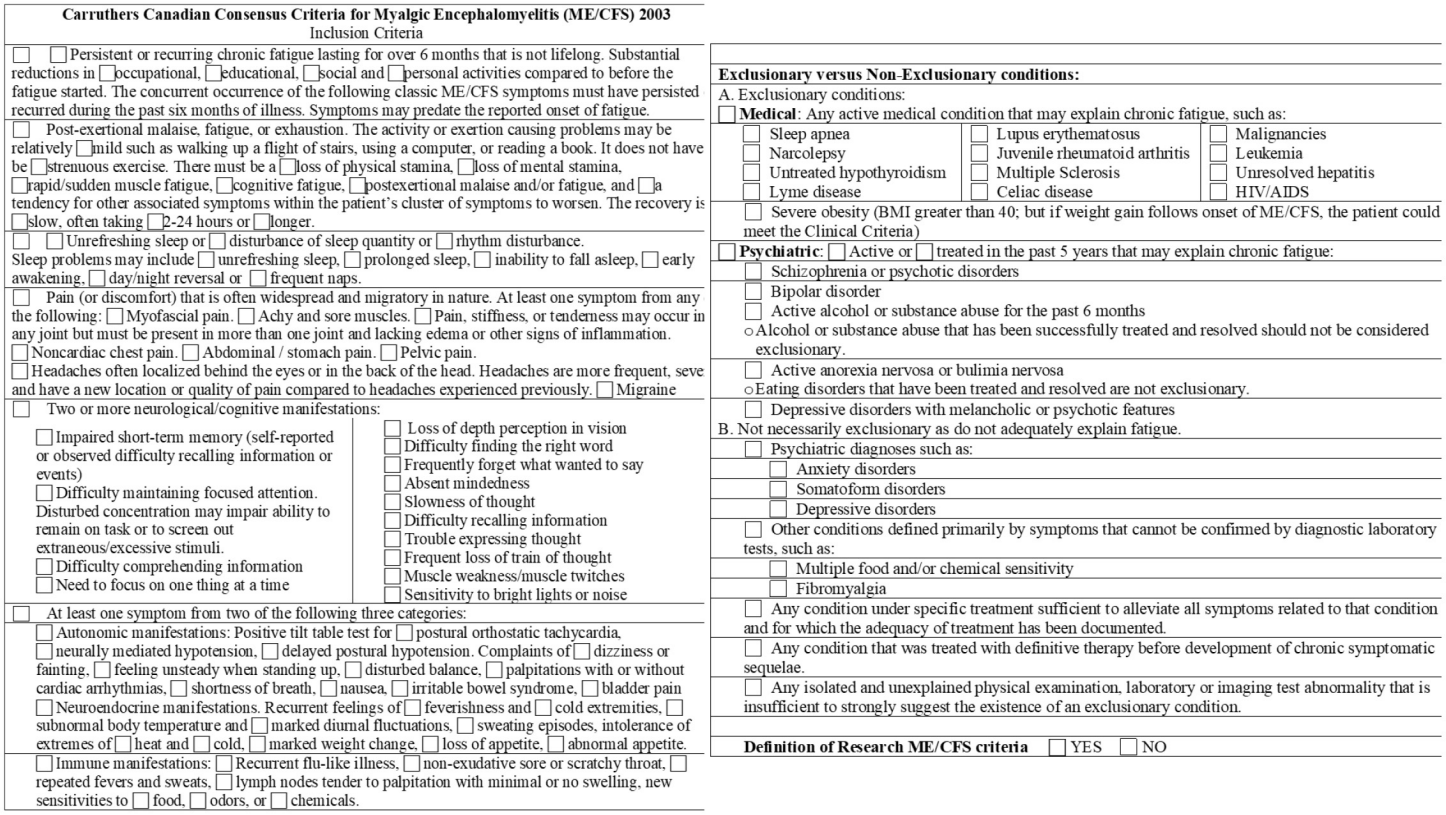
Carruthers Canadian consensus criteria for myalgic encephalomyelitis (ME/CFS) form. The checklist was taken from the original article (Carruthers et al., 2003). Symptoms were graded as present or absent in each section and were not graded for severity.

**Figure 4:**
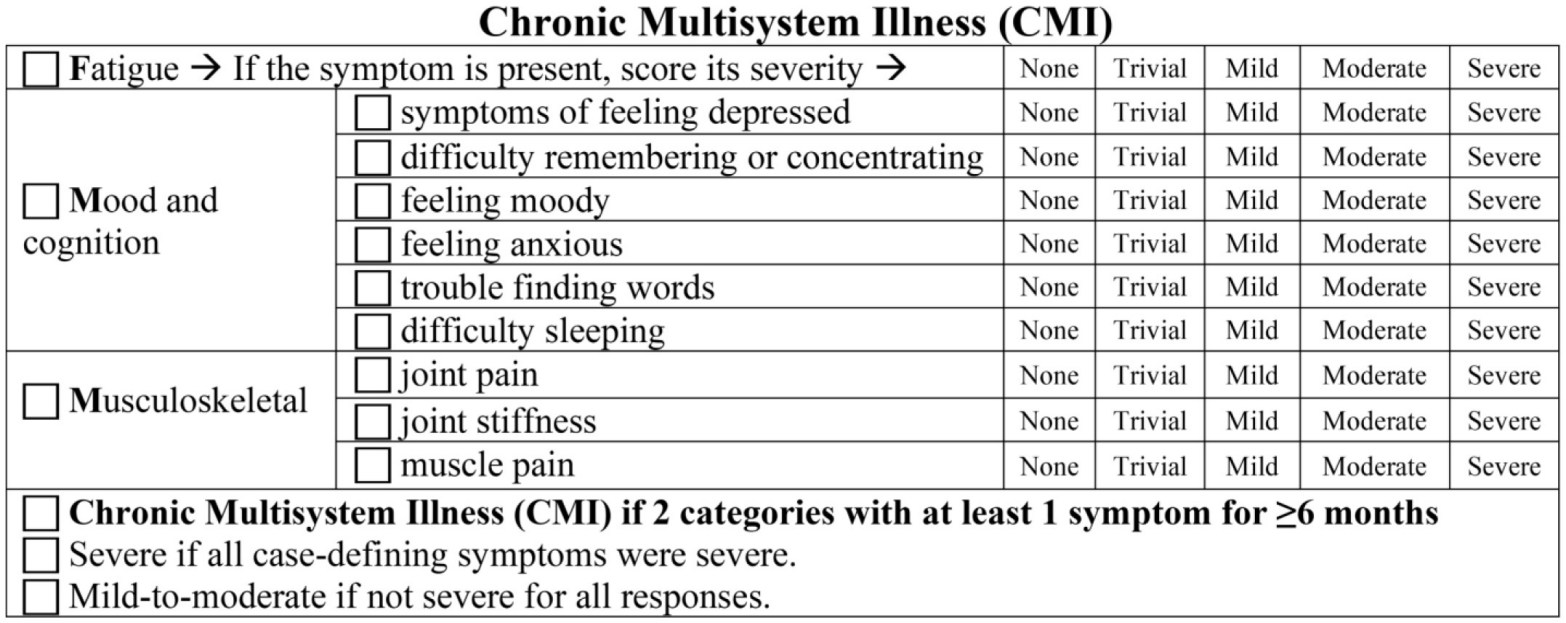
Chronic multisystem illness (CMI).

**Figure 5:**
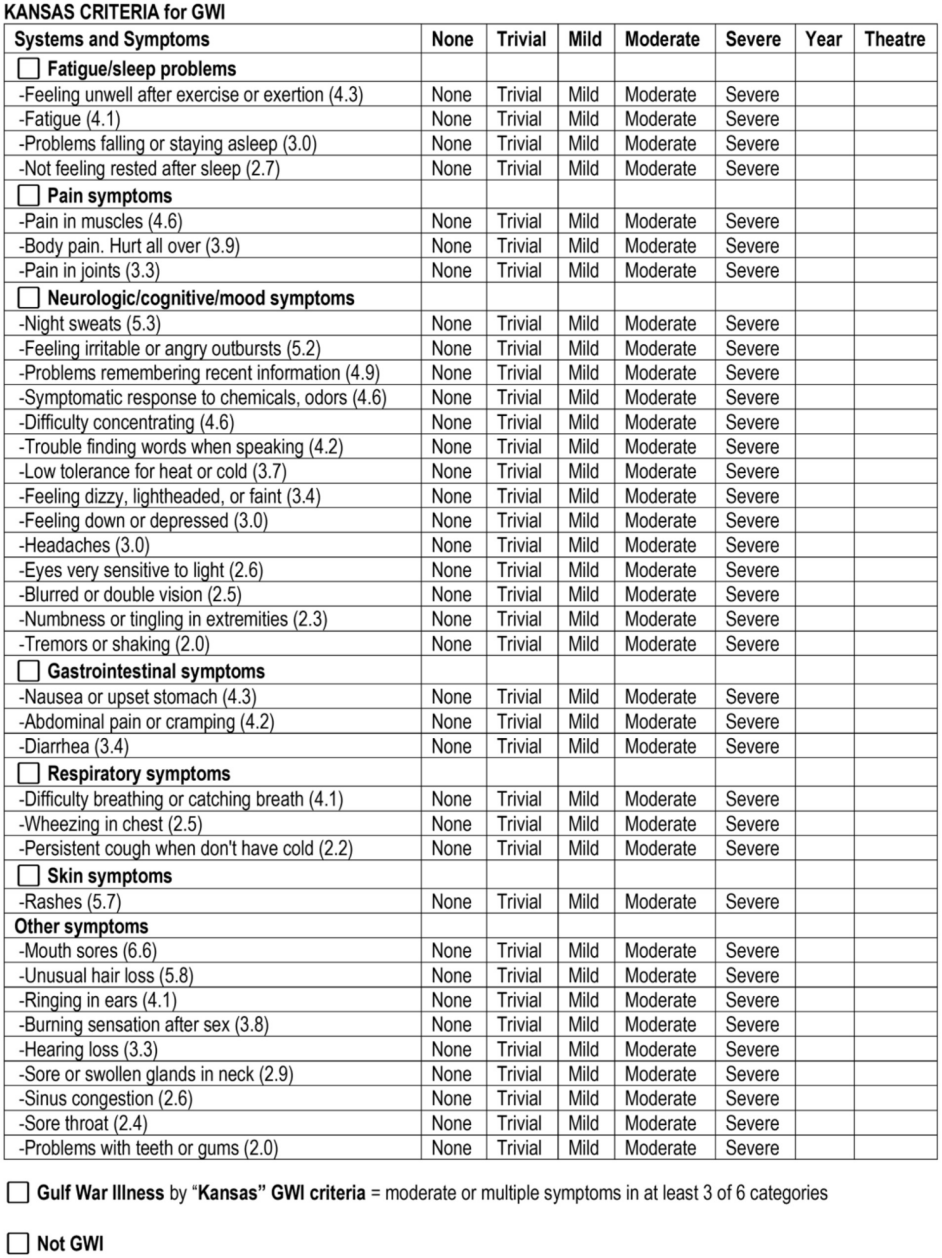
Kansas criteria for GWI. The Kansas criteria (Steele, 2000; Fappiano and Baraniuk, 2020) were operationalized as a questionnaire with anchored ordinal symptom scores. One moderate or severe symptom was required for each domain. At least three domains were required for GWI diagnosis. The numbers in parentheses are odds ratios for symptom prevalence in deployed vs. non-deployed veterans. Veterans reported the year of onset of their symptoms and whether the symptoms began in theater. Items can be scored as 0 = none, 1 = trivial, 2 = mild, 3 = moderate, and 4 = severe to calculate quantitative scores for each domain and total score, which were used as measures of GWI severity (Fappiano and Baraniuk, 2020).

GWI, ME/CFS, and healthy sedentary control (SC) subjects were recruited through personal contact, posters, and online advertisements and gave verbal informed consent to complete a battery of questionnaires (Baraniuk et al., 2013b). Paper versions were hand-entered in duplicate into Excel. All data were inspected for errors or missing data. Subjects provided in-person written informed consent for history and physical examinations to assess the Fukuda criteria (Fukuda et al., 1994; Figure 2) and Carruthers Canadian Consensus Criteria for ME/CFS (CCC; Carruthers et al., 2003; Carruthers, 2007; Figure 3), International Myalgic Encephalomyelitis (Carruthers et al., 2011), Systemic Exertion Intolerance Disease (SEID; Committee on the Diagnostic Criteria for Myalgic Encephalomyelitis/Chronic Fatigue Syndrome, Board on the Health of Select Populations, and Institute of Medicine, 2015), Chronic Multisymptom Illness (CMI; Fukuda et al., 1998; Figure 4), and Kansas (Steele, 2000; Figure 5) criteria, as well as confirmation of sedentary lifestyles (< 40 min of aerobic activity per week). Subjects were excluded because of serious chronic medical or psychiatric conditions such as psychosis (Gifford et al., 2021; Reeves et al., 2003; Jones et al., 2009; Nater et al., 2009). A history of posttraumatic stress disorder (PTSD) or depression was not an exclusion unless the subject had been hospitalized in the previous 5 years.

ME/CFS was defined by Carruthers' Canadian Consensus Criteria (CCC; Carruthers et al., 2003; Carruthers, 2007; Figure 3). The CCC requires the presence of chronic fatigue, disability, sleep disturbances, cognitive issues, PEM, and a range of diverse interoceptive complaints. GWI was defined by military service and by meeting both the CMI (Fukuda et al., 1998; Figure 4) and Kansas (Steele, 2000) criteria (Figure 5). The Kansas and CCC criteria incorporate a broad spectrum of interoceptive symptoms in contrast to the limited scope of the SEID and CMI criteria.

CMI requires two of three sets of complaints: fatigue, musculoskeletal pain, and cognitive/mood complaints. Symptoms are scored as present or absent, which leads to low specificity in the general population. To improve specificity, we required moderate or severe severity for CMI and the other criteria in our studies.

The Kansas criteria require symptoms in at least three of six domains (Figure 5). Note that it is possible to meet CMI and Kansas criteria without having fatigue.

Sedentary controls included civilians and healthy veterans who did not meet the CCC, Kansas, or CMI diagnostic criteria.

Fibromyalgia was evaluated by widespread pain, tender point counts, and the 1990, 2010, 2011, and 2016 criteria, as well as dolorimetry (Wolfe et al., 2010, 2011, 2016).

The questionnaires and physical exam allowed parallel analysis of central sensitization and nociplastic changes by dolorimetry; the number of tender points by thumb pressure at the traditional 18 tender points (Bennett, 1981); nociceptive complaints by widespread pain above and below the waist, in the axial skeleton, and on the left and right (Wolfe et al., 2010); the number of painful regions from the 2010 FM criteria (Wolfe et al., 2011); pain symptoms; fatigue; interoceptive complaints; and quality of life.

### Dolorimetry

Dolorimetry was performed with a strain gauge (DPP gauge; Chatillon Products, Ametek Inc, Largo, FL) fitted with a 1 cm^2^ rubber stopper, with pressure applied at a rate of 0.5–1 kg/s against the 18 traditional tender points (Naranch et al., 2002; Surian and Baraniuk, 2020; Bennett, 1981). The outcome point was the pressure that caused the subject to state that they were experiencing pain. A key aspect was to ensure that the patient felt in control of the process and trusted that the operator would stop pressing as soon as they indicated that pain had developed. The mean of the 18 measurements was the dolorimetry pressure threshold. The coefficient of variability for dolorimetry was 9.3% for 57 women and 12.5% for 58 men who had serial measurements on 3 consecutive days by different staff members (Surian and Baraniuk, 2020). The Pearson correlation coefficient between thumb pressure tender point counts and dolorimetry pressure thresholds was −0.862 (explained variance = 0.742).

### Questionnaires and domain scores

Symptoms were quantified using validated questionnaires.

Disability and impairment were assessed based on quality of life and the Medical Outcomes Survey Short Form 36 (SF-36; Ware and Sherbourne, 1992; McHorney et al., 1994). Responses were converted from nominal and anchored ordinal scores to scales from 0 (severely impaired) to 100 (no impairment; Hays et al., 1993). The average of Vitality, Role Physical, and Social Functioning (SF V, RP, SF) was calculated as it consistently gave the lowest scores and was superior to individual domains for differentiating ME/CFS and GWI from SC.

All subjects completed the CFS Symptom Severity Questionnaire (CFSQ; Baraniuk et al., 2013a; Figure 2). The 1994 Fukuda CFS criteria (Fukuda et al., 1994) were operationalized by scoring fatigue and eight ancillary symptoms from the previous 6 months on an anchored ordinal scale with grades of none = 0, trivial = 1, mild = 2, moderate = 3, and severe = 4. Unlike the original Fukuda criteria, we required moderate or severe symptom severities for fatigue and at least four of the eight criteria to be considered for ME/CFS diagnosis here.

Fatigue was corroborated using independent scales. The Revised Clinical Interview Schedule (CIS-R; Lewis et al., 1992) consists of six topics used for interviews and was adapted as six nominal items to gauge overall fatigue and tiredness (range 0–6). The Chalder Fatigue questionnaire was assessed as the total score summed for 11 items (range 0–33; Chalder et al., 1993). The Multidimensional Fatigue Inventory (MFI) addressed five domains with ranges of 0–20 and a sum (0–100; Smets et al., 1995).

The McGill Pain Questionnaire (Melzack, 1987) scored 11 “Sensory” pain descriptors and four “Affective” words (tiring, sickening, fearful, punishing). Severity was graded on an anchored ordinal scale: none = 0, mild = 1, moderate = 2, and severe = 3, and summed for “Sensory” (range 0–33), “Affective” (range 0–12), and “Total” (range 0–45) scores.

**Figure 6:**
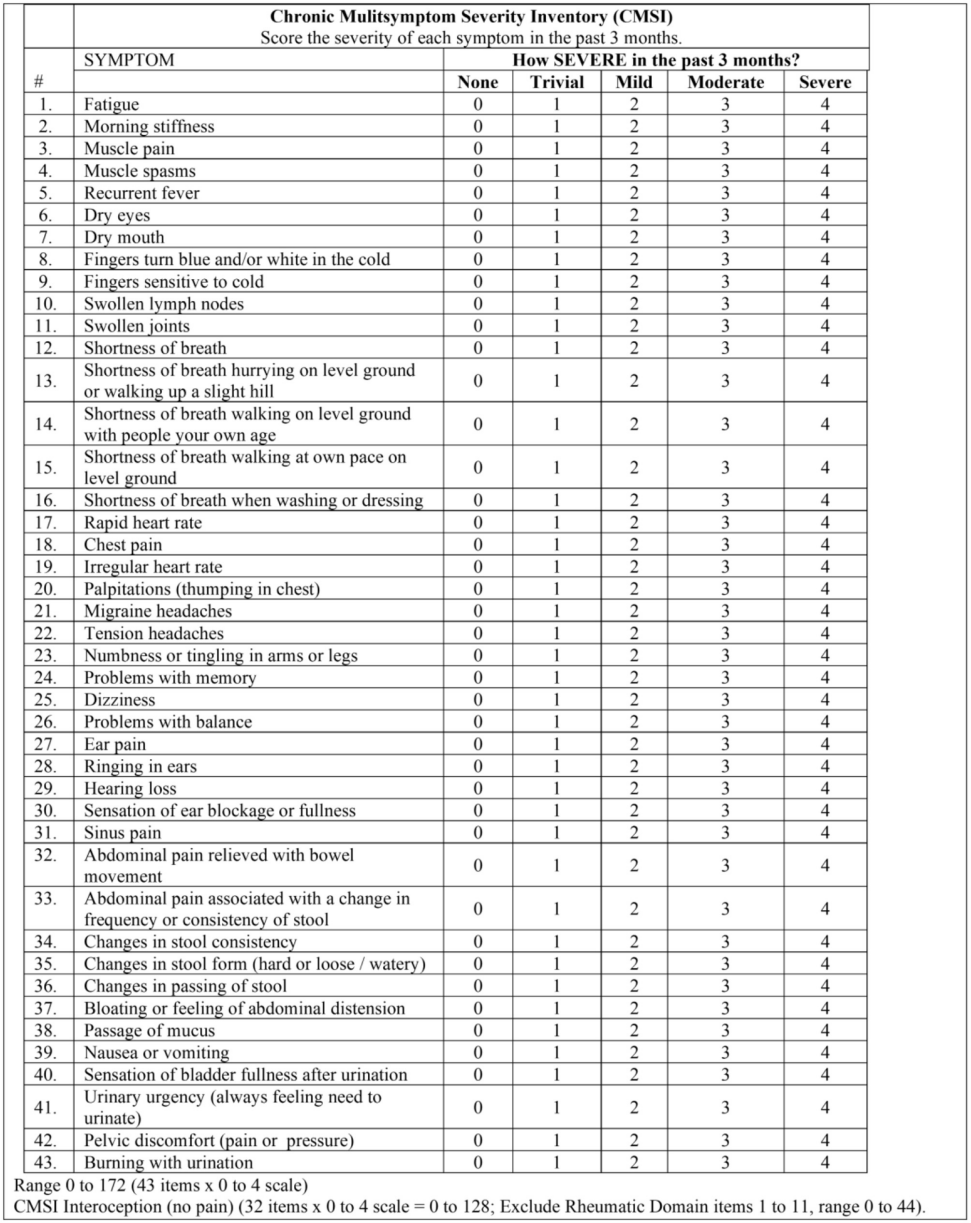
Chronic multisymptom severity inventory (CMSI).

Interoceptive symptoms were assessed from several questionnaires. The Chronic Multisymptom Severity Inventory (CMSI) assessed interoceptive symptoms using a 0–4 point anchored ordinal scale (Baraniuk et al., 1998; Figure 6). Domain scores were determined for Rheumatic, which included pain and fatigue symptoms (range 0–44), dyspnea (range 0–20), cardiac (range 0–16), headache (migraine and tension scored 0–4 each), ear sinus (range 0–20), neuro (range 0–16), irritable bowel syndrome based on Rome I criteria (range 0–32), bladder (range 0–16), and the sum of all items (range 0–172). An Interoceptive CMSI score (CMSI no pain, range 0–128) was calculated by subtracting the Rheum domain from the sum of the CMSI. The Dyspnea score (Ravindran et al., 2012) was compared to the UCSD Dyspnea scale (Eakin et al., 1998). A second questionnaire based on the Rome I criteria for irritable bowel syndrome (Kurland et al., 2006) was used for gastrointestinal symptoms. Migraines were assessed by International Headache Society criteria [Headache Classification Committee of the International Headache Society (IHS), 2013].

Upper and lower airway symptoms were assessed using the Rhinitis Score (Baraniuk et al., 1998) and Irritant Rhinitis Score (Baraniuk et al., 2000). Systemic irritant symptoms were assessed using the Chemical Exposures questionnaire domain scores (Miller and Prihoda, 1999). The Composite Autonomic Symptom Score (COMPASS-31) graded symptoms conveyed by cranial nerve, autonomic, and general afferent system pathways that are commonly associated with autonomic dysfunction (Sletten et al., 2012).

Psychiatric disorders were screened by the PRIMEMD questionnaire for major depressive syndrome, other depressive syndrome, panic syndrome, and difficulty completing the questionnaire (Spitzer et al., 1999). Generalized anxiety disorder (GAD) and major depression were assessed using the Diagnostic and Statistical Manual (DSM) 5 (American Psychiatric Association, 2013) and International Classification of Diseases version 10.^1^ Details of anxiety were examined with the Generalized Anxiety Disorder 7 questionnaire (GAD7; Kroenke et al., 2007), Mood and Anxiety Questionnaire (MASQ; Wardenaar et al., 2010; Schalet et al., 2014), and The Irritability Questionnaire (Craig et al., 2008). Major depression (Martin et al., 2017) and Somatic, Anhedonia, and Depressed domains were probed with the Center for Epidemiology—Depression questionnaire (Radloff, 1977; Geisser et al., 1997). Scores ≥16 out of 60 have been used in the past to infer the risk of major depression. However, the total score is biased by somatic complaints such as fatigue, which are common in both major depressive disorder and the general population (Fuhrer and Wessely, 1995) but are also inherent to ME/CFS and GWI criteria and diagnosis.

Psychological aspects of pain were examined with the Pain Beliefs and Perceptions questionnaire (Williams et al., 1994), the Beliefs in Pain Control Questionnaire (BPCQ; Brown, 2004), the Pain Catastrophizing Scale (Sullivan et al., 1995), and “Your Experiences with Pain” (Chronic Pain Stressor Scale, CPSS; Anderson et al., 1995).

Posttraumatic stress disorder (PTSD) was assessed by DSM5 criteria, the PTSD Checklist Civilian (PCL-C; Conybeare et al., 2012), and the Mississippi Posttraumatic Stress Disorder Scale for Gulf War Illness (M-PTSD-GWI; Holmes et al., 1998). The Big Five Personality Inventory assessed personality domains (Costa and McCrae, 2008).

### Statistical analysis

Subjects were classified in two ways for statistical analysis.

First, all subjects were classified as CCC-positive ME/CFS, CMI, and Kansas-positive GWI or SC (all other subjects).

Second, CIF was assessed by “quadrant analysis.” Symptoms related to the 1994 Fukuda criteria (Fukuda et al., 1994) were quantified by the CFS symptom severity questionnaire (CFSQ; Baraniuk et al., 2013a; Figure 2). Symptoms were dichotomized as absent (none, trivial, mild) or present (moderate or severe). The severities of each of the eight ancillary symptoms were dichotomized, and the number that were “present” was counted. Fatigue and the number of other significant symptoms were charted on a 2 × 2 matrix. Sedentary controls (SC) were defined by absent fatigue and ≤ 3 other criteria. Chronic Fatigue Syndrome-like with insufficient fatigue syndrome (CFSLWIFS) had absent fatigue and ≥4 of 8 ancillary criteria. CFSLWIFS would have met CFS criteria if fatigue had been more severe. CFS was defined in this Fukuda-based paradigm as significant fatigue plus ≥4 of 8 ancillary criteria and so included ME/CFS subjects defined by CCC. Most CMI+Kansas GWI subjects fit into the CFS category, although some with fewer or less severe complaints were classified into the CIF groups.

Dolorimetry thresholds and numbers of tender points (painful with about 4 kg thumb pressure) were tested as the dependent variables in univariate general linear modeling to assess significant confounders and the impacts of gender, race, and Hispanic status as fixed factors, with independent variables of age, body mass index (BMI), ME/CFS status, PTSD, Type II diabetes mellitus, FM by 1990 criteria, questionnaire items, and domain scores. An iterative process was used to remove non-significant variables. This confirmed the significant difference between female and male subjects (Surian and Baraniuk, 2020). Therefore, dolorimetry outcomes were analyzed separately for each gender.

Some subjects skipped items on paper questionnaires (< 0.1%). Data were imputed from other questionnaires covering the same topic for that individual, the average of their other answers on the same questionnaire, or the average for the appropriate control, ME/CFS, or GWI group. Some questionnaires were added or deleted in various versions of the protocols and were completed by fewer subjects. Questionnaires completed by fewer than 100 subjects were removed.

Significant differences between groups for each variable were found by ANOVA followed by Tukey Honest Significant Difference (*p* < 1.7 × 10^−4^) to correct for multiple comparisons between groups, then Bonferroni correction for the 300 variables that were assessed (*p* < 0.05). Means were reported with standard deviation or 95% confidence intervals. Hedges' g was calculated for significant differences between groups (Meta Essential software; Suurmond et al., 2017). Analysis of nominal outcomes was by Fisher Exact Test using VassarStats.^2^ Spearman correlations were calculated for the continuous, ordinal, and nominal data with *p*-values corrected for the number of variables. Correlations with |R| > 0.336 (explained variance *R*^2^ > 11%) had Bonferroni corrected *p*-values < 0.03. Correlations with |R| > 0.4 (*R*^2^ > 16%) had *p* < 0.001, and |R| > 0.5 (*R*^2^ > 25%) had *p* < 10^−6^.

Principal component analysis in females, males and the whole cohort used Ward's method with Varimax rotation and Kaiser normalization in SPSS version 29.^3^

Questionnaire outcomes were found to be equivalent in females and males and were thus pooled and recalculated for all subjects. Receiver operating characteristics (ROC) were determined for comparisons of SC vs. CIF, GWI, and ME/CFS groups. Thresholds were evaluated as potential diagnostic points and to define the ranges of normal.

## Results

Our objective was to determine if the physical sign of tenderness, a biomarker of central hypersensitivity, was correlated with demographics, fatigue, disability, interoceptive, nociceptive, or affective symptom profiles based on self-report questionnaire results. Subjects were classified by two algorithms to first compare SC vs. ME/CFS vs. GWI, then by quadrant analysis (Figure 2) to select CIF and compare SC, CIF, ME/CFS, and GWI. The latter provided insights into the characteristics of the CIF subgroup. Differences were determined by ANOVA. Thresholds for differentiating between control, CIF, ME/CFS, and GWI were determined by receiver operating characteristics (ROC). Spearman correlations assessed relationships between variables. Women were more tender than men, so correlations and PCA that included dolorimetry outcomes were investigated separately for each gender. Questionnaire responses were equivalent between men and women and were also pooled to find relationships between subjective complaints.

### SC vs. ME/CFS vs. GWI

Tenderness was assessed using dolorimetry data and a univariate general linear model with age, gender, BMI, somatic, and interoceptive symptom scores as independent variables. Males (4.4 kg [4.0–4.7, 95% CI]) had significantly higher average pressure thresholds than females (3.1 kg [2.7–3.4], *p* = 1 × 10^−6^). Therefore, male and female data regarding tenderness were analyzed separately to remove the gender confound.

The initial analysis contrasted SC vs. ME/CFS vs. GWI in males and females in order to simulate the usual circumstance in clinical research when CIF subjects are not identified. Pressure thresholds in females were higher for SC (4.2 kg ± 1.9, mean ± SD, *n* = 26) than ME/CFS (2.9 ± 1.4, *n* = 63, *p* = 0.0045 by two-tailed unpaired Student's *t*-test) and GWI (2.5 ± 1.4, *n* = 51, *p* = 0.000031). Male controls (6.9 ± 1.9, *n* = 37) were higher than ME/CFS (4.2 ± 1.9, *n* = 19, *p* = 7.7 × 10^−6^) and GWI (4.0 ± 2.2, *n* = 116, *p* = 8.4 × 10^−11^).

**Figure 7:**
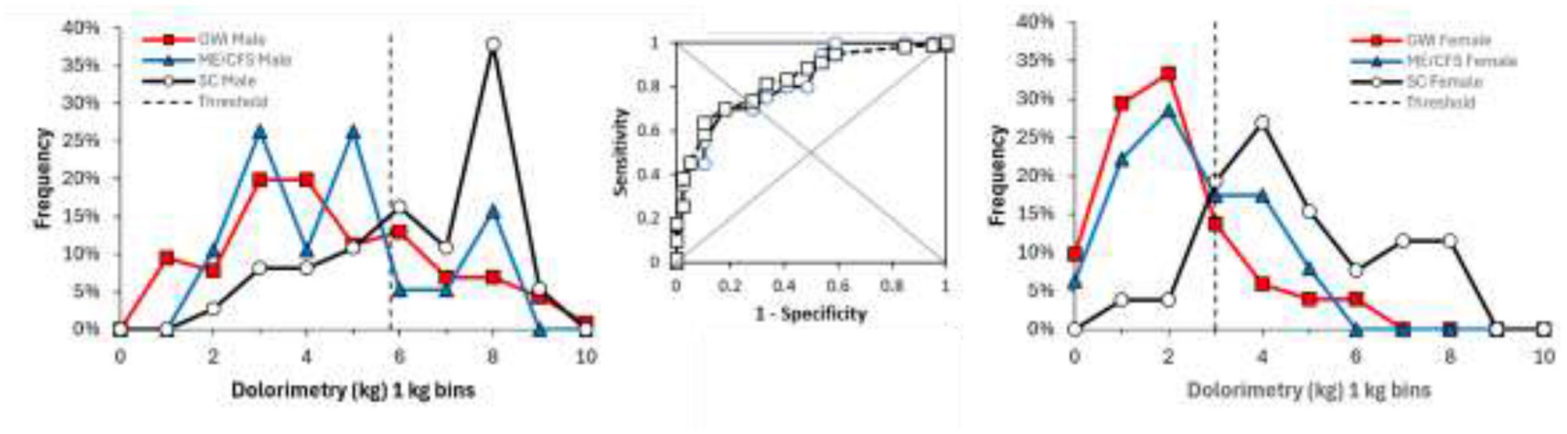
Frequency analysis for dolorimetry. The distributions of average dolorimetry pressures in males **(left)** and females **(right)** were plotted for sedentary control (SC, black line, white squares), ME/CFS (blue triangles and line), and GWI (red squares and line) with bins of 1 kg. The vertical line at 5.8 kg for males and 3.0 kg for females represents the ROC threshold for abnormal pressure sensitivity for GWI and ME/CFS vs. SC, respectively.

Frequency analysis for dolorimetry in males showed widely separated modes between sedentary control (8 kg) and ME/CFS (2 kg) and GWI (3 kg; Figure 7). Receiver operating characteristics showed reasonable AUC values >0.8 but with sensitivity and specificity < 80% and thresholds of about 5.5 kg despite the widely spaced modes (Table 1).

**Table 1:** Receiver operating characteristics for dolorimetry in females and males between SC, ME/CFS, and GWI.

		**Threshold**	**Sensitivity**	**Specificity**	**AUC**
**Dolorimetry (kg)**					
Female	SC vs. ME/CFS	3.2	0.654	0.650	0.718
	SC vs. GWI	3.0	0.731	0.729167	0.782
Male	SC vs. ME/CFS	5.8	0.765	0.778	0.854
	SC vs. GWI	5.7	0.765	0.763	0.847
**Tender point counts (0–18)**					
Female	SC vs. ME/CFS	6.8	0.692	0.717	0.753
	SC vs. GWI	9.5	0.769	0.771	0.851
Male	SC vs. ME/CFS	0.85	0.579	0.649	0.646
	SC vs. GWI	3.1	0.794	0.798	0.863

SC females had two modes at 4 and 7 kg compared to 3 kg in ME/CFS and GWI. As a result, the ROC threshold was about 3 kg and did not separate female groups with high sensitivity, specificity, or AUC.

To explain the long leftward tail of the dolorimetry distribution in SC males and the two modes in SC females, we proposed that CIF subjects contributed to overlapping distributions. Therefore, dolorimetry was compared between SC, CIF ME/CFS, and GWI (see below).

ROC for tender point counts in males found low thresholds that were consistent with minimal tenderness in SC males and a floor effect. The threshold for females was higher. Sensitivities and specificities were < 0.8, which limited the value of dolorimetry as a diagnostic tool for ME/CFS and GWI.

The SC, ME/CFS, and GWI groups were contrasted for fatigue, interoceptive, nociceptive, psychological, and quality of life scores. Data were assessed by ANOVA with Tukey and Bonferroni corrections for multiple comparisons. Hedges' g was calculated to facilitate sample size estimations for future comparison studies. Results were summarized here with data reported in Supplementary Table S1, 2.

The best discriminators for GWI>ME/CFS>SC males were aching pain on the McGill questionnaire and muscle pain by CFSQ.

GWI and ME/CFS men had equivalent dolorimetry pressure thresholds that were significantly lower than SC (GWI=ME/CFS>SC). GWI and ME/CFS had significantly elevated scores for core items of the CFSQ for Fukuda diagnosis of ME/CFS, Chalder Fatigue, and McGill tiring sensation. GWI were tested for ME/CFS diagnoses and satisfied the CCC (93.3%) and 1994 Fukuda (90.9%) criteria. Disability was evident from low SF36 scores on domains such as Vitality, Role Physical, and Social Functioning.

GWI had more tender points and widespread pain consistent with the 1990 FM criteria than ME/CFS and SC (GWI>ME/CFS=SC). Scores were higher for McGill Total, Sensory and Affective scores, interoception by CMSI, and autonomic dysfunction by COMPASS. GWI had a rate of 70% for Rome I criteria for irritable bowel syndrome and elevated Helplessness scores on the Pain Catastrophizing Scale.

Symptoms that were higher in GWI than in SC men, with intermediate, non-significant levels in ME/CFS (GWI>SC), included interoception by CMSI domain scores, irritant sensitivities by the Chemical Exposures questionnaire, and rates of migraine by International Headache Society criteria. Rates of PTSD were higher using the PCL-C and M-PTSD survey tools. The M-PTSD threshold score of 94 was exceeded by 57.3% of GWI veterans. Generalized anxiety disorder was inferred from elevated GAD7, Irritability, and Anxious Arousal scores. Traditionally, elevated CESD scores suggest a risk for depression, but in GWI, the Somatic Factor had the highest scores, which was consistent with the interoceptive profile.

Symptoms in females were comparable to those in males (Supplementary Table S2). McGill Total, Sensory, and Affective scores were graded GWI>ME/CFS>SC. The GWI and ME/CFS groups had higher Chalder Fatigue, MDFI domains, CISR scores for tiredness, interoceptive symptoms on the CESD Somatic Factor, CMSI domains, rhinitis, and irritable bowel syndrome scores, as well as disability by SF36.

Principal component analysis was performed separately for males and females with all data, as well as for GWI, ME/CFS, and SC subgroups. Data are summarized here, with full outcomes discussed in SOM PCA. Component 1 for all males included SF36 domains, CFSQ scores, McGill scores, and interoceptive symptom domains (20% variance; Supplementary Table S3). Component 1 in ME/CFS males had McGill Affective score and Chemical Exposures domains (35% variance; Supplementary Table S4). Component 1 in GWI males had anxiety, catastrophizing, anhedonia, and depression (10% variance; Supplementary Table S5). SC males had CFSQ, CESD, and SF36 domains in their initial component (43% variance; Supplementary Table S6).

The first PCA component for all women included disability, fatigue, CISR tiredness, PEM, cognition, and sleep (15% variance; Supplementary Table S7). Component 1 for ME/CFS females included the 1990 FM diagnosis and McGill items (8%; Supplementary Table S8). GWI women had catastrophizing items (12% variance, Supplementary Table S9), similar to the GWI males. SC females had McGill scores in their first component (21% variance; Supplementary Table S10). Overall, the PCA components were comparable for females and males and did not reveal any latent factors with these variables.

Spearman correlations were calculated separately for females and males for dolorimetry and questionnaire measures because of the sexual dimorphism in dolorimetry (Figure 7). Proxies were chosen for central sensitization (dolorimetry), fatigue (Chalder Fatigue score), pain (McGill Total Pain score), interoception (CMSI sum minus the Rheum domain to remove pain and fatigue items, CMSI no pain), and quality of life (average of SF36 Vitality, Role Physical, and Social Functioning domains). These questionnaires were completed by the largest number of subjects.

In females and males, the strongest correlations were found between McGill Total Pain and CMSI interoceptive complaints (*R* = 0.739 and *R* = 0.801, respectively, explained variances 0.55 and 0.64, respectively; Table 2). Both scores were correlated with dolorimetry (kg) and had explained variances of 0.17–0.40. Disability, fatigue, interoception, and pain were correlated with explained variances of 0.21–0.47. Correlations were slightly higher in males. Dolorimetry was weakly associated with fatigue and quality of life (explained variances 0.055–0.16). The variables were not highly correlated with each other (*R* < 0.85), making it unlikely that they hid an underlying latent factor. This corroborated the disparate PCA results that did not coalesce symptom scores into novel factors.

**Table 2:** Spearman correlations for females and males.

	**Dolorimetry (kg)**	**Chalder fatigue**	**SF36 V, RP, SF**	**CMSI (no pain)**	**McGill total**
**Female spearman**					
Dolorimetry (kg)	1	−0.234^*^	0.282^*^	−0.417	−0.629
Chalder Fatigue	−0.234^*^	1	−0.643	0.546	0.519
SF36 V, RP, SF	0.282^*^	−0.643	1	−0.498	−0.453
CMSI (no pain)	−0.417	0.546	−0.498	1	0.739
McGill total	−0.629	0.519	−0.453	0.739	1
**Male spearman**					
Dolorimetry (kg)	1	−0.336	0.398	−0.574	−0.545
Chalder fatigue	−0.336	1	−0.687	0.679	0.587
SF36 V, RP, SF	0.398	−0.687	1	−0.656	−0.622
CMSI (no pain)	−0.574	0.679	−0.656	1	0.801
McGill total	−0.545	0.587	−0.622	0.801	1

Proxies were chosen for central sensitization (dolorimetry), fatigue (Chalder Fatigue score), pain (McGill Total Pain score), interoception (CMSI “no pain” = CMSI total score sum minus the rheum domain to remove pain and fatigue items), and quality of life (average of SF36 Vitality, Role Physical, and Social Functioning domains) that covered the largest number of subjects. Correlation with |R| > 0.336 had Bonferroni corrected p-values < 0.03. Correlations with |R| > 0.4 had p < 0.001 and |R| > 0.5 had p < 10^−6^. ^*^Not significant.

Relationships between dolorimetry, tender point counts, and diagnosis of FM by 1990 criteria were evaluated in both genders. For women, dolorimetry and tender point counts were moderately correlated (*R* = −0.623; Table 3). FM 1990 diagnosis required both tender points (*R* = 0.733) and widespread pain (*R* = 0.528). The physical signs were correlated with McGill and other nociceptive scores (|R| = 0.42–0.64). Measures of fatigue, interoception, affect, and disability were weakly correlated (|R| < 0.4).

**Table 3:** Spearman correlations for dolorimetry with |R| > 0.5 (*R*^2^> 0.25, *p* < 10^−6^ after Bonferroni correction) for women.

**Spearman's rho**	**Dolorimetry**	**Tender point count**	**≥11/18 tender points**	**FM 1990**
Dolorimetry	1	−0.623	−0.487	−0.541
Tender point count	−0.623	1	0.79	0.733
Count ≥11/18	−0.487	0.79	1	0.797
FM 1990	−0.541	0.733	0.797	1
Widespread pain	−0.523	0.587	0.57	0.528
McGill total	−0.629	0.643	0.577	0.586
McGill sensory	−0.593	0.632	0.57	0.564
McGill affective	−0.592	0.583	0.534	0.565
McGill aching	−0.42	0.492	0.507	0.493
McGill sickening	−0.533	0.502	0.447	0.424
McGill tenderness	−0.453	0.452	0.403	0.517
McGill tiring	−0.503	0.518	0.479	0.577
CFSQ muscle pain	−0.59	0.566	0.444	0.493
CFSQ joint pain	−0.545	0.511	0.426	0.439
CFSQ sum of 8	−0.538	0.527	0.386	0.477
CMSI sum	−0.459	0.555	0.417	0.453
CMSI rheum	−0.515	0.549	0.398	0.487
SF36 bodily pain	0.601	−0.512	−0.41	−0.489
Chemical domain	−0.498	0.593	0.438	0.492
Chemical symptoms	−0.477	0.587	0.467	0.515
IRS rhinorrhea	−0.363	0.524	0.489	0.461

Correlations with |R| > 0.5 (R^2^ > 0.25) had p < 10^−6^ after Bonferroni correction.

In males, dolorimetry (kg) was correlated with the number of positive tender point counts (*R* = −0.766), diagnosis by FM 1990 criteria (*R* = 0.677) and nociceptive symptoms by McGill Total Score (*R* = −0.545; Table 4). Pressures were correlated with PEM (*R* = −0.514), widespread interoceptive complaints (CMSI, *R* = −0.569) and irritable bowel syndrome (*R* = −0.538). Counting the number of tender points by thumb pressure had a similar profile of correlations. Dichotomizing the tender points to < 11 vs. ≥11 as required for the 1990 FM criteria reduced the correlations for interoceptive and McGill pain scores to < 0.5.

**Table 4:** Spearman correlations for dolorimetry with |R| > 0.5 (*R*^2^ > 0.25, *p* < 10^−6^ after Bonferroni correction) for men.

**Spearman's rho**	**Dolorimetry**	**Number of tender points**	**≥11/18 tender points**	**FM 1990**
Dolorimetry kg	1	−0.766	−0.673	−0.677
Tender point counts (0–18)	−0.766	1	0.806	0.754
Tender points ≥11/18	−0.673	0.806	1	0.811
FM 1990 (%)	−0.677	0.754	0.811	1
McGill total score	−0.545	0.567	0.488	0.488
McGill sensory score	−0.561	0.579	0.494	0.493
McGill tender	−0.539	0.583	0.541	0.495
McGill heavy	−0.525	0.438	0.411	0.454
McGill throbbing	−0.465	0.507	0.475	0.442
CFS by Fukuda criteria	−0.542	0.548	0.465	0.434
CFSQ sum of 8 ancillary criteria	−0.524	0.536	0.478	0.46
CFSQ PEM	−0.514	0.476	0.362	0.409
CFSQ headache	−0.501	0.548	0.447	0.368
CMSI sum	−0.569	0.599	0.498	0.476
CMSI GI	−0.538	0.57	0.453	0.495
CMSI headache	−0.508	0.549	0.444	0.396
CMSI rheumatic	−0.49	0.556	0.474	0.422
Helplessness	−0.423	0.531	0.411	0.442

**Figure 8:**
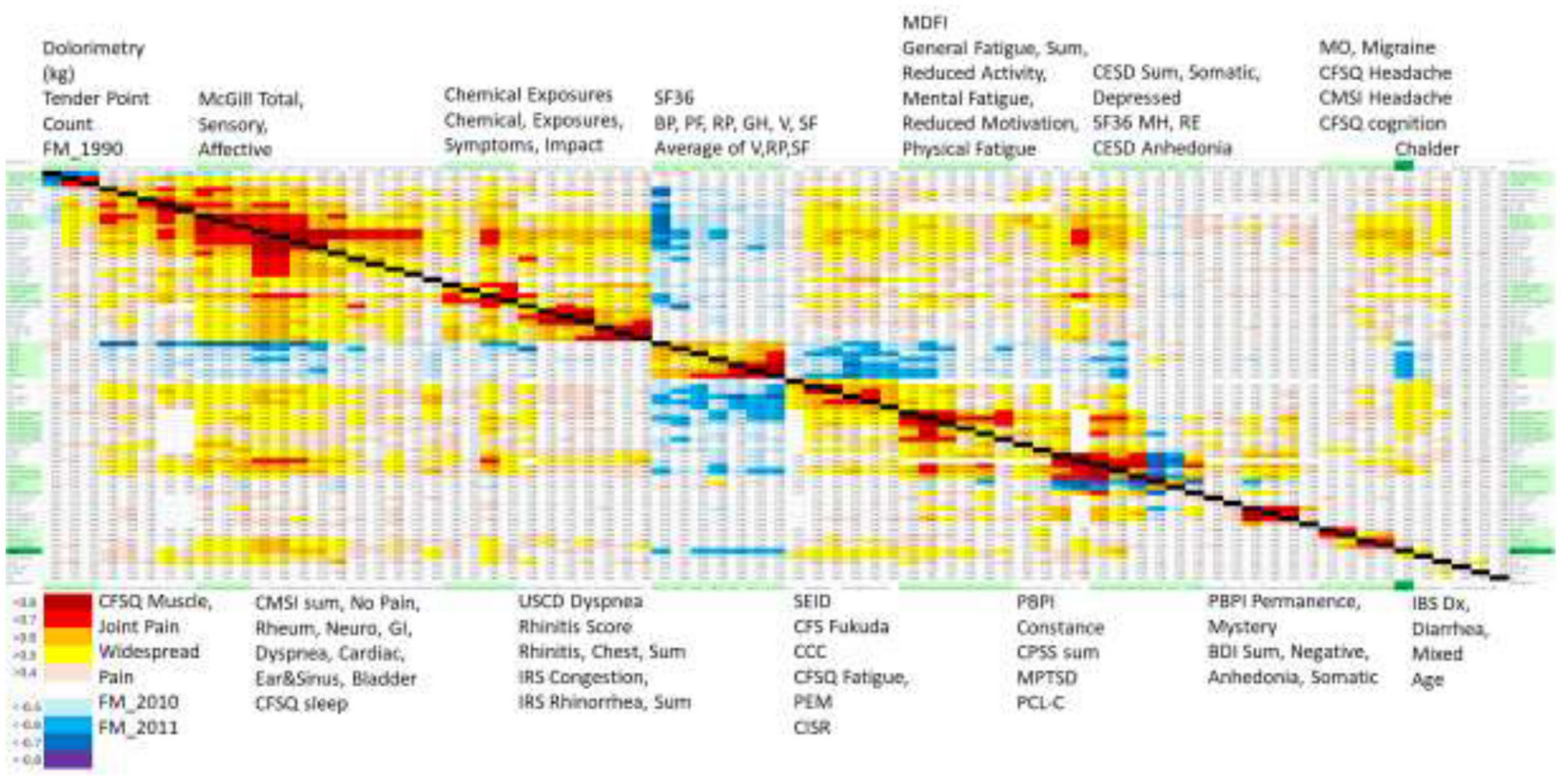
Spearman correlations for all domain data and subjects. Domains for the x and y axes are printed above and below the correlation matrix, with the left side of the upper lists aligned to the left of each green block, and those on the bottom aligned to the left of each white block. Chalder Fatigue was indicated by the dark green boxes on each side. Significant correlations were indicated in the graph with color coded probabilities in the lower left corner. Correlations with |R| > 0.5 had *p* < 10^−6^ after Bonferroni correction.

Correlations for symptoms were equivalent between men and women. Therefore, all subjects were pooled for the next level of Spearman correlations. The interesting result was the clustering of domains with |R| > 0.7 (*R*^2^ > 0.49, *p* < 10^−8^ after Bonferroni correction, red or dark blue in Figure 8). As anticipated, dolorimetry, number of tender point counts and FM by 1990 criteria were highly correlated with each other, but because of the gender difference were not anticipated to correlate with subjective outcomes for the entire group.

SF36 Bodily Pain, widespread pain and FM by 2010 criteria were the next most highly correlated group. A separate block was dominated by McGill Total Pain and CMSI total and its no pain and Rheum subscores.

Rhinitis domains were intercorrelated and also correlated with CMSI score (*R* = 0.6–0.7) consistent with the interoceptive complaints.

SF36 Vitality, Social Functioning, and Role Physical that assess quality of life were highly correlated with CFSQ Fatigue and PEM. This provides evidence for the large impact of fatigue and PEM on quality of life. The next hotspot included the MDI General Fatigue and Reduced Activity domains with SF36 Vitality.

A psychological grouping included MPTSD, PCL-C, CESD Somatic, Depressed and total score, and SF36 Mental Health and Role Emotion. Other separate sets with high correlations were for the BDI depression domains and headaches including migraine without aura. It was surprising that the Chalder Fatigue score had lower correlations (R~0.6) with CESD Somatic Factor and SF36 Vitality, Social Function, Role Physical and Bodily Pain, and poor correlation with other fatigue domains.

The themes with the highest sets of intercorrelations were tenderness, pain (McGill), interoception, and quality of life with fatigue and PEM. The MDFI and Chalder fatigue scores were not highly correlated with pain or interoceptive variables.

Clustering of Spearman correlations was similar for ME/CFS, GWI, and SC (Supplementary Table S11). Correlations with |R| > 0.5 were not as frequent as for the entire dataset, but the most highly correlated domains with |R| > 0.7 were essentially the same. Most correlations were within questionnaire domains rather than between questionnaires. The Dolorimetry outcomes were again independent as anticipated by the mix of female and male subjects. Summary scores for McGill and CMSI were correlated, followed by clusters for Chemical Exposures, Rhinitis, SF36, CESD and PCS domains.

**Figure 9:**
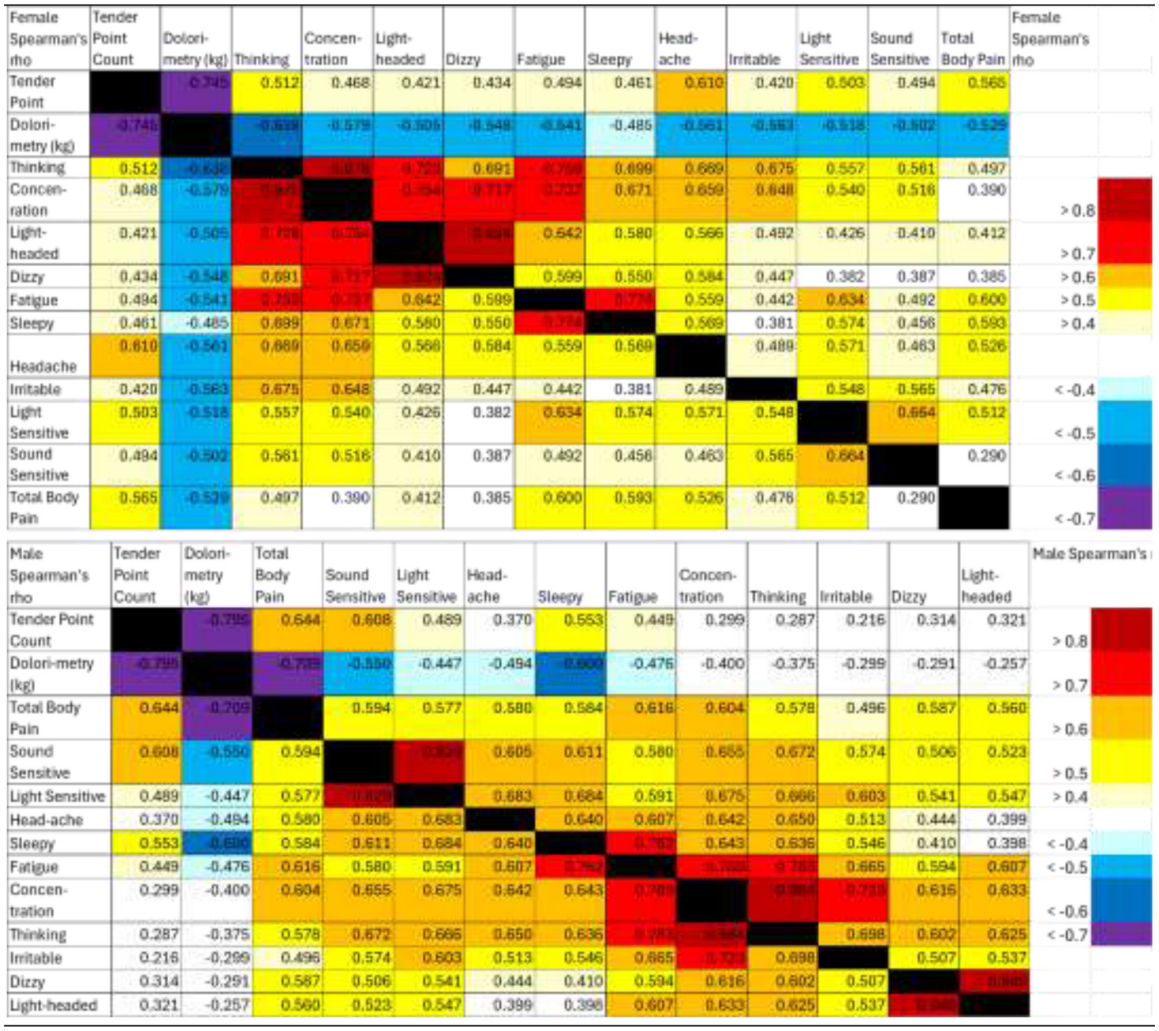
Spearman's rho for measures of tenderness with other symptoms in females and males while standing up.

Orthostatic intolerance was examined using Spearman correlations between dolorimetry, tender point counts, and 11 symptoms for a subset of 25 SC, 38 ME/CFS, and 56 GWI subjects who were assessed while standing up and recumbent (Figure 9). Symptoms were quantified by 20-point anchored ordinal Gracely Box Scores (Garner and Baraniuk, 2019). Females and males had different patterns of correlations. Dolorimetry (kg) in females (*n* = 51) was correlated with many symptoms (|R| > 0.5), including the block of concentration, problems thinking, lightheadedness, dizziness, and fatigue. In general, correlations with dolorimetry were higher in females than in males.

Upright males (*n* = 68) had high correlations of dolorimetry, tender point counts, and total body pain (|R| > 0.7). Sound and light sensitivities were highly correlated with each other (|R| > 0.8). The block of concentration, problems thinking, fatigue, and irritability had |R| > 0.7 but poor correlation with measures of tenderness (|R| < 0.5). Sleepiness was correlated with tenderness. Dizziness and lightheadedness were correlated with each other but not with tenderness, suggesting a weak relationship between antinociceptive mechanisms and vestibular symptoms. Correlations were comparable when subjects were recumbent. The incremental orthostatic change between recumbent and standing was not correlated with tenderness.

### Chronic idiopathic fatigue

Chronic Idiopathic Fatigue (CIF) is rarely considered in the differential diagnosis of ME/CFS or GWI. However, if the severity of diagnostic criteria is scored as present or absent, then overlap may occur.

Female and male subgroups were examined to characterize CIF in each gender and determine ranges of scores for CIF diagnosis that would significantly differentiate CIF from controls at one end of each scale and from GWI and ME/CFS at the other end. All subjects were pooled, then CFSQ and Fukuda criteria were applied in “quadrant analysis” with 2 × 2 factorial tables cross-referencing fatigue vs. other ME/CFS criteria at moderate or severe levels (Figure 2; Baraniuk et al., 2013a).

CIF was defined by moderate or severe fatigue but with ≤ 3 other criteria. Sedentary controls (SC) had no fatigue and ≤ 3 of the other 8 criteria. CFS-like with insufficient fatigue syndrome (CFSLWIFS) had low fatigue (none, trivial, or mild) and ≥4 of 8 ancillary criteria and thus would have met ME/CFS criteria if fatigue had been more severe. For this analysis, ME/CFS was defined by Fukuda criteria as moderate or severe fatigue plus ≥4 of 8 ancillary criteria. GWI met Kansas and CMI criteria. Frequency analysis was conducted for groups with n ≥16, which excluded male ME/CFS and CFSLWIFS and female CFSLWIFS groups. Therefore, ANOVA assessed male SC vs. CIF vs. GWI, and female SC vs. CIF vs. ME/CFS vs. GWI, with correction for multiple comparisons by Tukey's Honest Significant Difference followed by Bonferroni for 172 items (*p* < 0.05).

Male and female groups were equivalent for age, body mass index (BMI), ethnicity and race (Table 5).

**Table 5:** Demographics for CIF.

**Group**	**N**	**Age ±SD**	**BMI ±SD**	**White**	**Hispanic**
Female SC	20	41.6 ± 19.9	28.5 ± 5.8	18	1
Female CFSLWIFS	3	51.3 ± 12.1	28.9 ± 5.5	3	1
Female CIF	17	48.0 ± 13.2	28.3 ± 5.2	15	2
Female ME/CFS	51	47.4 ± 11.1	27.5 ± 6.7	45	5
Female GWI	49	50.0 ± 10.2	29.5 ± 5.8	35	6
Male SC	35	44.6 ± 14.5	28.4 ± 4.7	29	2
Male CFSLWIFS	5	51.0 ± 4.9	31.9 ± 6.4	4	0
Male CIF	25	47.2 ± 11.7	32.5 ± 6.2	22	1
Male ME/CFS	11	47.3 ± 8.7	30.5 ± 6.4	10	0
Male GWI	96	46.3 ±7.1	33.9 ± 3.5	86	3

**Figure 10:**
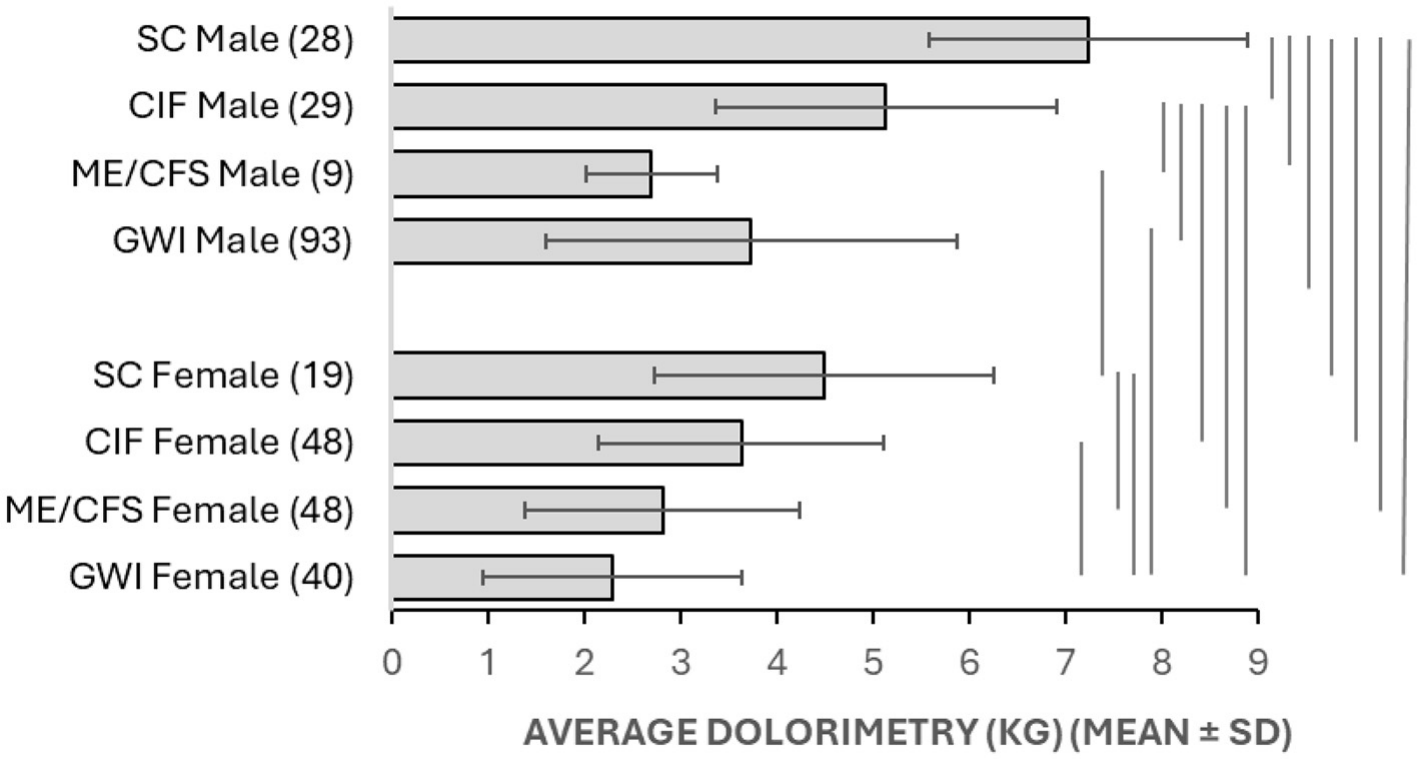
Dolorimetry. Average dolorimetry (±SD) is shown for each group in Table 5. Results that were significantly different between groups were indicated by bars at the right (*p* < 0.05 after Bonferroni corrections).

After accounting for CIF, dolorimetry thresholds were clustered into four strata based on *t*-tests. SC males were the least tender (*p* < 0.00016 vs. all other groups, Bonferroni corrected; Figure 10). Next were SC females and CIF males, followed by GWI males and CIF females. The most tender cluster included GWI females, CFS males, and females. Sexual dimorphism was apparent, with males being less tender than females for SC (*p* = 0.015 after Bonferroni correction), CIF (*p* = 0.00072), and GWI (*p* = 0.00014). The dimorphism was not seen for ME/CFS (*p* = 0.8), possibly because of the small sample size of the male subgroup.

**Figure 11:**
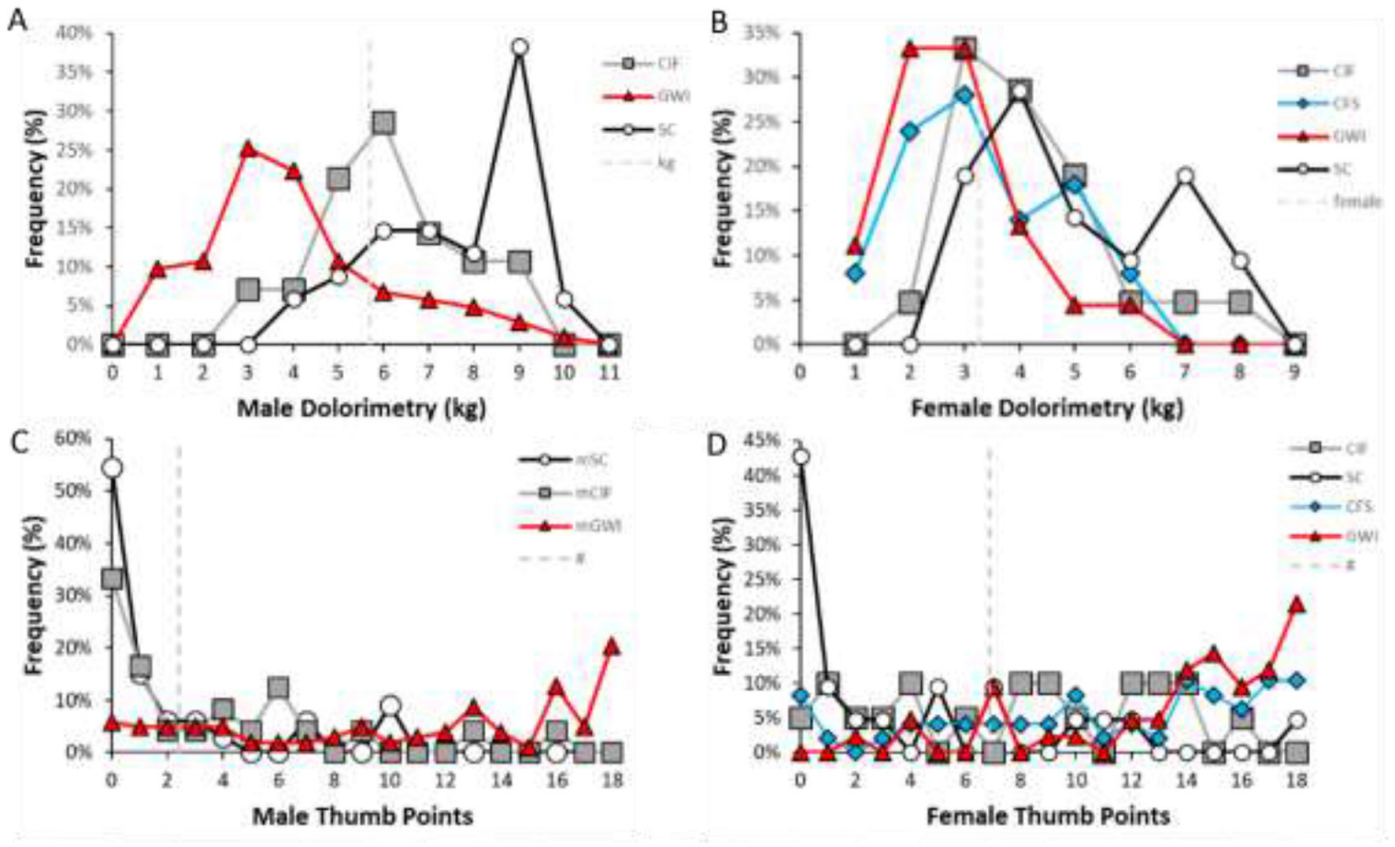
Frequency analysis for dolorimetry with CIF. Nociplastic outcomes were shown for subgroups with six or more subjects. Dolorimetry **(A)** and tender point counts **(C)** in males were compared between CIF (gray squares), GWI (red triangles), and SC (white circles, black line). Dolorimetry **(B)** and tender point counts **(D)** in females were compared between CIF (gray squares), ME/CFS (“CFS,” blue diamonds), GWI (red triangles), and SC (white circles, black line). The vertical dashed lines indicated receiver operating characteristic thresholds for SC vs. all other groups.

Male dolorimetry data were assessed by frequency analysis and ROC. Males had a mode at 9 kg that was higher than CIF (mode 6 kg; Figure 11) with an ROC threshold of 6.1 kg (AUC 0.769, sensitivity 0.714, specificity 0.719, Table 6). Therefore, the CIF subgroup explained a portion of the long left-sided tail for the SC group in Figure 7 and indicated that CIF had some element of central sensitization. The numbers of ME/CFS and CFSLWIFS males were too small to depict. GWI remained shifted to the left (mode 3 kg).

**Table 6:** ROC analysis of dolorimetry in CIF.

**Gender**	**Comparison**	**Threshold**	**Sensitivity**	**Specificity**	**AUC**
**Dolorimetry (kg)**					
Female	SC vs. CIF	3.6	0.600	0.619	0.748
	SC vs. ME/CFS	3.3	0.708	0.714	0.793
	SC vs. GWI	3.0	0.806	0.810	0.893
	CIF vs. ME/CFS	2.9	0.625	0.650	0.668
	CIF vs. GWI	2.7	0.714	0.700	0.805
Male	SC vs. CIF	6.1	0.714	0.719	0.769
	SC vs. ME/CFS	5.3	0.818	0.813	0.916
	SC vs. GWI	5.6	0.784	0.781	0.883
	CIF vs. ME/CFS	4.5	0.818	0.810	0.81
	CIF vs. GWI	4.9	0.742	0.762	0.77
**Tender point counts (0–18)**					
Female	SC vs. CIF	4.8	0.667	0.650	0.644
	SC vs. ME/CFS	6.8	0.762	0.729	0.807
	SC vs. GWI	9.5	0.810	0.833	0.921
	CIF vs. ME/CFS	9.6	0.600	0.604	0.664
	CIF vs. GWI	12.7	0.750	0.738	0.834
Male	SC vs. CIF	0.6	0.625	0.619	0.663
	SC vs. ME/CFS	1.3	0.750	0.727	0.81
	SC vs. GWI	2.8	0.875	0.866	0.914
	CIF vs. ME/CFS	2.1	0.524	0.545	0.656
	CIF vs. GWI	5.8	0.667	0.722	0.817

Thresholds for dolorimetry (kg) and tender point counts discriminated between subgroups of female and male subjects.

SC females continued to have modes at 4 and 7 kg (Figures 7, 11) that were higher than CIF (mode 3 kg), ME/CFS (mode 3 kg), and GWI (mode 2.5 kg). The ROC threshold for SC vs. CIF was 3.6 kg (AUC 0.748, sensitivity 0.600, specificity 0.619). The grouping of CIF, ME/CFS, GWI, and the lower mode of females suggested a shared trend toward central sensitization in these women.

Dolorimetry thresholds for ME/CFS vs. SC were 3.3 kg (AUC = 0.793) for females and 5.3 kg (AUC = 0.916) for males (Figure 11). GWI were more tender than SC, with thresholds of 3.0 kg (AUC = 0.893) for females and 5.6 kg (AUC = 0.883) for males. Results for ME/CFS and GWI were better when CIF subjects were accounted for (Tables 1, 6). The exquisite tenderness of male and female GWI subjects was apparent compared to both SC and CIF.

Tender point counts provided an additional perspective (Table 6, Figure 11). Most SC (55%) and CIF (33%) males had no tender points (medians 0.3 and 1.3, respectively). In contrast, GWI were shifted to the right (median 12.9), indicating significant tenderness. The ROC threshold between SC and CIF males was 2.4 tender points (AUC 0.844, sensitivity 0.758, specificity 0.767).

Tender point counts in SC females had one mode at zero tender points (43%, median 1.3; Table 6, Figure 11). CIF females had a flat distribution curve (median 8.7), indicating heterogeneity in their tenderness. In contrast, ME/CFS and GWI were shifted to the right with medians of 11.8 and 15, respectively. The patterns suggest that normal women had no tenderness (floor effect), with a transition through CIF to substantial generalized tenderness in ME/CFS and GWI (ceiling effect).

Questionnaire data for SC, CIF, ME/CFS, and GWI defined by quadrant analysis are in the SOM for males (Supplementary Table S12) and females (Supplementary Table S13). Outcomes were similar between genders, so the analysis was reported for all subjects (Supplementary Table S14).

CMI criteria were met by 73.8% of the CIF group, 93.5% of ME/CFS, and, by definition, 100% of the GWI group (Table 7). The high rates in CIF and ME/CFS show that the CMI criteria are not specific for GWI. Fatigue is the shared feature between CIF and CMI criteria. To qualify for CMI, the CIF subjects must have had either significant mood and cognition issues or musculoskeletal pain.

**Table 7:** Nominal results after quadrant analysis for CIF (numbers per group).

**Variable**	**SC**	**CIF**	**CFSLWIFS**	**ME/CFS**	**GWI**	**Chi squared *p***
CMI	10.9% (55)	73.8% (42)	87.5% (8)	93.5% (62)	100% (145)	200.47 *p* = 2 × 10^−5^
Kansas	5.5% (55)	52.4% (42)	75.0% (8)	83.9% (62)	100% (145)	189.67 *p* < 10^−10^
GWI = Kansas+CMI	5.5% (55)	52.4% (42)	62.5% (8)	83.9% (62)	100% (145)	189.67 *p* < 10^−10^
Widespread pain	10.2% (49)	46.2% (39)	87.5% (8)	65.5% (55)	85.7% (112)	85.8 *p* < 10^−8^
Tender point count ≥11/18	9.1% (55)	20.0% (40)	62.5% (8)	50.8% (61)	64.6% (144)	61.69 *p* < 10^−8^
FM 1990	0% (52)	5.4% (37)	50.0% (8)	43.5% (62)	52.5% (99)	59.06 *p* < 10^−8^
FM 2010	4.0% (25)	28.6% (21)	100% (5)	76.2% (21)	95.7% (46)	67.56 *p* = 10^−17^
FM 2011	0% (25)	20.0% (20)	75.0% (4)	69.6% (23)	84.8% (46)	57.91 *p* = 10^−15^
IBS diagnosis	9.5% (42)	32.3% (31)	50.0% (8)	43.4% (53)	80.9% (68)	57.9 *p* < 10^−8^
IBS mixed	7.1% (42)	22.6% (31)	12.5% (8)	18.9% (53)	41.2% (68)	17.91 *p* = 0.00046
IBS Rome I	11.5% (26)	14.3% (21)	40.0% (5)	34.8% (23)	76.6% (47)	39.9 *p* = 10^−9^
IBS Rome II	11.5% (26)	19.0% (21)	40.0% (5)	30.4% (23)	70.2% (47)	31.32 *p* = 10^−6^
IBS Rome III	11.5% (26)	19.0% (21)	40.0% (5)	30.4% (23)	68.1% (47)	29.09 *p* = 2 × 10^−6^
Migraine IHS	12.2% (49)	31.6% (38)	50.0% (8)	76.7% (60)	69.0% (87)	62.07 *p* < 10^−6^
Migraine without aura (MO) IHS	10.2% (49)	26.3% (38)	37.5% (8)	51.7% (60)	47.1% (87)	26.26 *p* = 8 × 10^−6^
Migraine with aura (MA) IHS	4.1% (49)	7.9% (38)	12.5% (8)	25.0% (60)	24.1% (87)	13.52 *p* = 0.00036
Multiple chemical sensitivity	3.8% (26)	4.8% (21)	20.0% (5)	24.0% (25)	36.2% (47)	14.6 *p* = 0.022
GAD DSM5	16.7% (6)	0% (7)	66.7% (3)	20.0% (5)	37.1% (35)	ns
GAD ICD10	16.7% (6)	14.3% (7)	66.7% (3)	33.3% (6)	47.1% (34)	ns
GAD7 ≥10	14.9% (47)	8.1% (37)	12.5% (8)	24.4% (45)	52.8% (127)	40.21 *p* < 10^−6^
Depression DSM IV	16.7% (6)	0% (7)	33.3% (3)	28.6% (7)	17.6% (34)	ns
PMD major depressive syndrome	2.6% (39)	18.5% (27)	25.0% (8)	21.4% (42)	46.6% (116)	31.73 *p* = 10^−6^
PMD other depressive syndrome	5.1% (39)	18.5% (27)	25.0% (8)	7.1% (42)	14.7% (116)	ns
PMD panic syndrome	0% (39)	0% (27)	0% (8)	0% (42)	0% (116)	ns
PTSD	11.5% (26)	0% (20)	75.0% (4)	30.4% (23)	60.9% (46)	31.47 *p* = 10^−6^
Veteran	27.3% (55)	38.1% (42)	50.0% (8)	6.5% (62)	100% (145)	201.33 *p* < 10^−12^
Type 2 DM	1.9% (53)	7.3% (41)	14.3% (7)	8.2% (61)	10.5% (143)	ns
CRP >3 mg/L	53.3% (45)	42.1% (38)	62.5% (8)	36.8% (57)	68.3% (104)	17.46 *p* = 0.00057

Results [% (n)] were compared between CIF, SC, ME/CFS, and GWI by 4 × 2 Chi squared (3 degrees of freedom). The CFSLWIFS group was too small for the computation.

The Kansas criteria were met by 83.9% of ME/CFS. Conversely, 95.4% of GWI met the CCC criteria (Chi squared 188.76, *p* < 10^−10^) demonstrating the overlap of symptom profiles in the two criteria.

FM by 1990 criteria is defined by complaints of widespread pain plus tender point counts ≥11/18. None of the SC group and only 5.4% of CIF met the 1990 FM criteria, in contrast to 43.5% of ME/CFS and 52.5% of GWI. The rates for 2010 and 2011 FM criteria were slightly higher.

Irritable bowel syndrome (IBS) was more prevalent in GWI (80.9%), ME/CFS (43.5%), and CIF (32.2%) than in SC (9.5%). Rates of migraine by IHS criteria followed the same stratification.

Generalized anxiety disorder (GAD) was inferred by GAD7 scores ≥10 for ME/CFS (24.4%) and GWI (52.8%). PRIMEMD suggested risks for major depression in ME/CFS and GWI. PTSD was diagnosed by history or questionnaire in 60.9% of GWI.

Criteria for CIF were defined by significant differences from SC (Supplementary Table S14) and ROC thresholds (Table 8). CIF had higher scores than SC for fatigue domains, including the Chalder Fatigue Score, CISR sum for tiredness, MDFI domains, CESD somatic factor, and CMSI rheumatological factors. Disability was apparent in impaired SF36 domains. Neurological problems were indicated by CMSI neuro, CFSQ PEM, sleep, and cognition. PBPI Constance and Mystery were elevated.

**Table 8:** ROC for CIF vs. SC for all subjects.

**CIF vs. SC**	**Threshold**	**Sensitivity**	**Specificity**	**AUC**
Chalder	16.5	0.842	0.864	0.847
CISR sum	4.5	0.895	0.864	0.907
MDFI general fatigue	14.5	0.895	0.909	0.910
MDFI mental fatigue	10.5	0.632	0.682	0.805
MDFI physical fatigue	13.0	0.737	0.773	0.929
MDFI reduced activity	11.5	0.895	0.864	0.890
MDFI reduced motivation	10.5	0.789	0.864	0.731
MDFI ΣDomains	65.0	0.895	0.909	0.900
PBPI constance	0.0	0.737	0.727	0.837
PBPI mystery	−0.6	0.842	0.818	0.836
PBPI permanence	0.3	0.737	0.727	0.830
PBPI self-blame	−1.8	0.526	0.636	0.537
SF36 (average V RP SF)	42.9	0.864	0.842	0.932
SF36 BP	68.8	0.727	0.842	0.804
SF36 GH	47.5	0.864	0.842	0.932
SF36 MH	66.0	0.773	0.789	0.873
SF36 PF	87.5	0.636	0.789	0.794
SF36 RE	83.3	0.864	0.737	0.827
SF36 RP	62.5	0.773	0.947	0.828
SF36 SF	68.8	0.818	0.842	0.916
SF36 V	32.5	0.864	0.842	0.934
CESD somatic factor	4.5	0.804	0.766	0.847
CFSQ sleep	2.5	0.717	0.760	0.802
CFSQ PEM	1.5	0.826	0.840	0.917
CFSQ cognition	1.5	0.848	0.760	0.861

Domain scores that were significantly different between groups by ANOVA after Tukey and Bonferroni corrections were evaluated for ROC. The values would separate CIF from SC groups.

At the more severe end of the scale, CIF was distinguished from ME/CFS and GWI by the elevated ROC thresholds for McGill Total and Sensory Pain indices, and SF36 Physical Functioning and Bodily Pain (Supplementary Table S15). ME/CFS and GWI had higher values for CFSQ PEM, cognition, muscle pain, joint pain, and headaches than CIF. ME/CFS was also differentiated from SC (Supplementary Table S16).

GWI had higher scores than CIF for most of the other measures, including Chalder Fatigue, McGill Affective, individual McGill pain sensations, interoceptive CMSI domain, and CESD Somatic Factor scores. Environmental irritation was indicated by irritant rhinitis and chemical exposure scores. GWI had worse psychological status, with elevated scores for M-PTSD, PCL-C, CESD and its Depressed Factor, Irritability, PCS Helplessness, Magnification and Rumination, MASQ Anxious Arousal, and General Distress and Loneliness (Supplementary Table S14).

GWI had higher scores than ME/CFS for McGill pain domains and affective symptoms, CMSI interoception, GI domains on CMSI and COMPASS, and nasal and lower airway complaints by CMSI Ear&Sinus, Irritant Rhinitis rhinorrhea and Rhinitis Scores (Supplementary Table S14).

### Correlation of dolorimetry with symptom domains

Dolorimetry was significantly different between subgroups of male and female participants. However, the data were highly skewed, leading to overlap between groups such as SC and CFS. As a result, many of the ROC thresholds did not show high specificity or sensitivity.

The relationships between nociplastic mechanisms of tenderness and symptoms of pain, fatigue, interoception, and disability were examined by Pearson correlation tests. Dolorimetry (kg) was correlated with symptom scores for females and males in each subgroup. Correlations that were significant after Bonferroni corrections (*p* < 0.05) were tabulated (Supplementary Tables S17, S18). Explained variances (*R*^2^) were reported for groups larger than 10.

Dolorimetry in SC males was correlated with COMPASS scores for autonomic dysfunction, followed by quality of life, migraine without aura, interoceptive sensing (True of You), and discomfort such as dyspnea (Supplementary Table S17). Anxious Arousal (MASQ) was present.

Elevated fatigue defined the CIF group. CIF had correlations of dolorimetry with Epworth Sleep Questionnaire items for sleepiness and inattentiveness, joint pain, interoceptive complaints including gastrointestinal issues, and impaired quality of life. Dolorimetry was associated with reduced body mass index.

In GWI males, dolorimetry was most highly correlated with tender point counts (*R*^2^ = 0.449), irritable bowel syndrome by Rome III criteria (*R*^2^ = 0.476), McGill Pain Score (*R*^2^ = 0.120), True of You questionnaire (*R*^2^ = 0.103), and interoception (*R*^2^ = 0.101).

All groups had correlations between dolorimetry and interoceptive items such as CMSI no pain, True of You, irritable bowel syndrome diagnoses, migraines and headaches, bladder, pupillary, and other complaints. Tenderness (reduced dolorimetry) was correlated with pain in GWI males, and poor quality of life and affective complaints were found in SC and CIF males. Low BMI in CIF males was correlated with dolorimetry.

Dolorimetry in SC females was associated with orthostatic complaints (COMPASS), widespread pain, poor quality of life, and mental fatigue (MDFI). Dolorimetry and BMI were positively correlated, suggesting a relationship between tenderness and “asthenia.”

CIF females had a correlation of dolorimetry with widespread pain, anhedonia, anxious arousal, sleep, and fatigue complaints. Migraines and gastrointestinal complaints were also correlated.

CFS females had correlations of dolorimetry with McGill pain scores (*R*^2^ = 0.310) and other measures of pain, interoceptive complaints, True of You scores and migraine.

GWI females had correlations with COMPASS GI scores (*R*^2^ = 0.800), disability (Physical Function *R*^2^ = 0.416), pain (McGill *R*^2^ = 0.258) and interoception.

## Discussion

Our hypothesis was that central sensitization and tenderness would correlate with fatigue, interoception, pain, and disability. The proxy for tenderness was dolorimetry pressure thresholds (kg), with Chalder for fatigue, McGill Total Score for pain, CMSI (no pain) for interoception, and the average of the SF36 Role Physical, Vitality, and Social Functioning domains for disability. Dolorimetry correlated well with the McGill Total Pain (explained variance *R*^2^ = 0.30–0.40) in women (Table 3) and men (Table 4) but had weaker correlations with interoception (CMSI no pain, *R*^2^ = 0.17–0.33) and poor correlations with Chalder Fatigue and disability domains (*R*^2^ = 0.05–0.16, not significant). The best match was between pain and interoception (*R*^2^ = 0.55–0.64), suggesting parallel increased messaging due to dampened antinociceptive and antiinteroceptive sensory inhibition. Fatigue and disability had the next best correlations (*R*^2^ = 0.41–0.47). Other correlations of fatigue, disability, pain, and interoception were higher in males (*R*^2^ = 0.34–0.46) than in females (*R*^2^ = 0.20–0.30).

The correlation of pain and interoception suggested increased sensory perceptions of warning signals that could be due to increased afferent traffic from the periphery (e.g. nociceptive pain) or a reduction in central antinociceptive (nociplastic pain) and antiinteroceptive mechanisms with a loss of their inhibitory activities. Tenderness (dolorimetry) was more highly correlated with pain than with interoception, suggesting that nociplastic mechanism(s) of central sensitization explained more of the variance of pain than interoception processes. This would be consistent with several independent pathways contributing to each mechanism. One explanation is the anatomical difference between dorsal horn and spinothalamic pain pathways vs. cranial nerve (vagal) interoceptive pathways.

Central sensitization was poorly correlated with fatigue and disability, suggesting that the midbrain and brainstem regulatory mechanisms had little impact or that fatigue and disability were derived from higher subcortical or cortical regions. These ideas suggest a dichotomy between midbrain and brainstem inhibitory antinociceptive and antiinteroceptive pathways, as opposed to subcortical and cortical systems that evaluate these sensations in the context of homeostasis, fatigue, effort valuation, affective, emotional, and limbic dysfunction. These hypotheses can now be tested and powered using the current outcomes for future investigations of causation in ME/CFS and GWI.

Dolorimetry findings were significantly different between groups by parametric analysis (Figure 10). The data were widely skewed, and so the non-parametric ROC did not provide strong support for any specific level of dolorimetry or tender point counts to diagnose ME/CFS or GWI in males or females. However, the outcomes do support the general concept of widespread tenderness in these diseases when CIF subjects are accounted for. This was most clear for males, where thresholds were stratified as SC>CIF>ME/CFS=GWI (Figure 11). CIF subjects contributed to the left-skewed distribution for dolorimetry in the SC males. SC females had two modes for tenderness even after removing the CIF subset (Figures 2, 10). This finding may be an artifact but should be investigated in larger groups.

A surprising finding was the correlation between systemic hyperalgesia and symptoms of irritable bowel syndrome in men (*R* = −0.538; Table 4). This was likely due to the large number of male GWI subjects and their gastrointestinal problems but suggested that nociplastic and interoplastic mechanisms may contribute to the perception of systemic and gastrointestinal distress as a complication of gut dysbiosis (König et al., 2021; Guo et al., 2023; Nagy-Szakal et al., 2017; Malhotra et al., 2023; Nono Djotsa et al., 2024).

Despite its limitations, dolorimetry was a reliable tool to quantify changes in systemic hyperalgesia in clinical trials of a low glutamate diet in GWI (Holton et al., 2020) and fibromyalgia (Holton et al., 2012). Alternative methods to measure mechanical and other modes of hyperalgesia may be developed for diagnostic purposes and as tools with measurable outcomes in clinical research (van Driel et al., 2024; Garfinkel et al., 2022).

Chronic idiopathic fatigue (CIF; Vincent et al., 2012) has been defined by exclusion as excessive tiredness that is not associated with sufficient ancillary symptoms to meet the CDC criteria for ME/CFS (Steele et al., 1998; Reeves et al., 2007; Son, 2019). ROC analysis provided thresholds to define CIF from the sedentary controls in quadrant analysis: Chalder Fatigue ≥17, CISR ≥5/6, MDFI General Fatigue ≥15 and sum ≥65, SF36 Vitality ≤ 33, CFSQ Sleep ≥2.5 (range 0–4), Cognition ≥1.5, and PEM ≥1.5 (Table 8). Dolorimetry and tender point counts were not effective (AUC < 0.8). McGill, other pain scores, and interoceptive complaints were not significantly different from SC. CIF individuals may have other complaints, but these were not consistent across all CIF subjects. Therefore, CIF can be defined by chronic persistent moderate or severe fatigue lasting longer than 6 months and associated with impaired vitality, sleep, cognition, and exertional exhaustion (Alexander et al., 2010; Matura et al., 2018).

Another novel finding was that 81% of CIF met CMI criteria. CMI requires two of the following: fatigue, cognition/mood symptoms, or musculoskeletal pain. The fatigue, cognition, sleep, PEM, and reduced vitality in CIF contributed to the overlap with CMI (Table 8). The CMI criteria were originally created based on the presence or absence of symptoms without regard to severity in Gulf War veterans [Fukuda et al., 1998; Centers for Disease Control and Prevention (CDC), 1995] but were found to have low specificity (Duong et al., 2022). The Kansas Criteria improve specificity for GWI by testing a larger number of symptoms (Shine et al., 2014). Requiring moderate or severe severities rather than just the presence of a symptom (i.e., mild complaints) improves specificity, as shown for the SEID, Kansas, and CFSQ for Fukuda criteria. Requiring impaired quality of life by specific SF36 domain scores is another valuable structure for a more specific diagnosis.

ME/CFS and CIF had equivalent scores for Chalder, CISR and MDFI fatigue measures. However, ME/CFS had higher scores than CIF for McGill Total and Sensory scores and the ancillary items of the CFSQ. Interoceptive and disability scores for CIF were intermediate between control and ME/CFS, classified by quadrant analysis. Although the differences were significant, ROC showed AUC < 0.78 with poor sensitivity and specificity that precluded setting thresholds to distinguish CIF from ME/CFS (Supplementary Table S14).

ME/CFS was distinguished from SC by ROC for Chalder Fatigue ≥16.5, MDFI General Fatigue ≥15.5 and sum ≥65, CMSI ≥22.5 and ≥11.5 for the no pain construct, COMPASS Sum ≥23.5, Pupillomotor ≥1.8, and McGill Total score ≥5.0, Sensory score ≥4.0, and Affective score ≥0.5 (Supplementary Table S15). ME/CFS subjects can differ in their symptom profiles, so these thresholds should be used as guidelines and verified by larger studies.

GWI was readily distinguished from SC by differences in many scores and ROC thresholds (Supplementary Table S20). Separating GWI from ME/CFS could be more of a challenge given the symptom overlap. However, GWI had more severe complaints on the McGill pain, CMSI interoceptive and GI domain, Irritant Rhinitis, and MPTSD questionnaires (Supplementary Table S14). The quality of pain was different in GWI, with higher complaints of punishing, sharp, shooting, sickening, stabbing, and throbbing. GWI males had higher scores for cramping, fearful, gnawing, heavy, hot burning, splitting, and tender. The higher scores for CMSI Ear&Sinus, Irritant Rhinitis, rhinorrhea, and Rhinitis Scores suggested greater sensitivity to volatile organic compounds and fine particulate matter in inhaled air that can stimulate irritant trigeminal and vagal afferent nerve endings in the nasal and tracheal airways (Baraniuk, 2008). Elevated rhinitis, sinusitis, and dyspnea scores in GWI were consistent with the designation of rhinosinusitis and asthma as presumptive diagnoses eligible for disability by the Department of Veterans Affairs.^4^ The GI scores were consistent with higher rates of irritable bowel syndrome in GWI than in ME/CFS.

Although no individual psychological domains were significantly different between GWI and ME/CFS, PCA component 1 for GWI females and males contained anxiety, catastrophizing, and anhedonia. Psychological symptoms did not correlate with tenderness, suggesting that central sensitization mechanisms had little direct impact on emotional, psychological, and limbic processes. As a result, we postulate that antinociceptive mechanisms act in the midbrain and medulla below the supratentorial level of cerebral and subcortical centers.

The relevance of these symptom complexes can be inferred by following the afferent innervation from somatosensory and visceral origins. Sensations from the airway, carotid sinus, cardiac, gastrointestinal, and urinary organs are conveyed by polymodal Type C and A delta afferent fibers via cranial nerves III (oculomotor), V (trigeminal ganglia), VII (facial), IX (inferior glossopharyngeal ganglion), and X (vagal nodose ganglion; Prescott and Liberles, 2022), sympathetic afferents, and spinal general afferent nerves (Christianson and Davis, 2010). They convey chemosensory and stretch information from vessels and hollow viscera. The ascending internal or “interoceptive” visceral afferents (Sherrington, 1911; Craig, 2002; Garfinkel et al., 2017) synapse in the brainstem glossopharyngeal, vagal solitary tract, paratrigeminal nucleus (Driessen, 2019), trigeminal cervical spinal columns, and other nuclei. Vagal afferents primarily carry mechanoreceptor and chemosensory sensations related to the contraction and dilation of hollow viscera and local mucosal and intralumenal environments. Inputs are projected to the insula and cingulate cortex for conscious perception according to the Embodied Predictive Interoception Coding model (Khalsa et al., 2018). They provoke subliminal reactionary brainstem autonomic and other motor reflex responses (Berntson and Khalsa, 2021).

Spinal interoceptive afferents carry signals related to temperature, pain, and tissue injury and synapse in lamina I of the dorsal horn. Secondary interneurons rise in autonomic spinothalamic columns to the ventromedial medulla, parabrachial nucleus, periaqueductal gray, catecholamine cell groups, and ventromedial posterior thalamic relay nucleus, which in turn projects to the dorsal posterior insular cortex (interoceptive cortex; Craig, 2007). Multimodal sensory projections are integrated in the middle insula and reprojected to subcortical hypothalamus and amygdala centers of homeostatic control (Craig, 2007). Stronger salient inputs trigger the anterior insula and limbic cortical regions of the anterior cingulate and orbitofrontal cortex that are part of the salience network (Menon, 2011) and then the dorsolateral prefrontal cortex and executive control network for contextual planning. Under normal circumstances, there is no need for conscious awareness of interoceptive afferent sensations or the homeostatic functions. It is likely that the innervation is tightly regulated by as yet poorly defined antiinteroceptive systems so that only moderate or severe stimuli of visceral injury or obstruction can arouse awareness in higher brain centers. There is a spectrum of interoceptive self-perception, as some people readily perceive visceral sensations such as heartbeat. It will be of interest to find if those control subjects with intermediate dolorimetry thresholds have increased self-perception. Defects of anti-interoception may allow the persistence of interoceptive sensory perception and contribute to functional somatic sensory syndromes that become attributed to dysfunction of peripheral organs.

There appear to be several parallel antinociceptive mechanisms; it is not clear if antinociceptive systems also regulate interoceptive pathways or if there are separate, specific antiinteroceptive systems devoted to the regulation of interception. Incoming interoceptive sensory signals are monitored by the ascending arousal network nuclei in the medulla and midbrain periaqueductal gray, which are designed to detect and respond to existential threats and trigger threat responses (Bracha, 2006). Threatening interoceptive, nociceptive, and exteroceptive inputs alert the periaqueductal gray and reticular nuclei, which in turn arouse ascending neurons from the locus coeruleus, dorsal raphe, ventral tegmental area, and parabrachial complex. These neurons release their respective noradrenergic, serotonergic, dopaminergic, and cholinergic amine neurotransmitters broadly throughout the cerebrum to heighten attention to the perceived threat. They also have caudal projections that may contribute to antinociceptive and antiinteroceptive pathways. The sensory consequences of a broken sensory filter are unfettered, excessive subcortical, and insula activation with enhanced perception of visceral sensations by higher centers of conscious awareness (Baraniuk et al., 2022). The salience network, hypothalamus, subcortical, and other brain regions are activated to plan and initiate protective actions such as sheltering, guarding, withdrawal from pain-provoking activities, and other defensive and self-preserving “sickness behaviors” (Bracha, 2004). Chronic persistent perception of subthreshold interoceptive sensations and activation of the reactive responses may contribute to anxiety, depression, sleep disorders, and posttraumatic stress disorder (PTSD; Berntson and Khalsa, 2021; Cao et al., 2024; Bracha et al., 2005).

**Figure 12:**
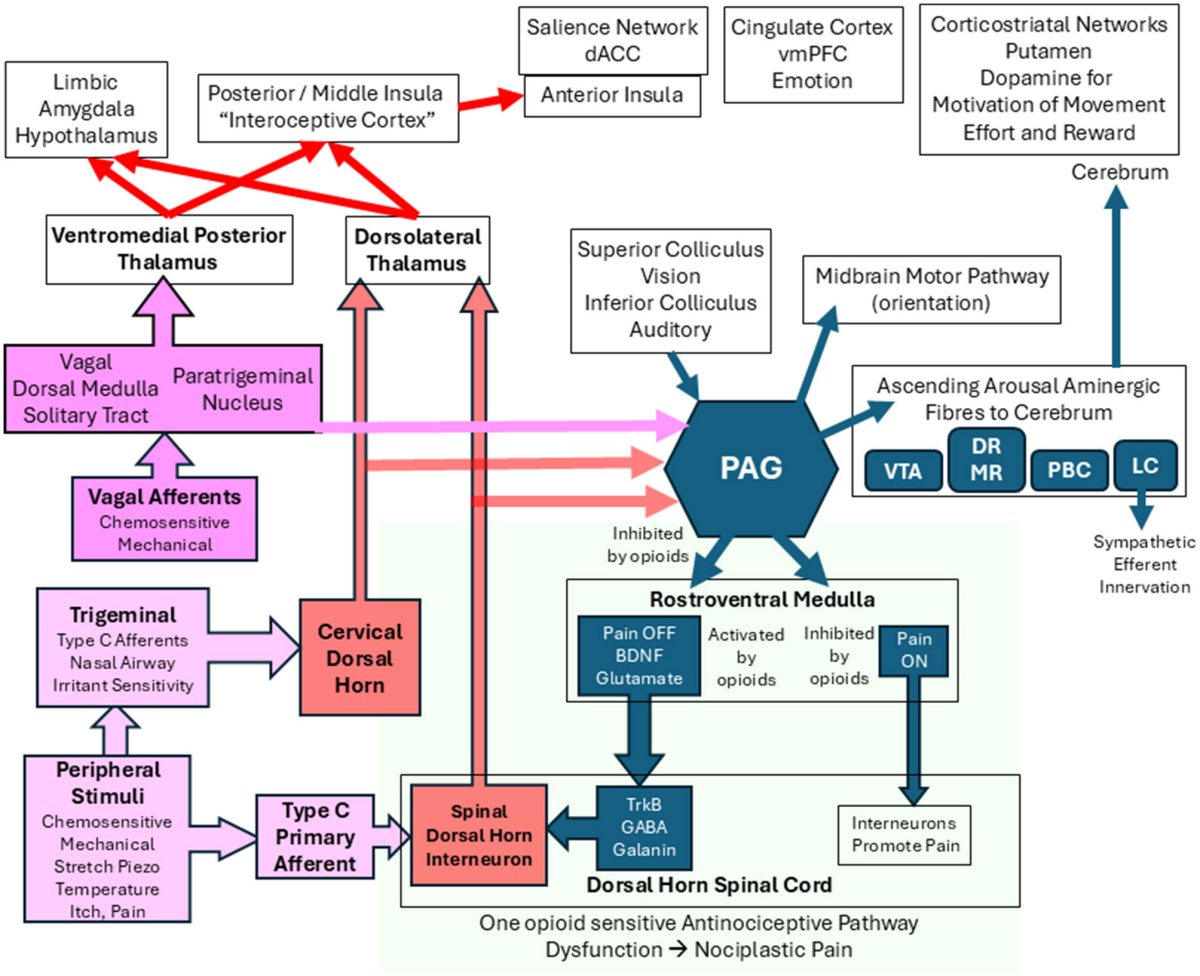
Putative neural pathways for antinociception and antiinteroception. Peripheral chemosensitive and other stimuli (pink box) activate Type C non-myelinated afferent fibers that synapse in the trigeminal and spinal dorsal horn and central vagal nuclei (red boxes). Ascending nociceptive afferents send collaterals to the midbrain periaqueductal gray matter (PAG, blue). Antinociceptive PAG neurons descend to the rostroventral medulla where “Pain Off” neurons (as opposed to “Pain On” neurons) are activated (blue). The “Pain Off” axons descend to the dorsal horn where they release BDNF and glutamate to activate TrkB GABA galanin inhibitory interneurons that block nociceptive signaling. This antinociceptive pathway (striped light blue background) is activated by opioids. It is likely that parallel serotonin and other molecular pathways are also present. It is not clear if these pathways serve antiinteroceptive processes or if there are additional separate parallel pathways. Dysfunction of the descending pathways leads to interplastic and nociplastic mechanisms. The ascending interoceptive and nociceptive pathways from the dorsal horn and vagal nuclei synapse in thalamic nuclei, with the signals conveyed to the posterior insula (“interplastic cortex”) and limbic hypothalamic and amygdala nuclei. The signals enter conscious perception if they reach the anterior insula and the salience network. Activation of the cingulate cortex and corticostriatal networks links the interoceptive and painful sensations to emotional motivation for effort and dopamine-related reward for action. Visual and auditory inputs through the superior and inferior colliculi to the PAG also cause alarm for threat perception and responses such as reorientation of the eyes and head via the midbrain motor pathways. Strong activation of the PAG stimulates ascending arousal centers in the ventral tegmental area (VTA), dorsal and medial raphe (DR, MR), parabrachial complex (PBC), and locus coeruleus (LC). Their respective dopaminergic, serotonergic, cholinergic, and noradrenergic innervation leads to general cerebral arousal. Descending adrenergic innervation from the locus coeruleus generates efferent sympathetic innervation.

The descending antinociceptive network from the midbrain and brainstem involves opioid, dopamine, serotonin, calcitonin gene-related peptide, and other antinociceptive mechanisms (Cao et al., 2024; Condon et al., 2024). One pathway originates in the periaqueductal gray (PAG) matter in opioid-responsive pain-monitoring neurons (Tobaldini et al., 2018) that descend to innervate the rostral ventromedial medulla (RVM; Figure 12). The RVM contains populations of pain “ON” neurons that are inhibited by opioids and pain “OFF” neurons that are activated by opioids (De Preter and Heinricher, 2024). One subset of glutamatergic “OFF” neurons expresses brain-derived neurotrophic factor (BDNF) and descends to the spinal dorsal horn, where they activate interneurons expressing the BDNF receptor, tropomyosin receptor kinase B (TRKB; Fatt et al., 2024). The TRKB interneurons release gamma-aminobutyric acid (GABA) and galanin that inhibit spinal processing of injurious mechanical afferent sensory neurons to prevent pain transmission. Dysfunction of this central nociplastic pathway would permit increased dorsal horn transmission of pain sensations, widespread hyperalgesia, and central sensitization. It remains to be determined if this opioid-sensitive pathway inhibits interoception or if there are additional parallel mechanisms. Understanding mechanisms of central sensitization may lead to new concepts for therapies (Arendt-Nielsen, 2015). BDNF derived from microglia and neurons in the hippocampus, parabrachial nucleus, and locus coeruleus also contribute to central sensitization (Merighi, 2024).

We anticipate that future animal studies of midbrain and brainstem sensory and autonomic regulation will reveal additional feedback mechanisms that may be generalized or more specific for pain vs. interoception.

The Central Fatigue Hypothesis (Meeusen et al., 2006) implicates serotonin and possibly dopamine pathways as braking systems that act to prevent exertion that exceeds the limits necessary to maintain homeostasis during exercise (Meeusen et al., 2006). These brakes will stop effort and exertion when neurocognitive reserves are threatened and will generate a perception of fatigue, negative reward (disincentive; Bonnelle et al., 2015), loss of motivation (Vassena et al., 2017), or apathy (Starkstein and Brockman, 2018) to complete the task at hand (Noakes, 2012). From a physiological perspective, molecular mechanisms of mitochondrial energy deficits, inflammation with elevated IL6 and TNF, oxidative stress, hypothalamic-pituitary adrenal axis dysfunction, autonomic dysfunction, obesity and adipokine inflammation, and other processes may contribute to fatigue in a wide variety of chronic autoimmune, neoplastic, infectious, cardiac, pulmonary, joint, brain, kidney, and other disease settings (Matura et al., 2018). These diseases are important exclusions for ME/CFS and GWI and can account for up to 78% of potential patients in case series evaluated for chronic fatigue (Vincent et al., 2012). Chronic fatigue increases with advanced age and is associated with frailty and mitochondrial dysfunction (Alexander et al., 2010), but it is not known if this mechanism also applies to the younger CIF subjects defined here.

The nociplastic hypothesis provides a single mechanism to explain widespread pain. The alternative is to propose widespread peripheral inflammation as a cause of whole-body nociceptive pain. We propose that a comparable antiinteroceptive system controls conscious perception of “autonomic” and visceral signaling. Compromise of this inhibitory system would lead to the wide range of internal sensations that have previously been considered separate independent functional somatic syndromes emanating from specific body systems. Instead, interoplastic pathology and reduced antiinteroceptive function would allow simultaneous conscious perception of a wide gamut of internal discomforts. This unifying hypothesis helps to explain the comorbidities of migraine, tinnitus, irritant non-allergic rhinitis, chronic cough, dyspepsia, irritable bowel syndrome, irritable bladder syndrome, and other conditions.

This hypothesis does not negate immune, autoimmune, microbiome, metabolomic, mitochondrial, or neurotoxic pathologies that may initiate brain or peripheral organ injury and exacerbate the sensory perceptions. These pathological mechanisms may also affect the midbrain and brainstem pathways and initiate the interoplastic and nociplastic dysfunction. The same reasoning can be applied to the new epidemic of Long COVID (Fernández-de-Las-Peñas, 2022). COVID infection in humans and murine models leads to elevated CCL11 (eotaxin) that is associated with hippocampal and other microglial activation, loss of oligodendrocytes, brain demyelination, structural changes (Douaud et al., 2022), and cognitive dysfunction (Fernández-Castañeda et al., 2022). Cognitive dysfunction in ME/CFS, GWI, and “COVID fog” has parallels with fibromyalgia cognitive dysfunction (“fibro fog”), cancer therapy-related cognitive impairment (“chemo fog”) that follows brain radiation, methotrexate and other chemotherapeutic agents, and low-dose lipopolysaccharide models of systemic inflammation (Geraghty et al., 2019; Gibson and Monje, 2021). Elevated eotaxin, aberrant microglial pruning of synapses, reactive astrocytes, oligodendrocyte cell death, and BDNF expression may contribute to a common final pathway for cognitive dysfunction (Polli et al., 2020). BDNF may also be dysregulated in exercise and post-exertional malaise (Venezia et al., 2017). Understanding the symptomatology of these diseases and correlation with molecular mechanisms of pathology will cross-fertilize investigations for all of these nociplastic, interoplastic, fatiguing illnesses (NIFTI) and may provide insights into new therapies (Ayoub et al., 2020; Acharya et al., 2016).

About one quarter to one third of military personnel exposed to the conditions of the Persian Gulf War have developed unremitting GWI. About 1% of the general population have had sudden onset viral-like syndromes or gradual onset of unremitting ME/CFS symptoms. In both cases, subjects consider it a mystery as to why they got sick and do not get better. Their reflections on the constancy and permanence of the symptoms have been interpreted as rumination. They have often been told that “it is all in your head” (Timbol and Baraniuk, 2019). The lack of empathy and therapies engenders frustration, anxiety, irritability, and a sense of helplessness. The fatigue, severe pain, unexplained interoceptive complaints, and exertional exhaustion preclude involvement in activities that were previously enjoyable (anhedonia). These psychological constructs describe the reality of ME/CFS, GWI, and Long COVID and are often mistaken for depression.

An alternative approach to this conspiracy of nomenclature and symptom salad (Figure 1) is to re-consider the psychic symptoms in GWI and ME/CFS as “motivational anhedonia” that is characterized by deficits in reward motivation, strongly impaired quality of life (Whitton et al., 2023), fatigue and lack of energy (Treadway and Zald, 2011; Felger and Treadway, 2017; Billones et al., 2020; Daumas et al., 2022). Elements of fatigue, anhedonia and apathy are correlated but are not synonymous. The central pathology of motivational anhedonia involves altered processes for valuation of cognitive and physical efforts when contrasted with potential positive rewards vs. inevitable punishing symptom exacerbation and relapse (PEM; Haber and Knutson, 2010; Treadway and Salamone, 2022; Cooper et al., 2018). Motivational anhedonia is linked to dysfunction of dopaminergic corticostriatal reward networks in the putamen, dorsal anterior cingulate cortex (dACC) and surrounding paracingulate and presupplementary motor areas (Figure 12; Pizzagalli, 2022; Tye et al., 2013; Husain and Roiser, 2018). These regions are critical hubs for effort-based decision-making (Arulpragasam et al., 2018; Bonnelle et al., 2016; Lopez-Gamundi et al., 2021; Walton et al., 2003; Rudebeck et al., 2006), evaluation of the cost of efforts (Westbrook et al., 2019) and consideration of the difficulty of tasks (Shenhav et al., 2014). We can anticipate that appropriate provocation studies with magnetic resonance imaging in ME/CFS and GWI will demonstrate dorsomedial prefrontal dysfunction or altered functional connectivity between the ventral striatum and ventromedial prefrontal cortex (Bekhbat et al., 2022). The dACC is a major hub of the salience network (Menon, 2011) that may be enlarged in persons at risk for major affective disorders such as depression (Lynch et al., 2024). Enlargement of salience nodes and overactivity of attention and vigilance networks may contribute to the continuous threat assessment, motivational anhedonia and increased scores on psychological and other questionnaires in GWI and ME/CFS. This pathomechanism is relevant to major depression where about one third of cases have an inflammatory phenotype with CRP levels >3 mg/L and respond to infliximab, a monoclonal antibody that antagonizes tumor necrosis factor alpha (TNFa; Treadway et al., 2024). About a third of our subjects had elevated CRP and possible motivational anhedonia suggesting that infliximab may be a viable experimental therapy for a “neuroinflammatory” phenotype of ME/CFS and GWI. Before considering motivational anhedonia as a component of ME/CFS and GWI it is necessary to demonstrate fMRI evidence of dysfunctional prefrontal striatal connectivity during tasks of reward discounting.

The rate of PTSD in this cohort is higher than in other studies (Weiner et al., 2011; Richardson et al., 2010). Emerging data indicate that the subgroup of GWI with PTSD may have more severe disease with aggravating pathological features (Sultana et al., 2024; Jeffrey et al., 2021). This places a large onus on the Department of Veterans Affairs medical system to incorporate questionnaires such as the MPTSD, PCLC, PCLM, and Kansas questionnaire into routine medical practice to improve diagnosis and expand healthcare for veterans at risk. A lack of insightful care will promote a sense of frustration in veterans and withdrawal from the medical system, potentially leading to self-medication and the morbid cycle of suicidality.

This analysis has limitations. The sample sizes were small and unbalanced after accounting for gender and CIF subsets. Verification of the current findings will require larger sample sizes, but the raw data, means and standard deviations, Hedges' g, and Spearman correlation values provide effect sizes that can be used to power future studies appropriately. However, larger studies may find smaller effect sizes than those inferred from the current results (Owens et al., 2021).

Bonferroni corrections for multiple comparisons were employed to correct for the large number of questionnaire domains that were compared. Spearman correlations with |R| > 0.5 were required for consideration of association, but do not demonstrate causality. The ROC thresholds provide guidance for diagnosis but only if the analysis is sufficiently significant. The speculative mechanistic inferences are hypotheses for future testing and to stimulate fresh insights.

Confounding factors were excluded by univariate general linear modeling with age, race, Hispanic status, body mass index (BMI), PTSD, Type II diabetes mellitus, and FM by 1990 criteria. Other comorbidities or underlying conditions may still be discovered that impact tenderness and central sensitization in ME/CFS and GWI. Additional factors such as atypical depression, drug abuse, cellular senescence, occult cardiovascular issues, infectious diseases, cancer, or other chronic diseases may contribute to CIF and will have to be evaluated in future studies.

The gender differences for dolorimetry (Figures 7, 9, Table 1) and other objective outcomes in ME/CFS (Cheema et al., 2020; Gamer et al., 2023; Jeffrey et al., 2019) demonstrate that sexual dimorphisms must be evaluated for all study outcomes before generalizing across genders for all subjects. We evaluated this limitation by assessing genders separately for differences in means between ME/CFS, GWI, and control, PCA, and Spearman correlations (Supplementary material). Women had greater nociplastic sensitivity, but questionnaire outcomes were more similar between sexes. Although interoceptive scores were similar between genders, correlations with objective mechanisms may differ, as suggested by higher correlations for women between interoceptive Self-Awareness Questionnaire scores and brain functional connectivity in salience and frontal-parietal executive control networks than for men (Alfano et al., 2023). Menopause represents another important variable to assess because of differences in nociception and dorsal horn gene regulation in animal models of males and oophorectomized females compared to fertile females (Dedek et al., 2022).

Assigning subjects into diagnostic groups tends to restrict the range of symptom scores they experience. Controls have few symptoms, indicating floor effects for correlation. Designation of CIF requires that fatigue be moderate or severe, so the full range from none to severe was not available for correlations. Dolorimetry pressures were similarly skewed to lower ranges in ME/CFS and GWI. The ceiling and floor effects greatly reduced the mathematical possibility of finding significant correlations. Pain, interoception, migraine, and disability were still significantly correlated with tenderness across the various groups and in both sexes. The explained variances were low, indicating that other variables or confounders contributed to the relationships but were not revealed by age, gender, body mass index, and questionnaire topics. Dolorimetry and interoception were correlated, but with a low, yet still significant, explained variance, indicating that nociplastic mechanisms contributing to pain had some overlap with interoceptive mechanisms regulating perceptions derived from vagal and other internal visceral sensing systems.

ME/CFS and GWI have different disease etiologies but share similar patterns of differences from control nociceptive, interoceptive, and other questionnaire outcomes. The average dolorimetry pressures and ROC analysis did not separate ME/CFS from GWI. This suggests that diverse initiating inflammatory or neurotoxic exposure events may be followed by shared “final common pathways” that produce central sensitization and similar symptomatic and tenderness disease manifestations. Future studies of long COVID will provide an additional “illness control,” where we predict central sensitization will lead to a comparable suite of dolorimetry, symptoms, and questionnaire scores.

Tenderness was demonstrated in ME/CFS, which makes it difficult to differentiate fibromyalgia using the 2010 and 2019 criteria. Future studies should report dolorimetry or other evidence of central sensitization in order to exclude fibromyalgia and study “pure” ME/CFS. However, our hypothesis of shared antiinteroceptive and antinociceptive mechanisms weakens the possibility of dichotomizing ME/CFS vs. ME/CFS/fibromyalgia. Univariate models that include dimensions of fatigue, pain, tenderness, and other outcomes are likely to be better statistical approaches. Our GWI cohort was more tender than others [e.g., an average of 6 tender points, 28% with ≥11/18 tender points (Steele et al., 2021)].

The separation of sensations into interoceptive and nociceptive is a reasonable starting point for sensory analysis but is likely to be simplistic. Additional sensory modalities, such as pruritogenic, thermal, and piezomechanical, exist that have not been as well characterized for increased central perception in ME/CFS, GWI, or other diseases with central sensitization (Dunphy et al., 2003). Sensory modalities are mediated by over two dozen subclasses of small-diameter unmyelinated and large myelinated dorsal root ganglion, trigeminal, and vagal nodose and jugular ganglion neurons (Kupari et al., 2019; Yu et al., 2024; Nguyen et al., 2017). These fiber types exhibit diverse properties, including differential presynaptic inhibition by opioid mu, delta, and kappa receptors, transcription factors, and mRNA expression. Each may have specific descending inhibitory neural pathways or other means of sensory regulation. Future investigations may identify specific inhibitors for these modalities and correct nociplastic, interoplastic, and other dysfunctional neural signaling mechanisms.

Inclusion of Common Data Elements, such as quality of life instruments, in published reports will improve data sharing for study design and power calculations (Cohen et al., 2022; Slavin et al., 2023; Grinnon et al., 2012). The questionnaires were chosen to assess fatigue, quality of life, pain, interoception, and psychological constructs, but the strongest correlations were within questionnaire domains rather than with fatigue or other indices. It is not clear if newer questionnaires that have been introduced since the start of data collection will improve the potential for correlated outcomes and specificity in ME/CFS and GWI. There is significant overlap of items with similar wording but different interpretations between different questionnaires that may lead to confusion about the validity of underlying latent factors. Despite this, there was surprisingly poor correlation between fatigue scales (Chalder, CISR, MDFI, Epworth). Questionnaires with more items and levels of severity appeared to be superior to those with dichotomous present/absent or yes/no answers. As a result, the Kansas criteria provide more information than the CMI classification. ME/CFS and GWI have many complaints with high severity, leading to wide ranges and large variance ceiling effects, but are in contrast to SC, who have minimal symptoms, narrow ranges for SD, and floor effects. Different questionnaires will be required to bypass the ceiling effects when studying the most severe ME/CFS cases.

The volunteers made active decisions to participate and were thus a motivated self-selected group who may have been seeking assistance and insight into their personal experiences, as opposed to subjects who have withdrawn from contact and did not participate. Population-based studies will be necessary to confirm our findings. One way to access the population is for medical providers and the VA medical system to incorporate the Kansas, DePaul, and MPTSD instruments into electronic medical records dashboards in order to cue doctors to investigate these diagnoses and educate doctors and patients who are familiar with GWI or ME/CFS about their disease. The exercise will generate clinical databases for further study.

The clinical criteria for both diseases continue to be challenging for clinicians. Precise case designation criteria must be applied in research manuscripts. Requiring moderate or severe complaints will improve diagnostic specificity and reduce the false positive impact of CIF and other diseases that feature fatigue. This will also apply to the overlap of functional somatic syndromes such as IBS, where central sensitization is inferred. Tenderness should be stringently measured in these other disorders in order to understand the holistic spectrum of bodily complaints, personal impairments, and coping strategies. The correlations are a starting point for planning new studies of shared deficits in fMRI, metabolomics, gut microbiome, and other mechanistic outcomes to bridge the gap between subjective and objective testing in order to define mechanisms of disease, clinical biomarkers, and diagnostic tests.

We demonstrate that people with ME/CFS and GWI have tenderness. Even though we did not define strong criteria for specific numbers of tender points or dolorimetry thresholds, it is clear that the general finding of systemic tenderness is consistent with these diagnoses and their symptom profiles. Animal models are revealing new antinociceptive pathways, but there is much less research into interoception and antiinteroceptive mechanisms. Future studies can be powered using the outcomes reported here (Supplementary Table S20). Our outcomes can also be used to define CIF. On a practical note, we advocate testing for tenderness by physical examination as an adjunct to the published symptomatic criteria. Future provocation methods may be developed that can be readily used in the clinic to investigate the finding of central sensitization and to demonstrate that lower levels of provocation can lead to exaggerated levels of complaints (i.e., tenderness).

## Data Availability

The datasets presented in this study can be found in online repositories. The names of the repository/repositories and accession number(s) can be found in PDF format in the article/Supplementary material.

## Footnotes

^1^ https://www.who.int/standards/classifications/classification-of-diseases

^2^ http://vassarstats.net

^3^ https://www.ibm.com/products/spss-statistics

^4^ https://www.federalregister.gov/documents/2021/08/05/2021-16693/presumptive-service-connection-for-respiratory-conditions-due-to-exposure-to-particulate-matter
