# Supplementary material for: Fatigue, interoplastic and nociplastic distress in myalgic encephalomyelitis/chronic fatigue syndrome, Gulf War Illness, and chronic idiopathic fatigue
Source: Front Neurosci. 2025 Aug 25;19:1530652. doi: 10.3389/fnins.2025.1530652 (PMC12415031; doi:10.3389/fnins.2025.1530652)
Supplement: Supplementary file 1 [file Data_Sheet_1.pdf]

SUPPLEMENTARY ONLINE MATERIAL

Fatigue, Interoplastic and Nociplastic Distress in Myalgic Encephalomyelitis / Chronic Fatigue Syndrome, Gulf War Illness and Chronic Idiopathic Fatigue

Emily Chen, Tamera Rudder, Charles Nwankwere, James N Baraniuk

SOM Table 17.
Data were compared between sedentary controls (SC), ME/CFS diagnosed by CCC, and GWI subjects diagnosed by CMI plus Kansas criteria. Data are reported for males (SOM Table S1) and females (SOM Table S2) and organized by significant differences between groups.

GWI > ME/CFS > SC: Aching quality on the McGill Pain questionnaire (98) (Hedges' g = 2.4) and muscle pain reported from the ME/CFSQ (42) (Hedges' g = 3.2) were ranked as most severe for male GWI > ME/CFS > control.

GWI = ME/CFS > SC: GWI and ME/CFS males had equivalent pressure thresholds (kg) that were significantly lower than controls. Differences in tenderness were evaluated further by receiver operating characteristics (see below).

The overlap of diagnostic criteria was evaluated. GWI males who met Kansas plus CMI criteria also met the Carruthers Canadian Consensus Criteria (CCC, 93.0%) (26,43) and 1994 Fukuda CFS criteria (90.9%) (25).The overlap was supported by high scores for the core CFSQ symptoms of postexertional malaise, fatigue, cognition and sleep (42) and Chalder Fatigue score (96). The qualitative sensation of tiring pain on the McGill questionnaire (98) was significantly elevated in GWI and ME/CFS males. Effect sizes were particularly large for GWI (Hedges' g 2.1 to 2.4).

The Pain Beliefs and Perceptions Inventory (PBPI) questionnaire (118) refers to the understanding of pain. The mystery sub-scale was significant for GWI and ME/CFS males. Disability was severely impacted based on SF36 domain scores that were significantly lower than control, especially vitality, role physical and social function (2,92). Effect sizes were particularly large for GWI (Hedges' g 2.0 to 2.5).

GWI > ME/CFS = SC: GWI males had more thumb pressure tender points and widespread pain that is consistent with the diagnosis of Fibromyalgia by 2010 criteria (5). The importance of pain in GWI is evident by significantly higher Total, Affective, and Sensory scores from the McGill Pain questionnaire. The quality of pain was described as punishing, sharp, shooting, sickening, stabbing, and throbbing (98). GWI males also reported greater joint pain by the CFSQ (42).

Interoceptive complaints in GWI were more severe using the Chronic Multisymptom Inventory (CMSI) (99) and COMPASS 31 of symptoms associated with autonomic dysfunction (106). In particular, GWI males had high scores for Irritable Bowel Syndrome and about 70% prevalence by Rome I criteria (99). GWI males were unique by having more impairment on the SF36 Role Emotional domain than ME/.CFS and control men (92). GWI had a sense of Helplessness from the Pain Catastrophizing Scale (PCS) (120)

GWI > SC: Rates of ME/CFS diagnosis by the Systemic Exertion Intolerance Disease criteria (79.5%) (45) and FM diagnosis by the 1990 (43.2%) (5) and 2011 (75%) criteria (60) were elevated in GWI.

McGill questionnaire scores categorized pain felt by GWI males as being more cramping, heavy, hot burning and tender [2] (98).

Interoceptive complaints were higher based on domains of the Chronic Multisymptom Illness (CMSI) questionnaire and its Neuro, Headache, Ear and Sinus, Rheum, Cardiac, Dyspnea and gastrointestinal / IBS domains (99). Pupillomotor complaints of blurred and double vision were elevated in GWI men compared to healthy controls suggesting autonomic dysregulation (106).

Rates of migraine headaches were higher in GWI (59%) [2] compared to controls (11.4%) using International Headache Society criteria (103) confirming previous investigations (203).

Irritant sensitivities were indicated by elevated scores for domains of the Chemical Exposures questionnaire (105). GWI and healthy veterans in the SC group were exposed to oil well fires, diesel, vaccinations, and combat (data not shown)

Psychological measures indicated greater General Distress, and Anxious Arousal using the Mood and Anxiety Symptoms Questionnaire (MASQ) (111), irritability (TIQ) (113), depression (115), and PTSD (123,204). The M-PTSD threshold score of 94 determined that 57.3% (55/96) of GWI males had PTSD (205). PTSD Check List for Civilians (PCL-C) (206) confirmed this high rate and graded the PTSD symptoms as severe (range of 45 to 85) in 64.3% of GWI (27/42) compared to 10% of ME/CFS (1/10) and none of the control (0/13) males (Chi2 p<0.0001).

The Pain Beliefs and Perception Inventory described pain with Constance indicating greater self-reported pain on a daily basis, and Permanence by believing that pain will be an enduring part of subjects' lives (118). GWI males tended to magnify and ruminate on pain (120). GWI scored significantly lower on the Internal Score (locus of control) but were higher for Powerful Doctors on the Beliefs in Pain Control Questionnaire (119). They had the least robust mental health amongst the groups by SF36 (92). The absence of medical therapies and treatment model that considers GWI to be a psychological disorder were indicated by the high frequency of GWI males reporting cognitive behavioural therapy (CBT) (77.8%).

Over half of GWI had GAD7 ≥ 10 that is consistent with elevated anxiety and irritability (TIQ). Risk of depression was suggested by CESD scores ≥ 16 / 60 in 83% of GWI. However, CESD scores in GWI were dominated by the Somatic factor (GWI: 12.4 ± 4.1, range 0 to 21, g = 2.0, vs SC: 4.4 ± 4.1) with its complaints of fatigue, sleep disturbance and other symptoms that overlap with ME/CFS and GWI criteria.

SOM Table S1. Male SC v ME/CFS v GWI. Differences between SC, ME/CFS and GWI males were assessed by ANOVA with Tukey Honest Significant Differences then Bonferroni correction for 300 questionnaire domains and items. Hedges' g was calculated for significant differences (p<0.05) between groups. Proportions (%) were compared by Fisher Exact Test.

Male variable	SC Male	ME/CFS Male	GWI Male	g ME/CFS > SC	g GWI > SC
**GWI = ME/CFS > SC**					
CCC Dx (%)	0% (16)	90.9% (11)	93.0% (44)		
CFS Fukuda 1994 (%)	0% (16)	100% (16)	90.9% (44)		
Dolorimetry (kg)	6.9 ± 1.9 (37)	4.2 ± 2.2 (20)	4.0 ± 2.2 (115)	1.3	1.3
CFSQ Exertion (0-4)	0.74 ± 1.19 (34)	3.17 ± 1.10 (18)	3.37 ± 0.93 (108)	2.1	2.6
CFSQ Fatigue (0-4)	1.38 ± 1.13 (34)	3.39 ± 1.04 (18)	3.56 ± 0.59 (108)	1.8	2.9
CFSQ Memory (0-4)	1.12 ± 1.23 (34)	2.67 ± 1.03 (18)	3.07 ± 0.82 (108)	1.3	2.1
CFSQ Sleep (0-4)	1.47 ± 1.26 (34)	3.22 ± 1.00 (18)	3.57 ± 0.69 (108)	1.5	2.4
Chalder fatigue (0-33)	13.12 ± 5.64 (33)	22.61 ± 5.34 (18)	24.64 ± 5.23 (107)	1.7	2.2
McGill tiring (0-3)	0.30 ± 0.68 (33)	1.83 ± 1.29 (18)	2.48 ± 0.84 (107)	1.6	2.7
SF36 Bodily Pain (0-100)	82.14 ± 25.17 (35)	47.36 1 ± 32.85 (18)	29.69 ± 19.30 (106)	1.2	2.5
SF36 General Health (0-100)	66.14 ± 22.75 (35)	35.28 ± 22.52 (18)	22.55 ± 15.36 (106)	1.3	2.5
SF36 Physical Function (0-100)	85 ± 23.33 (35)	55 ± 26.07 (18)	46.98 ± 24.95 (106)	1.2	1.5
SF36 Role Physical (0-100)	77.86 ± 35.75 (35)	11.11 ± 24.59 (18)	11.32 ± 24.18 (106)	2	2.4
SF36 SF10 (0-100)	72.61 ± 26.10 (35)	18.47 ± 20.06 (18)	19.25 ± 16.66 (106)	2.2	2.7
SF36 Social Function (0-100)	79.64 ± 26.62 (35)	31.25 ± 27.54 (18)	29.48 ± 23.67 (106)	1.8	2
SF36 Vitality (0-100)	55.29 ± 28.02 (35)	13.33 ± 16.63 (18)	15 ± 14.26 (106)	1.7	2.2
PBPI Mystery	-0.81 ± 1.38 (22)	1.36 ± 0.66 (7)	0.77 ± 0.92 (61)	1.7	1.5

**GWI > ME/CFS > SC**					
CFSQ Muscle pain (0 to 4)	0.79 ± 1.01 (34)	2.11 ± 1.68 (18)	3.31 ± 0.70 (108)	1	3.2
McGill aching (0-3)	0.61 ± 0.75 (33)	1.67 ± 1.28 (18)	2.42 ± 0.75 (107)	1.1	2.4
**GWI > ME/CFS = SC**					
FM 2010 (%)	6.6% (15)	27% (11)	88.6% (44)		
Widespread Pain (%)	3.0% (33)	23% (13)	73.0% (37)		
Tender points ≥11/18 (%)	5.7% (35)	15.0% (20)	55.0% (115)		
Tender point count (0-18)	2.0 ± 3.4 (34)	4.1 ± 5.4 (19)	10.2 ± 6.5 (113)	0.5	1.4
CFSQ Joint pain (0-4)	1.0 ± 1.1 (34)	1.5 ± 1.4 (18)	3.2 ± 1.0 (108)	0.4	2.3
Chemical Symptoms	11.9 ± 17.1 (34)	32.1 ± 22.0 (18)	53.7 ± 20.7 (105)	1.1	2.1
CMSI sum (0-172)	19.1 ± 22.8 (34)	45.1 ± 28.0 (18)	78.2 ± 30.0 (103)	1	2.1
CMSI GI domain (Rome 1)	3.0 ± 5.9 (34)	6.6 ± 8.2 (18)	16.7 ± 8.4 (103)	0.5	1.7
COMPASS GI domain (0-25)	3.1 ± 2.8 (12)	6.1 ± 3.9 (10)	12.4 ± 3.4 (43)	0.9	2.8
COMPASS sum (0-100)	14.7 ± 8.5 (12)	23.3 ± 13.5 (10)	43.3 ± 13.8 (43)	0.7	2.2
McGill Affective (0-12)	0.6 ± 1.3 (33)	2.67 ± 2.81 (18)	5.83 ± 3.14 (107)	1	1.8
McGill Sensory (0-33)	4.2 ± 6.5 (33)	9.17 ± 9.26 (18)	17.58 ± 6.32 (107)	0.6	2.1
McGill Total (0-45)	4.8 ± 7.5 (33)	11.83 ± 11.59 (18)	23.42 ± 8.63 (107)	0.8	2.2
McGill punishing (0-3)	0.1 ± 0.4 (33)	0.2 ± 0.7 (18)	1.1 ± 1.2 (107)	0.1	0.9
McGill sharp (0-3)	0.6 ± 1.0 (33)	0.78 ± 1.11 (18)	1.97 ± 0.97 (107)	0.2	1.4
McGill shooting (0-3)	0.5 ± 1.0 (33)	0.61 ± 0.98 (18)	1.78 ± 1.04 (107)	0.1	1.2
McGill sickening (0-3)	0.1 ± 0.3 (33)	0.4 ± 1.0 (18)	1.4 ± 1.1 (107)	0.4	1.2
McGill stabbing (0-3)	0.6 ± 1.0 (33)	0.6 ± 0.9 (18)	1.7 ± 1.0 (107)	0	1.1
McGill throbbing (0-3)	0.5 ± 0.8 (33)	0.78 ± 1.00 (18)	1.75 ± 0.93 (107)	0.4	1.4
SF36 Role Emotional (0-100)	85.7 ± 29.5 (35)	74.1 ± 43.6 (18)	28.9 ± 38.8 (106)	0.3	1.5
PCS Helplessness	3.0 ± 4.7 (35)	3.8 ± 4.3 (18)	11.3 ± 6.2 (103)	0.2	1.4
IBS Rome 1 %	0% (16)	9% (11)	77% (44)		
IBS Rome 2 %	0% (16)	9% (11)	70% (44)		
IBS Rome 3 %	0% (16)	9.1% (11)	68.2% (44)		
IBS %	0% (28)	8% (13)	76% (49)		
**GWI > SC**					
SEID 2015	18.1% (11)	88.9% (9)	79.5% (39)		
FM 1990	0% (36)	10% (20)	43.2% (81)		
FM 2011	0% (14)	27.3 (11)	75% (44)		
Migraine Dx HIS	11.4% (35)	45% (20)	59% (75)		
McGill cramping (0-3)	0.30 ± 0.59 (33)	0.78 ± 1.17 (18)	1.58 ± 1.06 (107)	0.6	1.3
McGill fearful (0-3)	0.09 ± 0.29 (33)	0.28 ± 0.83 (18)	0.93 ± 1.04 (107)	0.3	0.9
McGill gnawing (0-3)	0.12 ± 0.42 (33)	0.56 ± 1.04 (18)	1.14 ± 1.05 (107)	0.6	1.1
McGill heavy (0-3)	0.24 ± 0.61 (33)	1.28 ± 1.18 (18)	1.28 ± 1.13 (107)	1.2	1
McGill hot burning (0-3)	0.27 ± 0.72 (33)	0.94 ± 1.30 (18)	1.34 ± 1.12 (107)	0.7	1
McGill splitting (0-3)	0.18 ±0.46 (33)	0.44 ± 0.98 (18)	1.25 ± 1.13 (107)	0.4	1
McGill tender (0-3)	0.33 ± 0.78 (33)	0.78 ± 1.00 (18)	1.43 ± 0.95 (107)	0.5	1.2
CFSQ Headache (0-4)	0.97 ± 1.17 (34)	2.00 ± 1.33 (18)	2.81 ± 1.10 (108)	0.8	1.6
CFSQ Lymph nodes (0-4)	0.18 ± 0.58 (34)	0.72 ± 0.89 (18)	1.47 ± 1.29 (108)	0.8	1.1

CFSQ Sore throat (0-4)	0.29 ± 0.58 (34)	1.11 ± 1.08 (18)	1.46 ± 1.23 (108)	1	1
Chemical domain	17.97 ± 22.78 (34)	24.56 ± 26.40 (18)	40.50 ± 24.24 (105)	0.3	0.9
Chemical Impact of sensitivities	5.53 ± 10.66 (34)	21.28 ± 30.50 (18)	35.47 ± 25.11 (105)	0.8	1.3
Chemical Other exposures	7.50 ± 10.66 (34)	20.06 ± 23.78 (18)	20.99 ± 14.94 (104)	0.8	1
CMSI Bladder	1.91 ± 2.75 (34)	3.28 ± 3.79 (18)	5.67 ± 4.02 (105)	0.4	1
CMSI Cardiac	0.62 ± 1.39 (34)	2.56 ± 3.03 (18)	3.82 ± 3.49 (103)	0.9	1
CMSI Dyspnea	1.24 ± 1.83 (34)	5.83 ± 5.09 (18)	8.68 ± 5.92 (103)	1.4	1.4
CMSI Ear Sinus	1.91 ± 2.68 (34)	3.72 ± 3.61 (18)	7.70 ± 4.48 (103)	0.6	1.4
CMSI Headache	1.35 ± 2.14 (34)	2.22 ± 2.24 (18)	4.06 ± 2.65 (103)	0.4	1.1
CMSI Neuro	2.38 ± 3.50 (34)	6.17 ± 3.76 (18)	8.58 ± 3.42 (103)	1	1.8
CMSI Rheum	6.00 ± 7.51 (34)	14.06 ± 7.79 (18)	21.87 ± 7.55 (103)	1	2.1
COMPASS Pupillomotor (0-5)	0.9 ± 0.7 (12)	2.1 ± 0.9 (10)	2.9 ± 1.1 (43)	1.5	2
Difficulty score	0.34 ± 0.61 (29)	1.56 ± 1.15 (16)	2.01 ± 0.99 (96)	1.4	1.8
IBS score (0-23)	5.1 ± 6.1 (22)	9.5 ± 8.6 (8)	13.4 ± 7.0 (55)	0.6	1.2
∑Urinary Activities	5.71 ± 5.64 (35)	8.47 ± 8.04 (19)	14.41 ± 8.76 (105)	0.4	1.1
CPSS sum	36.45 ± 18.66 (22)	65.00 ± 19.94 (7)	69.15 ± 17.50 (59)	1.5	1.8
CESD ∑ (0-60)	10.56 ± 9.19 (34)	16.61 ± 8.01 (18)	27.72 ± 11.55 (101)	0.7	1.5
CESD Anhedonia	3.03 ± 2.54 (34)	4.61 ± 3.15 (18)	6.40 ± 3.19 (108)	0.6	1.1
CESD Depressed	2.50 ± 3.07 (34)	2.72 ± 2.42 (18)	7.06 ± 4.93 (108)	0.1	1
CESD Somatic	4.44 ± 4.11 (34)	8.78 ± 3.54 (18)	12.44 ± 4.05 (108)	1.1	2
CESD≥16 (%)	32% (34)	44% (18)	83% (108)		
GAD7	2.65 ± 4.00 (34)	4.75 ± 3.19 (16)	10.41 ± 6.15 (43)	0.5	1.4
GAD7≥10 (%)	0.15 ± 0.36 (34)	0.06 ± 0.25 (16)	0.52 ± 0.50 (103)		
MASQ ∑Anxious Arousal	13.25 ± 1.96 (12)	19.20 ± 2.86 (10)	25.23 ± 6.51 (43)	2.4	2
MASQ ∑General Distress	13.92 ± 4.17 (12)	20.30 ± 7.41 (10)	24.95 ± 8.75 (103)	1	1.4
MPTSD ∑	68.00 ± 10.37 (25)	72.11 ± 19.17 (9)	98.37 ± 24.55 (97)	0.3	1.3
MPTSD situations military	1.56 ± 0.87 (25)	1.56 ± 1.33 (9)	2.73 ± 24.55 (97)	0	0.9
MPTSD control worrying	0.26 ± 0.57 (34)	0.38 ± 0.50 (16)	1.30 ± 1.14 (103)	0.2	1
MPTSD easily annoyed	0.59 ± 0.74 (34)	1.13 ± 0.89 (16)	1.80 ± 1.10 (103)	0.7	1.2
MPTSD feeling afraid	0.15 ± 0.36 (34)	0.19 ± 0.40 (16)	1.12 ± 1.08 (103)	0.1	1
MPTSD feeling nervous	0.50 ± 0.96 (34)	0.94 ± 1.06 (16)	1.62 ± 1.09 (103)	0.4	1.1
MPTSD had more close friends	1.68 ± 1.03 (25)	2.33 ± 1.66 (9)	3.72 ± 1.43 (97)	0.5	1.5
MPTSD hard to sit still	0.26 ± 0.51 (34)	0.31 ± 0.60 (16)	1.32 ± 1.07 (103)	0.1	1.1
MPTSD keep job separation	2.16 ± 1.31 (25)	3.44 ± 1.51 (9)	3.59 ± 1.46 (97)	0.9	1
MPTSD memory good as ever	3.16 ± 1.43 (25)	3.78 ± 1.30 (9)	4.61 ± 0.77 (97)	0.4	1.5
MPTSD no one understands	1.48 ± 0.77 (25)	2.22 ± 1.39 (9)	3.06 ± 1.42 (97)	0.8	1.2
MPTSD remind military upset	1.48 ± 0.77 (25)	1.67 ± 1.32 (9)	2.75 ± 1.38 (97)	0.2	1
MPTSD trouble concentrating	2.04 ± 1.10 (25)	3.22 ± 1.30 (9)	3.69 ± 1.18 (97)	1	1.4
MPTSD trouble relaxing	0.50 ± 0.83 (34)	1.13 ± 1.15 (16)	1.82 ± 1.01 (103)	0.7	1.4
MPTSD worrying different	0.38 ± 0.74 (34)	0.69 ± 0.60 (16)	1.44 ± 1.13 (103)	0.4	1
SF36 Mental Health	75.09 ± 14.62 (35)	66.89 ± 17.24 (18)	50.08 ± 22.15 (106)	0.5	1.2
∑PCL-C	23.83 ± 8.05 (12)	30.50 ± 15.07 (10)	50.56 ± 17.02 (43)	0.5	1.7
∑TIQ	36.03 ± 13.07 (29)	43.09 ± 15.55 (11)	58.88 ± 19.67 (61)	0.5	1.2
∑True of You	21.59 ± 6.59 (22)	26.25 ± 7.55 (8)	32.41 ± 6.88 (61)	0.7	1.6
Big 5 Neuroticism	11.40 ± 6.15 (25)	13.11 ± 7.37 (9)	17.76 ± 7.27 (94)	0.3	0.9

BPCQ Internal Score	15.96 ± 4.76 (23)	12.29 ± 4.86 (7)	11.14 ± 4.40 (59)	0.7	1.1
BPCQ Powerful Doctors	10.30 ± 4.09 (23)	12.43 ± 2.37 (7)	14.66 ± 3.64 (59)	0.5	1.1
PBPI Constance	-0.56 ± 1.55 (22)	-0.04 ± 1.00 (7)	1.40 ± 0.71 (61)	0.4	1.9
PBPI Permanence	-0.44 ± 1.23 (22)	0.40 ± 1.06 (7)	0.80 ± 1.10 (61)	0.7	1.1
PCS Magnification	1.71 ± 2.74 (35)	2.33 ± 2.45 (18)	5.30 ± 3.34 (103)	0.2	1.1
PCS Rumination	2.57 ± 3.94 (35)	3.83 ± 3.59 (18)	8.47 ± 4.69 (103)	0.3	1.3
STAI	34.87 ± 12.33 (23)	45.25 ± 17.99 (8)	53.61 ± 13.61 (103)	0.7	1.4
USCD Dyspnea (0-25)	5.6 ± 5.2 (21)	18.3 ± 19.8 (8)	35.7 ± 31.1 (60)	1.1	1.1
IBS Mixed type (%)	0% (28)	6.3% (16)	37.5% (56)		

End

SC v ME/CFS v GWI in Females

Questionnaire and diagnostic outcomes were compared between female ME/CFS, GWI and SC groups with significance reported after ANOVA, Tukey and Bonferonni corrections for multiple comparisons for the 300 variables ($p<0.05$) (SOM Table S2).

GWI > CFS > SC females: The McGill Pain questionnaire Total and Sensory scores and sum of the Chronic Multisymptom Inventory (CMSI) were stratified as GWI > ME/CFS > SC indicating the significantly greater pain and interoceptive complaints in GWI females than ME/CFS and control.

GWI = ME/CFS > SC females: GWI and ME/CFS females had equivalent rates of diagnosis by CCC criteria and 1994 Fukuda criteria for ME/CFS, SEID, and 1990, 2010 and 2011 Fibromyalgia criteria.

Fatigue was equivalent in GWI and ME/CFS based on the Multidimensional Fatigue Inventory domains and Chalder Fatigue scores. Tiredness was apparent from the CISR. All scores for the CFSQ were moderate or higher for fatigue, PEM and sleep. Sore throat and lymph node scores were low and not significantly different from control. Muscle pain, joint pain, and the qualities of aching and tiring pain from the McGill Pain questionnaire were elevated. Migraines were diagnosed by International Headache Society criteria (103) in 71% of ME/CFS, 78% of GWI and 23% of SC. Interoceptive complaints were common and contributed to high Center for Epidemiology Depression (CESD) Somatic Factor scores. The summed CESD scores ≥ 16 / 60 has been used as a proxy for depression; more ME/CFS (65.5%) and GWI (74.4%) exceeded this threshold compared to the control (12.5%) group. However, the elevated scores were due to the high Somatic Factor scores and not Depressed or Anhedonia Factors. Weighted Pupillomotor scores were increased indicating blurred eyesight and other “autonomic” visual complaints. Disability was severely impacted based on SF36 scores that were significantly lower than control for bodily pain, general health, physical function, role physical, social function and vitality. Disability is an important but often unreported component for the diagnosis of ME/CFS and GWI. The data supports the use of SF36 or other quality of life instruments as Common Data Elements during the evaluation and reporting of ME/CFS and GWI subjects and studies.

GWI > ME/CFS = SC females: GWI females had higher scores for Irritable Bowel Syndrome questionnaire (17.5 ± 5.1) compared to ME/CFS (8.3 ± 6.3) and SC (5.3 ± 7.9). Elevated scores for cramping pain on the McGill questionnaire and a second questionnaire based on the Rome 1 criteria supported this finding. Higher rates of Irritable Bowel Syndrome may be an important distinguishing feature between GWI and ME/CFS.

GWI > SC females: A number of outcomes were significantly worse in GWI than controls, but ME/CFS women had intermediate results that were not different from the other two groups. Pressure thresholds, the number of thumb pressure tender points, and widespread pain were higher in GWI than SC. Widespread pain and tenderness were consistent with the diagnosis of fibromyalgia by 1990 criteria. The quality of pain was tender, sharp, shooting, stabbing, gnawing, hot burning and sickening. There were widespread interoceptive complaints of headache, chest discomfort and shortness of breath with exertion (99). The Chemical Exposures questionnaire indicated that irritant chemicals had significant impacts indicating chemical sensitivity (105). Women were easily annoyed, had trouble concentrating and scored high for anxious arousal on the MASQ (112). They had worse scores for SF-36 Role Emotional than controls.

SOM Table S2. Questionnaire responses in ME/CFS, GWI and SC females. Differences between groups were determined by ANOVA, Tukey's Honest Significant Difference for multiple comparison then Bonferroni for 300 domains and other variables ($p<0.05$). Hedges' g was calculated for significant differences between SC and ME/CFS and GWI. Mean±SD (N)

Females	SC Female	ME/CFS Female	GWI Female	g SC v ME/CFS	g SC v GWI
GWI > ME/CFS >SC					
McGill Pain Sensory (0 to 33)	4.7 ± 6.8 (24)	12.7 ± 7.2 (55)	19.2 ± 7.2 (42)	1.1	2.0
McGill Pain Total (0 to 45)	5.5 ± 8.1 (24)	16.1 ± 9.2 (55)	25.0 ± 10.0 (42)	1.2	2.1
Chronic Multisystem Illness (0 to 172)	19.3 ± 20.7 (24)	54.1 ± 25.8 (55)	79.6 ± 35.0 (43)	1.4	1.9
GWI = ME/CFS > SC					
SEID 2015	0% (4)	93.8% (16)	71.4% (7)		
CCC 2003	8.3% (12)	92.3% (26)	83.3% (12)		
CFS Fukuda 1994	8.3% (12)	96.3% (27)	83.3% (12)		
FM 1990	9.1% (24)	49.2% (65)	61.9% (42)		
FM 2010	0% (11)	70.4% (27)	83.3% (12)		
FM 2011	0% (11)	70.4% (27)	83.3% (12)		
Migraine IHS criteria	22.7% (22)	71.4% (63)	77.8% (27)		
CFSQ exertion (0 to 4)	1.1 ± 1.6 (24)	3.5 ± 0.9 (63)	3.4 ± 0.9 (44)	2.1	1.9
CFSQ fatigue (0 to 4)	1.2 ± 1.3 (24)	3.6 ± 0.6 (63)	3.7 ± 0.5 (44)	2.9	2.8
CFSQ headaches (0 to 4)	0.8 ± 1.2 (24)	2.4 ± 1.3 (63)	2.7 ± 1.3 (44)	1.3	1.4
CFSQ joint pain (0 to 4)	0.7 ± 0.9 (24)	2.3 ± 1.3 (63)	3.0 ± 1.1 (44)	1.4	2.2
CFSQ memory (0 to 4)	1.2 ± 1.1 (24)	3.1 ± 0.8 (63)	3.0 ± 0.7 (44)	2.1	2.0
CFSQ muscle pain (0 to 4)	1.2 ± 1.5 (24)	2.8 ± 1.1 (63)	3.1 ± 1.0 (44)	1.3	1.6
CFSQ sleep (0 to 4)	1.6 ± 1.7 (24)	3.4 ± 0.9 (63)	3.5 ± 0.8 (44)	1.5	1.6
Chalder Fatigue Index (0 to 33)	13.7 ± 5.6 (24)	22.6 ± 7.2 (58)	25.0 ± 4.4 (42)	1.3	2.3
CISR ∑ (0 to 6)	1.5 ± 2.4 (23)	5.7 ± 0.7 (56)	5.7 ± 0.7 (44)	2.9	2.8
MFI Σ (0 to 100)	39.3 ± 15.2 (12)	78.5 ± 13.7 (32)	76.9 ± 12.8 (31)	2.7	2.7
MFI Reduced Motivation (0 to 20)	6.8 ± 2.5 (12)	12.4 ± 4.2 (32)	12.1 ± 2.7 (31)	1.5	2.0
MFI Reduced Activity (0 to 20)	6.9 ± 3.4 (12)	16.9 ± 3.3 (32)	16.1 ± 3.5 (31)	2.9	2.6
MFI Physical Fatigue (0 to 20)	9.2 ± 5.7 (12)	16.6 ±3.7 (32)	15.8 ± 3.2 (31)	1.7	1.6
MFI Mental Fatigue (0 to 20)	7.6 ± 3.7 (12)	14.4 ± 4.3 (32)	15.5 ± 3.5 (31)	1.6	2.2
MFI General Fatigue (0 to 20)	8.8 ± 4.8 (12)	18.1 ± 3.0 (32)	17.4 ± 3.2 (31)	2.5	2.2
CESD Sum ≥16/60 (%)	12.5% (24)	65.5% (58)	74.4% (43)		
CESD Somatic Factor (0 to 21)	3.5 ± 3.9 (24)	10.0 ± 4.3 (58)	11.4 ± 3.8 (43)	1.5	2.1

Chemical Sensitivity symptoms (0 to 100)	12.1 ± 12.7 (20)	38.9 ± 23.5 (48)	51.4 ± 27.1 (41)	1.3	1.7
CMSI Neuro (0 to 16)	2.0 ± 3.1 (24)	6.7 ± 3.8 (55)	9.2 ± 3.9 (43)	1.3	1.9
CMSI Rheum (0 to 44)	6.3 ± 7.4 (24)	17.7 ± 7.5 (55)	21.9 ± 9.1 (43)	1.5	1.8
COMPASS Pupillomotor (0 to 5)	1.1 ± 0.6 (11)	2.5 ± 0.9 (24)	2.7 ± 1.0 (13)	1.8	1.9
McGill aching (0 to 3)	0.8 ± 1.2 (23)	2.2 ± 0.9 (55)	2.4 ± 0.8 (43)	1.4	1.7
McGill tiring (0 to 3)	0.6 ± 1.0 (23)	1.9 ± 1.2 (55)	2.5 ± 1.0 (43)	1.2	2.0
Difficulty Score (0 to 3)	0.3 ± 0.6 (15)	1.8 ± 1.0 (39)	2.0 ± 1.0 (37)	1.6	1.8
SF36 SF10 (0 to 100)	69.4 ± 31.4 (24)	16.3 ± 17.1 (59)	16.2 ± 13.7 (43)	2.4	2.4
SF36 Role Physical (0 to 100)	67.7 ± 46.9 (24)	7.6 ± 21.9 (59)	4.7 ± 14.7 (43)	1.9	2.1
SF36 Vitality (0 to 100)	59.0 ± 24.0 (24)	16.8 ± 14.0 (59)	19.2 ± 14.3 (43)	2.4	2.1
SF36 Social Function (0 to 100)	79.7 ± 27.0 (24)	28.6 ± 24.5 (59)	31.7 ± 22.1 (43)	2.0	2.0
SF36 General Health (0 to 100)	71.5 ± 21.1 (24)	34.1 ± 21.1 (59)	33.8 ± 19.6 (43)	1.8	1.8
SF36 Bodily Pain (0 to 100)	80.3 ± 25.8 (24)	41.4 ± 24.2 (59)	31.6 ± 23.2 (43)	1.6	2.0
SF36 Physical Function (0 to 100)	85.2 ± 21.4 (24)	41.2 ± 23.9 (59)	40.0 ± 25.5 (43)	1.9	1.8
GWI > ME/CFS = SC					
CMSI Ear Sinus (0 to 20)	1.4 ± 2.9 (24)	3.6 ± 3.4 (55)	7.5 ± 4.9 (43)	0.7	1.4
McGill cramping (0 to 3)	0.6 ± 1.0 (23)	0.9 ± 1.0 (55)	1.8 ± 1.1 (43)	0.3	1.2
IBS score (0 to 23)	5.3 ± 7.9 (8)	8.3 ± 6.3 (25)	17.5 ± 5.1 (29)	0.4	2.1
IBS Rome I score (0 to 32)	3.1 ± 5.1 (24)	8.1 ± 7.3 (55)	15.9 ± 8.3 (43)	0.7	1.7
Rhinorrhea Score (IRS) (0 to 32)	3.5 ± 5.0 (23	6.0 ± 6.0 (54)	12.8 ± 7.2 (43)	0.4	1.4
Rhinitis score (Nasal) (0 to 40)	5.0 ± 5.3 (23)	10.5 ± 7.1 (54)	17.3 ± 8.2 (43)	0.8	1.7
Rhinitis score (Nasal + Chest) (0 to 76)	6.1 ± 6.7 (23)	14.9 ± 11.0 (54)	26.0 ± 14.2 (43)	0.9	1.6
Irritant Rhinitis Score (IRS) (0 to 64)	8.0 ± 9.7 (23)	14.7 ± 11.9 (54)	26.7 ± 14.5 (43)	0.6	1.4
GWI > SC					
Dolorimetry (kg)	4.2 ± 2.0 (24)	3.0 ± 1.4 (65)	2.4 ± 1.3 (51)	0.7	1.1
FM1990 Widespread Pain (%)	18.1% (22)	59.3% (59)	89.1% (37)		
FM1990 Positive thumb pressure at ≥ 11/18 points (%)	20.8% (24)	44.6% (65)	73.1% (52)		
Tender points (0 to 18)	5.1 ± 5.9 (24)	10.1 ± 5.6 (62)	12.9 ± 4.9 (48)	0.9	1.5
McGill ∑ Affective Score (0 to 12)	0.8 ± 1.6 (24)	3.4 ± 3.0 (55)	5.8 ± 3.3 (42)	0.9	1.8
McGill gnawing (0 to 3)	0.3 ± 0.7 (23)	0.8 ± 1.1 (55)	1.5 ± 1.2 (43)	0.5	1.2
McGill hot burning (0 to 3)	0.0 ± 0.2 (23)	0.9 ± 1.1 (55)	1.4 ± 1.1 (43)	0.9	1.5

McGill sharp (0 to 3)	0.3 ± 0.6 (23)	1.3 ± 1.2 (55)	2.0 ± 0.9 (43)	0.9	1.9
McGill shooting (0 to 3)	0.3 ± 0.5 (23)	1.0 ± 1.1 (55)	1.7 ± 1.1 (43)	0.8	1.5
McGill sickening (0 to 3)	0.1 ± 0.3 (23)	0.7 ± 1.0 (55)	1.3 ± 1.1 (43)	0.6	1.3
McGill stabbing (0 to 3)	0.3 ± 0.6 (23)	0.9 ± 1.1 (55)	1.6 ± 1.2 (43)	0.6	1.2
McGill tender (0 to 3)	0.5 ± 1.0 (23)	1.6 ± 1.2 (55)	2.1 ± 0.9 (43)	1.0	1.7
Chemical Sensitivity chemical (0 to 100)	13.4 ± 16.6 (20)	39.6 ± 26.0 (48)	50.8 ± 25.0 (41)	1.1	1.6
Chemical Sensitivity impact of sensitivities (0 to 100)	5.1 ± 7.6 (20)	31.6 ± 28.8 (48)	44.8 ± 30.7 (41)	1.1	1.5
Chemical Sensitivity other exposures (0 to 100)	9.8 ± 12.5 (20)	24.6 ± 17.1 (48)	32.3 ± 17.4 (41)	0.9	1.4
Borg Dyspnea score (0 to 10)	0.3 ± 0.5 (23)	1.5 ± 1.4 (56)	1.9 ± 1.5 (33)	1.0	1.3
CMSI Dyspnea (0 to 20)	1.8 ± 2.7 (24)	6.7 ± 5.5 (55)	8.4 ± 5.4 (43)	1.0	1.4
CMSI Cardiac (0 to 16)	0.5 ± 1.4 (24)	2.9 ± 3.0 (55)	4.3 ± 3.9 (43)	0.9	1.1
CMSI Headache (0 to 16)	1.0 ± 2.0 (24)	2.5 ± 2.3 (55)	4.2 ± 2.7 (43)	0.7	1.3
Rhinitis Score (Chest) (0 to 36)	1.1 ± 1.9 (23)	4.4 ± 5.5 (54)	8.6 ± 7.2 (43)	0.7	1.2
Irritant Rhinitis Congestion (0 to 32)	4.5 ± 5.3 (23)	8.7 ± 7.2 (54)	13.9 ± 8.3 (43)	0.6	1.3
COMPASS-31 ∑ (0-100)	10.5 ± 6.8 (11)	24.5 ± 8.3 (24)	30.9 ± 11.3 (13)	1.7	2.1
CPSS sum (0 to 22)	33.3 ± 17.4 (12)	58.5 ± 17.8 (30)	66.3 ± 17.6 (29)	1.4	1.8
CESD ∑ (0 to 60)	9.6 ± 9.9 (24)	19.9 ± 10.5 (58)	23.9 ± 10.9 (43)	1.0	1.3
GAD7 easily annoyed (0 to 3)	0.4 ± 0.8 (19)	0.9 ± 0.9 (49)	1.6 ± 1.1 (43)	0.6	1.2
MASQ ∑Anxious Arousal (10 to 50)	14.5 ± 3.1 (11)	22.3 ± 5.3 (25)	24.2 ± 6.3 (13)	1.6	1.8
MPTSD trouble concentrating (1 to 5)	1.4 ± 1.1 (8)	3.1 ± 1.1 (18)	3.9 ± 0.9 (34)	1.5	2.7
SF36 Role Emotion (0 to 100)	84.7 ± 32.6 (24)	63.3 ± 44.9 (59)	34.1 ± 40.8 (43)	0.5	1.3

Principal component analysis (PCA) for males

Principal component analysis (PCA) was performed for all males (SOM Table S3), CCC defined ME/CFS (SOM Table S4), Kansas plus CMI GWI males (SOM Table S5), and SC males (SOM Table S6).

PCA was dominated by CESD, GAD7, McGill and dolorimetry measures. Component 1 (20.2% variance) included SF36 domains (V, RP, SF, BP, PF); CFSQ scores for fatigue, PEM, cognitive, sleep and myalgia; McGill Total, Sensory and Affective summary pain scores; and interoceptive complaints from the CESD Somatic Component and Chemical Exposures symptoms scales (SOM Table S3). This was consistent with disability, core ME/CFS symptoms, pain and interoceptive symptoms in this cohort of men. Component 2 (10.2% variance) covered psychoactive areas including scores for MPTSD and CESD Depressed, Anhedonia and Interpersonal domains. Component 3 (7.5% variance) collected interoceptive complaints including Dyspnea and IBS scores. Component 4 (7.0%) was diagnosis by each of the Rome criteria for IBS. Component 5 (6.3% variance) included diagnosis of ME/CFS by CCC and Fukuda criteria, FM by 2010 and 2011 criteria that rely on fatigue, cognitive, sleep as well as pain symptoms, and PTSD by PCL-C. Component 6 (5.7% variance) encompassed dolorimetry, tender point counts and FM 1990 diagnosis. Component 7 (4.8% variance) included the Chemical Exposures domains. Component 8 (3.4% variance) was Migraine headache severity.

PCA was repeated separately for ME/CFS, GWI and SC males.

In **ME/CFS males**, component 1 (35% variance) was dominated by dolorimetry pressures (kg), McGill Pain scores and sensations (SOM Table S4). Component 2 (12% variance) had McGill Affective score, and Chemical Exposures domains. Component 3 (10% variance) included ME/CFSQ items for fatigue, PEM, memory and sleep for Fukuda CFS diagnosis, and SF36 domains indicating disability. Component 4 (7% variance) included McGill pain measures and CMSI Neurological and Gastrointestinal domain scores. Component 5 (6% variance) linked tender point counts, diagnosis by 1990 FM criteria, and urine complaints.

Each component for **GWI males** (n=104) had low % variance beginning with 10% for Component 1 that included anxiety, catastrophizing, anhedonia and depression (SOM Table S5). Fatigue, PEM, and social function domain were in component 2, with McGill pain domains and symptoms in component 3. Component 4 was dolorimetry (kg), tender point counts and FM 1990 diagnosis. Component 5 had McGill Sensory domain (6% variance).

SC males (n=36) were characterized by component 1 (43% variance) with scores in the normal range for SF36 domains and CFSQ items (SOM Table S6). Component 2 (8% variance) included Chemical Exposures and CMSI scores. Components 3 (7% variance) and 4 (5% variance) had McGill Affective score and urinary symptoms with other McGill items. Rhinitis scores were in component 5 (5% variance).

SOM Table S3. Rotated PCA component matrix of factor loadings in males.

**Rotated Component Matrix**	1	2	3	4	5	6	7	8
% of Variance	20.236	10.209	7.542	6.981	6.318	5.660	4.802	3.367
Predominant questionnaire domains	SF Ftg McG Intero	Anhedonia MPTSD	CMSI	Rome	CCC Fuk FM2010 PCL	Dolo TP FM1990	Chemical	Migraine
SF10	-0.892	-0.216	-0.197	-0.078	-0.134	-0.088	-0.127	-0.093
CFSQ fatigue	0.882	0.185	0.097	0.096	0.057	0.086	0.087	0.105
sf36_v	-0.837	-0.203	-0.109	-0.067	-0.139	-0.084	-0.072	-0.122
CFSQ exertion	0.830	0.094	0.219	0.032	0.113	0.183	0.079	0.068
sf36_rp	-0.828	-0.172	-0.132	-0.100	-0.138	-0.057	-0.167	-0.034
sf36_sf	-0.778	-0.296	-0.241	-0.070	-0.093	-0.091	-0.025	-0.145
CFSQ sleep	0.769	0.176	0.113	0.067	0.015	0.143	-0.078	0.101
sf36_gh	-0.758	-0.333	-0.152	-0.054	-0.092	-0.151	-0.170	-0.084
CFSQ memory	0.754	0.187	0.044	0.034	0.049	0.105	0.064	0.031
sf36_bp	-0.691	-0.206	-0.260	-0.097	-0.208	-0.177	-0.213	-0.172
Chalder Fatigue	0.683	0.236	0.258	0.090	0.193	0.078	0.169	0.005
CFSQ muscle pain	0.621	0.174	0.172	0.099	0.191	0.212	0.140	0.093
CESD_Somatic Factor	0.615	0.568	0.227	0.073	0.108	0.167	0.066	0.055
sf36_pf	-0.589	-0.128	-0.448	0.034	-0.020	-0.196	-0.132	-0.043
CMSI Neuro	0.576	0.203	0.496	0.144	0.188	0.118	0.210	0.063
McGill Total Score	0.516	0.280	0.405	0.123	0.167	0.268	0.259	0.214
C:symptoms	0.510	0.320	0.278	0.208	0.089	0.080	0.451	0.109
McGill Affective Score	0.501	0.309	0.412	0.069	0.131	0.137	0.225	0.125
McGill Sensory Score	0.489	0.250	0.375	0.137	0.171	0.303	0.256	0.236
CESD Depressed	0.206	0.879	0.234	0.037	0.046	0.064	0.048	0.086
CESD sum	0.408	0.825	0.207	0.065	0.114	0.145	0.099	0.094
sf36_mh	-0.318	-0.790	-0.201	-0.156	-0.096	0.006	-0.065	-0.025
CESD Interpersonal	0.101	0.711	0.119	-0.073	0.052	0.095	0.113	0.160
CESD Anhedonia	0.281	0.698	0.054	0.120	0.183	0.165	0.156	0.077
CESD score ≥16	0.380	0.669	-0.032	0.143	0.081	0.148	0.198	0.126
MPTSD sum	0.253	0.584	0.328	0.065	0.073	0.059	0.156	0.004
sf36_re	-0.370	-0.537	-0.235	-0.125	-0.129	-0.032	-0.108	0.210
CMSI Bladder	0.201	0.222	0.654	0.160	0.104	0.169	0.058	0.083
CMSI Sum	0.478	0.311	0.651	0.184	0.141	0.214	0.243	0.109
CMSI SOB	0.373	0.255	0.625	0.012	0.061	0.205	0.180	0.041
CMSI Cardiac	0.311	0.231	0.605	-0.004	0.102	-0.014	0.051	0.143
CMSI Rheum	0.547	0.258	0.558	0.120	0.114	0.200	0.236	0.054
CMSI EarSinus	0.306	0.193	0.469	0.198	0.180	0.136	0.224	0.045
CMSI GI Rome I	0.373	0.320	0.465	0.313	0.129	0.226	0.260	0.064
IBS Rome3	0.037	0.090	0.063	0.884	0.216	0.193	0.033	0.069
IBS Rome1	0.072	0.105	0.099	0.862	0.315	0.143	0.041	0.070
ISBS Rome2	0.037	0.097	0.076	0.851	0.267	0.185	0.061	0.038
IBS sum	0.244	0.060	0.102	0.826	0.213	0.147	0.124	0.046
Carruthers CCC	0.386	0.057	0.056	0.255	0.772	0.007	0.049	-0.009

CFS Fukuda 1994	0.323	0.062	0.027	0.346	0.753	0.016	0.030	-0.012
FM_2011	0.131	0.183	0.167	0.272	0.706	0.223	0.000	0.111
FM_2010	0.186	0.059	0.231	0.232	0.657	0.190	0.047	0.052
PCL-C	0.063	0.455	0.120	0.201	0.571	0.144	0.087	0.084
SEID	0.293	-0.010	-0.098	0.243	0.517	-0.090	0.103	-0.048
Multiple Chemical Sensitivity	-0.039	0.280	0.128	0.154	0.403	0.020	0.257	-0.027
Tender Point Count ≥ 11/18	0.176	0.163	0.175	0.150	-0.022	0.844	0.001	0.071
Tender Point Count	0.250	0.211	0.167	0.167	0.069	0.832	0.093	0.031
Dolorimetry (kg)	-0.361	-0.072	-0.129	-0.163	-0.087	-0.747	-0.178	-0.055
FM_1990	0.167	0.042	0.056	0.294	0.196	0.706	0.053	0.040
C:other_exposures	0.246	0.130	0.108	0.098	0.092	0.084	0.786	0.090
C:impact_of_sensitivities	0.277	0.252	0.266	0.111	0.098	0.072	0.733	0.024
C:chemical_domain	0.215	0.268	0.201	-0.013	0.067	0.160	0.691	0.087
Migraine without Aura MO HIS	0.179	0.118	0.054	0.013	0.047	0.053	-0.023	0.879
Migraine HIS	0.323	0.107	0.205	0.173	0.074	-0.001	0.220	0.717
CMSI Headache	0.258	0.247	0.482	0.153	0.048	0.263	0.251	0.493
CFSQ Headaches	0.472	0.201	0.236	0.146	-0.015	0.258	0.123	0.490

SOM Table S4. PCA for ME/CFS Males. Data for ME/CFS males (n=18) were analyzed by Varimax rotation and Kaiser normalization.

ME/CFS Male Predominant items	McGill Dolorimetry	Chemical Exposures (C:) McGill Affective	Somatic CFSQ	McGill sharp	Tender Bladder
% of Variance	34.94	12.084	10.037	7.351	5.659
Component	1	2	3	4	5
McG_heavy	0.879	0.176	0.194	0.034	0.155
McG_tiring	0.838	0.203	0.199	0.212	0.099
cfsq_muscle_pain	0.821	0.168	0.2	0.142	0.3
McG_aching	0.796	0.146	0.221	0.314	0.206
CmSOB	0.738	0.142	0.045	0.186	0.209
sf36_bp	-0.704	-0.27	-0.264	-0.161	-0.109
McG_hot_burning	0.68	-0.014	0.001	0.319	0.177
McGSen33	0.664	0.312	0.077	0.546	0.284
McG_Total45	0.654	0.419	0.087	0.505	0.266
CmRheum	0.635	0.458	0.291	0.309	0.14
Sy_kg_ave	-0.6	-0.016	-0.455	-0.111	-0.099
McG_cramping	0.594	0.425	0.119	0.168	0.084
cfsq_joint_pain	0.586	0.187	0.199	0.418	0.256
C:impact_of_sensitivities	0.214	0.922	0.1	0.081	0.119
C:other_exposures	0.079	0.876	0.199	-0.057	0.065
C:symptoms	0.231	0.83	0.316	0.191	-0.12
C:chemical_domain	0.155	0.826	0.071	0.315	-0.101
CmCardiac	0.061	0.808	0.051	0.301	-0.041
RhSc_chest	0.332	0.758	0.209	0.107	0.066
McG_Affective	0.509	0.7	0.103	0.287	0.16
McG_punishing	0.021	0.682	-0.051	0.226	0.221
McG_fearful	0.139	0.676	0.071	-0.211	0.231
McG_sickening	0.219	0.676	0.009	0.559	-0.028
Rumination	0.529	0.641	-0.104	0.015	0.261
McG_splitting	0.298	0.604	0.015	0.492	0.083
Thumb11	0.399	-0.486	0.025	0.373	0.435
RhSc Rhinitis	0.268	0.466	0.355	0.127	-0.408
Helplessness	0.365	0.462	-0.147	0.295	0.442
∑CISR	0.159	0.017	0.918	0.125	0.053
cfsq_sleep	-0.01	0.017	0.887	0.273	0.036
cfsq_fatigue	0.038	0.221	0.887	0.016	-0.024
sf36_rp	-0.101	-0.155	-0.875	-0.052	-0.038
sf36_v	-0.384	-0.139	-0.836	0.134	0.092
cfsq_memory	-0.099	-0.029	0.74	-0.13	-0.079

sf36_gh	-0.353	-0.059	-0.724	0.205	0.03
sf36_sf	-0.071	-0.215	-0.715	-0.07	-0.033
CESD Somatic factor	0.531	-0.028	0.652	-0.068	-0.047
cfsq_exertion	0.192	0.247	0.615	0.118	0.372
cfsq_Ftg&4/8	0.418	0.052	0.603	0.123	0.134
sf36_pf	-0.321	-0.33	-0.438	-0.205	-0.292
CmEarSinus	0.078	-0.03	0.144	0.902	0.112
McG_stabbing	0.367	0.231	0.018	0.756	0.165
McG_throbbing	0.398	0.186	-0.038	0.739	0.008
McG_shooting	0.326	0.367	0.003	0.716	0.197
CMSI GI Rome_I	0.202	0.254	0.097	0.709	0.303
McG_sharp	0.357	0.324	0.024	0.667	0.204
CMSI Neuro	0.366	0.383	0.38	0.444	0.086
Magnification	0.341	0.199	0.013	-0.075	0.813
Urinary score	0.305	0.071	0.116	0.361	0.785
CMSI Bladder	0.199	0.304	0.023	0.383	0.674
McG_tender	0.443	0.194	0.062	0.204	0.639
FM_1990	0.32	-0.447	-0.025	0.307	0.635
TP_ave	0.43	-0.493	-0.012	0.123	0.569
McG_gnawing	0.329	0.12	-0.022	0.456	0.539

SOM Table S5. PCA for GWI Males. GWI male data were analyzed by PCA with Varimax rotation and Kaiser normalization (n=104).

Rotated Component Matrix<br>Predominant Variables	CESD<br>GAD	Fatigue<br>SF36	McGill<br>Affective	Dolorimetry	McC<br>Sensory
% of Variance	9.816	6.491	6.433	6.371	5.657
Component	1	2	3	4	5
sf36_mh	-0.867	-0.111	-0.097	0.06	-0.039
CESD_Dep3,6,9,10,14,17,18	0.83	0.057	0.053	-0.011	0.061
∑GAD7	0.761	0.024	0.229	-0.073	0.194
Helplessness	0.729	0.175	0.319	0.207	0.102
Rumination	0.712	0.098	0.197	0.19	0.047
Magnification	0.669	0.027	0.261	0.088	0.092
CESD_Anhedonia	0.669	0.207	-0.041	0.082	0.132
sf36_re	-0.612	-0.008	0.003	0.047	0.014
CESD_Inter15,19	0.587	-0.031	-0.005	0.055	0.153
CESD_Somatic	0.558	0.363	0.092	0.098	0.127
sf36_gh	-0.391	-0.302	-0.077	-0.052	-0.129
sf36_v	-0.112	-0.855	-0.087	0.062	-0.044
cfsq_fatigue	0.139	0.733	0.134	-0.044	0.112
∑CISR	0.164	0.619	0.159	0.152	0.161
cfsq_exertion	-0.08	0.563	0.263	0.171	-0.008
sf36_sf	-0.334	-0.528	-0.074	-0.027	-0.155
McG_tiring	0.021	0.487	0.32	0.071	0.05
cfsq_sleep	0.124	0.438	-0.02	0.085	0.117
McG_Affective	0.284	0.272	0.791	0.066	0.135
McG_fearful	0.39	-0.031	0.713	-0.064	-0.016
McG_punishing	0.274	0.17	0.628	0.078	0.098
McG_sickening	0.117	0.24	0.625	0.108	0.249
McG_Total	0.192	0.209	0.601	0.212	0.526
McG_gnawing	0.071	0.257	0.481	-0.052	0.11
McG_throbbing	-0.023	0.056	0.48	0.27	0.315
Tender Points ≥11/18	0.056	-0.003	0.07	0.888	0.041
Tender Point Count	0.098	0.022	0.013	0.885	0.098
FM_1990	0.014	0.123	0.036	0.88	0.094
Dolorimetry (kg)	0.017	0.017	-0.072	-0.828	-0.146
McG_stabbing	0.128	-0.001	0.068	0.024	0.838
McG_sharp	0.182	0.127	0.019	0.118	0.793
McG_shooting	0.17	0.125	0.102	0.107	0.787
McG Sensory	0.121	0.149	0.425	0.254	0.646

SOM Table S6. PCA for SC males. SC male data (n=36) were analyzed by PCA with Varimax rotation and Kaiser normalization.

SC Males<br>Rotated Component Matrix<br>Predominant items	CFSQ<br>SF36<br>CESD	Chemical Exposures	McGill Bladder	McGill	Rhinitis Scores
% of Variance	43.4%	7.6%	6.8%	5.2%	4.7%
Component	1	2	3	4	5
sf36_gh	-0.841	-0.347	-0.064	-0.174	0.065
∑CISR	0.837	0.313	0.109	0.182	0.069
sf36_v	-0.773	-0.431	-0.095	0.014	-0.028
sf36_sf	-0.771	-0.216	-0.127	-0.036	0.041
CESD_Somatic Factor	0.738	0.132	0.167	0.08	0.019
cfsq_exertion PEM	0.732	0.158	0.368	0.174	0.269
Chalder	0.729	0.296	0.133	0.063	0.106
cfsq_fatigue	0.707	0.233	0.199	-0.026	0.301
sf36_rp	-0.67	-0.148	-0.438	-0.291	-0.1
CmNeuro	0.628	0.396	0.38	0.148	0.181
CFS Fukuda 1994	0.59	0.147	0.114	0.145	0.277
cfsq_sleep	0.568	-0.284	0.138	0.122	0.172
cfsq_memory	0.56	0.078	0.071	0.417	0.47
CMSI Rheum	0.552	0.357	0.406	0.305	0.277
CESD Anhedonia Factor	0.529	0.453	-0.023	0.25	-0.138
sf36_bp	-0.509	-0.17	-0.355	-0.288	-0.131
sf36_pf	-0.468	0.033	-0.231	-0.307	-0.047
C:other_exposures	0.178	0.877	0.147	0.203	0.086
C:impact_of_sensitivities	0.249	0.836	0.024	0.31	0.006
C:chemical_domain	0.163	0.766	0.128	0.365	0.119
McG_hot_burning	0.231	0.761	0.282	0.195	0.137
C:symptoms	0.254	0.714	0.297	0.268	0.365
CMSI EarSinus	0.277	0.696	0.016	-0.171	0.148
CMSI GI Rome_I	0.313	0.658	0.166	0.123	0.178
CMSI Sum	0.487	0.552	0.388	0.194	0.23
McG_aching	0.265	0.479	-0.146	0.445	0.205
McGSen33	0.308	0.466	0.384	0.425	0.281
CMSI Headache	0.381	0.459	0.198	0.074	0.223
McG_Total	0.317	0.454	0.413	0.408	0.258
McG_stabbing	0.294	0.44	0.396	0.298	0.101
McG_sickening	0.072	0.177	0.892	0.031	0.101
McG_heavy	0.187	0.167	0.845	0.282	0.151
CmBladder	0.225	0.05	0.822	-0.022	0.082
Urinary activity	0.322	0.119	0.771	0.227	0.076
McG_tender	0.291	0.094	0.555	0.259	0.115

McGill Affective	0.314	0.316	0.502	0.252	0.098
Dolorimetry (kg)	-0.379	-0.185	-0.492	0.087	-0.153
sf36_re	-0.249	0.04	-0.479	-0.186	-0.143
McG_punishing	0.125	0.132	0.048	0.895	0.133
cfsq_LN	0.171	0.211	0.129	0.859	0.157
cfsq_sore_throat	0.198	0.19	0.184	0.767	0.137
CMSI Cardiac	0.216	0.46	0.292	0.653	0.198
McG_gnawing	0.023	0.25	0.414	0.622	0.23
McG_sharp	0.297	0.34	0.236	0.502	0.06
RhSc Chest	0.076	0.067	0.025	-0.017	0.937
RhSc Total	0.082	0.173	0.195	0.228	0.888
RhSc Rhinitis	0.074	0.206	0.26	0.329	0.736
DM_II	-0.075	0.023	0.011	0.081	0.653
McG_cramping	0.183	-0.008	0.397	0.148	0.518

PCA Female SC v ME/CFS v GWI

PCA was performed for 126 women using 77 domain and item scores from tenderness, McGill pain, CFSQ, SF36, Rhinitis, Chemical Exposures and Catastrophizing questionnaires. PCA was repeated for SC, ME/CFS and GWI women. The first five components were discussed for each subgroup.

The first principal component for **all women** (15.4% variance) contained core features of disability and ME/CFS diagnostic criteria with SF36 domain scores for Vitality, Role Physical and Social Function and core measures of fatigue (Chalder Fatigue Index) and tiredness (CISR), postexertional malaise, cognitive and sleep problems (SOM Table S7). The second and third components included McGill pain characteristics (8.8% and 7.8% variance, respectively). The next components contained rhinitis and dyspnea scores (IRS, 7.8% variance) and CESD domain scores (7.2% variance). PCA for ME/CFS and GWI females were predominated by McGill, CESD and dolorimety outcomes.

PCA for ME/CFS females (n=56) had small % variances. Component 1 had 1990 FM diagnosis and McGill items (8% variance) (SOM Table S8). Component 2 (8% variance) included dolorimetry, tender point counts and myalgia items. Component 3 (7% variance) was anxiety and CESD anhedonia, depressed and somatic factors. Component 4 included Chemical Exposure domains and Congestion from the Irritant Rhinitis Score. CMSI domains were in component 5.

PCA for GWI females (n=43) began with catastrophizing, anxiety and depression scores in component 1 (12% variance) (SOM Table S9). Dolorimetry and pain symptoms from ME/CFSQ and McGill questionnaires were in components 2 (12% variance) and 3 (8% variance). Component 4 encompassed Chemical Exposures domains (6% variance). The fifth component had McGill Sensory domain scores (6% variance).

Component 1 (21% variance) for **SC females** was predominantly related to McGill scores (SOM Table S10). Component 2 (12% variance) included CISR, SF36 Vitality, and Chemical Exposures domains. Component 3 (9 % variance) had airway symptoms and tenderness. CESD factor scores and anxiety (GAD7) were in component 4 (8% variance). Component 5 included dolorimetry (6% variance).

SOM Table S7. PCA for all Females.

Rotated Component Matrix						
% of Variance	15.446	8.792	7.831	7.755	7.244	5.013
Component	1	2	3	4	5	6
Predominant questionnaire items	CFSQ Somatic	McGill	McGill Affective	IRS	CESD	Chemical Exposures
∑CISR	0.869	0.116	0.044	0.096	0.059	0.09
sf36_v	-0.865	-0.226	-0.009	-0.072	-0.068	-0.032
sf36_rp	-0.808	-0.165	-0.123	0.012	-0.054	-0.15
cfsq_exertion	0.797	0.194	0.088	0.164	0.054	0.132
cfsq_fatigue	0.794	0.23	0.058	0.153	0.129	0.146
sf36_sf	-0.777	-0.104	-0.063	-0.149	-0.177	-0.008
cfsq_SUM8	0.753	0.293	0.223	0.288	0.075	0.231
cfsq_sleep	0.748	0.24	0.076	0.053	0.156	0.198
cfsq_memory	0.746	0.032	0.136	0.18	0.009	0.065
Chalder Fatigue	0.732	0.033	0.284	0.108	0.123	0.073
sf36_gh	-0.691	-0.253	-0.137	-0.203	-0.234	0.032
sf36_pf	-0.644	-0.228	-0.046	-0.253	-0.019	-0.124
CESD_Som_1,2,5,7,11,13,20	0.589	0.135	0.129	0.229	0.507	0.17
sf36_bp	-0.538	-0.528	-0.261	-0.146	-0.112	-0.175
CmNeuro	0.508	0.095	0.335	0.493	0.139	0.226
cfsq_headaches	0.475	-0.001	0.427	0.206	0.01	0.19
cfsq_joint_pain	0.432	0.405	0.127	0.264	0.179	0.108
cfsq_sore_throat	0.343	0.127	-0.006	0.338	0.095	0.193
McG_tender	0.341	0.691	0.17	0.208	0.238	0.156
McG_aching	0.467	0.658	0.149	0.085	0.105	0.091
McG_heavy	0.351	0.64	0.351	-0.069	0.127	0.131
McG_tiring	0.393	0.618	0.402	0.108	0.177	0.136
WSP	0.266	0.586	0.147	0.003	0.017	0.21
FM_1990	0.172	0.572	0.087	0.237	0.181	0.071
cfsq_muscle_pain	0.49	0.563	0.185	0.107	0.033	0.253
McGSen33	0.335	0.563	0.413	0.219	0.174	0.242
Thumb11	0.099	0.561	0.031	0.242	0.182	0.037
McG_Total45	0.325	0.542	0.522	0.224	0.203	0.211
McG_throbbing	0.13	0.536	0.221	0.299	0.17	0.016
TP_ave	0.223	0.513	0.072	0.231	0.049	0.196
McG_sickening	0.158	0.239	0.73	0.176	0.135	0.191
McG_Affective12	0.245	0.396	0.717	0.2	0.244	0.095
McG_splitting	0.18	0.227	0.697	-0.024	0.128	0.061
McG_punishing	0.141	0.226	0.652	0.115	0.26	-0.111
McG_fearful	0.031	0.101	0.616	0.296	0.246	0.072
CmHA	0.256	0.105	0.593	0.177	0.139	0.139
McG_gnawing	0.116	0.284	0.423	0.174	-0.008	0.416
cmGIRome_I	0.284	0.114	0.416	0.374	0.245	0.29

Rh_chest36_	0.15	0.137	0.036	0.722	0.167	0.186
CmSOB	0.398	0.024	0.071	0.716	0.053	0.048
Rh_rhinitis40_	0.178	0.167	0.106	0.648	0.236	0.194
CmCardiac	0.252	0.082	0.285	0.629	0.143	0.098
Urine∑35_	0.228	0.289	0.107	0.555	-0.059	0.173
IRS_Cong32_	0.125	0.113	0.147	0.55	0.271	0.322
CmRheum	0.488	0.317	0.36	0.495	0.071	0.217
CmBladder	0.25	0.24	0.363	0.48	-0.044	0.133
IRS_Rhino32_	0.1	0.206	0.2	0.466	0.33	0.327
CmEarSinus	0.245	0.013	0.401	0.465	0.185	0.24
sf36_mh	-0.121	-0.164	-0.125	-0.141	-0.842	-0.017
CESD_Dep3,6,9,10,14,17,18	0.204	0.166	0.011	0.159	0.829	0.07
∑GAD7	0.15	0.095	0.208	0.143	0.788	0.122
sf36_re	-0.171	-0.123	-0.284	0.048	-0.691	-0.142
CESD_Anh4,8,12,16	0.057	0.111	0.097	0.113	0.69	-0.013
CESD_Inter15,19	0.007	-0.112	0.056	0.07	0.625	0.1
C:other_exposures	0.284	0.288	0.04	0.225	0.102	0.714
C:impact_of_sensitivities	0.288	0.156	0.204	0.268	0.173	0.712
C:chemical_domain	0.283	0.208	0.12	0.295	0.129	0.702
C:symptoms	0.321	0.15	0.151	0.391	0.189	0.613
McG_cramping	0.154	0.233	0.339	0.249	0.061	0.412
McG_stabbing	0.15	0.224	0.29	0.113	0.157	0.138
McG_shooting	0.184	0.206	0.217	0.279	0.149	0.128
McG_sharp	0.309	0.326	0.252	0.053	0.11	0.269
McG_hot_burning	0.196	0.301	0.111	0.325	0.098	0.032
Rumination_0	0.241	0.259	0.253	-0.003	0.389	0.077
Helplessness_0	0.224	0.252	0.258	0.005	0.432	0.081
Magnification_0	0.183	0.131	0.282	0.124	0.469	0.169
Sin_kg_ave	-0.179	-0.178	-0.149	-0.145	-0.094	-0.125
Sy_kg_ave	-0.243	-0.336	-0.187	-0.155	0.01	-0.178
MO_IHS	0.161	0.044	0.082	0.003	0.2	0.075
Mig_IHS	0.272	0.089	-0.003	0.144	0.117	0.021
BMI	-0.086	0.098	-0.046	0.032	0.106	-0.018
IBS_1	0.191	0.134	0.199	0.188	0.021	0.153
CmURTI	0.12	0.073	0.157	0.028	0.073	0.033
MA_IHS	0.118	0.059	-0.031	0.165	-0.051	0.004
Tension_IHS	0.124	-0.012	0.122	-0.076	0.197	0.089
cfsq_LN	0.351	0.071	0.213	0.378	-0.151	0.2
C:masking_index	-0.063	0.049	0.127	-0.035	0.003	-0.088
DM_II	0.078	0.039	0.045	-0.055	0.088	0.139

SOM Table S8. PCA for CFS Females.

Rotated Component Matrixa	McG	Dolo	CESD	C:	cmsi
% of Variance	7.836	7.637	7.208	5.739	5.369
Component	1	2	3	4	5
McG_Affective12	0.918	0.171	0.158	0.064	0.092
McG_punishing	0.877	0.051	-0.047	0.027	0.015
McG_sickening	0.828	0.089	0.007	0.049	0.028
McG_splitting	0.693	0.07	0.023	0.191	0.032
McG_fearful	0.589	-0.064	0.254	0.061	0.175
McG_Total45	0.589	0.391	0.219	0.242	0.151
McG_tiring	0.541	0.37	0.281	0.062	0.087
McG_gnawing	0.401	0.257	0.26	0.353	0.226
FM_1990	0.092	0.874	-0.009	0.025	0.112
Thumb11	0.033	0.857	0.015	-0.082	0.027
TP_ave	0.018	0.75	-0.085	0.116	0
McG_aching	0.227	0.66	0.217	0.214	-0.113
Sy_kg_ave	-0.277	-0.543	0.182	-0.1	-0.071
cfsq_muscle_pain	0.23	0.532	0.164	0.259	-0.117
WSP	0.228	0.524	0.087	0.137	0.003
cfsq_joint_pain	-0.001	0.449	0.236	0.064	0.17
Sin_kg_ave	-0.203	-0.396	0.011	0.118	-0.087
McG_heavy	0.34	0.393	0.141	0.238	-0.074
sf36_mh	-0.052	-0.02	-0.878	-0.025	-0.054
CESD_Dep3,6,9,10,14,17,18	0.005	0.115	0.852	0.039	-0.001
CESD_Anh4,8,12,16	0.095	-0.034	0.794	0.132	0.089
∑GAD7	0.208	0.048	0.673	0.012	0.057
sf36_re	-0.198	-0.066	-0.588	0	0.089
CESD_Som_1,2,5,7,11,13,20	0.075	-0.052	0.539	0.268	0.325
McG_tender	0.169	0.371	0.457	0.25	0.188
C:other_exposures	0.079	0.165	0.106	0.875	0.154
C:impact_of_sensitivities	0.212	-0.043	0.06	0.849	0.074
C:chemical_domain	0.103	0.07	0.001	0.787	0.043
C:symptoms	0.164	0.348	0.251	0.59	0.188
IRS_Cong32_	-0.211	0.084	0.089	0.503	0.395
CmURTI	0.155	0.304	0.229	-0.395	0.114
CmCardiac	0.009	-0.068	0.115	0.103	0.803
CmNeuro	0.141	0.056	-0.04	0.145	0.705
CmRheum	0.249	0.192	0.048	0.223	0.687
CmSOB	-0.173	-0.043	-0.048	-0.08	0.589

SOM Table S9 . PCA for GWI Females.

Rotated Component Matrixa	CESD	Dolo CFSQ	McG Aff	C:	McG Sens
% of Variance	11.858	11.516	8.106	6.34	6.337
Component	1	2	3	4	5
CESD_Anh4,8,12,16	0.844	-0.085	-0.063	-0.044	0.052
Magnification_0	0.784	0.172	0.137	0.122	0.149
Helplessness_0	0.783	0.242	-0.022	0.025	0.172
Rumination_0	0.776	0.253	0.125	0.135	0.069
CESD_Dep3,6,9,10,14,17,18	0.775	0.191	0.038	0.171	0.027
sf36_mh	-0.766	-0.117	-0.019	-0.111	-0.143
∑GAD7	0.757	0.046	0.086	0.173	0.284
McG_punishing	0.756	0.248	0.236	-0.164	0.234
CESD_Som_1,2,5,7,11,13,20	0.744	0.172	-0.017	0.231	0.088
sf36_gh	-0.417	-0.356	0.033	-0.164	0.08
McG_aching	0.402	0.392	0.072	-0.142	-0.026
sf36_bp	-0.296	-0.783	-0.196	-0.075	-0.122
cfsq_joint_pain	0.31	0.775	0.195	-0.026	0.143
sf36_rp	-0.018	-0.761	-0.015	-0.261	-0.187
McG_tender	0.287	0.724	0.075	-0.059	0.025
McG_tiring	0.095	0.723	0.27	0.187	0.17
cfsq_exertion	0.184	0.699	0.2	0.119	-0.065
WSP	0.051	0.68	-0.143	0.23	0.093
McG_heavy	0.226	0.641	0.284	-0.186	0.185
cfsq_muscle_pain	0.082	0.614	0.222	0.141	0.194
cfsq_fatigue	0.029	0.6	0.057	0.174	0.153
Sy_kg_ave	-0.213	-0.556	0.032	-0.199	-0.197
sf36_v	-0.12	-0.556	-0.02	0.05	0.365
cfsq_headaches	-0.127	-0.029	0.871	0.056	-0.015
CmHA	0.029	0.22	0.871	0.061	0.092
McG_fearful	0.465	0.126	0.617	0.141	0.204
McG_splitting	0.222	0.245	0.55	-0.134	0.303
McG_sickening	0.235	0.247	0.529	0.28	0.252
CmBladder	0.072	0.192	0.527	0.129	0.205
McG_Affective12	0.504	0.402	0.513	0.129	0.272
McG_Total45	0.424	0.432	0.48	0.135	0.468
CmNeuro	0.246	0.281	0.456	0.314	0.164
05_Chld_11	0.159	0.137	0.433	-0.114	0.181
sf36_pf	-0.147	-0.406	0.428	-0.366	-0.106
C:impact_of_sensitivities	0.148	0.128	0.123	0.789	0.292
C:other_exposures	0.186	0.236	-0.081	0.782	-0.082
C:symptoms	0.085	-0.058	0.191	0.766	0.15
C:chemical_domain	0.043	0.214	0.068	0.612	0.357
McG_cramping	-0.019	0.12	0.407	0.571	-0.076

cmGIRome_I	0.268	0.264	0.284	0.422	0.356
McG_stabbing	0.38	0.057	0.167	0.145	0.777
McG_shooting	0.313	0.142	0.214	0.238	0.758
sf36_re	-0.505	-0.217	0.035	-0.034	-0.612
McG_throbbing	0.473	0.325	0.051	-0.081	0.561
McGSen33	0.36	0.419	0.435	0.129	0.528
McG_sharp	0.128	0.204	0.345	0.132	0.47

SOM Table S10. PCA for SC females.

Rotated Component Matrixa	McG	Somatic	Cmsi	CESD	McG
% of Variance	21.105	11.993	9.401	8.086	6.196
Component	1	2	3	4	5
McG_heavy	0.902	0.099	0.163	-0.055	0.316
sf36_bp	-0.891	-0.088	-0.046	0.054	-0.075
cfsq_muscle_pain	0.888	0.111	0.035	-0.055	0.108
McGill Total	0.883	0.152	0.075	-0.036	0.153
McG Sensory	0.868	0.163	0.073	-0.037	0.103
McG_tender	0.86	0.239	-0.039	-0.112	-0.142
McG_tiring	0.858	0.119	-0.016	-0.023	-0.093
McG_aching	0.851	0.324	-0.004	-0.162	0.037
Rumination	0.837	0.306	0.115	0.199	0.267
CmRheum	0.823	0.374	0.221	0.121	0.041
McG_Affective	0.816	0.08	0.072	-0.026	0.35
McG_throbbing	0.803	0.194	0.052	-0.182	0.01
Helplessness	0.802	0.193	0.138	0.351	0.275
McG_gnawing	0.688	0.052	0.176	-0.047	0.358
WSP	0.648	0.135	0.037	-0.026	-0.001
McG_hot_burning	0.561	0.105	0	-0.029	-0.148
Magnification	0.543	0.244	0.149	0.338	0.374
CMSI GI	0.519	0.018	0.216	0.169	0.357
BMI	-0.394	-0.102	-0.219	-0.24	0.169
CESD_Som_1,2,5,7,11,13,20	0.149	0.85	0.116	0.359	-0.105
Chlader Fatigue	0.269	0.819	0.142	0.235	0.136
∑CISR	0.291	0.812	0.295	0.135	0.031
cfsq_sleep	0.287	0.741	0.155	0.172	-0.13
C:symptoms	0.201	0.727	-0.064	0.101	0.115
sf36_v	-0.244	-0.717	-0.463	-0.206	0.027
sf36_gh	-0.382	-0.647	-0.078	-0.417	-0.132
sf36_rp	-0.553	-0.606	-0.372	-0.171	0.127
C:impact_of_sensitivities	0.39	0.598	0.012	0.104	0.157
cfsq_exertion	0.453	0.595	0.444	-0.005	0.091
cfsq_headaches	0.134	0.585	-0.086	0.252	0.015
CMSI BBladder	0.48	0.506	0.396	-0.378	0.179
C:other_exposures	0.402	0.506	-0.029	-0.037	0.144
cfsq_fatigue	0.224	0.469	0.423	0.103	-0.049
MA HIS	-0.244	0.113	0.908	-0.035	-0.127
Rhinitis Score (chest)	0.258	0.094	0.808	-0.137	0.181
CmSOB	0.16	0.216	0.797	-0.086	0.181
CmNeuro	0.202	0.411	0.707	0.353	0.08
Thumb11	0.212	0.031	0.629	-0.108	0.478
CmEarSinus	-0.038	0.505	0.59	-0.199	-0.23
TP_ave	0.271	-0.019	0.498	-0.221	0.441

sf36_mh	0.046	-0.112	0.02	-0.881	0.018
∑GAD7	-0.05	0.286	-0.033	0.839	0.069
sf36_re	0.073	-0.152	0.17	-0.833	0.18
CESD_Dep3,6,9,10,14,17,18	-0.104	0.441	-0.053	0.802	-0.149
CESD_Inter15,19	0.026	0.258	-0.114	0.689	-0.066
sf36_sf	-0.211	-0.359	-0.503	-0.63	-0.166
CESD_Anh4,8,12,16	-0.068	-0.07	0.09	0.566	0.498
Tension_IHS	-0.052	-0.014	-0.241	0.489	0.071
McG_sickening	0.366	-0.015	0.061	-0.003	0.788
McG_punishing	0.543	0.023	0.191	-0.031	0.657
McG_fearful	0.543	0.023	0.191	-0.031	0.657
IBS_1	0.204	0.022	0.546	-0.054	0.604
C:masking_index	0.156	0.094	-0.05	-0.064	0.519

SOM Table S11. Spearman correlations >0.6 were shown in orange, >0.7 in red and < -0.6 in blue.
A.ME/CFS subjects were clustered for dolorimetry and 1990 FM diagnosis, McGill and Q pain scores, Interoception, Chemical Exposures, Rhinitis and Irritant Rhinitis, SF36 domains, MDFI domains, MPTSD and GAD7, CESD domains, PCS, BDI domains, Migraine, IBS, PBPI, CCC diagnosis and Chalder Fatigue, and related CFSQ items.
B. GWI subjects had clusters for dolorimetry and FM 1990, weak correlations for CFSQ pain and FM 2010; SF36 BP and McGill domains, CMSI, rhinitis scores, SF36, CCC and Fukuda criteria, Chemical Exposures, GAD7 and PTSD, MDFI, CESD, PCS, BDI, migraine, IBS, Chalder and other fatigue indices, and PBPI domains.
C. SC had correlated dolorimetry and FM 1990, FM 2010 and CFSQ pain, SF36 BP and McGill, CMSI domains, weak Chemical exposures, Rhinitis, disability, MDFI, GAD7 and MPTSD, CESD, PCS, BDI, migraine, CCC and Fukuda criteria, PBPI and IBS.

A

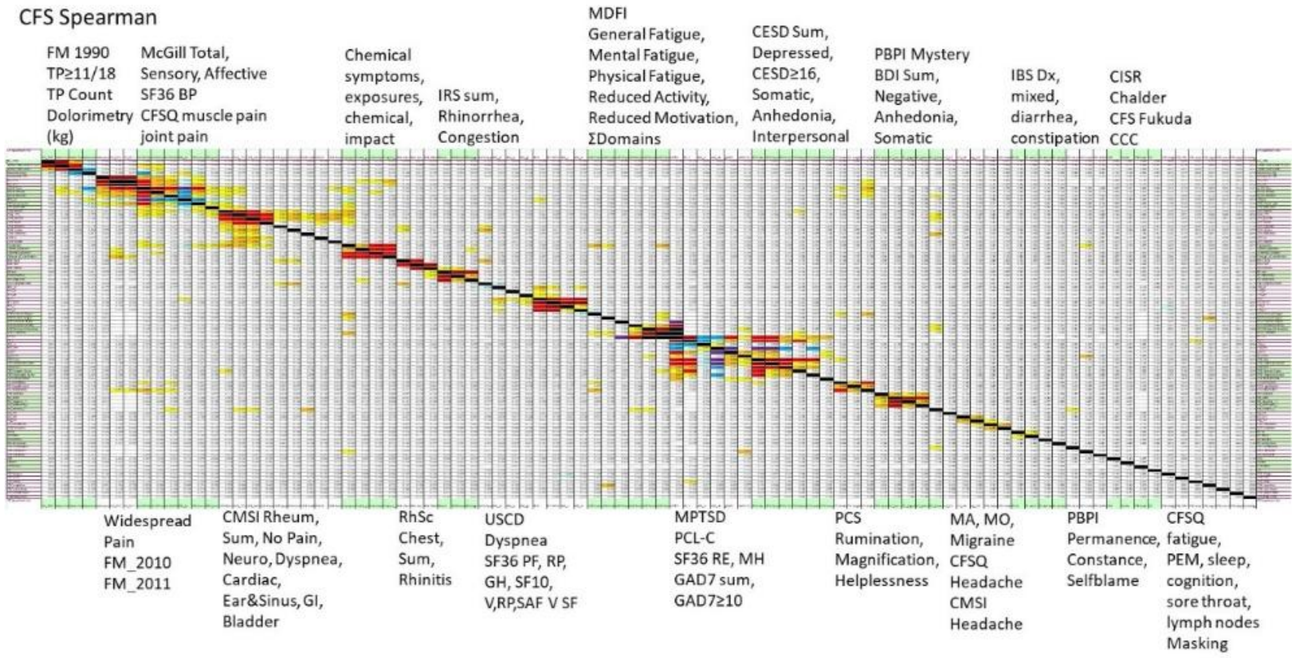

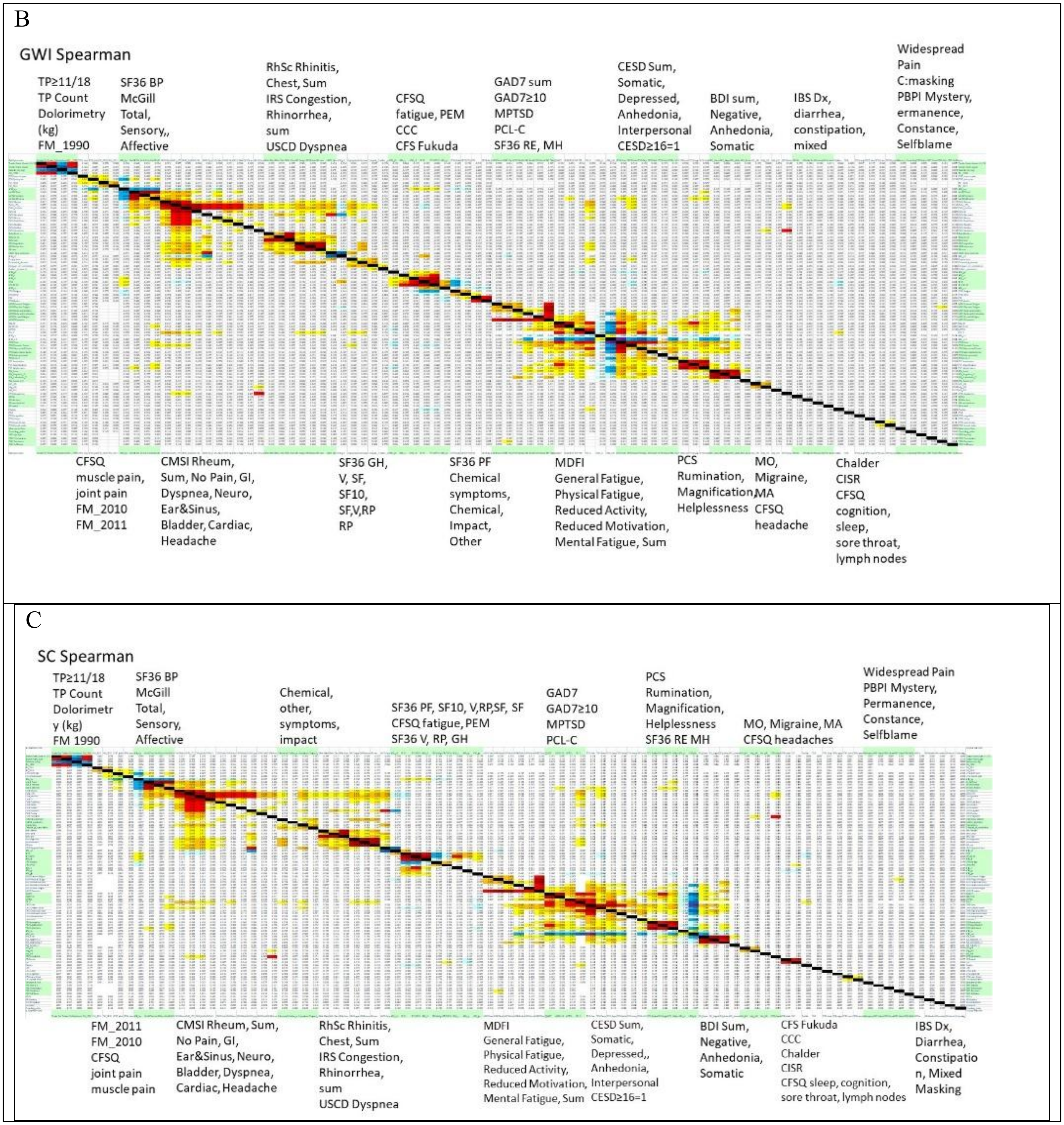
B
GWI Spearman
TP≥11/18
TP Count
Dolorimetry
(kg)
FM_1990
SF36 BP
McGill
Total,
Sensory,,
Affective
RhSc Rhinitis,
Chest, Sum
IRS Congestion,
Rhinorrhea,
sum
USCD Dyspnea
CFSQ
fatigue, PEM
CCC
CFS Fukuda
GAD7 sum
GAD7≥10
MPTSD
PCL-C
SF36 RE, MH
CESD Sum,
Somatic,
Depressed,
Anhedonia,
Interpersonal
CESD≥16=1
BDI sum,
Negative,
Anhedonia,
Somatic
IBS Dx,
diarrhea,
constipation,
mixed
Widespread
Pain
C:masking
PBPI Mystery,
ermanence,
Constance,
Selfblame
CFSQ
muscle pain,
joint pain
FM_2010
FM_2011
CMSI Rheum,
Sum, No Pain, GI,
Dyspnea, Neuro,
Ear&Sinus,
Bladder, Cardiac,
Headache
SF36 GH,
V, SF,
SF10,
SF,V,RP
RP
SF36 PF
Chemical
symptoms,
Chemical,
Impact,
Other
MDFI
General Fatigue,
Physical Fatigue,
Reduced Activity,
Reduced Motivation,
Mental Fatigue, Sum
PCS
Rumination,
Magnification,
Helplessness
MO,
Migraine,
MA
CFSQ
headache
Chalder
CISR
CFSQ
cognition,
sleep,
sore throat,
lymph nodes
C
SC Spearman
TP≥11/18
TP Count
Dolorimetr
y (kg)
FM 1990
SF36 BP
McGill
Total,
Sensory,
Affective
Chemical,
other,
symptoms,
impact
SF36 PF, SF10, V,RP,SF, SF
CFSQ fatigue, PEM
SF36 V, RP, GH
GAD7
GAD7≥10
MPTSD
PCL-C
PCS
Rumination,
Magnification,
Helplessness
SF36 RE MH
MO, Migraine, MA
CFSQ headaches
Widespread Pain
PBPI Mystery,
Permanence,
Constance,
Selfblame
FM_2011
FM_2010
CFSQ
joint pain
muscle pain
CMSI Rheum, Sum,
No Pain, GI,
Ear&Sinus, Neuro,
Bladder, Dyspnea,
Cardiac, Headache
RhSc Rhinitis,
Chest, Sum
IRS Congestion,
Rhinorrhea,
sum
USCD Dyspnea
MDFI
General Fatigue,
Physical Fatigue,
Reduced Activity,
Reduced Motivation,
Mental Fatigue, Sum
CESD Sum,
Somatic,
Depressed,,
Anhedonia,
Interpersonal
CESD≥16=1
BDI Sum,
Negative,
Anhedonia,
Somatic
CFS Fukuda
CCC
Chalder
CISR
CFSQ sleep, cognition,
sore throat, lymph nodes
IBS Dx,
Diarrhea,
Constipatio
n, Mixed
Masking

CIF was defined by quadrant analysis. Values were compared for SC vs CIF vs GWI males by ANOVA with Tukey and Bonferroni corrections. There were too few male ME/CFS and CFSLWIFS subjects for ANOVA.

SOM Table S12. Comparisons of male SC vs CIF vs GWI when selected by the quadrant method.

Descriptives	SC Male mean	SC Male SD (n=11 to 30)	CIF Male mean	CIF Male SD (n=10 to 25)	GWI Male mean	GWI Male SD (n=37 to 110)	SC CIF Bonf	GWI CIF Bonf	GWI SC Bonf
Adult_Affective	2.2	3.3	5.3	4.6	4.4	3.6			
Adult_Confidant	4.6	5.7	9.6	7.4	7.9	5.5			
Age	45.0	14.1	47.2	11.7	46.1	7.9			
ASRS∑A	8.0	3.8	7.2	4.4	13.7	4.8		0.029	
ASRS∑B	14.2	4.8	12.8	7.0	23.4	7.9		0.019	
BDI_Anhedonia	2.5	3.4	7.8	3.5	9.9	3.7			1.1E-06
BDI_Negative	1.9	3.4	7.9	8.3	8.0	5.9			
BDI_Somatic	0.8	1.1	2.7	2.2	3.3	1.9			0.0055
BDI_Sum	5.3	7.5	18.3	12.0	21.2	9.8			0.00012
Big 5 Agreeableness	26.4	4.5	27.3	4.6	23.8	5.9			
Big 5 Conscientiousness	25.1	5.1	23.4	5.2	22.2	7.3			
Big 5 Extraversion	15.0	6.2	13.6	8.4	12.6	5.8			
Big 5 Neuroticism	11.7	6.6	12.6	7.3	18.4	7.0			0.040
Big 5 Openness	26.5	5.5	27.8	8.0	23.3	9.0			
BMI	28.9	4.7	29.3	6.3	30.4	5.0			
bp Chance_Happenings	10.1	4.4	12.7	3.3	12.2	3.7			
bp Internal_Score	16.4	5.0	11.9	3.3	11.2	4.6			0.029
bp Powerful_Doctors	10.3	4.3	12.3	3.4	14.6	3.7			0.039
c_Helplessness	2.4	3.3	5.5	5.7	11.2	6.4		0.0096	8.9E-07
c_Magnification	1.2	2.1	2.8	3.0	5.4	3.3			1.5E-06
c_Rumination	2.4	3.7	4.4	4.4	8.5	4.7		0.043	1.7E-06
CESD_Anh	2.6	2.4	4.8	3.1	6.5	3.1			2.6E-06
CESD_Dep	1.7	2.2	4.0	3.4	7.2	5.0			5.2E-06
CESD_Inter	0.3	0.6	0.7	1.1	1.9	1.7			0.00021
CESD_Som	3.0	2.9	8.4	3.7	12.9	3.6	3.3E-05	1.9E-05	8.8E-07
CESD∑60_	7.4	6.9	17.9	9.1	28.6	11.0		0.0023	8.8E-07
cfsq_exertion	0.3	0.7	2.5	1.1	3.7	0.6	8.8E-07	8.8E-07	8.8E-07
cfsq_fatigue	1.0	0.8	3.4	0.5	3.7	0.5	8.8E-07		8.8E-07
cfsq_headaches	0.9	1.2	1.7	1.1	3.0	1.0		9.1E-05	8.8E-07
cfsq_joint_pain	0.7	0.9	1.7	1.4	3.3	0.9		8.9E-07	8.8E-07
cfsq_LN	0.1	0.3	0.5	0.7	1.6	1.3		0.0096	1.0E-06
cfsq_memory	0.7	0.8	2.4	0.8	3.2	0.7	8.8E-07	0.0025	8.8E-07
cfsq_muscle_pain	0.6	0.9	1.8	1.5	3.4	0.6	0.00011	8.8E-07	8.8E-07
cfsq_sleep	1.3	1.3	2.9	1.0	3.7	0.5	8.9E-07	0.0088	8.8E-07
cfsq_sore_throat	0.1	0.3	0.5	0.8	1.7	1.2		0.00065	8.9E-07
cfsq_SUM8	4.7	4.2	14.0	3.0	23.5	3.7	8.8E-07	8.8E-07	8.8E-07
Chalder Fatigue	12.2	5.4	20.9	5.1	25.1	5.1	4.6E-06		8.8E-07
Chemical chemical_domain	13.8	18.9	22.5	18.6	42.3	25.2			1.3E-05

Chemical impact_of_sensitivities	6.2	13.7	15.0	15.4	36.6	27.2		0.047	3.9E-06
Chemical masking_index	3.8	1.7	3.9	2.1	3.7	1.6			
Chemical other_exposures	7.4	12.9	16.8	11.5	21.6	17.1			0.013
Chemical symptoms	10.8	17.8	30.3	17.0	54.2	21.7		0.00039	8.8E-07
Child_Affective	2.8	3.7	5.5	4.3	3.2	3.8			
Child_Confidant	6.6	6.6	10.3	7.5	6.9	6.6			
CISR	1.6	2.1	5.5	1.2	5.8	0.7	8.8E-07		8.8E-07
CmBladder	1.8	1.9	2.6	3.3	5.8	4.0			0.00023
CmCardiac	0.4	1.2	1.5	2.1	4.1	3.5			2.7E-05
CmEarSinus	1.4	2.3	4.7	2.8	7.6	4.7			9.0E-07
CmGIRome	2.1	5.3	6.2	5.4	17.3	8.5		1.2E-06	8.8E-07
CmHA	1.0	1.9	2.0	2.2	4.3	2.6		0.034	2.4E-06
CmNeuro	1.7	3.1	5.5	2.8	8.9	3.4	0.013	0.0043	8.8E-07
CmRheum	4.6	7.0	12.8	6.1	22.4	7.4	0.023	9.5E-06	8.8E-07
CmSOB	1.5	2.4	3.7	4.7	9.1	5.8		0.0024	9.1E-07
CmSum	15.0	20.1	39.8	20.0	80.6	29.8		1.0E-06	8.8E-07
COMPASS 0-100Σ	19.7	19.2	26.1	8.7	42.2	15.5			0.020
COMPASS Bladder	1.8	2.7	1.1	1.8	2.2	2.1			
COMPASS GI	4.6	4.8	7.0	3.4	12.2	4.2			0.00066
COMPASS OI	8.7	9.0	13.3	7.9	18.3	10.3			
COMPASS Pupillomotor	1.2	1.2	1.8	0.6	2.9	1.1			0.0021
COMPASS Secromotor	3.4	3.6	2.3	3.0	6.0	3.7			
COMPASS Vasomotor	0.1	0.2	0.5	0.9	0.6	1.0			
CPSSsum	31.5	16.9	55.7	15.6	70.4	17.5			8.8E-07
Dolorimetry kg	7.0	1.8	5.3	1.5	3.8	2.2			8.8E-07
Epworth∑	8.8	4.6	7.4	3.0	12.2	6.7			
F General Fatigue	8.6	3.8	17.1	1.3	18.5	2.4	8.8E-07		8.8E-07
F Mental Fatigue	9.0	4.0	13.8	2.4	15.7	3.4			9.6E-07
F MFIΣDomains	38.4	15.1	72.9	6.4	81.3	12.6	9.0E-07		8.8E-07
F Physical Fatigue	6.8	3.6	13.7	2.7	16.6	3.0	1.2E-05		8.8E-07
F Reduced Activity	6.6	3.1	14.6	1.8	16.5	3.8	6.2E-06		8.8E-07
F Reduced Motivation	7.4	3.1	13.8	3.5	14.1	3.8	0.0027		1.3E-06
GAD7	3.3	4.9	4.5	4.9	10.6	6.0		0.0035	4.2E-06
IBS_sum	2.8	3.7	9.1	6.1	13.8	7.1			9.9E-06
IRS Cong	3.8	4.4	6.1	5.3	12.7	7.4		0.017	1.4E-05
IRS Rhino	2.6	3.6	5.5	4.0	11.2	7.7			2.9E-05
IRS Sum	6.4	7.2	11.7	8.3	23.9	14.7		0.026	7.3E-06
MASQ∑Anhedonic Depr	28.0	10.0	30.6	8.4	37.2	7.9			
MASQ∑Anxious Arousal	15.0	4.3	17.1	4.1	26.0	6.1		0.0037	5.3E-05
MASQ∑General Distress	15.3	5.9	15.8	4.8	26.6	8.4		0.020	0.0099
McG_aching	0.6	0.7	1.5	1.2	2.5	0.7	0.041	8.3E-05	8.8E-07
McG_cramping	0.2	0.6	0.6	1.0	1.7	1.0		0.0039	9.6E-07
McG_fearful	0.1	0.3	0.2	0.4	1.0	1.1		0.036	0.00071
McG_gnawing	0.0	0.2	0.5	1.0	1.2	1.0			1.1E-05
McG_heavy	0.1	0.4	0.5	0.9	1.5	1.1		0.018	1.4E-06
McG_hot_burning	0.1	0.5	0.5	0.8	1.5	1.1		0.018	2.3E-06

McG_punishing	0.0	0.2	0.1	0.4	1.1	1.2		0.0017	0.00018
McG_sharp	0.4	0.7	0.8	1.0	2.0	0.9		5.8E-06	8.8E-07
McG_shooting	0.3	0.7	0.7	1.0	1.8	1.0		0.00098	9.0E-07
McG_sickening	0.1	0.3	0.3	0.8	1.5	1.1		4.7E-05	9.2E-07
McG_splitting	0.2	0.5	0.3	0.8	1.3	1.1		0.0045	5.5E-05
McG_stabbing	0.3	0.8	0.6	0.8	1.7	1.0		0.00043	9.3E-07
McG_tender	0.2	0.6	0.4	0.8	1.6	0.9		4.5E-06	8.8E-07
McG_throbbing	0.4	0.7	0.8	0.9	1.8	0.9		0.00020	8.8E-07
McG_tiring	0.3	0.6	1.5	1.2	2.6	0.7	3.0E-05	4.5E-06	8.8E-07
McGill Affective	0.4	1.1	2.1	2.0	6.3	3.0		8.9E-07	8.8E-07
McGill Sensory	2.9	5.0	7.3	5.6	18.5	6.2		8.8E-07	8.8E-07
McGill Total	3.4	6.0	9.4	6.9	24.8	8.3		8.8E-07	8.8E-07
MPTSD∑	69.2	14.7	73.7	16.8	100.0	24.3		0.0032	1.3E-05
mTBI∑	10.6	15.1	13.7	6.2	33.8	14.4		0.014	0.0011
NPI∑	12.6	8.0	10.0	6.0	8.6	4.3			
Pain&Tenderness∑	6.9	2.8	9.8	2.5	10.6	1.8			0.0019
pbpi Constance	-1.1	1.3	1.5	0.4	1.2	0.9	9.1E-07		8.8E-07
pbpi Mystery	-1.3	1.2	0.9	0.8	0.8	0.9	2.0E-05		8.8E-07
pbpi Permanance	-0.7	1.2	1.1	0.6	0.7	1.1	0.019		0.0081
pbpi Self_blame	-1.1	1.2	-1.6	0.6	-1.3	1.2			
PCL-C∑	27.4	12.3	23.3	4.9	52.7	16.5		2.7E-05	0.00073
pm Difficulty	0.27	0.53	1.19	1.03	2.09	0.97			
RhSc chest	1.3	2.9	4.7	6.7	11.4	9.6			4.7E-05
RhSc rhinitis	4.7	5.2	10.1	7.0	17.3	10.1			1.3E-06
RhSc sum	6.0	6.8	14.8	11.4	28.7	18.6			1.3E-06
Rumination∑	33.6	9.6	30.8	4.4	48.9	16.0			
SF10	79.2	22.0	29.7	17.2	15.9	14.9	8.8E-07		8.8E-07
SF36 bp	87.6	17.8	56.1	26.6	26.7	18.6	1.8E-05	1.3E-06	8.8E-07
SF36 gh	72.2	18.0	35.2	16.4	21.5	16.0	8.8E-07		8.8E-07
SF36 mh	77.3	14.1	62.6	18.5	49.9	22.4			1.6E-06
SF36 pf	85.8	24.2	70.4	23.4	43.6	23.5		0.00060	8.8E-07
SF36 re	86.7	29.8	58.7	44.4	32.3	41.6			1.4E-06
SF36 rp	85.8	31.3	25.0	26.0	7.8	21.9	8.8E-07		8.8E-07
SF36 sf	87.1	20.9	41.5	24.4	25.5	21.8	8.8E-07		8.8E-07
SF36 v	62.2	22.5	17.8	13.6	12.9	14.3	8.8E-07		8.8E-07
Sinus kg ave	3.0	1.1	1.8	0.7	1.8	1.0			0.00054
STAI	31.6	11.8	48.2	15.1	53.2	13.8			3.7E-05
TBS∑	11.5	10.2	21.3	17.7	14.6	17.0			
Tender point count	2.0	3.3	3.7	4.6	10.3	6.6		0.0031	1.0E-06
TIQ_How_Much	18.8	7.3	22.3	7.4	30.5	10.1			4.8E-05
TIQ_How_Often	17.5	6.8	20.2	6.3	29.5	10.1		0.014	1.1E-05
TIQ∑	36.3	13.8	42.5	13.5	60.0	20.0		0.040	1.7E-05
TOY	19.5	5.7	26.9	5.7	32.5	7.0			1.1E-06
Urine∑	5.8	6.8	9.2	8.8	14.1	8.5			0.0017
USCD_Sum	4.2	4.0	10.3	11.7	37.0	31.0			0.013
Worry∑	33.8	9.9	33.7	9.3	47.1	14.6			

Bladder activities	0.0%	0.0%	0.0%	0.0%	14.6%	35.8%			
CCC	15.4%	37.6%	75.0%	45.2%	90.2%	30.0%			
CESD≥16	13.8%	35.1%	56.0%	50.7%	86.1%	34.7%			
CFS 1994 Fukuda	23.1%	43.9%	75.0%	45.2%	87.8%	33.1%			
CMI & KANSAS	3.3%	18.3%	72.0%	45.8%	95.1%	21.6%			
CMI_Dx	6.7%	25.4%	84.0%	37.4%	98.1%	13.9%			
CMI_Kanasas (short Q)	0.0%	0.0%	48.0%	51.0%	96.1%	19.4%			
DM_II	3.3%	18.3%	8.0%	27.7%	12.3%	33.0%			
FM_1990	0.0%	0.0%	12.0%	33.2%	45.4%	50.0%			
FM_2010	8.3%	28.9%	16.7%	38.9%	85.4%	35.8%			
FM_2011	0.0%	0.0%	8.3%	28.9%	78.0%	41.9%			
FM_WSP	7.1%	26.2%	52.2%	51.1%	79.8%	40.4%			
IBS	12.5%	33.8%	26.7%	45.8%	70.9%	45.8%			
IBS_constipation	0.0%	0.0%	0.0%	0.0%	14.8%	35.9%			
IBS_diarrhea	4.2%	20.4%	13.3%	35.2%	20.4%	40.7%			
IBS_Funct_Diarrhea	0.0%	0.0%	0.0%	0.0%	5.0%	22.1%			
IBS_mixed	8.3%	28.2%	13.3%	35.2%	33.3%	47.6%			
IBS_untyped	0.0%	0.0%	8.3%	28.9%	0.0%	0.0%			
IHS MA	6.9%	25.8%	4.8%	21.8%	26.0%	44.2%			
IHS MidFacial Pain	0.0%	0.0%	0.0%	0.0%	14.6%	35.8%			
IHS Migraine	10.3%	31.0%	23.8%	43.6%	63.0%	48.6%			
IHS MO	6.9%	25.8%	19.0%	40.2%	37.0%	48.6%			
IHS Tension	24.1%	43.5%	42.9%	50.7%	52.1%	50.3%			
MCS	7.7%	27.7%	0.0%	0.0%	31.0%	46.8%			
ME_Intl	7.7%	27.7%	33.3%	49.2%	43.9%	50.2%			
Military	33.3%	47.9%	60.9%	49.9%	94.2%	23.5%			
pm Major Depressive	3.8%	19.6%	23.8%	43.6%	48.3%	50.3%			
pm Other Depress	3.8%	19.6%	19.0%	40.2%	15.7%	36.6%			
pm Panic Syndrome	0.0%	0.0%	0.0%	0.0%	0.0%	0.0%			
PTSD	15.4%	37.6%	0.0%	0.0%	52.5%	50.6%			
RomeI	23.1%	43.9%	16.7%	38.9%	68.3%	47.1%			
RomeII	23.1%	43.9%	16.7%	38.9%	61.0%	49.4%			
RomeIII	23.1%	43.9%	16.7%	38.9%	58.5%	49.9%			
Sinus headache	0.0%	0.0%	6.7%	25.8%	13.3%	34.3%			
Thumb ≥11/18	6.7%	25.4%	8.7%	28.8%	55.5%	49.9%			

SOM Table S13. Comparisons between female SC, CIF, CFS and GWI segregated by the quadrant method.

Variable	Group	N	Mean	SD	SEM	95% CI	Upper	High>	>lo	Bonferonni
∑GAD7	CFS	37	6.19	5.95	0.98	4.20	8.17			
∑GAD7	CIF	16	3.13	3.18	0.80	1.43	4.82			
∑GAD7	GWI	39	8.31	5.72	0.92	6.45	10.16			
∑GAD7	SC	16	2.75	5.64	1.41	-0.25	5.75			
∑Urinary_Act	CFS	42	11.88	7.45	1.15	9.56	14.20			
∑Urinary_Act	CIF	19	6.26	5.19	1.19	3.76	8.77			
∑Urinary_Act	GWI	39	13.85	8.85	1.42	10.98	16.72			
∑Urinary_Act	SC	19	4.84	3.78	0.87	3.02	6.66			
∑Urinary_Act								GWI	SC	0.011
C:chemical_domain	CFS	37	44.00	23.48	3.86	36.17	51.83			
C:chemical_domain	CIF	15	24.47	26.47	6.83	9.81	39.13			
C:chemical_domain	GWI	38	52.63	24.78	4.02	44.49	60.78			
C:chemical_domain	SC	16	10.50	12.10	3.02	4.05	16.95			
C:chemical_domain								CFS	SC	0.0025
C:chemical_domain								GWI	SC	1.0E-05
C:impact_of_sensitivities	CFS	37	35.32	28.36	4.66	25.87	44.78			
C:impact_of_sensitivities	CIF	15	19.27	25.23	6.51	5.29	33.24			
C:impact_of_sensitivities	GWI	38	46.63	30.72	4.98	36.53	56.73			
C:impact_of_sensitivities	SC	16	3.75	5.80	1.45	0.66	6.84			
C:impact_of_sensitivities								GWI	SC	0.00028
C:masking_index	CFS	37	3.32	1.56	0.26	2.80	3.85			
C:masking_index	CIF	15	3.40	1.76	0.46	2.42	4.38			
C:masking_index	GWI	38	4.00	1.47	0.24	3.52	4.48			
C:masking_index	SC	16	3.56	1.36	0.34	2.84	4.29			
C:other_exposures	CFS	37	27.35	16.96	2.79	21.70	33.01			
C:other_exposures	CIF	15	18.53	15.11	3.90	10.17	26.90			
C:other_exposures	GWI	38	32.61	18.08	2.93	26.66	38.55			
C:other_exposures	SC	16	6.38	6.43	1.61	2.95	9.80			
C:other_exposures								CFS	SC	0.016
C:other_exposures								GWI	SC	0.00017
C:symptoms	CFS	37	43.57	22.13	3.64	36.19	50.95			
C:symptoms	CIF	15	24.93	21.41	5.53	13.08	36.79			
C:symptoms	GWI	38	52.45	27.49	4.46	43.41	61.48			
C:symptoms	SC	16	10.75	12.64	3.16	4.02	17.48			
C:symptoms								CFS	SC	0.0036
C:symptoms								GWI	SC	1.29E-05
CESD Anhesonia Factor	CFS	43	4.60	3.02	0.46	3.67	5.54			
CESD Anhesonia Factor	CIF	20	3.20	2.71	0.61	1.93	4.47			
CESD Anhesonia Factor	GWI	40	5.33	3.55	0.56	4.19	6.46			
CESD Anhesonia Factor	SC	19	3.79	3.66	0.84	2.03	5.55			
CESD Depressed Factor	CFS	43	5.53	5.06	0.77	3.98	7.09			
CESD Depressed Factor	CIF	20	2.75	3.08	0.69	1.31	4.19			
CESD Depressed Factor	GWI	40	6.33	4.47	0.71	4.89	7.76			
CESD Depressed Factor	SC	19	2.53	4.19	0.96	0.50	4.55			
CESD Interpersonal	CFS	43	0.70	1.37	0.21	0.28	1.12			
CESD Interpersonal	CIF	20	0.75	1.21	0.27	0.18	1.32			
CESD Interpersonal	GWI	40	1.05	1.26	0.20	0.65	1.45			
CESD Interpersonal	SC	19	0.74	1.63	0.37	-0.05	1.52			
CESD Somatic Factor	CFS	43	10.95	4.12	0.63	9.69	12.22			
CESD Somatic Factor	CIF	20	7.10	3.18	0.71	5.61	8.59			

CESD Somatic Factor	GWI	40	11.70	3.72	0.59	10.51	12.89			
CESD Somatic Factor	SC	19	3.21	4.49	1.03	1.05	5.38			
CESD Somatic Factor								CFS	SC	3.7E-08
CESD Somatic Factor								GWI	CIF	0.020
CESD Somatic Factor								GWI	SC	1.7E-09
CESD Sum	CFS	43	21.79	10.65	1.62	18.51	25.07			
CESD Sum	CIF	20	13.80	6.92	1.55	10.56	17.04			
CESD Sum	GWI	40	24.40	11.05	1.75	20.87	27.93			
CESD Sum	SC	19	10.26	11.42	2.62	4.76	15.77			
CESD Sum								GWI	SC	0.0019
CESD≥16=1	CFS	43	0.70	0.46	0.07	0.55	0.84			
CESD≥16=1	CIF	20	0.45	0.51	0.11	0.21	0.69			
CESD≥16=1	GWI	40	0.75	0.44	0.07	0.61	0.89			
CESD≥16=1	SC	19	0.21	0.42	0.10	0.01	0.41			
CESD≥16=1								GWI	SC	0.025
CFSQ cognition	CFS	48	3.31	0.62	0.09	3.13	3.49			
CFSQ cognition	CIF	21	2.33	1.02	0.22	1.87	2.80			
CFSQ cognition	GWI	40	3.03	0.77	0.12	2.78	3.27			
CFSQ cognition	SC	19	1.00	0.94	0.22	0.55	1.45			
CFSQ cognition								CFS	CIF	0.0034
CFSQ cognition								CFS	SC	4.5E-11
CFSQ cognition								CIF	SC	0.00027
CFSQ cognition								GWI	SC	4.6E-11
CFSQ fatigue	CFS	48	3.69	0.47	0.07	3.55	3.82			
CFSQ fatigue	CIF	21	3.48	0.51	0.11	3.24	3.71			
CFSQ fatigue	GWI	40	3.73	0.51	0.08	3.56	3.89			
CFSQ fatigue	SC	19	0.68	0.82	0.19	0.29	1.08			
CFSQ fatigue								CFS	SC	4.5-11
CFSQ fatigue								CIF	SC	4.5E-11
CFSQ fatigue								GWI	SC	4.5E-11
CFSQ headaches	CFS	48	2.65	1.21	0.17	2.29	3.00			
CFSQ headaches	CIF	21	1.24	1.09	0.24	0.74	1.73			
CFSQ headaches	GWI	40	2.85	1.21	0.19	2.46	3.24			
CFSQ headaches	SC	19	0.74	1.19	0.27	0.16	1.31			
CFSQ headaches								CFS	CIF	0.0077
CFSQ headaches								CFS	SC	1.6E-05
CFSQ headaches								GWI	CIF	0.00094
CFSQ headaches								GWI	SC	1.8E-06
CFSQ joint pain	CFS	48	2.65	1.21	0.17	2.29	3.00			
CFSQ joint pain	CIF	21	1.24	1.09	0.24	0.74	1.73			
CFSQ joint pain	GWI	40	3.13	1.07	0.17	2.78	3.47			
CFSQ joint pain	SC	19	0.58	0.84	0.19	0.18	0.98			
CFSQ joint pain								CFS	CIF	0.0016
CFSQ joint pain								CFS	SC	1.09E-07
CFSQ joint pain								GWI	CIF	1.86E-06
CFSQ joint pain								GWI	SC	1.22E-10
CFSQ lymph nodes	CFS	48	1.54	1.29	0.19	1.17	1.92			
CFSQ lymph nodes	CIF	21	0.62	0.67	0.15	0.31	0.92			
CFSQ lymph nodes	GWI	40	1.50	1.34	0.21	1.07	1.93			
CFSQ lymph nodes	SC	19	0.11	0.46	0.11	-0.12	0.33			
CFSQ lymph nodes								CFS	SC	0.0045
CFSQ lymph nodes								GWI	SC	0.013
CFSQ muscle pain	CFS	48	3.19	0.94	0.14	2.92	3.46			
CFSQ muscle pain	CIF	21	1.81	1.25	0.27	1.24	2.38			

CFSQ muscle pain	GWI	40	3.25	0.84	0.13	2.98	3.52			
CFSQ muscle pain	SC	19	0.89	1.37	0.31	0.23	1.56			
CFSQ muscle pain								CFS	CIF	0.00078
CFSQ muscle pain								CFS	SC	2.4E-10
CFSQ muscle pain								GWI	CIF	0.00056
CFSQ muscle pain								GWI	SC	2.5E-10
CFSQ PEM	CFS	48	3.54	0.77	0.11	3.32	3.77			
CFSQ PEM	CIF	21	3.00	1.26	0.28	2.42	3.58			
CFSQ PEM	GWI	40	3.48	0.85	0.13	3.20	3.75			
CFSQ PEM	SC	19	0.63	1.21	0.28	0.05	1.22			
CFSQ PEM								CFS	SC	4.5E-11
CFSQ PEM								CIF	SC	1.3E-09
CFSQ PEM								GWI	SC	4.5E-11
CFSQ sleep	CFS	48	3.56	0.71	0.10	3.36	3.77			
CFSQ sleep	CIF	21	2.86	1.31	0.29	2.26	3.46			
CFSQ sleep	GWI	40	3.60	0.59	0.09	3.41	3.79			
CFSQ sleep	SC	19	1.11	1.56	0.36	0.35	1.86			
CFSQ sleep								CFS	SC	4.5E-11
CFSQ sleep								CIF	SC	4.1E-05
CFSQ sleep								GWI	SC	4.5E-11
CFSQ sore throat	CFS	48	1.44	1.22	0.18	1.08	1.79			
CFSQ sore throat	CIF	21	0.90	0.83	0.18	0.53	1.28			
CFSQ sore throat	GWI	40	1.73	1.04	0.16	1.39	2.06			
CFSQ sore throat	SC	19	0.32	0.75	0.17	-0.05	0.68			
CFSQ sore throat								GWI	SC	0.0021
Chalder	CFS	43	23.93	6.30	0.96	21.99	25.87			
Chalder	CIF	20	18.65	7.33	1.64	15.22	22.08			
Chalder	GWI	39	25.31	4.43	0.71	23.87	26.74			
Chalder	SC	19	12.84	5.78	1.33	10.06	15.63			
Chalder								CFS	SC	1.97E-07
Chalder								GWI	CIF	0.038
Chalder								GWI	SC	4.8E-09
CISR	CFS	42	5.76	0.53	0.08	5.60	5.93			
CISR	CIF	20	5.10	1.59	0.35	4.36	5.84			
CISR	GWI	39	5.72	0.72	0.12	5.48	5.95			
CISR	SC	19	1.21	2.27	0.52	0.11	2.31			
CISR								CFS	SC	4.1E-11
CISR								CIF	SC	4.1E-11
CISR								GWI	SC	4.1E-11
CMSI Bladder	CFS	41	4.15	4.28	0.67	2.80	5.50			
CMSI Bladder	CIF	20	2.25	2.95	0.66	0.87	3.63			
CMSI Bladder	GWI	39	5.65	4.28	0.68	4.27	7.04			
CMSI Bladder	SC	19	1.05	1.96	0.45	0.11	2.00			
CMSI Bladder								GWI	SC	0.017
CMSI Cardiac	CFS	41	3.17	3.11	0.49	2.19	4.15			
CMSI Cardiac	CIF	20	2.05	2.50	0.56	0.88	3.22			
CMSI Cardiac	GWI	39	4.51	4.03	0.64	3.21	5.82			
CMSI Cardiac	SC	19	0.58	1.50	0.34	-0.15	1.30			
CMSI Cardiac								GWI	SC	0.012
CMSI Dyspnea	CFS	41	7.41	5.82	0.91	5.58	9.25			
CMSI Dyspnea	CIF	20	4.60	4.45	1.00	2.52	6.68			
CMSI Dyspnea	GWI	39	8.72	5.41	0.87	6.96	10.47			
CMSI Dyspnea	SC	19	1.74	2.33	0.53	0.61	2.86			
CMSI Dyspnea								GWI	SC	0.0015

CMSI Ear&Sinus	CFS	41	4.02	3.59	0.56	2.89	5.16		
CMSI Ear&Sinus	CIF	20	3.65	4.17	0.93	1.70	5.60		
CMSI Ear&Sinus	GWI	39	7.64	4.89	0.78	6.06	9.22		
CMSI Ear&Sinus	SC	19	0.84	1.64	0.38	0.05	1.63		
CMSI Ear&Sinus								GWI	CFS	0.044
CMSI Ear&Sinus								GWI	SC	6.7E-06
CMSI GI	CFS	41	8.37	7.35	1.15	6.04	10.69			
CMSI GI	CIF	20	7.55	8.19	1.83	3.72	11.38			
CMSI GI	GWI	39	16.37	7.94	1.27	13.80	18.95			
CMSI GI	SC	19	3.05	5.24	1.20	0.53	5.58			
CMSI GI								GWI	CFS	0.0024
CMSI GI								GWI	CIF	0.018
CMSI GI								GWI	SC	1.8E-06
CMSI Headache	CFS	41	2.76	2.41	0.38	2.00	3.52			
CMSI Headache	CIF	20	1.15	1.50	0.33	0.45	1.85			
CMSI Headache	GWI	39	4.59	2.54	0.41	3.77	5.41			
CMSI Headache	SC	19	1.00	1.86	0.43	0.11	1.89			
CMSI Headache								GWI	CIF	9.8E-05
CMSI Headache								GWI	SC	5.1E-05
CMSI Neuro	CFS	41	7.46	3.87	0.60	6.24	8.69			
CMSI Neuro	CIF	20	4.85	3.08	0.69	3.41	6.29			
CMSI Neuro	GWI	39	9.63	3.72	0.60	8.42	10.83			
CMSI Neuro	SC	19	1.47	2.50	0.57	0.27	2.68			
CMSI Neuro								CFS	SC	6.6E-06
CMSI Neuro								GWI	CIF	0.0014
CMSI Neuro								GWI	SC	1.69E-10
CMSI Rheum	CFS	41	19.34	7.65	1.19	16.93	21.76			
CMSI Rheum	CIF	20	12.20	6.17	1.38	9.31	15.09			
CMSI Rheum	GWI	39	23.15	8.36	1.34	20.44	25.86			
CMSI Rheum	SC	19	5.26	6.32	1.45	2.22	8.31			
CMSI Rheum								CFS	SC	3.06E-07
CMSI Rheum								GWI	CIF	0.00028
CMSI Rheum								GWI	SC	7.7E-11
CMSI_172_	CFS	41	57.20	26.29	4.11	48.90	65.49			
CMSI_172_	CIF	20	38.30	21.16	4.73	28.40	48.20			
CMSI_172_	GWI	39	80.27	33.21	5.32	69.50	91.04			
CMSI_172_	SC	19	15.00	18.79	4.31	5.94	24.06			
CMSI_172_								CFS	SC	7.8E-05
CMSI_172_								GWI	CIF	7.1E-05
CMSI_172_								GWI	SC	6.7E-11
CMSI_NoPain	CFS	41	37.85	20.12	3.14	31.50	44.21			
CMSI_NoPain	CIF	20	26.10	17.60	3.94	17.86	34.34			
CMSI_NoPain	GWI	39	57.12	26.37	4.22	48.57	65.66			
CMSI_NoPain	SC	19	9.74	12.89	2.96	3.53	15.95			
CMSI_NoPain								CFS	SC	0.0028
CMSI_NoPain								GWI	CFS	0.046
CMSI_NoPain								GWI	CIF	0.00027
CMSI_NoPain								GWI	SC	6.4E-10
COMPASS Bladder	CFS	17	1.36	1.53	0.37	0.57	2.15			
COMPASS Bladder	CIF	11	0.90	1.08	0.33	0.17	1.63			
COMPASS Bladder	GWI	9	2.58	2.15	0.72	0.92	4.23			
COMPASS Bladder	SC	10	0.00	0.00	0.00	0.00	0.00			
COMPASS GI	CFS	17	8.34	4.99	1.21	5.78	10.91			
COMPASS GI	CIF	11	8.28	3.56	1.07	5.89	10.68			

COMPASS GI	GWI	9	12.01	3.97	1.32	8.96	15.07			
COMPASS GI	SC	10	4.03	4.61	1.46	0.73	7.33			
COMPASS OI	CFS	17	18.59	7.74	1.88	14.61	22.57			
COMPASS OI	CIF	11	13.45	7.43	2.24	8.46	18.45			
COMPASS OI	GWI	9	17.33	10.00	3.33	9.65	25.02			
COMPASS OI	SC	10	8.80	12.04	3.81	0.18	17.42			
COMPASS Pupil	CFS	17	2.65	0.74	0.18	2.27	3.03			
COMPASS Pupil	CIF	11	2.27	0.87	0.26	1.69	2.86			
COMPASS Pupil	GWI	9	2.84	1.10	0.37	2.00	3.69			
COMPASS Pupil	SC	10	0.98	0.51	0.16	0.62	1.34			
COMPASS Pupil								CFS	SC	0.0031
COMPASS Pupil								GWI	SC	0.0054
COMPASS Secretomotor	CFS	17	4.04	3.86	0.94	2.05	6.02			
COMPASS Secretomotor	CIF	11	4.27	3.45	1.04	1.96	6.59			
COMPASS Secretomotor	GWI	9	7.39	4.03	1.34	4.29	10.49			
COMPASS Secretomotor	SC	10	0.64	1.45	0.46	-0.39	1.67			
COMPASS Sum	CFS	17	35.68	12.72	3.08	29.14	42.22			
COMPASS Sum	CIF	11	29.96	11.56	3.48	22.20	37.73			
COMPASS Sum	GWI	9	43.18	15.93	5.31	30.94	55.42			
COMPASS Sum	SC	10	14.44	13.22	4.18	4.98	23.90			
COMPASS Sum								GWI	SC	0.013
COMPASS Vasomotor	CFS	17	0.69	1.00	0.24	0.18	1.21			
COMPASS Vasomotor	CIF	11	0.76	1.15	0.35	-0.01	1.53			
COMPASS Vasomotor	GWI	9	1.02	1.30	0.43	0.02	2.02			
COMPASS Vasomotor	SC	10	0.00	0.00	0.00	0.00	0.00			
Depr_DSMIV	CFS	4	0.25	0.50	0.25	-0.55	1.05			
Depr_DSMIV	CIF	3	0.00	0.00	0.00	0.00	0.00			
Depr_DSMIV	GWI	3	0.00	0.00	0.00	0.00	0.00			
Depr_DSMIV	SC	1	0.00							
Dolorimetry (kg)	CFS	50	2.78	1.40	0.20	2.38	3.18			
Dolorimetry (kg)	CIF	21	3.71	1.47	0.32	3.04	4.38			
Dolorimetry (kg)	GWI	46	2.33	1.33	0.20	1.93	2.72			
Dolorimetry (kg)	SC	20	4.43	1.74	0.39	3.62	5.24			
Dolorimetry (kg)								SC	CFS	0.015
Dolorimetry (kg)								SC	GWI	0.00012
Epworth	CFS	17	8.76	5.29	1.28	6.05	11.48			
Epworth	CIF	11	7.64	3.11	0.94	5.55	9.72			
Epworth	GWI	9	13.89	4.31	1.44	10.57	17.20			
Epworth	SC	10	5.90	5.57	1.76	1.92	9.88			
FM_1990	CFS	50	0.50	0.51	0.07	0.36	0.64			
FM_1990	CIF	21	0.10	0.30	0.07	-0.04	0.23			
FM_1990	GWI	29	0.66	0.48	0.09	0.47	0.84			
FM_1990	SC	18	0.00	0.00	0.00	0.00	0.00			
FM_1990								CFS	SC	0.023
FM_1990								GWI	CIF	0.0068
FM_1990								GWI	SC	0.00074
FM_2010	CFS	17	0.82	0.39	0.10	0.62	1.03			
FM_2010	CIF	12	0.58	0.51	0.15	0.26	0.91			
FM_2010	GWI	8	0.88	0.35	0.13	0.58	1.17			
FM_2010	SC	10	0.00	0.00	0.00	0.00	0.00			
FM_2010								CFS	SC	0.0014
FM_2010								GWI	SC	0.0093
FM_2011	CFS	19	0.74	0.45	0.10	0.52	0.95			
FM_2011	CIF	11	0.55	0.52	0.16	0.19	0.90			

FM_2011	GWI	8	0.63	0.52	0.18	0.19	1.06			
FM_2011	SC	10	0.00	0.00	0.00	0.00	0.00			
FM_2011								CFS	SC	0.042
GAD_DSM5	CFS	2	0.00	0.00	0.00	0.00	0.00			
GAD_DSM5	CIF	3	0.00	0.00	0.00	0.00	0.00			
GAD_DSM5	GWI	3	0.00	0.00	0.00	0.00	0.00			
GAD_DSM5	SC	1	0.00							
GAD_ICD10	CFS	3	0.33	0.58	0.33	-1.10	1.77			
GAD_ICD10	CIF	3	0.00	0.00	0.00	0.00	0.00			
GAD_ICD10	GWI	3	0.00	0.00	0.00	0.00	0.00			
GAD_ICD10	SC	1	0.00							
GAD7≥10	CFS	37	0.27	0.45	0.07	0.12	0.42			
GAD7≥10	CIF	16	0.00	0.00	0.00	0.00	0.00			
GAD7≥10	GWI	39	0.44	0.50	0.08	0.27	0.60			
GAD7≥10	SC	16	0.13	0.34	0.09	-0.06	0.31			
IBS constipation	CFS	43	0.12	0.32	0.05	0.02	0.22			
IBS constipation	CIF	19	0.11	0.32	0.07	-0.05	0.26			
IBS constipation	GWI	21	0.05	0.22	0.05	-0.05	0.15			
IBS constipation	SC	15	0.00	0.00	0.00	0.00	0.00			
IBS diarrhea	CFS	43	0.12	0.32	0.05	0.02	0.22			
IBS diarrhea	CIF	19	0.21	0.42	0.10	0.01	0.41			
IBS diarrhea	GWI	21	0.33	0.48	0.11	0.11	0.55			
IBS diarrhea	SC	15	0.00	0.00	0.00	0.00	0.00			
IBS Dx	CFS	43	0.47	0.50	0.08	0.31	0.62			
IBS Dx	CIF	19	0.47	0.51	0.12	0.23	0.72			
IBS Dx	GWI	21	0.71	0.46	0.10	0.50	0.92			
IBS Dx	SC	15	0.07	0.26	0.07	-0.08	0.21			
IBS Dx								GWI	SC	0.049
IBS mixed	CFS	43	0.21	0.41	0.06	0.08	0.34			
IBS mixed	CIF	19	0.26	0.45	0.10	0.05	0.48			
IBS mixed	GWI	21	0.33	0.48	0.11	0.11	0.55			
IBS mixed	SC	15	0.07	0.26	0.07	-0.08	0.21			
IRS Congestion	CFS	40	9.48	6.97	1.10	7.25	11.70			
IRS Congestion	CIF	19	6.84	7.61	1.75	3.17	10.51			
IRS Congestion	GWI	39	14.36	8.35	1.34	11.65	17.06			
IRS Congestion	SC	19	3.79	4.61	1.06	1.57	6.01			
IRS Congestion								GWI	SC	0.000498
IRS Rhinorrhea	CFS	40	6.60	6.17	0.98	4.63	8.57			
IRS Rhinorrhea	CIF	19	5.26	6.07	1.39	2.34	8.19			
IRS Rhinorrhea	GWI	39	13.33	7.03	1.13	11.06	15.61			
IRS Rhinorrhea	SC	19	2.58	4.00	0.92	0.65	4.51			
IRS Rhinorrhea								GWI	CFS	0.0022
IRS Rhinorrhea								GWI	CIF	0.0046
IRS Rhinorrhea								GWI	SC	4.7E-06
IRS sum	CFS	40	16.08	12.01	1.90	12.23	19.92			
IRS sum	CIF	19	12.11	12.36	2.84	6.15	18.06			
IRS sum	GWI	39	27.69	14.31	2.29	23.06	32.33			
IRS sum	SC	19	6.37	8.15	1.87	2.44	10.30			
IRS sum								GWI	CFS	0.033
IRS sum								GWI	CIF	0.0092
IRS sum								GWI	SC	6.7E-06
MA_IHS	CFS	48	0.23	0.42	0.06	0.11	0.35			
MA_IHS	CIF	19	0.11	0.32	0.07	-0.05	0.26			
MA_IHS	GWI	25	0.20	0.41	0.08	0.03	0.37			

MA_IHS	SC	17	0.00	0.00	0.00	0.00	0.00			
MASQ Anhedonic Depression	CFS	18	31.67	8.20	1.93	27.59	35.75			
MASQ Anhedonic Depression	CIF	11	28.00	7.89	2.38	22.70	33.30			
MASQ Anhedonic Depression	GWI	9	33.00	10.01	3.34	25.30	40.70			
MASQ Anhedonic Depression	SC	10	28.10	11.64	3.68	19.77	36.43			
MASQ Anxious Arousal	CFS	18	23.33	5.44	1.28	20.63	26.04			
MASQ Anxious Arousal	CIF	11	19.45	4.20	1.27	16.63	22.28			
MASQ Anxious Arousal	GWI	9	26.56	5.43	1.81	22.38	30.73			
MASQ Anxious Arousal	SC	10	14.50	3.24	1.02	12.18	16.82			
MASQ Anxious Arousal								CFS	SC	0.015
MASQ Anxious Arousal								GWI	SC	0.0011
MASQ General Distress	CFS	18	21.00	8.46	1.99	16.79	25.21			
MASQ General Distress	CIF	11	17.18	4.81	1.45	13.95	20.42			
MASQ General Distress	GWI	9	25.78	7.21	2.40	20.24	31.32			
MASQ General Distress	SC	10	18.10	9.88	3.12	11.03	25.17			
McGill Affective	CFS	42	3.38	2.85	0.44	2.49	4.27			
McGill Affective	CIF	18	2.39	2.97	0.70	0.91	3.87			
McGill Affective	GWI	39	6.10	3.19	0.51	5.07	7.14			
McGill Affective	SC	19	0.53	1.07	0.25	0.01	1.04			
McGill Affective								GWI	CFS	
McGill Affective								GWI	CIF	0.0046
McGill Affective								GWI	SC	5.2E-08
McGill Sensory	CFS	42	13.67	6.78	1.05	11.55	15.78			
McGill Sensory	CIF	18	9.61	7.88	1.86	5.69	13.53			
McGill Sensory	GWI	39	19.62	7.19	1.15	17.29	21.95			
McGill Sensory	SC	19	4.16	6.58	1.51	0.99	7.33			
McGill Sensory								CFS	SC	0.0019
McGill Sensory								GWI	CIF	0.0013
McGill Sensory								GWI	SC	1.56E-09
McGill Total	CFS	42	17.05	8.58	1.32	14.37	19.72			
McGill Total	CIF	18	12.00	10.18	2.40	6.94	17.06			
McGill Total	GWI	39	25.72	9.95	1.59	22.49	28.94			
McGill Total	SC	19	4.68	7.54	1.73	1.05	8.32			
McGill Total								CFS	SC	0.0019
McGill Total								GWI	CFS	0.023
McGill Total								GWI	CIF	0.00038
McGill Total								GWI	SC	2.6E-10
MDFI General Fatigue	CFS	25	18.56	1.92	0.38	17.77	19.35			
MDFI General Fatigue	CIF	9	15.78	5.78	1.93	11.33	20.22			
MDFI General Fatigue	GWI	30	17.30	3.26	0.60	16.08	18.52			
MDFI General Fatigue	SC	9	8.00	4.24	1.41	4.74	11.26			
MDFI General Fatigue								CFS	SC	1.5E-08
MDFI General Fatigue								CIF	SC	0.0044
MDFI General Fatigue								GWI	SC	3.9E-07
MDFI Mental Fatigue	CFS	25	15.40	3.64	0.73	13.90	16.90			
MDFI Mental Fatigue	CIF	9	9.78	4.32	1.44	6.45	13.10			
MDFI Mental Fatigue	GWI	30	15.47	3.61	0.66	14.12	16.81			
MDFI Mental Fatigue	SC	9	8.33	4.30	1.43	5.03	11.64			
MDFI Mental Fatigue								CFS	SC	0.004956623
MDFI Mental Fatigue								GWI	SC	0.002771036
MDFI Physical Fatigue	CFS	25	16.76	3.15	0.63	15.46	18.06			
MDFI Physical Fatigue	CIF	9	14.78	6.50	2.17	9.78	19.77			
MDFI Physical Fatigue	GWI	30	15.70	3.20	0.58	14.51	16.89			
MDFI Physical Fatigue	SC	9	9.89	6.66	2.22	4.77	15.01			

MDFI Physical Fatigue								CFS	SC	0.04288508
MDFI Reduced Activity	CFS	25	17.56	2.00	0.40	16.73	18.39			
MDFI Reduced Activity	CIF	9	14.33	5.57	1.86	10.05	18.61			
MDFI Reduced Activity	GWI	30	15.97	3.51	0.64	14.66	17.28			
MDFI Reduced Activity	SC	9	7.00	4.44	1.48	3.58	10.42			
MDFI Reduced Activity								CFS	SC	4.0E-08
MDFI Reduced Activity								CIF	SC	0.020
MDFI Reduced Activity								GWI	SC	2.7E-06
MDFI Reduced Motivation	CFS	25	12.96	4.12	0.82	11.26	14.66			
MDFI Reduced Motivation	CIF	9	10.22	4.18	1.39	7.01	13.43			
MDFI Reduced Motivation	GWI	30	12.13	2.75	0.50	11.11	13.16			
MDFI Reduced Motivation	SC	9	6.56	2.40	0.80	4.71	8.40			
MDFI Reduced Motivation								CFS	SC	0.0048
MDFI Reduced Motivation								GWI	SC	0.032
MDFI ΣDomains	CFS	25	81.24	9.88	1.98	77.16	85.32			
MDFI ΣDomains	CIF	9	64.89	21.76	7.25	48.17	81.61			
MDFI ΣDomains	GWI	30	76.57	12.90	2.35	71.75	81.38			
MDFI ΣDomains	SC	9	39.78	20.12	6.71	24.31	55.25			
MDFI ΣDomains								CFS	SC	1.2E-07
MDFI ΣDomains								GWI	SC	2.1E-06
Mig_IHS	CFS	48	0.79	0.41	0.06	0.67	0.91			
Mig_IHS	CIF	19	0.47	0.51	0.12	0.23	0.72			
Mig_IHS	GWI	25	0.76	0.44	0.09	0.58	0.94			
Mig_IHS	SC	17	0.18	0.39	0.10	-0.03	0.38			
Mig_IHS								CFS	SC	0.0011
Mig_IHS								GWI	SC	0.022
MO_IHS	CFS	48	0.56	0.50	0.07	0.42	0.71			
MO_IHS	CIF	19	0.42	0.51	0.12	0.18	0.67			
MO_IHS	GWI	25	0.64	0.49	0.10	0.44	0.84			
MO_IHS	SC	17	0.18	0.39	0.10	-0.03	0.38			
MPTSD	CFS	14	69.57	11.32	3.03	63.04	76.11			
MPTSD	CIF	5	70.60	16.41	7.34	50.22	90.98			
MPTSD	GWI	31	87.84	25.41	4.56	78.52	97.16			
MPTSD	SC	9	53.78	7.85	2.62	47.74	59.82			
MPTSD								GWI	SC	0.023
Multiple Chemical Sensitivity	CFS	20	0.25	0.44	0.10	0.04	0.46			
Multiple Chemical Sensitivity	CIF	12	0.08	0.29	0.08	-0.10	0.27			
Multiple Chemical Sensitivity	GWI	9	0.56	0.53	0.18	0.15	0.96			
Multiple Chemical Sensitivity	SC	10	0.00	0.00	0.00	0.00	0.00			
PCL-C	CFS	18	34.28	12.89	3.04	27.87	40.69			
PCL-C	CIF	11	25.55	5.96	1.80	21.54	29.55			
PCL-C	GWI	9	50.22	9.58	3.19	42.86	57.58			
PCL-C	SC	10	25.90	18.23	5.76	12.86	38.94			
PCL-C								GWI	CIF	0.038
PTSD	CFS	19	0.32	0.48	0.11	0.09	0.55			
PTSD	CIF	11	0.18	0.40	0.12	-0.09	0.45			
PTSD	GWI	9	0.67	0.50	0.17	0.28	1.05			
PTSD	SC	10	0.10	0.32	0.10	-0.13	0.33			
RhSc Chest	CFS	40	5.00	6.09	0.96	3.05	6.95			
RhSc Chest	CIF	19	4.05	6.40	1.47	0.97	7.14			
RhSc Chest	GWI	39	8.64	6.65	1.06	6.49	10.80			
RhSc Chest	SC	19	0.79	1.36	0.31	0.14	1.44			
RhSc Chest								GWI	SC	0.0029
RhSc Rhinitis	CFS	40	11.50	7.45	1.18	9.12	13.88			

RhSc Rhinitis	CIF	19	9.58	8.11	1.86	5.67	13.49			
RhSc Rhinitis	GWI	39	17.23	7.99	1.28	14.64	19.82			
RhSc Rhinitis	SC	19	4.74	5.38	1.24	2.14	7.33			
RhSc Rhinitis								GWI	SC	1.47E-05
RhSc Sum	CFS	40	16.50	11.96	1.89	12.68	20.32			
RhSc Sum	CIF	19	13.63	13.06	3.00	7.33	19.93			
RhSc Sum	GWI	39	25.87	13.42	2.15	21.52	30.22			
RhSc Sum	SC	19	5.53	6.37	1.46	2.46	8.60			
RhSc Sum								GWI	SC	9.7E-06
SF V RP SF	CFS	44	15.39	14.62	2.20	10.94	19.83			
SF V RP SF	CIF	21	27.24	23.12	5.04	16.72	37.76			
SF V RP SF	GWI	39	17.85	13.31	2.13	13.53	22.16			
SF V RP SF	SC	19	74.16	27.92	6.41	60.70	87.62			
SF V RP SF								SC	CFS	4.4E-11
SF V RP SF								SC	CIF	5.1E-10
SF V RP SF								SC	GWI	4.4E-11
SF10	CFS	44	14.15	14.91	2.25	9.62	18.68			
SF10	CIF	21	25.74	24.11	5.26	14.76	36.71			
SF10	GWI	39	15.42	13.46	2.16	11.06	19.79			
SF10	SC	19	74.79	28.74	6.59	60.94	88.64			
SF10								SC	CFS	4.4E-11
SF10								SC	CIF	2.7E-10
SF10								SC	GWI	4.4E-11
sf36_bp	CFS	44	36.08	21.96	3.31	29.40	42.75			
sf36_bp	CIF	21	59.29	26.25	5.73	47.34	71.23			
sf36_bp	GWI	39	28.78	21.25	3.40	21.89	35.67			
sf36_bp	SC	19	84.21	23.50	5.39	72.89	95.54			
sf36_bp								CIF	GWI	0.00145
sf36_bp								SC	CFS	2.5E-09
sf36_bp								SC	GWI	5.6E-11
sf36_gh	CFS	44	31.82	20.63	3.11	25.55	38.09			
sf36_gh	CIF	21	43.57	20.44	4.46	34.27	52.88			
sf36_gh	GWI	39	33.85	19.45	3.11	27.54	40.15			
sf36_gh	SC	19	74.74	22.82	5.24	63.74	85.74			
sf36_gh								SC	CFS	4.3E-09
sf36_gh								SC	CIF	0.0027
sf36_gh								SC	GWI	5.4E-08
sf36_mh	CFS	44	65.27	18.15	2.74	59.75	70.79			
sf36_mh	CIF	21	70.48	13.41	2.93	64.37	76.58			
sf36_mh	GWI	39	60.41	19.74	3.16	54.01	66.81			
sf36_mh	SC	19	73.26	18.76	4.30	64.22	82.31			
sf36_pf	CFS	44	37.39	22.01	3.32	30.70	44.08			
sf36_pf	CIF	21	51.67	25.90	5.65	39.88	63.46			
sf36_pf	GWI	39	40.90	25.90	4.15	32.50	49.29			
sf36_pf	SC	19	88.16	21.23	4.87	77.93	98.39			
sf36_pf								SC	CFS	2.0E-09
sf36_pf								SC	CIF	0.0023
sf36_pf								SC	GWI	6.2E-08
sf36_re	CFS	44	61.36	44.86	6.76	47.72	75.00			
sf36_re	CIF	21	63.49	43.34	9.46	43.76	83.22			
sf36_re	GWI	39	34.19	42.22	6.76	20.50	47.88			
sf36_re	SC	19	82.46	35.78	8.21	65.21	99.70			
sf36_re								SC	GWI	0.047
sf36_rp	CFS	44	6.82	19.71	2.97	0.83	12.81			

sf36_rp	CIF	21	13.10	31.24	6.82	-1.13	27.32			
sf36_rp	GWI	39	4.49	15.03	2.41	-0.39	9.36			
sf36_rp	SC	19	73.68	45.24	10.38	51.88	95.49			
sf36_rp								SC	CFS	4.4E-11
sf36_rp								SC	CIF	1.9E-08
sf36_rp								SC	GWI	4.4E-11
sf36_sf	CFS	44	24.72	22.15	3.34	17.98	31.45			
sf36_sf	CIF	21	43.45	28.94	6.32	30.28	56.63			
sf36_sf	GWI	39	30.45	20.84	3.34	23.69	37.20			
sf36_sf	SC	19	84.21	25.97	5.96	71.69	96.73			
sf36_sf								SC	CFS	4.5E-11
sf36_sf								SC	CIF	0.00016
sf36_sf								SC	GWI	3.0E-10
sf36_v	CFS	44	14.55	13.37	2.02	10.48	18.61			
sf36_v	CIF	21	25.24	16.54	3.61	17.71	32.77			
sf36_v	GWI	39	18.46	14.01	2.24	13.92	23.00			
sf36_v	SC	19	64.47	21.53	4.94	54.10	74.85			
sf36_v								SC	CFS	4.4E-11
sf36_v								SC	CIF	7.4E-10
sf36_v								SC	GWI	4.4E-11
Tender Point Count	CFS	48	10.78	5.70	0.82	9.12	12.43			
Tender Point Count	CIF	20	7.89	4.91	1.10	5.59	10.18			
Tender Point Count	GWI	43	13.44	4.74	0.72	11.98	14.90			
Tender Point Count	SC	20	4.00	5.24	1.17	1.55	6.45			
Tender Point Count								CFS	SC	0.0017
Tender Point Count								GWI	SC	3.5E-07
Tender Point Count ≥11/18	CFS	49	0.55	0.50	0.07	0.41	0.70			
Tender Point Count ≥11/18	CIF	21	0.43	0.51	0.11	0.20	0.66			
Tender Point Count ≥11/18	GWI	45	0.73	0.45	0.07	0.60	0.87			
Tender Point Count ≥11/18	SC	20	0.15	0.37	0.08	-0.02	0.32			
Tender Point Count ≥11/18								GWI	SC	0.0045
USCD Dyspnea Score	CFS	22	34.77	27.69	5.90	22.50	47.05			
USCD Dyspnea Score	CIF	8	23.25	24.05	8.50	3.15	43.35			
USCD Dyspnea Score	GWI	30	36.27	25.06	4.57	26.91	45.62			
USCD Dyspnea Score	SC	9	7.33	10.89	3.63	-1.03	15.70			
Widespread Pain	CFS	45	0.64	0.48	0.07	0.50	0.79			
Widespread Pain	CIF	20	0.50	0.51	0.11	0.26	0.74			
Widespread Pain	GWI	32	0.88	0.34	0.06	0.75	1.00			
Widespread Pain	SC	18	0.17	0.38	0.09	-0.02	0.36			
Widespread Pain								GWI	SC	0.00014

end

SOM Table S14. Data for all subjects reclassified to SC, CIF, ME/CFS and GWI groups (quadrant analysis). Mean, SD, n, ANOVA with Tukey and Bonferroni corrections (p).

Variable	SC	Mean	Sd	n	CIF	mean	sd	n	WIFS	mean	sd	n	CFS	mean	sd	n	GWI	mean	sd	n	GWI >CFS	GWI >CIF	GWI >SC	GWI> WIFS	CFS >SC	CFS >CIF	CIF >SC	WIFS>SC	CFS> WIFS	CIF> WIFS
Age	SC	43.5	16.6	55	CIF	47.5	12.1	42	WIFS	51.1	7.5	8	CFS	47.4	10.6	62	GWI	47.6	8.4	145										
BMI	SC	28.3	4.8	54	CIF	27.6	5.9	42	WIFS	29.3	6.7	8	CFS	28.4	7.0	62	GWI	30.2	5.1	145										
CFSQ cognition	SC	0.8	0.9	49	CIF	2.3	0.9	42	WIFS	3.3	0.5	8	CFS	3.3	0.6	58	GWI	3.2	0.7	134		8.8E-06	6.8E-11		6.8E-11	1.7E-05	6.8E-11	7.8E-11		
CFSQ fatigue	SC	0.9	0.8	49	CIF	3.4	0.5	42	WIFS	1.9	0.4	8	CFS	3.7	0.5	58	GWI	3.7	0.5	134			6.8E-11	6.8E-11	6.8E-11		6.8E-11	0.0062	7.0E-11	6.2E-09
CFSQ headaches	SC	0.8	1.2	49	CIF	1.6	1.2	42	WIFS	1.5	1.3	8	CFS	2.6	1.2	58	GWI	2.9	1.1	134		1.1E-07	6.8E-11		1.1E-10	0.023				
CFSQ joint pain	SC	0.7	0.9	49	CIF	1.4	1.3	42	WIFS	2.9	0.8	8	CFS	2.7	1.1	58	GWI	3.2	1.0	134		6.8E-11	6.8E-11		6.8E-11	2.7E-05		0.00011		
CFSQ lymph nodes	SC	0.1	0.3	49	CIF	0.6	0.7	42	WIFS	0.8	1.2	8	CFS	1.4	1.3	58	GWI	1.6	1.3	134		0.00153	9.9E-11		3.8E-06					
CFSQ muscle pain	SC	0.7	1.1	49	CIF	1.8	1.3	42	WIFS	3.1	1.0	8	CFS	3.2	0.9	58	GWI	3.3	0.7	134		6.8E-11	6.8E-11		6.8E-11	6.0E-09	0.0002	2E-07		
CFSQ PEM	SC	0.4	0.9	49	CIF	2.8	1.2	42	WIFS	2.1	1.4	8	CFS	3.5	0.8	58	GWI	3.6	0.7	134		0.00040	6.8E-11	0.010	6.8E-11	0.022	6.8E-11	0.00099	0.032	
CFSQ sleep	SC	1.2	1.4	49	CIF	2.9	1.1	42	WIFS	3.1	0.8	8	CFS	3.6	0.7	58	GWI	3.6	0.6	134		0.0030	6.8E-11		6.8E-11		6.8E-11	6.6E-05		
CFSQ sore throat	SC	0.2	0.5	49	CIF	0.7	0.8	42	WIFS	1.1	1.1	8	CFS	1.5	1.2	58	GWI	1.6	1.2	134		0.00115	7.5E-11		1.6E-06					
Variable	SC	Mean	Sd	n	CIF	mean	sd	n	WIFS	mean	sd	n	CFS	mean	sd	n	GWI	mean	sd	n	GWI >CFS	GWI >CIF	GWI >SC	GWI> WIFS	CFS >SC	CFS >CIF	CIF >SC	WIFS>SC	CFS> WIFS	CIF> WIFS
Chalder Fatigue	SC	12.4	5.4	48	CIF	19.8	6.4	42	WIFS	19.6	3.2	8	CFS	23.7	6.0	53	GWI	25.4	4.8	131		2.4E-05	7.1E-11		7.1E-11		6.5E-07			
CISR Sum	SC	1.5	2.1	50	CIF	5.3	1.4	41	WIFS	5.1	1.5	8	CFS	5.8	0.5	52	GWI	5.8	0.6	132			5.2E-11		5.2E-11		5.2E-11	8.2E-11		
MDFI General Fatigue	SC	8.4	3.9	27	CIF	16.4	3.8	21	WIFS	14.3	4.6	4	CFS	18.6	1.9	32	GWI	18.0	2.8	83			7.6E-11		7.6E-11		7.7E-11			

MDFI Mental Fatigue	SC	8.8	4.0	27	CIF	12.0	3.8	21	WIFS	12.5	5.7	4	CFS	15.7	3.5	32	GWI	15.6	3.5	83			1.4E-10		4.7E-08					
MDFI Physical Fatigue	SC	7.8	4.9	27	CIF	13.9	4.5	21	WIFS	11.5	3.3	4	CFS	16.7	3.1	32	GWI	16.3	3.1	83			7.6E-11		7.6E-11		8.9E-05			
MDFI Reduced Activity	SC	6.7	3.5	27	CIF	14.2	3.6	21	WIFS	10.0	4.7	4	CFS	17.5	2.4	32	GWI	16.3	3.7	83			7.6E-11		7.6E-11		1.1E-08			
MDFI Reduced Motivation	SC	7.1	2.8	27	CIF	12.3	4.2	21	WIFS	11.0	3.8	4	CFS	12.6	3.9	32	GWI	13.6	3.6	83			3.0E-10		5E-05		0.0030			
MDFI ΣDomains	SC	38.9	16.6	27	CIF	68.8	14.6	21	WIFS	59.3	17.3	4	CFS	81.1	9.3	32	GWI	79.8	13.2	83			7.6E-11		7.6E-11		2.9E-09			
Epworth	SC	7.2	5.2	23	CIF	7.3	3.0	20	WIFS	11.8	7.7	4	CFS	9.1	5.4	20	GWI	12.7	6.1	46										
Variable	SC	Mean	Sd	n	CIF	mean	sd	n	WIFS	mean	sd	n	CFS	mean	sd	n	GWI	mean	sd	n	GWI>CFS	GWI>CIF	GWI>SC	GWI>WIFS	CFS>SC	CFS>CIF	CIF>SC	WIFS>SC	CFS>WIFS	CIF>WIFS
SF V RP SF	SC	76.8	23.8	50	CIF	27.9	20.1	42	WIFS	43.6	21.7	8	CFS	15.5	14.4	54	GWI	16.0	13.4	131			7.0E-11	0.021	7E-11		7E-11	0.0010	0.031	
SF10	SC	77.7	24.4	50	CIF	28.3	21.0	42	WIFS	44.8	23.6	8	CFS	14.4	14.4	54	GWI	15.5	13.3	131			7.0E-11	0.010	7E-11		7E-11	0.0021	0.011	
sf36_bp	SC	86.4	19.8	50	CIF	57.4	26.4	42	WIFS	46.6	20.0	8	CFS	35.2	21.9	54	GWI	27.5	19.4	131		1.3E-10	7.0E-11		7E-11	0.00095	5E-07	0.0022		
sf36_gh	SC	73.6	19.8	50	CIF	39.5	18.3	42	WIFS	36.3	19.6	8	CFS	31.9	19.7	54	GWI	24.2	17.1	131		0.0060	7.0E-11		7E-11		7E-11	0.00031		
sf36_mh	SC	75.8	15.9	50	CIF	65.2	17.0	42	WIFS	70.0	12.8	8	CFS	64.7	17.9	54	GWI	52.6	22.4	131			1.5E-08							
sf36_pf	SC	87.0	22.7	50	CIF	64.8	24.6	42	WIFS	61.3	27.1	8	CFS	39.4	22.0	54	GWI	41.6	23.9	131		0.00012	7.0E-11		7E-11	0.00052	0.016			
sf36_re	SC	85.3	31.7	50	CIF	63.5	44.1	42	WIFS	54.2	43.4	8	CFS	61.1	44.7	54	GWI	30.3	40.0	131	0.0060	0.0087	8.4E-11							
sf36_rp	SC	81.5	37.1	50	CIF	20.8	29.7	42	WIFS	34.4	37.6	8	CFS	6.5	18.3	54	GWI	6.3	18.9	131			7.0E-11		7E-11		7E-11	0.0025		
sf36_sf	SC	85.8	22.6	50	CIF	42.3	26.0	42	WIFS	59.4	19.8	8	CFS	25.5	22.8	54	GWI	26.9	21.4	131			7.0E-11		7E-11		7E-11			
sf36_v	SC	63.2	21.7	50	CIF	20.7	15.2	42	WIFS	36.9	14.6	8	CFS	14.4	14.1	54	GWI	14.5	13.6	131			7.0E-11		7E-11		7E-11	0.023		

Variable	SC	Mean	Sd	n	CIF	mean	sd	n	WIFS	mean	sd	n	CFS	mean	sd	n	GWI	mean	sd	n	GWI>CFS	GWI>CIF	GWI>SC	GWI>WIFS	CFS>SC	CFS>CIF	CIF>SC	WIFS>SC	CFS>WIFS	CIF>WIFS
McGill aching	SC	0.7	0.8	49	CIF	1.5	1.1	40	WIFS	2.0	1.3	8	CFS	2.4	0.8	52	GWI	2.5	0.7	130		4.5E-07	6.4E-11		6.4E-11	0.0016	0.012			
McGill Affective	SC	0.5	1.0	49	CIF	2.3	2.5	40	WIFS	2.9	2.9	8	CFS	3.5	2.9	52	GWI	6.3	3.0	130	4.4E-07	6.6E-11	6.4E-11		4.8E-05					
McGill cramping	SC	0.4	0.8	49	CIF	0.6	1.0	40	WIFS	1.1	0.8	8	CFS	1.1	1.1	52	GWI	1.7	1.0	130		4.9E-06	1.1E-10							
McGill fearful	SC	0.1	0.2	49	CIF	0.3	0.5	40	WIFS	0.1	0.4	8	CFS	0.3	0.8	52	GWI	1.0	1.0	130	0.0047	0.0019	1.7E-07							
McGill gnawing	SC	0.1	0.4	49	CIF	0.5	0.9	40	WIFS	0.6	0.7	8	CFS	0.9	1.1	52	GWI	1.3	1.1	130		0.0049	9.9E-10							
McGill heavy	SC	0.2	0.6	49	CIF	0.7	1.0	40	WIFS	1.4	1.1	8	CFS	1.5	1.2	52	GWI	1.5	1.1	130		0.0078	2.2E-10		1.7E-06					
McGill hot_burning	SC	0.1	0.4	49	CIF	0.6	0.8	40	WIFS	0.9	1.1	8	CFS	1.1	1.2	52	GWI	1.5	1.1	130		0.0038	8.9E-11		0.0008					
McGill punishing	SC	0.0	0.2	49	CIF	0.2	0.6	40	WIFS	1.0	1.2	8	CFS	0.4	0.9	52	GWI	1.2	1.2	130	0.0054	3.8E-05	2.3E-08							
McGill Sensory	SC	3.4	5.6	49	CIF	7.8	6.6	40	WIFS	14.3	6.4	8	CFS	14.1	7.2	52	GWI	19.1	6.1	130	0.0036	6.4E-11	6.4E-11		6.6E-11	0.0057		0.016		
McGill sharp	SC	0.4	0.8	49	CIF	0.8	1.1	40	WIFS	1.8	1.2	8	CFS	1.4	1.2	52	GWI	2.0	0.9	130	0.044	4.6E-08	6.4E-11		0.0036					
McGill shooting	SC	0.3	0.6	49	CIF	0.8	1.0	40	WIFS	1.6	1.5	8	CFS	1.1	1.2	52	GWI	1.8	1.0	130		2.6E-05	6.4E-11							
McGill sickening	SC	0.1	0.2	49	CIF	0.4	0.9	40	WIFS	0.6	0.7	8	CFS	0.7	1.0	52	GWI	1.5	1.1	130	0.00019	4.6E-07	6.4E-11							
McGill splitting	SC	0.2	0.6	49	CIF	0.4	0.9	40	WIFS	0.5	0.5	8	CFS	0.7	0.9	52	GWI	1.4	1.1	130		7.5E-05	3.3E-08							
McGill stabbing	SC	0.3	0.7	49	CIF	0.6	0.8	40	WIFS	1.6	1.3	8	CFS	0.9	1.1	52	GWI	1.7	1.0	130	0.0028	3.3E-06	6.5E-11							
McGill tender	SC	0.3	0.6	49	CIF	0.7	1.0	40	WIFS	1.3	1.0	8	CFS	1.8	1.1	52	GWI	1.8	0.9	130		4.1E-07	6.4E-11		1.8E-10	0.00012				
McGill throbbing	SC	0.5	0.8	49	CIF	0.8	0.9	40	WIFS	1.5	1.2	8	CFS	1.3	1.1	52	GWI	1.8	0.9	130		2.0E-06	6.5E-11		0.022					
McGill tiring	SC	0.3	0.6	49	CIF	1.4	1.2	40	WIFS	1.1	1.4	8	CFS	2.0	1.1	52	GWI	2.7	0.7	130	0.039	3.9E-10	6.4E-11	0.0049	6.4E-11		1.4E-05			

McGill Total	SC	3.9	6.5	49	CIF	10.1	8.5	40	WIFS	17.1	7.3	8	CFS	17.6	9.2	52	GWI	25.4	8.3	130	2.3E-05	6.4E-11	6.4E-11		6.8E-11	0.029		0.047		
Pain&Tenderness	SC	6.2	2.6	23	CIF	9.9	2.6	20	WIFS	8.8	1.0	4	CFS	10.5	1.6	21	GWI	10.6	1.7	45			2.8E-10		3.5E-07		4.5E-05			
Variable	SC	Mean	Sd	n	CIF	mean	sd	n	WIFS	mean	sd	n	CFS	mean	sd	n	GWI	mean	sd	n	GWI>CFS	GWI>CIF	GWI>SC	GWI>WIFS	CFS>SC	CFS>CIF	CIF>SC	WIFS>SC	CFS>WIFS	CIF>WIFS
CMSI Bladder	SC	0.8	1.9	48	CIF	1.7	2.6	41	WIFS	4.6	4.9	7	CFS	3.9	4.2	51	GWI	4.7	3.9	129		0.0051	5.4E-07		0.032					
CMSI Cardiac	SC	0.5	1.3	48	CIF	1.7	2.3	41	WIFS	2.1	2.5	7	CFS	3.2	3.2	51	GWI	4.3	3.7	129		0.0067	5.0E-09		0.031					
CMSI Dyspnea	SC	1.6	2.4	48	CIF	4.0	4.5	41	WIFS	3.6	4.2	7	CFS	7.3	5.6	51	GWI	9.1	5.6	129		4.8E-05	7.0E-11		6.3E-05					
CMSI Ear&Sinus	SC	1.2	2.1	48	CIF	4.1	3.2	41	WIFS	4.3	4.6	7	CFS	4.0	3.7	51	GWI	7.9	4.7	129	1.5E-05	0.00024	7.0E-11							
CMSI GI	SC	2.5	5.2	48	CIF	6.4	6.1	41	WIFS	8.0	6.7	7	CFS	8.8	7.7	51	GWI	17.5	8.1	129	8.7E-09	7.2E-11	7.0E-11		0.036					
CMSI Headache	SC	1.0	1.9	48	CIF	1.8	2.0	41	WIFS	2.4	2.1	7	CFS	2.8	2.4	51	GWI	4.4	2.6	129		2.5E-06	7.3E-11							
CMSI Neuro	SC	1.6	2.9	48	CIF	5.2	2.9	41	WIFS	4.6	3.6	7	CFS	7.5	3.9	51	GWI	9.2	3.4	129		6.0E-07	7.0E-11		7.1E-11		0.0011			
CMSI no PAIN	SC	9.2	13.3	48	CIF	24.8	14.2	41	WIFS	29.6	18.3	7	CFS	37.8	20.8	51	GWI	57.1	23.5	129	3.9E-05	7.0E-11	7.0E-11		2.5E-08					
CMSI Rheum	SC	4.9	6.7	48	CIF	12.8	6.0	41	WIFS	15.9	8.8	7	CFS	19.0	7.6	51	GWI	22.7	7.7	129		1.1E-09	7.0E-11		7E-11		0.0011			
CMSI Sum 172	SC	14.1	19.4	48	CIF	37.6	18.7	41	WIFS	45.4	26.4	7	CFS	56.8	26.9	51	GWI	79.8	29.8	129	0.00037	7.0E-11	7.0E-11		9.8E-11					
Variable	SC	Mean	Sd	n	CIF	mean	sd	n	WIFS	mean	sd	n	CFS	mean	sd	n	GWI	mean	sd	n	GWI>CFS	GWI>CIF	GWI>SC	GWI>WIFS	CFS>SC	CFS>CIF	CIF>SC	WIFS>SC	CFS>WIFS	CIF>WIFS
Chemical chemical	SC	12.4	16.7	47	CIF	22.9	22.2	36	WIFS	36.3	24.9	8	CFS	42.7	25.1	47	GWI	45.7	24.7	128		0.00060	5.8E-11		2E-06					
Chemical impact	SC	5.4	11.4	47	CIF	16.3	19.7	36	WIFS	23.6	18.3	8	CFS	33.9	30.2	47	GWI	40.1	27.5	128		0.00119	7.2E-11		0.00011					
Chemical masking	SC	3.8	1.6	47	CIF	3.6	2.0	36	WIFS	5.4	1.8	8	CFS	3.3	1.5	47	GWI	3.8	1.5	128										

Chemical other	SC	6.9	10.9	47	CIF	16.5	12.9	36	WIFS	19.1	14.1	8	CFS	27.2	19.9	47	GWI	24.9	16.7	127			4.1E-07		4.6E-06					
Chemical symptoms	SC	10.6	15.9	47	CIF	27.4	18.6	36	WIFS	40.1	18.9	8	CFS	42.8	22.7	47	GWI	54.8	22.5	128		5.3E-08	5.4E-11		2E-09					
COMPASS Bladder	SC	0.9	2.1	23	CIF	0.9	1.4	20	WIFS	2.8	3.5	4	CFS	1.3	1.5	20	GWI	2.3	2.1	46										
COMPASS GI	SC	4.2	4.6	23	CIF	7.0	3.1	20	WIFS	8.7	3.8	4	CFS	7.7	5.0	20	GWI	12.9	3.2	46	0.0036	0.00022	1.5E-10							
COMPASS OI	SC	8.9	10.0	23	CIF	13.4	7.5	20	WIFS	13.0	9.5	4	CFS	16.4	9.2	20	GWI	18.9	9.7	46										
COMPASS Pupil	SC	1.1	0.9	23	CIF	2.0	0.8	20	WIFS	2.6	0.9	4	CFS	2.6	0.8	20	GWI	3.0	1.0	46		0.049	2.3E-10		0.00026					
COMPASS Secretomotor	SC	2.0	3.1	23	CIF	3.1	3.3	20	WIFS	4.3	3.1	4	CFS	4.4	3.9	20	GWI	6.3	3.7	46			0.012							
COMPASS Sum	SC	17.2	16.2	23	CIF	27.0	8.6	20	WIFS	31.6	16.9	4	CFS	33.1	14.1	20	GWI	44.1	13.9	46		0.016	1.6E-08							
COMPASS Vasomotor	SC	0.0	0.2	23	CIF	0.6	0.9	20	WIFS	0.2	0.4	4	CFS	0.7	1.0	20	GWI	0.7	1.1	46										
Variable	SC	Mean	Sd	n	CIF	mean	sd	n	WIFS	mean	sd	n	CFS	mean	sd	n	GWI	mean	sd	n	GWI>CFS	GWI>CIF	GWI>SC	GWI>WIFS	CFS>SC	CFS>CIF	CIF>SC	WIFS>SC	CFS>WIFS	CIF>WIFS
IRS Congestion	SC	3.7	4.4	45	CIF	6.1	6.3	40	WIFS	11.4	8.1	7	CFS	9.6	7.1	50	GWI	13.5	7.6	123		1.3E-05	8.3E-11							
IRS Rhinorrhea	SC	2.6	3.7	45	CIF	5.1	4.6	40	WIFS	7.7	6.8	7	CFS	6.7	6.2	50	GWI	12.3	7.4	123	0.00045	2.6E-06	6.3E-11							
IRS sum	SC	6.3	7.5	45	CIF	11.2	9.5	40	WIFS	19.1	13.7	7	CFS	16.3	12.2	50	GWI	25.8	14.4	123	0.013	7.5E-07	6.3E-11							
RhSc Airway	SC	5.9	6.5	48	CIF	13.1	9.8	39	WIFS	14.0	10.3	8	CFS	17.0	12.3	50	GWI	28.8	17.4	126	0.00143	4.9E-06	5.9E-11							
RhSc Chest	SC	1.1	2.4	48	CIF	3.9	5.6	39	WIFS	3.9	4.8	8	CFS	5.3	5.8	50	GWI	11.0	9.1	126	0.0041	0.00020	8.3E-11							
RhSc Rhinitis	SC	4.8	5.1	48	CIF	9.2	6.6	39	WIFS	10.1	6.5	8	CFS	11.8	8.0	50	GWI	17.8	9.4	126	0.023	3.6E-05	5.9E-11		0.040					
mTBI ∑	SC	8.6	13.4	23	CIF	14.9	6.2	20	WIFS	21.0	7.9	4	CFS	23.0	10.9	20	GWI	33.6	14.7	46		0.00027	5.9E-09							
Urinary_Act	SC	5.3	5.8	50	CIF	7.9	7.6	41	WIFS	14.0	8.8	8	CFS	11.5	7.4	53	GWI	14.3	8.6	129		0.015	7.4E-08							

USCD Dyspnea Score	SC	5.3	7.2	25	CIF	15.5	18.3	20	WIFS	13.3	8.0	4	CFS	32.3	25.8	29	GWI	37.8	29.6	82			0.00011							
Tobacco Score	SC	17.6	15.0	23	CIF	20.7	19.5	20	WIFS	9.3	12.8	4	CFS	7.7	9.3	21	GWI	14.6	16.6	46										
TOY Sum	SC	20.8	6.8	26	CIF	25.9	6.6	21	WIFS	34.0	5.6	4	CFS	32.0	5.8	31	GWI	32.6	7.5	82			5.2E-09		1.4E-05					
Variable	SC	Mean	Sd	n	CIF	mean	sd	n	WIFS	mean	sd	n	CFS	mean	sd	n	GWI	mean	sd	n	GWI>CFS	GWI>CIF	GWI>SC	GWI>WIFS	CFS>SC	CFS>CIF	CIF>SC	WIFS>SC	CFS>WIFS	CIF>WIFS
GAD7	SC	3.0	5.1	47	CIF	3.8	4.4	37	WIFS	5.5	4.7	8	CFS	6.1	5.6	45	GWI	10.1	6.0	127		6.0E-06	1.5E-09							
STAI	SC	30.6	10.4	27	CIF	44.7	14.3	21	WIFS	44.5	7.3	4	CFS	44.1	15.1	30	GWI	50.2	14.9	80			7.0E-06							
MASQ Anhedonic Depression	SC	27.8	10.4	23	CIF	28.3	8.0	20	WIFS	32.5	15.9	4	CFS	33.3	8.8	21	GWI	36.3	8.0	46										
MASQ Anxious Arousal	SC	14.7	3.7	23	CIF	17.9	4.3	20	WIFS	19.0	5.6	4	CFS	22.9	5.2	21	GWI	26.4	5.7	46		6.8E-06	9.6E-11		0.00058					
MASQ General Distress	SC	16.5	7.7	23	CIF	15.9	4.4	20	WIFS	19.5	2.1	4	CFS	21.9	8.7	21	GWI	26.2	8.0	46		0.00164	0.0025							
TIQ	SC	35.5	16.3	42	CIF	40.4	13.7	31	WIFS	57.8	15.6	5	CFS	45.2	17.7	28	GWI	59.3	19.5	123		0.00065	4.1E-09							
TIQ How much	SC	18.3	8.5	42	CIF	21.2	7.5	31	WIFS	30.6	8.5	5	CFS	23.5	9.9	28	GWI	30.3	9.9	123		0.0044	1.8E-08							
TIQ How often	SC	17.2	8.0	42	CIF	19.2	6.4	31	WIFS	27.2	7.8	5	CFS	21.6	7.9	28	GWI	29.0	9.8	123		0.00015	2.4E-09							
MPTSD	SC	64.8	14.8	32	CIF	72.6	16.5	21	WIFS	95.0	24.3	5	CFS	73.3	14.6	19	GWI	96.7	25.3	114	0.048	0.013	2.1E-08							
PCL-C	SC	26.4	14.6	23	CIF	23.7	4.5	20	WIFS	44.5	13.7	4	CFS	36.8	14.1	21	GWI	51.5	15.8	46		2.9E-08	2.3E-07							
Trauma SQ	SC	1.9	3.0	14	CIF	1.2	1.4	10	WIFS	5.3	3.3	4	CFS	3.4	3.3	14	GWI	5.5	3.1	36										
Variable	SC	Mean	Sd	n	CIF	mean	sd	n	WIFS	mean	sd	n	CFS	mean	sd	n	GWI	mean	sd	n	GWI>CFS	GWI>CIF	GWI>SC	GWI>WIFS	CFS>SC	CFS>CIF	CIF>SC	WIFS>SC	CFS>WIFS	CIF>WIFS
BDI_Anhedonia	SC	2.4	3.6	24	CIF	7.0	3.9	20	WIFS	7.5	5.1	4	CFS	7.7	3.8	29	GWI	9.0	4.1	81			9.2E-08		0.0064					

BDI_Negative	SC	1.6	2.9	24	CIF	6.0	6.9	20	WIFS	7.0	6.3	4	CFS	4.4	4.8	29	GWI	7.2	5.7	81			0.022							
BDI_Score	SC	4.6	6.8	24	CIF	15.3	10.5	20	WIFS	15.8	10.9	4	CFS	13.8	8.2	29	GWI	19.2	10.2	81			8.0E-07							
BDI_Somatic	SC	0.6	1.0	24	CIF	2.4	2.0	20	WIFS	1.3	1.0	4	CFS	1.7	1.4	29	GWI	3.0	1.8	81			4.6E-06							
CESD ≥16=1	SC	0.2	0.4	49	CIF	0.5	0.5	42	WIFS	0.5	0.5	8	CFS	0.7	0.5	53	GWI	0.8	0.4	133		0.026	7.0E-11		1.1E-06					
CESD Anhesdonia	SC	3.0	3.0	49	CIF	4.0	3.0	42	WIFS	3.8	2.4	8	CFS	5.0	3.1	53	GWI	6.2	3.3	133			6.1E-06							
CESD Depressed	SC	2.2	3.3	49	CIF	3.5	3.3	42	WIFS	2.6	2.4	8	CFS	5.3	4.7	53	GWI	7.1	4.9	133		0.011	2.0E-07							
CESD Interpersonal	SC	0.5	1.2	49	CIF	0.7	1.2	42	WIFS	0.8	1.2	8	CFS	0.8	1.3	53	GWI	1.7	1.7	133			0.0082							
CESD Somatic Factor	SC	3.3	3.9	49	CIF	7.8	3.6	42	WIFS	8.0	3.8	8	CFS	10.9	3.9	53	GWI	12.6	3.7	133		7.4E-09	7.0E-11		7E-11		4.7E-05			
CESD Sum	SC	9.0	9.3	49	CIF	16.0	8.6	42	WIFS	15.1	7.0	8	CFS	22.0	9.9	53	GWI	27.5	11.4	133		1.9E-06	7.0E-11		1.6E-06					
Variable	SC	Mean	Sd	n	CIF	mean	sd	n	WIFS	mean	sd	n	CFS	mean	sd	n	GWI	mean	sd	n	GWI>CFS	GWI>CIF	GWI>SC	GWI>WIFS	CFS>SC	CFS>CIF	CIF>SC	WIFS>SC	CFS>WIFS	CIF>WIFS
BCP Chance Happenings	SC	10.9	4.5	26	CIF	10.7	4.1	20	WIFS	10.0	4.1	4	CFS	12.0	4.0	31	GWI	11.4	4.0	79										
BCP Internal Score	SC	15.7	5.3	26	CIF	12.6	4.6	20	WIFS	14.8	3.5	4	CFS	10.5	3.3	31	GWI	11.2	4.5	79			0.027		0.027					
BCP Powerful Doctors	SC	10.6	4.9	26	CIF	10.8	4.7	20	WIFS	12.0	1.6	4	CFS	12.6	4.1	31	GWI	13.3	4.7	79										
CPSSsum	SC	32.2	15.5	26	CIF	51.2	22.1	20	WIFS	55.3	15.4	4	CFS	60.9	16.0	30	GWI	69.7	17.2	79			8.1E-11		9.8E-06					
PBPI Constance	SC	-1.1	1.3	24	CIF	0.7	1.5	20	WIFS	0.1	1.3	4	CFS	0.4	1.3	30	GWI	1.2	0.9	79			1.0E-10		0.018		0.0015			
PBPI Mystery	SC	-1.2	1.2	24	CIF	0.4	1.3	20	WIFS	0.6	0.9	4	CFS	0.6	1.3	30	GWI	0.6	1.0	79			1.5E-07		3.3E-05		0.0056			
PBPI Permanance	SC	-0.7	1.1	24	CIF	0.6	1.1	20	WIFS	-0.5	0.6	4	CFS	0.5	1.0	31	GWI	0.7	1.1	79			0.00017							
PCS Helplessness	SC	2.6	4.0	49	CIF	5.0	5.3	41	WIFS	9.6	6.4	8	CFS	7.3	6.2	52	GWI	10.7	6.5	128		0.00022	8.1E-11							

PCS Magnification	SC	1.2	2.3	49	CIF	2.3	2.6	41	WIFS	3.5	2.9	8	CFS	3.0	2.7	52	GWI	5.1	3.4	128	0.035	0.00070	4.5E-10							
PCS Rumination	SC	2.4	3.8	49	CIF	4.4	4.3	41	WIFS	6.3	5.1	8	CFS	5.7	4.7	52	GWI	8.0	4.8	128		0.024	5.9E-09							
PMD Difficulty Score	SC	0.3	0.6	39	CIF	1.2	1.0	27	WIFS	1.5	1.1	8	CFS	1.8	1.1	42	GWI	2.1	0.9	116		0.012	5.5E-11		1.5E-08					
Variable	SC	Mean	Sd	n	CIF	mean	sd	n	WIFS	mean	sd	n	CFS	mean	sd	n	GWI	mean	sd	n	GWI>CFS	GWI>CIF	GWI>SC	GWI>WIFS	CFS>SC	CFS>CIF	CIF>SC	WIFS>SC	CFS>WIFS	CIF>WIFS
Loneliness	SC	43.2	12.4	13	CIF	34.3	12.2	10	WIFS	55.3	9.5	4	CFS	48.3	12.6	14	GWI	53.7	12.1	35		0.0497								
ASRS_A	SC	8.3	5.1	23	CIF	8.2	4.5	20	WIFS	12.5	3.7	4	CFS	10.8	4.5	21	GWI	14.3	4.9	46		0.0086	0.0064							
ASRS_B	SC	15.4	8.6	23	CIF	14.9	6.6	20	WIFS	18.3	5.6	4	CFS	19.1	8.1	21	GWI	24.4	8.3	46		0.034	0.037							
Rumination	SC	33.7	14.5	22	CIF	31.6	6.4	20	WIFS	39.8	15.9	4	CFS	41.6	13.8	21	GWI	48.2	14.2	46		0.012								
SSAS	SC	23.0	9.9	21	CIF	25.6	5.5	19	WIFS	29.3	5.9	4	CFS	32.6	7.1	20	GWI	29.4	5.4	45					0.030					
Big 5 Neuroticism	SC	11.5	6.5	33	CIF	12.3	7.0	21	WIFS	12.3	5.3	4	CFS	16.0	7.6	22	GWI	17.9	6.8	112			0.0061							
Big 5 Agreeableness	SC	27.0	4.3	33	CIF	27.0	4.5	21	WIFS	26.8	6.7	4	CFS	26.1	5.2	22	GWI	24.8	5.8	112										
Big 5 Conscientiousness	SC	26.6	5.3	33	CIF	23.6	4.9	21	WIFS	26.8	2.4	4	CFS	25.7	7.2	22	GWI	22.6	6.9	112										
Big 5 Extraversion	SC	16.2	6.9	33	CIF	13.6	8.1	21	WIFS	12.8	6.7	4	CFS	14.8	7.6	22	GWI	14.0	6.3	112										
Big 5 Openness	SC	26.5	5.2	33	CIF	27.4	7.7	21	WIFS	28.3	11.5	4	CFS	30.1	6.5	22	GWI	24.7	9.1	112										
Adult_Affective	SC	1.5	2.8	26	CIF	5.1	3.9	21	WIFS	4.0	3.2	4	CFS	3.7	3.1	31	GWI	4.3	3.5	82										
Adult_Confidant	SC	3.3	5.0	26	CIF	8.6	6.2	21	WIFS	8.3	4.8	4	CFS	7.6	6.0	31	GWI	8.0	5.6	82										
Child_Affective	SC	2.2	3.3	26	CIF	4.7	3.8	21	WIFS	3.3	3.8	4	CFS	4.4	3.9	31	GWI	3.7	3.9	82										
Child_Confidant	SC	5.2	6.4	26	CIF	9.0	6.6	21	WIFS	6.8	7.0	4	CFS	8.5	7.1	31	GWI	7.3	6.6	82										

CD-RISC	SC	76.2	14.6	23	CIF	72.0	13.2	19	WIFS	62.5	12.6	4	CFS	68.2	18.3	19	GWI	65.9	18.1	46									
NPI	SC	12.8	8.7	19	CIF	10.4	5.8	11	WIFS	0.0	0.0	0	CFS	9.9	6.7	7	GWI	9.5	5.5	77									
Worry_PSWQ	SC	38.1	15.9	23	CIF	35.4	9.4	20	WIFS	38.3	9.6	4	CFS	45.6	15.5	21	GWI	46.9	14.0	45									
Variable	SC	Mean	Sd	n	CIF	mean	sd	n	WIFS	mean	sd	n	CFS	mean	sd	n	GWI	mean	sd	n	GWI>CFS	GWI>CIF	GWI>SC	GWI>WIFS	CFS>SC	CFS>CIF	CIF>SC	WIFS>SC	CFS>WIFS	CIF>WIFS

.

SOM Table S15. ROC CIF vs ME/CFS.

CIF vs ME/CFS	Threshold	Sensitivity	Specificity	AUC	p
CFSQ cognition	2.5	0.902	0.558	0.778	9.28E-09
CFSQ headaches	2.5	0.686	0.837	0.743	3.82E-06
CFSQ joint pain	2.5	0.647	0.791	0.756	3.62E-07
CFSQ lymph nodes	0.5	0.647	0.512	0.675	1.56E-03
CFSQ muscle pain	2.5	0.882	0.674	0.768	2.27E-07
CFSQ PEM	3.5	0.627	0.628	0.705	2.61E-04
CFSQ sleep	3.5	0.686	0.698	0.728	1.79E-05
CFSQ sore throat	0.5	0.686	0.512	0.672	1.78E-03
McGill Affective	2.5	0.627	0.628	0.661	4.70E-03
McGill Sensory	11.5	0.608	0.651	0.720	2.74E-05
McGill Total	14.5	0.647	0.651	0.724	2.21E-05

SOM Table S16. ROC ME/CFS v SC

Variable	Threshold	Sensitivity	Specificity	Area
MDFI General Fatigue	15.5	0.903	0.926	0.977
MDFI Mental Fatigue	12.5	0.774	0.815	0.891
MDFI Physical Fatigue	13.5	0.774	0.815	0.909
MDFI Reduced Activity	13	0.968	0.926	0.976
MDFI Reduced Motivation	9.5	0.806	0.778	0.875
MDFI ΣDomains	65	0.935	0.926	0.968
Chalder Fatigue	16.5	0.880	0.870	0.897
Chemical domain	20.5	0.722	0.714	0.848
CMSI 172 sum	22.5	0.833	0.810	0.888
CMSI no pain	11.5	0.833	0.810	0.889
McGill Affective	0.5	0.833	0.810	0.882
McGill Sensory	4.0	0.778	0.762	0.933
McGill Total	5.0	0.778	0.762	0.935
COMPASS Pupil	1.8	0.778	0.905	0.931
COMPASS Sum	23.5	0.778	0.762	0.820
COMPASS OI	14.0	0.611	0.667	0.743
COMPASS GI	4.0	0.722	0.667	0.717
COMPASS Secretomotor	1.1	0.667	0.571	0.714
COMPASS Bladder	0.6	0.556	0.810	0.701
COMPASS Vasomotor	0.8	0.333	1.000	0.667

CORRELATION OF DOLORIMETRY WITH SYMPTOM DOMAINS

Dolorimetry (kg) was correlated with symptom scores for females and males in each subgroup. Correlations that were significant after Bonferroni corrections ($p < 0.05$) were tabulated. Explained variances (R^2) were reported for groups larger than 10.

DOLORIMETRY IN MALES

Dolorimetry in SC males was correlated with COMPASS scores for autonomic dysfunction, followed by quality of life, migraine without aura and interoceptive discomfort such as dyspnea (Table S17). Anxious Arousal (MASQ) was present.

When fatigue became elevated in the CIF group, than dolorimetry correlated with Epworth Sleep Questionnaire items for sleepiness and inattentiveness, True of You, joint pain, interoceptive complaints including gastrointestinal and impaired quality of life. Dolorimetry was associated with reduced body mass index.

In GWI males, dolorimetry was most highly correlated with tender point counts (R^2=0.0449), irritable bowel syndrome by Rome III criteria (R^2=0.476), True of You questionnaire (R^2=0.103), McGill Pain Score (R^2=0.120) and interoception (R^2=0.101).

Pain was related to dolorimetry in GWI males. All groups had correlations between dolorimetry and interoceptive items such as CMSI No Pain, True of You (TOY), irritable bowel syndrome diagnoses, migraine and headaches, bladder, pupillary and other complaints. Tenderness (reduced dolorimetry) was correlated with poor quality of life and affective complaints were found in SC and CIF males. Low BMI in CIF males was correlated with dolorimetry.

Table S17. Explained variances (R^2) for dolorimetry with questionnaire domains in male subgroups. All Pearson correlations (R) had $p<0.05$ after Bonferroni correction for multiple comparisons.

GWI Male			CIF Male			SC Male	
83			25			28	
**PAIN**							
Tender Point Count ≥11/18	0.449						
FM_1990	0.476		CFSQ joint pain	0.372			
McGill Sensory	0.120						
**TRUE OF YOU (TOY)**							
TOY Sum	0.103		TOY Sum	0.457			
**INTEROCEPTION**							
CMSI_No Pain	0.101						
IRS Congestion	0.107					RhSc Chest	0.125
			CMSI Neuro	0.174		CMSI Neuro	0.151
						CMSI Cardiac	0.160
			C:masking	0.158		CFSQ lymph nodes	0.101
**GASTROINTESTINAL**							
IBS Rome III	0.286		IBS Rome III	0.105			
IBS Dx	0.176		IBS diarrhea	0.206			
COMPASS GI	0.172		COMPASS GI	0.187			
CMSI GI	0.136						
**COMPASS**							

			COMPASS Bladder	0.299		COMPASS Bladder	0.765
			CMSI Bladder	0.128		COMPASS Secretomotor	0.744
						COMPASS Sum	0.179
			COMPASS Pupil	0.101		COMPASS Pupil	0.270
**MIGRAINE**							
			MO_IHS	0.192		MO_IHS	0.187
CFSQ headaches	0.113		Migraine_IHS	0.121		Migraine_IHS	0.187
CMSI Headache	0.106					CMSI Headache	0.249
**SLEEP**							
			Epworth	0.483			
**QUALITY OF LIFE**							
			sf36_sf	0.357		sf36_sf	0.103
			SF V RP SF	0.295		SF V RP SF	0.123
			SF10	0.248		SF10	0.144
			sf36_bp	0.198		sf36_pf	0.174
			sf36_v	0.193		sf36_gh	0.138
**AFFECT**							
			MASQ Anxious Arousal	0.119		MASQ Anxious Arousal	0.305
						∑GAD7	0.124
**BODY MASS INDEX**							
			BMI	0.115			

DOLORIMETRY IN FEMALES

Dolorimetry in SC females was associated with orthostatic complaints (COPASS), widespread pain, poor quality of life, and Mental Fatigue (MDFI) (Table S18). Dolorimetry and BMI were positively correlated suggesting a relationship between tenderness and "asthenia".

CIF was associated with widespread pain, anhedonia and anxious arousal, sleep and fatigue complaints. Migraine and gastrointestinal complaints were also correlated.

CFS females had the highest correlation of dolorimetry with McGill pain scores (R^2=0.310) and other measures of pain. TOY scores, interoceptive complaints and migraine were correlated with tenderness.

GWI females had correlations with COMPASS GI scores (R^2=0.800), disability (Physical Function R^2=0.416), pain (McGill R^2=0.258) and interoception.

SOM Table S18. Explained variances (R^2) for Dolorimetry (kg) with questionnaire result for females.

GWI Females			CFS Females			CIF Females			SC Females	
n=40			n=48			n=21			n=19	
**PAIN**										
sf36_bp	0.444		McGill Total	0.310		CFSQ joint pain	0.347		Widespread Pain	0.344
CMSI Rheum	0.333		sf36_bp	0.286		Widespread Pain	0.161		CFSQ muscle pain	0.267
CFSQ joint pain	0.322		CFSQ muscle pain	0.285					McGill Affective	0.234
McGill Total	0.258		McGill Sensory	0.276					CMSI Rheum	0.185

CFSQ muscle pain	0.211		FM_1990	0.262					sf36_bp	0.171
FM_1990	0.250		Tender Point Count ≥11/18	0.194					McGill Total	0.113
Tender Point Count ≥11/18	0.211		CMSI Rheum	0.121						
Widespread Pain	0.273									
**DISABILITY**										
sf36_pf	0.462								sf36_rp	0.223
sf36_rp	0.301								SF V RP SF	0.162
SF V RP SF	0.226								SF10	0.160
SF10	0.297								sf36_sf	0.137
									sf36_gh	0.107
**COMPASS AUTONOMIC SCORES**										
COMPASS GI	0.796					COMPASS GI	0.108			
			COMPASS OI	0.148					COMPASS OI	0.353
COMPASS Bladder	0.232		COMPASS Bladder	0.214		COMPASS Secretomotor	0.133		COMPASS Sum	0.423
**INTERO CEPTION**										
IBS Dx	0.212					IBS Rome III	0.164		IBS Dx	0.142
CMSI_Sum	0.235		CMSI Bladder	0.113					CMSI Neuro	0.116
USCD Dyspnea Score	0.436					CMSI Cardiac	0.130		CMSI Cardiac	0.158
IRS Rhinorrhea	0.300		TOY Sum	0.191						
**CHEMICAL EXPOSURES**										
C:chemical	0.278		C:chemical	0.135					C:impact	0.104
C:other	0.207		C:symptoms	0.237						
**FUKUDA CFS CRITERIA**										
CFSQ PEM	0.216		CFSQ sore throat	0.113		CFSQ fatigue	0.133		CFSQ PEM	0.200
			CFSQ lymph nodes	0.100		CFSQ sleep	0.140		Epworth	0.108
**MIGRAINE**										
			MA_IHS	0.106						
			MO_IHS	0.101		MO_IHS	0.127			
**AFFECTIVE DOMAINS**										
						MASQ Anhedonic Depression	0.333		MDFI Mental Fatigue	0.222
						MASQ General Distress	0.257		MASQ General Distress	0.157
						∑GAD7	0.175		MDFI Reduced Activity	0.119
						MASQ Anxious Arousal	0.168			

						CESD Depressed Factor	0.119			
**BODY MASS INDEX**										
									BMI	0.130

Limitation of Dolorimetry Correlations. Once subjects were grouped, their range of symptom scores became restricted. For example, fatigue had to be moderate or severe for CIF diagnosis and so the full range from none to severe was not available for correlations. Dolorimetry pressures were similarly skewed to lower ranges in ME/CFS and GWI. The ceiling and floor effects greatly reduced the mathematical possibility to find significant correlations. Pain, interoception, migraine and disability were still significantly correlated with tenderness across the various groups and in both sexes. The explained variances were low indicating that other variables or confounders that were not recognized in our calculations contributed to the relationships. Dolorimetry and interoception were correlated but with a low, but still significant, explained variance indicating that nociplastic mechanisms contributing to pain had some overlap with interoplastic mechanisms regulating perceptions derived from vagal and other internal visceral sensing systems.

Table S19. True of You questionnaire. Please indicate the degree to which each of the following statements are **TRUE OF YOU** ***in general*** by circling the most appropriate number.

	Not at all true	A little bit true	Moderately true	Quite a bit true	Extremely true
1. I can’t stand smoke, smog, or pollutant in the air.	1	2	3	4	5
2. I am often aware of various things happening within my body.	1	2	3	4	5
3. When I bruise myself, it stays noticeable for a long time.	1	2	3	4	5
4. I sometimes feel the blood flowing through my body.	1	2	3	4	5
5. Sudden loud noises really bother me.	1	2	3	4	5
6. I can sometimes hear my pulse or my heartbeat throbbing in my ear.	1	2	3	4	5
7. I hate to be too hot or too cold.	1	2	3	4	5
8. I am quick to sense the hunger contractions in my stomach.	1	2	3	4	5
9. Even something minor, like an insect bite or a splinter, really bothers me.	1	2	3	4	5
10. I can’t stand pain.	1	2	3	4	5

Table S20. Summary of ROC and effect sizes (Hedges' g) for questionnaires to designate CIF, ME/CFS and GWI from SC.

**CIF v SC**	**Threshold**	**Sensitivity**	**Specificity**	**AUC**	**Hedges' g**
Chalder	15.5	0.857	0.850	0.905	1.4
CISR	4.0	0.929	0.900	0.923	2.3
MDFI General Fatigue	12	0.929	0.850	0.929	2.1
McGill Total	7.5	0.714	0.700	0.745	0.3
CMSI_NoPain	11.5	0.786	0.800	0.763	1.3
SF V RP SF	45.8	0.143	0.150	0.063	2.4
CFSQ fatigue	2.5	1.000	1.000	1.000	3.8
CFSQ PEM	1.5	0.714	0.850	0.875	2.2
CFSQ cognition	1.5	0.786	0.850	0.852	1.7
CFSQ sleep	2.5	0.786	0.800	0.809	1.3
CFSQ joint pain	1.5	0.643	0.700	0.754	0.8
CFSQ muscle pain	1.5	0.643	0.750	0.680	0.9
CFSQ headaches	0.5	0.857	0.650	0.752	0.6
CFSQ lymph nodes	0.5	0.357	1.000	0.679	0.5
CFSQ sore throat	0.5	0.429	0.850	0.639	0.7
**ME/CFS v SC**	**Threshold**	**Sensitivity**	**Specificity**	**AUC**	**Hedges' g**
Chalder	17.5	0.800	0.808	0.872	2.1
CISR	5.5	0.680	0.808	0.865	2.7
MDFI General Fatigue	15.5	0.920	0.808	0.942	2.4
McGill Total	11.5	0.760	0.731	0.833	2.2
CMSI_NoPain	18.5	0.720	0.731	0.811	1.9
SF V RP SF	25.8	0.120	0.115	0.073	2.7
CFSQ fatigue	2.5	1.000	0.885	0.945	3.0
CFSQ PEM	2.5	0.920	0.769	0.882	2.2
CFSQ cognition	2.5	0.760	0.885	0.909	2.2
CFSQ sleep	2.5	0.920	0.692	0.851	1.9
CFSQ joint pain	1.5	0.840	0.692	0.878	2.3
CFSQ muscle pain	2.5	0.800	0.808	0.810	2.2
CFSQ headaches	1.5	0.720	0.808	0.815	1.6
CFSQ lymph nodes	0.5	0.520	0.885	0.713	1.1
CFSQ sore throat	0.5	0.640	0.731	0.730	1.0
**GWI v SC**	**Threshold**	**Sensitivity**	**Specificity**	**AUC**	**Hedges' g**
Chalder	18.5	0.875	0.846	0.944	2.1
CISR	5.5	0.857	0.808	0.886	2.7
MDFI General Fatigue	15.5	0.821	0.808	0.922	2.4
McGill Total	17.5	0.786	0.846	0.906	2.2
CMSI_NoPain	33.5	0.750	0.769	0.866	1.9
SF V RP SF	28.8	0.161	0.154	0.226	2.7
CFSQ fatigue	2.5	0.982	0.885	0.935	3.0
CFSQ PEM	2.5	0.821	0.769	0.839	2.2
CFSQ cognition	2.5	0.768	0.885	0.892	2.2
CFSQ sleep	3.5	0.643	0.885	0.871	1.9

CFSQ joint pain	2.5	0.821	0.923	0.934	2.3
CFSQ muscle pain	2.5	0.946	0.808	0.864	2.2
CFSQ headaches	1.5	0.875	0.808	0.858	1.6
CFSQ lymph nodes	0.5	0.696	0.885	0.801	1.1
CFSQ sore throat	0.5	0.750	0.731	0.774	1.0

Table S21. Raw data.

Age	Gender	BMI	race	ethnicity	Type 2 DM	CCC	CFSFukuda
34	Female	27.4	White	non-hispan	0	1	1
46	Female	24.5	White	Hispanic	0	1	1
43	Female	22.0	White	non-hispan	0	1	1
59	Female	21.3	White	non-hispan	0	1	1
52	Female	27.0	Black	non-hispan	0	1	1
57	Female	28.5	White	non-hispan	0	1	1
59	Female	18.3	White	non-hispan	0	1	1
44	Female	28.0	White	non-hispan	0	1	1
45	Male	40.8	White	non-hispan	0	1	1
57	Male	31.9	White	non-hispan	0	1	1
59	Female	35.3	White	non-hispan	0	1	1
61	Female	32.2	White	non-hispan	1	1	1
41	Female	34.9	White	non-hispan	0	1	1
55	Female	48.0	White	non-hispan	0	1	1
57	Female	20.7	White	non-hispan	0	1	1
48	Male	22.7	White	non-hispan	0	1	1
41	Female	25.0	White	Hispanic	0	1	1
39	Male	26.9	White	non-hispan	0	1	1
35	Female	21.9	White	non-hispan	0	1	1
54	Female	26.4	More than r	non-hispan	0	1	1
31	Female	34.1	White	non-hispan	0	1	1
42	Female	42.5	White	non-hispan	0	1	1
63	Female	23.1	White	non-hispan	0	1	1
52	Female	29.7	White	non-hispan	0	1	1
46	Female	20.4	White	non-hispan	0	1	1
35	Female	28.6	white	non-hispan	0	1	1
50	Male	36.0	AA	non-hispan	1	0	0
33	Female	29.0	white	non-hispan	0	1	1
48	Female	23.9	white	Hispanic	0	1	1
56	Female	34.5	white	non-hispan	0	1	1
65	Female	21.3	white	non-hispan	0	1	1
49	Female	22.0	white	Hispanic	0	1	1
55	Female	23.9	white	non-hispan	0	1	1
52	Female	21.0	white	non-hispan	0	1	1
63	Female	27.2	white	non-hispan	1	1	0
56	Female	35.9	white	non-hispan	0	1	1
47	Female	32.6	white	non-hispan	0	1	1
61	Female	21.4	white	non-hispan	0	1	1
60	Female	19.2	white	non-hispan	0	1	1
49	Female	21.3	white	non-hispan	0	1	1
31	Female	22.9	Asian	non-hispan	0	1	1
31	Male	21.2	white	non-hispan	0	1	1
56	Male	39.4	White	non-hispan	1	1	1

21	Female	35.2	white	non-hispan		0	1	1
51	Female	27.2	White	non-hispan		0	1	1
43	Female	26.0	white	non-hispan		0	1	1
39	Male	37.0	White	non-hispan		0	1	1
49	Male	29.7	white	non-hispan		0	1	1
35	Female	28.1	white	non-hispan		0	1	1
37	Female	26.2	multi/bi rac	non-hispan		0	1	1
33	Female	26.3	Black	non-hispan		0	1	1
35	Female	39.1	White	Hispanic		0	1	1
35	Female	25.5	White	non-hispan		0	1	1
44	Female	25.3	White	non-hispan		0	1	1
59	Female	19.3	white	non-hispan		0	1	1
54	Female	24.1	White	non-hispan		0	1	1
59	Female	44.3	NativeAM	non-hispan		0	1	1
47	Female	40.8	white	non-hispan		0	1	1
30	Male	21.0	Asian	non-hispan		0	1	1
39	Male	25.1	White	non-hispan		0	1	1
34	Male	25.5	White	non-hispan		0	1	1
58	Female	21.2	White	non-hispan		0	1	1
35	Female	21.0	White	non-hispan		0	1	1
20	Female	33.7	White	non-hispan		0	1	1
57	Female	21.8	White	non-hispan		0	1	1
50	Female	24.1	white	non-hispan		0	1	1
45	Female	33.8	white	non-hispan		0	1	1
64	Female	24.4	white	Hispanic	1		1	1
64	Male	34.5	white	non-hispan	1		1	1
47	Female	25.6	white	non-hispan		0	1	1
74	Female	22.4	white	non-hispan		0	1	1
42	Female	21.8	white	non-hispan		0	1	1
50	Male	28.2	black	non-hispan		0	1	1
64	Male	31.8	white	non-hispan		0	1	1
44	Female	22.5	white	non-hispanic			1	1
36	Male	26.0	white	non-hispan		0	1	1
41	Female	28.2	White	non-hispan		0	1	1
20	Male	17.8	white	non-hispan		0	1	1
53	Female	24.0	White	non-hispan		0	1	1
24	Female	33.8	White	non-hispan		0	1	1
46	Male	40.7	White	non-hispan	1		1	1
60	Male	30.7	white	non-hispanic			1	1
50	Male	24.6	white	non-hispanic			1	1
59	Male	27.4	white	non-hispan	1		1	1
52	Male	34.6	Black	non-hispan		0	0	0
47	Male	25.6	white	non-hispan		0	0	0
69	Male	25.7	White	non-hispan		0	1	1

47	Male	25.0	white	non-hispan		0	1	1
54	Male	27.8	White	non-hispan		0	1	1
40	Male	38.8	White	non-hispan		0	0	0
32	Female	30.3	More than r	non-hispan		0	0	0
48	Male	37.7	AA	non-hispan	1		0	0
47	Male	36.4	white	Hispanic		0	1	1
45	Male	25.1	white	non-hispan		0	1	1
61	Female	28.3	white	non-hispan		0	0	0
46	Female	25.6	white	non-hispan		0	1	1
41	Male	40.3	White	non-hispan		0	0	0
35	Male	28.7	White	non-hispan		0	1	0
45	Female	38.6	white	non-hispan		0	1	1
42	Male	35.1	White	non-hispan		0	0	1
53	Male	33.4	White	non-hispan		0	0	0
42	Male	38.1	White	non-hispan		0	0	0
48	Female	37.9	Asian	non-hispan	1		0	0
55	Male	28.0	White	non-hispan		0	0	0
53	Male	33.4	White	non-hispan		0	0	0
45	Male	27.2	white	non-hispan		0	1	1
47	Female	23.2	white	non-hispan		0	1	1
50	Male	23.0	white	non-hispan		0	1	1
31	Male	26.2	white	non-hispan		0	1	1
72	Male	23.2	white	non-hispan		0	1	1
50	Male	30.7	white	non-hispan		0	1	1
48	Male	24.2	white	non-hispan		0	1	1
45	Male	26.0	white	non-hispan		0	1	1
47	Male	32.3	Other	Hispanic		0	1	1
48	Male	29.9	white	non-hispan		0	1	1
51	Male	29.9	white	non-hispan		0	1	1
46	Male	35.5	white	non-hispan		0	1	1
41	Male	27.0	White	non-hispan		0	1	1
43	Male	29.9	AA	non-hispan		0	1	1
57	Male	29.3	white	non-hispan	1		1	1
47	Male	30.2	white	non-hispan		0	1	1
62	Male	29.5	white	Hispanic		0	0	0
45	Female	25.7	Prefer NOT	non-hispan		0	1	1
43	Female	24.8	White	non-hispan		0	1	1
40	Male	33.8	White	non-hispan		0	1	1
40	Male	35.8	White	non-hispan		0	1	1
42	Male	28.6	White	non-hispan		0	1	1
56	Male	31.9	White	non-hispan		0	1	1
55	Male	28.0	White	non-hispan		0	1	1
66	Female	33.7	White	non-hispan		0	1	1
49	Male	33.3	white	non-hispan		0	1	1

41	Male	27.2	White	non-hispan	0	1	1
45	Male	27.9	White	non-hispan	0	1	1
36	Male	27.4	White	non-hispan	0	1	1
45	Male	32.5	White	non-hispan	1	1	1
41	Male	33.0	White	non-hispan	0	1	1
47	Male	23.1	White	non-hispan	0	1	1
31	Male	22.5	White	non-hispan	0	1	1
39	Male	24.6	White	non-hispan	0	1	1
55	Female	32.7	White	non-hispan	0	1	1
42	Female	21.2	White	Prefer not t	0	1	1
43	Female	24.5	White	non-hispan	0	1	1
52	Female	21.3	white	non-hispan	0	0	0
53	Male	30.5	white	non-hispanic		1	1
49	Male	26.6	AA	non-hispan	0	1	1
46	Male	31.4	white	non-hispan	0	1	1
50	Male	34.1	white	non-hispan	1	1	1
47	Male	28.1	white	Hispanic	0	1	1
46	Male	30.4	white	non-hispan	0	1	1
52	Male	32.5	white	non-hispan	0	1	1
53	Male	30.8	white	non-hispan	0	1	1
55	Male	41.3	Multi	non-hispan	1	1	1
51	Male	36.8	white	non-hispan	0	1	0
36	Female	26.2	Multi	Hispanic	0	1	1
40	Female	18.5	asian	non-hispan	0	1	1
35	Female	20.4	Native Ame	non-hispan	0	1	1
55	Male	28.3	white	non-hispan	0	1	1
60	Female	27.1	White	non-hispan	0	1	1
33	Female	22.3	white	non-hispan	0	1	1
42	Male	26.5	White	non-hispan	0	1	1
52	Male	31.0	White	non-hispan	0	1	1
43	Male	27.4	White	non-hispan	0	0	1
39	Male	24.8	White	non-hispan	0	1	1
63	Male	27.0	White	non-hispan	0	1	1
51	Female	31.0	white	non-hispan	0	1	1
55	Male	31.9	white	non-hispan	0	1	1
42	Female	24.5	White	non-hispan	0	1	1
52	Female	20.7	White	non-hispan	0	1	1
32	Female	22.6	White	non-hispan	0	1	1
54	Male	25.3	white	non-hispan	0	1	1
49	Male	40.5	AA	non-hispan	0	1	1
42	Female	29.2	White	non-hispan	0	1	1
42	Female	29.2	White	non-hispan	0	1	1
42	Female	29.2	White	non-hispan	0	1	1
49	Male	45.9	white	non-hispan	1	1	1

49	Female	28.2	white	hispanic		0	1	1
51	Male	27.2	white	non-hispan		0	1	1
46	Male	31.9	Black	non-hispan		0	1	1
52	Female	20.7	White	non-hispan		0	1	1
50	Male	37.3	White	non-hispan		0	1	1
43	Male	27.7	Black	non-hispan		0	1	1
40	Male	26.6	White	non-hispan		0	1	1
43	Male	28.7	White	non-hispan		0	1	1
52	Female	31.3	White	non-hispan		0	1	1
50	Male	32.4	White	non-hispan		0	1	1
47	Male	28.7	white	non-hispan		0	1	1
56	Male	39.0	white	non-hispan		0	1	1
31	Female	30.3	More than r	non-hispan		0	0	1
52	Male	31.0	White	non-hispan		0	1	1
41	Male	34.0	White	non-hispan		0	1	1
53	Female	40.5	More than r	Hispanic		0	1	1
50	Male	37.4	White	non-hispan		0	1	1
41	Female	30.6	White	non-hispan		0	1	1
63	Female	28.6	White	non-hispan	1		1	1
42	Male	35.1	White	non-hispan		0	1	1
45	Male	38.2	white	non-hispan		0	1	1
52	Male	43.2	White	non-hispan	1		1	1
51	Female	31.3	White	non-hispan		0	1	1
48	Male	38.3	White	non-hispan	1		1	1
48	Male	36.3	White	non-hispan	1		1	1
48	Female	39.3	Prefer NOT	non-hispan		0	1	1
42	Male	35.1	White	non-hispan		0	0	1
35	Male	24.3	white	non-hispan		0	1	1
58	Female	29.2	White	non-hispan		0	1	1
42	Male	34.0	White	non-hispan		0	1	1
42	Male	34.0	White	non-hispan		0	1	1
54	Female	35.8	More than r	Hispanic		0	1	1
54	Female	35.8	More than r	Hispanic		0	1	1
41	Male	30.0	White	Hispanic		0	1	1
53	Male	33.4	White	non-hispan		0	1	1
35	Male	28.5	White	non-hispan		0	1	1
49	Female	39.3	Prefer NOT	non-hispan	1		1	1
41	Male	25.2	Black	non-hispan		0	1	1
43	Male	27.4	White	non-hispan		0	1	1
47	Female	27.3	White	non-hispan		0	1	1
38	Male	27.4	White	non-hispan		0	1	1
41	Female	29.2	native ame	non-hispan		0	1	1
52	Male	31.0	White	non-hispan		0	1	1
68	Female	33.7	White	non-hispan		0	1	1

40	Male	33.8	More than r	non-hispan	1		1	1
68	Female	33.7	White	non-hispan		0	1	1
68	Female	33.7	White	non-hispan		0	1	1
41	Male	26.5	White	non-hispan		0	1	1
40	Male	33.8	More than r	non-hispan	1		1	1
52	Male	31.0	White	non-hispan		0	1	1
51	Male	32.4	White	non-hispan		0	1	1
52	Female	20.7	White	non-hispan		0	1	1
41	Male	27.0	White	non-hispan		0	1	1
31	Male	22.9	White	non-hispan		0	1	1
42	Female	24.5	White	non-hispan		0	1	1
63	Male	26.2	White	non-hispan		0	1	1
43	Male	33.8	Prefer NOT	non-hispan	1		1	1
39	Male	24.6	White	non-hispan		0	1	1
55	Male	28.0	White	non-hispan		0	1	1
51	Male	32.4	White	non-hispan		0	1	1
31	Male	22.9	White	non-hispan		0	1	1
51	Male	32.4	White	non-hispan		0	1	1
53	Male	33.4	White	non-hispan		0	0	1
42	Male	34.0	White	non-hispan		0		1
49	Female	39.3	Prefer NOT	non-hispan	1			1
54	Female	35.8	More than r	Hispanic		0		1
68	Female	33.7	White	non-hispan		0		1
42	Female	24.5	White	non-hispan		0		1
42	Male	26.5	White	non-hispan		0		1
49	Male	30.7	white	non-hispanic			1	1
68	Female	33.7	White	non-hispan		0		1
68	Female	33.7	White	non-hispan		0		1
42	Male	35.1	White	non-hispan		0		1
42	Male	35.1	White	non-hispan		0		1
42	Female	19.4	White	Hispanic		0	0	0
65	Female	33.3	White	non-hispan		0	0	0
56	Male	24.3	White	non-hispan		0	0	0
48	Female	24.0	White	non-hispan		0	0	0
45	Female	20.4	White	Hispanic		0	0	0
48	Female	27.4	white	non-hispan		0	1	1
60	Male	20.1	white	non-hispan		0	0	0
66	Female	22.6	white	non-hispan		0	0	0
57	Male	24.3	white	non-hispan		0	0	0
47	Male	28.7	White	non-hispan		0	0	0
51	Male	30.1	white	non-hispan		0	0	0
22	Female	22.9	White	non-hispan		0	0	0
33	Female	25.5	White	non-hispan		0	0	0
54	Male	32.9	White	non-hispan		0	0	0

62	Male	37.5	White	non-hispan	1	0	0
26	Female	22.9	White	non-hispan	0	0	0
50	Female	39.0	White	non-hispan	0	0	0
46	Male	26.2	White	non-hispan	0	0	0
34	Male	27.6	White	non-hispan	0	0	0
55	Male	31.2	White	non-hispan	0	0	0
46	Male	29.3	White	non-hispan	0	0	0
32	Male	24.9	White	non-hispan	0	0	0
19	Male	30.2	black	non-hispan	0	0	0
30	Male	21.9	white	non-hispan	0	1	1
19	Female	24.2	Pacific Isla	non-hispan	0	0	0
20	Female	21.7	Asian	non-hispan	0	0	0
20	Female	23.4	white	Hispanic	0	0	0
21	Female	25.2	white	non-hispan	0	0	0
63	Female	28.9	white	non-hispan	0	0	0
64	Male	27.7	white	non-hispan	0	0	0
59	Male	25.6	White	non-hispan	0	0	0
43	Female	37.7	white	non-hispan	0	0	0
26	Male	25.8	black	non-hispan	0	0	0
22	Male	24.4	white	non-hispan	0	0	0
41	Male	33.4	White	non-hispan	0	0	0
63	Male	33.9	white	non-hispan	0	0	0
19	Male	31.6	white	non-hispan	0	0	0
71	Female	28.6	White	non-hispan	0	0	0
23	Female	24.6	White	non-hispan	0	0	0
71	Female	28.6	White	non-hispan	0	0	0
53	Male	25.7	white	non-hispan	0	0	0
38	Male	32.8	Black	non-hispan	0	0	0
45	Male	30.6	White	Hispanic	0	0	0
32	Male	24.3	White	non-hispan	0	0	0
49	Male	27.4	White	non-hispan	0	0	0
62	Female	29.1	white	non-hispan	0	0	0
45	Male	35.2	White	non-hispan	0	0	0
56	Female	23.3	White	non-hispan	0	0	0
49	Female	35.3	white	non-hispan	0	0	0
60	Female	25.7	White	non-hispan	0	0	0
53	Male	33.4	White	non-hispan	0	0	0
24	Female	33.6	white	non-hispan	0	0	0
28	Female	31.4	White	non-hispan	0	0	0
64	Male	26.7	White	non-hispan	0	0	0
26	Male	23.3	White	non-hispan	0	0	0
57	Male	21.7	White	non-hispan	0	0	0
53	Male	22.9	White	non-hispan	0	0	0
71	Female	28.6	White	non-hispan	0	0	0

35	Male	22.0	white	non-hispan	0	0	0
26	Male	21.4	Asian	non-hispan	0	0	0
50	Male	28.2	white	non-hispanic		0	0
72	Male	27.4	white	non-hispanic		0	0
30	Male		Black	non-hispan	0	0	0

SEID	Veteran	Kansas	CMI	Kansas+CN	GWI=Vet+Kan+CMI	GWI CCC S
	0	1	1	1	0	CCC
	0	1	1	1	0	CCC
	0	1	1	1	0	CCC
	0	1	1	1	0	CCC
	0	1	1	1	0	CCC
	0	1	1	1	0	CCC
	0	1	1	1	0	CCC
	0	1	1	1	0	CCC
	0	1	1	1	0	CCC
	0	1	1	1	0	CCC
	0	1	1	1	0	CCC
	0	1	1	1	0	CCC
	0	0	1	0	0	CCC
	0	1	1	1	0	CCC
	0	1	1	1	0	CCC
	0	1	1	1	0	CCC
	0	1	1	1	0	CCC
	0	1	1	1	0	CCC
	1	1	1	1	0	CCC
	0	1	1	1	0	CCC
	0	1	1	1	0	CCC
	0	1	1	1	0	CCC
	0	1	1	1	0	CCC
	0	1	1	1	0	CCC
	0	0	1	0	0	CCC
1	0	1	1	1	0	CCC
0	1	0	0	0	0	CCC
	0	1	1	1	0	CCC
	0	0	1	0	0	CCC
1	0	1	1	1	0	CCC
	0	1	1	1	0	CCC
	0	0	1	0	0	CCC
1	0	1	1	1	0	CCC
1	0	1	1	1	0	CCC
	0	1	1	1	0	CCC
	0	1	1	1	0	CCC
1	0	0	1	0	0	CCC
1	0	1	1	1	0	CCC
1	0	1	1	1	0	CCC
0	0	0	0	0	0	CCC
	0	1	1	1	0	CCC
1	0	1	1	1	0	CCC
	0	1	1	1	0	CCC

1	0	1	1	1	0	CCC
	0	1	1	1	0	CCC
	0	1	1	1	0	CCC
	0	1	1	1	0	CCC
0	0	1	1	1	0	CCC
1	0	1	1	1	0	CCC
1	0	1	1	1	0	CCC
	0	1	1	1	0	CCC
	0	1	1	1	0	CCC
	0	1	1	1	0	CCC
	0	1	1	1	0	CCC
1	0	1	1	1	0	CCC
	0	1	1	1	0	CCC
	0	1	1	1	0	CCC
1	0	1	1	1	0	CCC
1	0	1	1	1	0	CCC
	0	1	1	1	0	CCC
	0	1	1	1	0	CCC
	0	0	1	0	0	CCC
	0	0	0	0	0	CCC
	0	0	0	0	0	CCC
	0	0	0	0	0	CCC
	0	1	1	1	0	CCC
1	0	0	1	0	0	CCC
	0	0	1	0	0	CCC
	0	0	1	0	0	CCC
	0	0	0	0	0	CCC
	0	1	1	1	0	CCC
1	0	1	1	1	0	CCC
1	0	0	1	0	0	CCC
1	0	1	1	1	0	CCC
1	0	0	1	0	0	CCC
1	0	1	1	1	0	CCC
	0	0	0	0	0	CCC
1	0	1	1	1	0	CCC
	0	1	1	1	0	CCC
	0	0	0	0	0	CCC
	0	0	0	0	0	CCC
1	1	1	1	1	0	CCC
1	1	1	1	1	1	GWI
1	1	1	1	1	1	GWI
	1	1	1	1	1	GWI
0	1	1	1	1	1	GWI
	1	1	1	1	1	GWI

1	1	1	1	1	1	GWI
	1	1	1	1	1	GWI
	1	1	1	1	1	GWI
	1	1	1	1	1	GWI
0	1	1	1	1	1	GWI
1	1	1	1	1	1	GWI
	1	1	1	1	1	GWI
0	1	1	1	1	1	GWI
1	1	1	1	1	1	GWI
	1	1	1	1	1	GWI
	1	1	1	1	1	GWI
1	1	1	1	1	1	GWI
	1	1	1	1	1	GWI
	1	0	0	0		GWI
	1	1	1	1	1	GWI
	1	1	1	1	1	GWI
	1	1	1	1	1	GWI
	1	0	0	0		GWI
1	1	1	1	1	1	GWI
1	1	1	1	1	1	GWI
1	1	1	1	1	1	GWI
1	1	1	1	1	1	GWI
1	1	1	1	1	1	GWI
1	1	1	1	1	1	GWI
1	1	1	1	1	1	GWI
1	1	1	1	1	1	GWI
1	1	1	1	1	1	GWI
1	1	1	1	1	1	GWI
0	1	1	1	1	1	GWI
1	1	1	1	1	1	GWI
	1	1	1	1	1	GWI
1	1	1	1	1	1	GWI
1	1	1	1	1	1	GWI
1	1	1	1	1	1	GWI
0	1	1	1	1	1	GWI
	1	1	1	1	1	GWI
	1	1	1	1	1	GWI
	1	1	1	1	1	GWI
	1	1	1	1	1	GWI
	1	1	1	1	1	GWI
	1	1	1	1	1	GWI
	1	1	1	1	1	GWI
	1	1	1	1	1	GWI
1	1	1	1	1	1	GWI

	1	1	1	1	1	GWI
	1	1	1	1	1	GWI
	1	1	1	1	1	GWI
	1	1	1	1	1	GWI
	1	1	1	1	1	GWI
	1	1	1	1	1	GWI
	1	1	1	1	1	GWI
	1	1	1	1	1	GWI
	1	1	1	1	1	GWI
	1	0	1	0		GWI
	1	1	1	1	1	GWI
1	1	1	1	1	1	GWI
1	1	1	1	1	1	GWI
1	1	1	1	1	1	GWI
0	1	1	1	1	1	GWI
0	1	1	1	1	1	GWI
1	1	1	1	1	1	GWI
1	1	1	1	1	1	GWI
1	1	1	1	1	1	GWI
1	1	1	1	1	1	GWI
	1	1	1	1	1	GWI
	1	1	1	1	1	GWI
	1	1	1	1	1	GWI
	1	1	1	1	1	GWI
	1	1	1	1	1	GWI
	1	1	1	1	1	GWI
	1	1	1	1	1	GWI
	1	1	1	1	1	GWI
	1	1	1	1	1	GWI
	1	1	1	1	1	GWI
	1	1	1	1	1	GWI
	1	1	1	1	1	GWI
	1	1	1	1	1	GWI
0	1	1	1	1	1	GWI
	1	1	1	1	1	GWI
	1	1	1	1	1	GWI
	1	1	1	1	1	GWI
	1	1	1	1	1	GWI
1	1	1	1	1	1	GWI
0	1	1	1	1	1	GWI
	1	1	1	1	1	GWI
	1	1	1	1	1	GWI
	1	1	1	1	1	GWI
1	1	1	1	1	1	GWI

	1	1	1	1	1	GWI
1	1	1	1	1	1	GWI
	1	1	1	1	1	GWI
	1	1	1	1	1	GWI
	1	1	1	1	1	GWI
	1	1	1	1	1	GWI
	1	1	1	1	1	GWI
	1	1	1	0	1	GWI
	1	1	1	1	1	GWI
	1	1	1	1	1	GWI
1	1	1	1	1	1	GWI
1	1	1	1	1	1	GWI
	1	1	1	1	1	GWI
	1	1	1	1	1	GWI
	1	1	1	1	1	GWI
	1	1	1	1	1	GWI
	1	1	1	1	1	GWI
	1	1	1	1	1	GWI
	1	1	1	1	1	GWI
	1	1	1	1	1	GWI
1	1	1	1	1	1	GWI
	1	1	1	1	1	GWI
	1	1	1	1	1	GWI
	1	1	1	1	1	GWI
	1	1	1	1	1	GWI
	1	1	1	1	1	GWI
	1	1	1	1	1	GWI
0	1	1	1	1	1	GWI
	1	1	1	1	1	GWI
	1	1	1	1	1	GWI
	1	1	1	1	1	GWI
	1	1	1	1	1	GWI
	1	1	1	1	1	GWI
	1	1	1	1	1	GWI
	1	1	1	1	1	GWI
	1	1	1	1	1	GWI
	1	1	1	1	1	GWI
	1	1	1	1	1	GWI
	1	1	1	1	1	GWI
	1	1	1	1	1	GWI
	1	1	1	1	1	GWI
1	1	1	1	1	1	GWI
	1	1	1	1	1	GWI
	1	1	1	1	1	GWI

	1	1	1	1	1	GWI
	1	1	1	1	1	GWI
	1	1	1	1	1	GWI
	1	1	1	1	1	GWI
	1	1	1	1	1	GWI
	1	1	1	1	1	GWI
	1	1	1	1	1	GWI
	1	1	1	1	1	GWI
	1	1	1	1	1	GWI
	1	1	1	1	1	GWI
	1	1	1	1	1	GWI
	1	1	1	1	1	GWI
	1	1	1	1	1	GWI
	1	1	1	1	1	GWI
	1	1	1	1	1	GWI
	1	1	1	1	1	GWI
	1	1	1	1	1	GWI
	1	1	1	1	1	GWI
	1	1	1	1	1	GWI
	1	1	1	1	1	GWI
	1	1	1	1	1	GWI
	1	1	1	1	1	GWI
	1	1	1	1	1	GWI
	1	1	1	1	1	GWI
	1	1	1	1	1	GWI
1	1	1	1	1	1	GWI
	1	1	1	1	1	GWI
	1	1	1	1	1	GWI
	1	1	1	1	1	GWI
	1	1	1	1	1	GWI
	0	1	1	1	0	SC
	0	0	0	0	0	SC
	0	0	0	0	0	SC
	0	0	0	0	0	SC
	0	0	0	0	0	SC
	1	0	1	0	0	SC
	0	0	1	0	0	SC
	0	0	1	0	0	SC
1	0	0	0	0	0	SC
	0	0	1	0	0	SC
0	0	0	0	0	0	SC
	0	0	0	0	0	SC
	0	0	0	0	0	SC
	0	0	0	0	0	SC

	0	0	0	0	0	SC
	0	0	0	0	0	SC
	0	0	0	0	0	SC
	1	0	0	0	0	SC
	0	0	0	0	0	SC
	1	0	0	0	0	SC
	1	0	0	0	0	SC
	0	0	0	0	0	SC
0	0	0	0	0	0	SC
	0	0	0	0	0	SC
	0	0	0	0	0	SC
	0	0	0	0	0	SC
	0	0	0	0	0	SC
	0	0	0	0	0	SC
	0	0	0	0	0	SC
	0	0	0	0	0	SC
0	0	0	0	0	0	SC
0	0	0	0	0	0	SC
0	0	0	0	0	0	SC
0	0	0	0	0	0	SC
0	1	0	0	0	0	SC
	1	0	0	0	0	SC
0	0	0	0	0	0	SC
	1	0	0	0	0	SC
	0	0	0	0	0	SC
	1	0	0	0	0	SC
	0	0	0	0	0	SC
	0	0	0	0	0	SC
	1	0	0	0	0	SC
	0	0	0	0	0	SC
	0	0	0	0	0	SC
	0	0	0	0	0	SC
	0	0	0	0	0	SC
0	0	0	0	0	0	SC
0	1	0	0	0	0	SC
	0	0	0	0	0	SC
	1	0	0	0	0	SC
0	0	0	0	0	0	SC
	0	0	1	0	0	SC
	1	0	1	0	0	SC
	0	0	0	0	0	SC
	0	0	0	0	0	SC
	0	0	0	0	0	SC
	1	0	0	0	0	SC

0		0	0	0	0	0	SC
		0	0	0	0	0	SC
		0	0	0	0	0	SC
1		1	0	1	0	0	SC
		0	0	0	0	0	SC

CFSQ fatigu	CFSQ cogn	CFSQ sore	CFSQ lymp	CFSQ musc	CFSQ joint	CFSQ head	CFSQ sleep
4	4	3	3	4	3	4	4
4	3	3	3	4	3	1	4
4	3	2	3	4	4	3	3
4	4	3	3	3	1	3	4
4	4	3	1	4	4	4	4
3	3	2	1	4	3	4	3
4	3	2	2	3	3	3	3
3	3	0	0	4	4	3	4
4	4	3	0	4	3	2	4
4	3	2	1	4	3	3	3
4	3	0	3	3	3	1	4
4	3	1	0	4	4	3	4
4	2	2	2	4	3	3	4
3	4	2	0	4	4	1	3
4	4	0	2	3	2	4	4
3	3	0	0	3	3	0	3
4	3	2	2	3	3	3	2
3	2	1	0	4	4	3	4
4	3	2	2	3	2	3	4
4	4	3	0	3	3	1	4
3	3	4	3	4	1	1	1
4	3	2	4	4	3	1	2
3	4	0	1	1	2	3	4
3	4	0	0	0	3	0	4
4	2	0	1	4	2	4	3
4	3	1	0	3	2	3	2
3	3	2	1	3	3	3	3
4	4	0	3	4	4	3	4
3	2	0	0	3	2	3	3
4	3	0	0	4	1	1	4
3	3	0	0	3	0	2	3
3	4	1	0	1	1	3	4
4	3	3	4	3	1	1	4
4	3	2	1	4	4	4	4
4	3	0	0	3	0	1	4
3	4	0	0	3	2	2	4
4	3	2	2	2	2	3	4
4	4	3	3	4	4	4	4
4	4	0	0	3	4	2	4
3	3	2	3	2	1	2	3
3	3	2	2	1	0	3	3
4	3	2	0	3	1	1	4
4	4	0	3	3	3	3	4

3	2	2	2	3	3	3	3
4	4	3	3	4	3	4	4
4	3	1	1	3	2	4	4
4	2	3	2	3	3	3	4
3	3	1	1	4	2	3	3
4	4	3	1	4	4	4	3
4	3	1	2	4	4	1	4
4	4	0	0	4	4	4	4
4	4	3	3	3	4	4	4
4	3	1	3	3	3	3	4
3	3	2	1	3	3	3	3
3	4	0	2	3	3	4	4
4	4	1	2	3	2	3	4
4	3	0	0	3	4	0	4
2	3	2	0	2	4	2	3
3	2	0	0	3	0	2	2
4	4	0	1	1	0	3	4
4	3	2	0	0	0	4	4
3	2	0	0	3	2	2	3
4	2	1	0	2	1	1	3
4	2	2	1	2	2	1	4
4	1	0	0	0	1	2	0
3	4	0	0	4	0	0	2
4	3	2	2	2	2	2	3
3	4	1	1	2	2	2	4
3	3	0	0	0	0	0	3
3	1	2	1	2	0	3	2
4	3	0	1	2	1	2	1
3	2	0	0	2	3	0	3
4	3	2	1	0	1	1	4
4	1	0	0	4	2	0	3
4	3	2	1	1	1	1	4
4	3	2	1	1	2	1	3
4	2	0	1	1	0	4	4
4	3	1	2	0	0	2	3

2	3	1	0	4	3	1	4
2	3	0	0	4	3	0	3
2	4	2	3	3	3	2	2
2	3	0	0	4	3	0	4
3	3	0	1	2	2	2	3

3	2	0	1	3	3	2	3
4	2	0	0	4	3	1	3
3	3	1	0	1	2	2	3
3	3	2	0	0	0	0	2
3	3	0	0	2	2	4	2
3	2	0	0	3	3	0	2
3	3	1	1	1	1	2	3
3	2	1	0	4	3	1	0
4	3	0	0	2	2	1	4
3	2	0	1	3	4	1	4
3	2	2	2	4	3	2	3
3	3	1	1	2	3	1	3
4	2	0	0	4	4	2	4
3	2	0	0	0	2	2	0
3	2	0	1	3	4	1	4
3	3	0	0	0	0	0	4
3	1	0	0	3	0	3	1
3	2	1	1	0	2	3	2
4	4	3	3	4	4	4	3
4	3	4	3	3	3	2	4
4	4	0	0	4	4	2	3
4	3	2	2	2	3	3	4
4	3	0	0	2	3	1	4
4	3	0	0	3	4	3	4
4	4	0	2	4	4	2	3
4	3	4	4	4	4	3	3
4	3	2	2	4	3	4	4
4	4	1	2	3	4	4	4
3	2	3	2	3	3	3	4
4	4	2	2	4	4	4	4
4	4	2	2	3	3	3	4
3	3	3	3	3	3	2	4
3	2	1	1	3	3	1	4
4	3	0	1	4	4	4	4
2	1	0	0	2	3	1	3
4	2	3	4	4	4	4	4
4	3	0	3	4	4	3	4
3	3	0	3	3	3	3	4
4	3	1	3	4	4	3	4
3	3	3	3	4	4	1	3
3	3	2	2	3	4	3	4
4	3	1	2	3	4	4	4
4	3	2	1	4	4	4	4
4	3	0	3	4	3	4	3

4	3	3	1	3	2	3	4
4	4	0	0	3	3	3	4
3	2	2	2	3	3	3	4
3	2	1	2	4	4	3	4
4	4	0	0	4	4	2	4
4	2	2	1	4	3	4	4
3	3	2	2	3	3	2	3
4	4	2	1	4	4	2	4
3	0	1	0	3	4	0	3
3	3	3	0	1	2	2	3
4	3	2	0	3	4	2	4
2	3	0	2	0	0	4	2
4	3	1	0	4	4	3	4
4	4	4	4	4	4	4	4
4	3	1	1	2	3	2	4
3	3	3	3	3	3	3	4
4	3	3	2	4	4	4	4
4	4	2	1	3	3	3	4
3	4	0	0	3	4	1	4
4	4	2	3	4	4	3	4
4	4	2	3	3	3	3	4
4	4	0	3	4	4	3	4
4	4	1	2	4	4	4	4
4	4	3	1	2	3	4	4
4	3	2	3	3	4	1	4
4	3	1	1	4	4	4	3
4	3	1	0	3	3	2	3
4	4	2	2	4	4	1	4
4	4	2	1	3	4	3	4
4	3	0	0	3	4	3	4
4	4	3	3	3	4	0	4
4	1	3	0	3	3	4	3
4	3	2	0	3	0	4	4
4	3	2	3	3	2	4	4
3	3	1	1	4	3	4	2
4	4	3	1	3	3	3	2
3	3	0	0	3	3	3	3
3	1	1	1	3	3	2	4
4	4	3	3	4	4	4	3
2	1	0	1	1	1	2	1
4	3	2	0	3	4	1	4
4	3	2	0	4	4	1	4
4	3	2	1	3	3	0	3
4	3	0	0	3	4	3	4

4	3	3	0	4	4	4	4
4	3	2	1	3	2	4	4
3	3	2	2	3	3	3	3
4	3	1	2	3	4	4	4
3	4	2	4	4	2	3	4
3	3	1	1	4	4	4	4
4	2	1	3	4	4	4	4
3	2	0	0	3	3	4	4
4	3	1	2	4	1	2	4
4	3	1	1	3	3	3	3
3	2	1	0	3	3	3	4
4	4	3	0	4	4	2	4
3	3	3	3	3	0	3	3
4	4	3	3	3	4	4	4
4	4	4	4	4	4	4	4
4	3	3	4	4	3	4	4
3	4	3	3	3	3	2	2
4	3	1	1	4	4	4	4
3	3	1	0	3	3	3	3
4	4	0	0	4	4	3	4
3	3	0	0	4	4	4	3
4	3	2	4	4	4	3	4
4	4	1	2	3	1	2	4
3	4	0	2	3	2	2	3
3	4	0	2	3	2	2	3
3	3	1	3	3	3	3	3
4	3	2	0	3	4	2	4
4	3	0	0	3	3	3	4
4	3	1	0	3	3	2	3
4	4	4	4	4	4	4	4
4	4	4	4	3	4	4	4
4	3	3	3	4	3	3	4
4	3	3	4	4	3	4	4
4	4	3	3	4	4	4	4
4	4	3	3	3	4	4	4
4	4	3	3	4	3	4	4
3	3	1	3	3	3	3	3
4	4	2	4	4	4	4	4
3	3	3	3	4	4	1	3
4	4	2	2	4	4	4	4
3	2	3	2	3	3	4	4
4	4	2	2	3	4	4	4
4	4	2	2	3	4	3	4
4	3	2	1	4	4	4	4

3	3	2	0	3	3	4	4
4	3	2	1	4	4	4	4
3	3	0	0	3	3	3	3
4	4	2	1	3	4	3	4
3	3	2	0	3	3	4	4
4	4	2	1	3	4	4	4
4	3	1	1	3	3	3	3
4	4	0	0	3	3	4	4
4	3	3	1	3	2	3	4
3	3	2	2	3	3	2	3
4	2	2	0	4	3	3	3
4	3	2	0	3	0	4	4
4	2	1	2	4	4	3	4
4	4	2	1	4	4	2	4
4	2	0	0	4	4	2	4
4	2	1	2	3	1	3	3
3	2	2	1	3	3	3	2
4	3	2	1	4	1	3	2
3	3	0	0	3	2	3	3

2	3	0	2	4	1	4	4
1	4	3	0	2	3	1	3
4	1	0	0	2	0	0	3
4	2	1	2	0	0	1	4
3	0	1	0	1	1	0	2
3	3	2	1	2	2	2	4
3	4	0	0	1	1	2	3
4	1	1	1	4	0	0	4
3	2	1	0	1	1	1	3
2	3	1	1	2	3	2	2
2	2	0	0	0	0	3	4
0	2	2	0	4	2	1	4
1	2	0	0	3	0	3	2
1	0	0	0	0	2	0	3

1	1	1	0	2	2	0	3
2	1	0	0	0	0	0	0
0	0	0	0	0	0	0	0
0	0	0	0	0	0	0	0
0	0	0	0	0	0	0	0
1	2	1	0	1	2	1	2
1	0	0	0	1	1	1	1
0	0	0	0	0	0	0	0
0	1	1	0	0	0	0	1
2	2	1	1	1	0	4	3
2	2	0	0	0	0	2	4
1	0	2	2	1	0	2	1
0	1	0	0	0	0	0	0
0	0	0	0	0	0	0	0
1	1	0	0	2	0	0	2
1	0	0	0	0	2	0	1
0	0	0	0	0	0	0	3
0	0	0	0	0	0	0	0
2	0	0	0	2	2	2	2
2	2	0	0	0	0	0	0
1	1	0	0	1	1	0	1
2	1	0	0	0	2	1	1
0	0	0	0	0	0	0	0
1	1	0	0	1	0	0	0
0	1	0	0	0	1	0	0
0	1	0	0	1	1	0	0
0	0	0	0	0	0	1	0
1	1	0	0	0	0	1	1
2	1	0	0	1	2	4	3
1	0	0	0	0	0	1	1
0	0	0	0	0	0	0	0
0	0	0	0	0	0	0	0
0	0	0	0	0	0	0	0
0	2	0	0	0	2	0	0
0	0	0	0	0	1	0	1
2	0	0	0	4	2	0	0
2	0	0	0	3	0	2	0
1	2	0	0	0	0	3	3
2	3	2	0	1	2	3	4
2	3	0	0	2	0	2	3
0	0	0	0	0	0	0	0
1	1	0	0	1	1	0	1
1	1	0	0	1	1	0	1

CFSQ PEM	QuadrantFa	Quad≥4/8 r	Quadrant	CIF Quad	Chalder	CISR	MDFI Gene
4	FatigueCFS	8	CFS	CFS	31	6	20
4	FatigueCFS	7	CFS	CFS	26	6	20
3	FatigueCFS	7	CFS	CFS	27	6	18
4	FatigueCFS	7	CFS	CFS	31	6	16
4	FatigueCFS	7	CFS	CFS	31	6	20
3	FatigueCFS	6	CFS	CFS		6	15
4	FatigueCFS	6	CFS	CFS	26	6	19
3	FatigueCFS	6	CFS	CFS	22	6	20
4	FatigueCFS	6	CFS	CFS	26	6	20
4	FatigueCFS	6	CFS	CFS	21	6	20
4	FatigueCFS	6	CFS	CFS	16	5	17
4	FatigueCFS	6	CFS	CFS	11	5	20
4	FatigueCFS	5	CFS	CFS	26	6	19
3	FatigueCFS	5	CFS	CFS	27	5	20
4	FatigueCFS	5	CFS	CFS	33	6	20
3	FatigueCFS	5	CFS	CFS	26	6	20
4	FatigueCFS	5	CFS	CFS	19	5	20
4	FatigueCFS	5	CFS	CFS	24	6	16
3	FatigueCFS	5	CFS	CFS	18	6	19
2	FatigueCFS	5	CFS	CFS	18	4	14
2	FatigueCFS	4	CFS	CFS			
0	FatigueCFS	4	CFS	CFS			
3	FatigueCFS	4	CFS	CFS	20	6	20
4	FatigueCFS	4	CFS	CFS	26	4	16
4	FatigueCFS	4	CFS	CFS			
4	FatigueCFS	4	CFS	CFS	24	6	
2	FatigueCFS	5	CFS	CFS	11	5	
4	FatigueCFS	7	CFS	CFS	33	6	
3	FatigueCFS	4	CFS	CFS	20	6	
4	FatigueCFS	4	CFS	CFS	16		
4	FatigueCFS	4	CFS	CFS	11	6	
4	FatigueCFS	4	CFS	CFS	24	6	
3	FatigueCFS	6	CFS	CFS	32	6	
4	FatigueCFS	6	CFS	CFS	28	6	
3	FatigueCFS	4	CFS	CFS	26	6	
3	FatigueCFS	4	CFS	CFS	30	6	
4	FatigueCFS	4	CFS	CFS	23	6	
4	FatigueCFS	8	CFS	CFS	31	6	
3	FatigueCFS	5	CFS	CFS	28	6	
4	FatigueCFS	4	CFS	CFS	25	6	
3	FatigueCFS	4	CFS	CFS	29	6	18
3	FatigueCFS	4	CFS	CFS	25	6	
3	FatigueCFS	7	CFS	CFS	24	6	16

3	FatigueCFS	5	CFS	CFS	22	5	
4	FatigueCFS	8	CFS	CFS	23	6	20
4	FatigueCFS	5	CFS	CFS	17	6	
4	FatigueCFS	6	CFS	CFS	26	6	20
4	FatigueCFS	5	CFS	CFS	23	6	
4	FatigueCFS	7	CFS	CFS	30	6	
4	FatigueCFS	5	CFS	CFS	30	6	
4	FatigueCFS	6	CFS	CFS	11	5	19
4	FatigueCFS	8	CFS	CFS			
4	FatigueCFS	7	CFS	CFS	24	6	20
3	FatigueCFS	6	CFS	CFS	22	6	15
4	FatigueCFS	6	CFS	CFS	21	6	
4	FatigueCFS	5	CFS	CFS	11	6	19
4	FatigueCFS	5	CFS	CFS	30		20
3	notFatigue	4	CFSLWIFS	CFSLWIFS	18	6	
3	FatigueCFS	2	CIF	CIF (CFS)	27	6	
2	FatigueCFS	3	CIF	CIF (CFS)	11	6	18
2	FatigueCFS	3	CIF	CIF (CFS)	28	6	16
2	FatigueCFS	2	CIF	CIF (CFS)	11	5	18
4	FatigueCFS	2	CIF	CIF (CFS)	23	6	19
4	FatigueCFS	2	CIF	CIF (CFS)	17	6	20
4	FatigueCFS	1	CIF	CIF (CFS)	23	6	15
3	FatigueCFS	3	CIF	CIF (CFS)	24	4	
3	FatigueCFS	3	CIF	CIF (CFS)	17	6	
4	FatigueCFS	3	CIF	CIF (CFS)	33	6	
4	FatigueCFS	3	CIF	CIF (CFS)	23	6	
4	FatigueCFS	2	CIF	CIF (CFS)	11	5	
4	FatigueCFS	2	CIF	CIF (CFS)	26	6	
3	FatigueCFS	3	CIF	CIF (CFS)	11	6	
4	FatigueCFS	3	CIF	CIF (CFS)	21	5	
4	FatigueCFS	3	CIF	CIF (CFS)	20	6	
4	FatigueCFS	3	CIF	CIF (CFS)	15	2	
3	FatigueCFS	3	CIF	CIF (CFS)	28	6	
4	FatigueCFS	3	CIF	CIF (CFS)	24	6	20
4	FatigueCFS	3	CIF	CIF (CFS)	24	6	

1	notFatigue	4	CFSLWIFS	CFSLWIFS	18	5	
0	notFatigue	4	CFSLWIFS	CFSLWIFS	22	6	
3	notFatigue	5	CFSLWIFS	CFSLWIFS	21	6	14
2	notFatigue	4	CFSLWIFS	CFSLWIFS	22	4	
1	FatigueCFS	2	CIF	CIF (GWI)	19	5	14

2	FatigueCFS	3	CIF	CIF (GWI)	19	2	
2	FatigueCFS	3	CIF	CIF (GWI)	24	6	19
1	FatigueCFS	2	CIF	CIF (GWI)	24	6	18
0	FatigueCFS	1	CIF	CIF (GWI)	0		5
2	FatigueCFS	2	CIF	CIF (GWI)	15	2	
3	FatigueCFS	3	CIF	CIF (GWI)	14	6	
4	FatigueCFS	3	CIF	CIF (GWI)	11	6	
2	FatigueCFS	2	CIF	CIF (GWI)	21	5	
4	FatigueCFS	3	CIF	CIF (GWI)	24	6	
2	FatigueCFS	3	CIF	CIF (GWI)	25	6	17
2	FatigueCFS	3	CIF	CIF (GWI)	24	6	18
2	FatigueCFS	3	CIF	CIF (GWI)	19	5	
2	FatigueCFS	3	CIF	CIF (GWI)	20	6	16
1	FatigueCFS	0	CIF	CIF (GWI)	24	6	18
2	FatigueCFS	3	CIF	CIF (GWI)	25	6	17
1	FatigueCFS	2	CIF	CIF (GWI)		6	20
1	FatigueCFS	2	CIF	CIF (GWI)	17	4	17
1	FatigueCFS	1	CIF	CIF (GWI)	19	6	18
4	FatigueCFS	8	CFS	GWI	26	5	
4	FatigueCFS	7	CFS	GWI	30	6	
4	FatigueCFS	5	CFS	GWI	25	6	
4	FatigueCFS	5	CFS	GWI	27	6	
3	FatigueCFS	4	CFS	GWI	30	6	
4	FatigueCFS	6	CFS	GWI	28	6	
4	FatigueCFS	5	CFS	GWI	29	6	
4	FatigueCFS	8	CFS	GWI	24	6	
3	FatigueCFS	6	CFS	GWI	31	6	
3	FatigueCFS	6	CFS	GWI	26	6	
3	FatigueCFS	6	CFS	GWI	19	6	
4	FatigueCFS	6	CFS	GWI	33	6	
4	FatigueCFS	6	CFS	GWI	30	6	20
3	FatigueCFS	7	CFS	GWI	21	6	
4	FatigueCFS	4	CFS	GWI	19	6	
4	FatigueCFS	6	CFS	GWI	25	6	
2	notFatigue	2	SC	GWI	18	5	
4	FatigueCFS	7	CFS	GWI	26	6	20
4	FatigueCFS	7	CFS	GWI	22	6	20
3	FatigueCFS	7	CFS	GWI	14	5	20
4	FatigueCFS	7	CFS	GWI	24	6	20
3	FatigueCFS	7	CFS	GWI	27	5	19
3	FatigueCFS	6	CFS	GWI	26	6	14
4	FatigueCFS	6	CFS	GWI	25	6	20
4	FatigueCFS	6	CFS	GWI	29	4	20
2	FatigueCFS	6	CFS	GWI	22	6	

4	FatigueCFS	6	CFS	GWI	27	6	12
4	FatigueCFS	6	CFS	GWI	30	6	19
4	FatigueCFS	5	CFS	GWI	17	6	20
3	FatigueCFS	5	CFS	GWI	20	6	18
4	FatigueCFS	5	CFS	GWI	16	6	18
4	FatigueCFS	5	CFS	GWI	28	6	20
3	FatigueCFS	5	CFS	GWI	29	6	18
4	FatigueCFS	5	CFS	GWI	29	6	20
3	FatigueCFS	4	CFS	GWI	19	4	19
4	FatigueCFS	4	CFS	GWI	21	6	19
2	FatigueCFS	4	CFS	GWI	24	6	9
2	notFatigue	2	SC	GWI	24	3	
4	FatigueCFS	6	CFS	GWI	30	6	
4	FatigueCFS	8	CFS	GWI	27	6	
4	FatigueCFS	4	CFS	GWI	24	6	
4	FatigueCFS	8	CFS	GWI	29	6	
3	FatigueCFS	7	CFS	GWI	30	6	
4	FatigueCFS	6	CFS	GWI	33	6	
4	FatigueCFS	5	CFS	GWI	25	6	
4	FatigueCFS	7	CFS	GWI	32	6	
4	FatigueCFS	7	CFS	GWI	33	6	
4	FatigueCFS	7	CFS	GWI	28	6	
4	FatigueCFS	6	CFS	GWI	32	6	
4	FatigueCFS	6	CFS	GWI	29	6	
4	FatigueCFS	6	CFS	GWI	30	6	
4	FatigueCFS	6	CFS	GWI	24	5	
3	FatigueCFS	5	CFS	GWI	23	6	15
4	FatigueCFS	5	CFS	GWI	21	6	
4	FatigueCFS	6	CFS	GWI	25	6	19
4	FatigueCFS	6	CFS	GWI	27	6	20
2	FatigueCFS	6	CFS	GWI	19	6	
4	FatigueCFS	6	CFS	GWI	28	6	19
4	FatigueCFS	5	CFS	GWI	25	6	20
2	FatigueCFS	5	CFS	GWI	23	6	
4	FatigueCFS	5	CFS	GWI	25	6	
4	FatigueCFS	6	CFS	GWI	26	6	15
3	FatigueCFS	6	CFS	GWI	31	6	17
3	FatigueCFS	4	CFS	GWI	20	6	18
4	FatigueCFS	8	CFS	GWI	32	6	
1	notFatigue	0	SC	GWI	26	6	
4	FatigueCFS	5	CFS	GWI	28	6	20
4	FatigueCFS	5	CFS	GWI	28	6	20
4	FatigueCFS	5	CFS	GWI	18	6	19
4	FatigueCFS	6	CFS	GWI	30	6	

3	FatigueCFS	7	CFS	GWI		24	6	
3	FatigueCFS	5	CFS	GWI		23	6	
3	FatigueCFS	6	CFS	GWI		19	6	17
4	FatigueCFS	6	CFS	GWI		31	6	20
4	FatigueCFS	6	CFS	GWI		27	4	18
3	FatigueCFS	6	CFS	GWI		18	5	11
4	FatigueCFS	6	CFS	GWI		16	6	18
4	FatigueCFS	5	CFS	GWI				
3	FatigueCFS	4	CFS	GWI		27	5	12
4	FatigueCFS	6	CFS	GWI		22	6	18
4	FatigueCFS	5	CFS	GWI		24	6	
4	FatigueCFS	6	CFS	GWI		29	6	
0	FatigueCFS	6	CFS	GWI		21	6	15
4	FatigueCFS	8	CFS	GWI		30	6	20
4	FatigueCFS	8	CFS	GWI		27	6	20
4	FatigueCFS	8	CFS	GWI		18	6	16
4	FatigueCFS	6	CFS	GWI		32	4	20
4	FatigueCFS	6	CFS	GWI		29	6	20
3	FatigueCFS	6	CFS	GWI		21	6	20
3	FatigueCFS	6	CFS	GWI		25	6	18
3	FatigueCFS	6	CFS	GWI		24	6	
4	FatigueCFS	7	CFS	GWI		32	6	19
4	FatigueCFS	4	CFS	GWI		30	6	20
3	FatigueCFS	4	CFS	GWI		26	6	18
3	FatigueCFS	4	CFS	GWI		26	6	18
3	FatigueCFS	7	CFS	GWI		25	6	20
2	FatigueCFS	4	CFS	GWI		19	6	16
4	FatigueCFS	6	CFS	GWI		25	5	
3	FatigueCFS	5	CFS	GWI				
4	FatigueCFS	8	CFS	GWI		27	6	20
4	FatigueCFS	8	CFS	GWI		22	6	20
4	FatigueCFS	8	CFS	GWI		16	6	18
4	FatigueCFS	8	CFS	GWI		18	6	16
4	FatigueCFS	8	CFS	GWI		30	6	20
4	FatigueCFS	8	CFS	GWI		29	6	20
4	FatigueCFS	8	CFS	GWI		30	6	
3	FatigueCFS	7	CFS	GWI		25	6	20
4	FatigueCFS	7	CFS	GWI		33	6	20
3	FatigueCFS	7	CFS	GWI		27	5	19
4	FatigueCFS	6	CFS	GWI		27	6	18
4	FatigueCFS	6	CFS	GWI		16	6	20
4	FatigueCFS	6	CFS	GWI		28	5	
4	FatigueCFS	6	CFS	GWI		31	6	20
4	FatigueCFS	6	CFS	GWI		29	4	12

3	FatigueCFS	6	CFS	GWI	20	5	20
4	FatigueCFS	6	CFS	GWI	29	6	19
3	FatigueCFS	6	CFS	GWI	30	6	16
4	FatigueCFS	6	CFS	GWI	25	6	19
4	FatigueCFS	6	CFS	GWI	11	6	20
4	FatigueCFS	6	CFS	GWI	30	6	20
4	FatigueCFS	6	CFS	GWI	22	6	18
4	FatigueCFS	6	CFS	GWI	31	6	17
4	FatigueCFS	6	CFS	GWI	27	6	8
3	FatigueCFS	5	CFS	GWI	29	6	18
4	FatigueCFS	5	CFS	GWI	24	6	9
4	FatigueCFS	5	CFS	GWI	25	6	20
4	FatigueCFS	5	CFS	GWI	26	2	18
4	FatigueCFS	5	CFS	GWI	29	6	20
4	FatigueCFS	4	CFS	GWI	25	6	20
4	FatigueCFS	4	CFS	GWI	27	6	
3	FatigueCFS	4	CFS	GWI	9	4	14
4	FatigueCFS	4	CFS	GWI	23	6	19
2	FatigueCFS	4	CFS	GWI	24	6	18

4	notFatigue	5	CFSLWIFS	CFSLWIFS	24	6	19
1	notFatigue	4	CFSLWIFS	CFSLWIFS	14	2	8
4	FatigueCFS	2	CIF	CIF (SC)	25	6	16
4	FatigueCFS	2	CIF	CIF (SC)	19	6	18
1	FatigueCFS	0	CIF	CIF (SC)	11	0	7
4	FatigueCFS	3	CIF	CIF (SC)	24	6	
3	FatigueCFS	3	CIF	CIF (SC)	12	6	
2	FatigueCFS	2	CIF	CIF (SC)	20	4	
3	FatigueCFS	2	CIF	CIF (SC)	24	6	
3	notFatigue	3	SC	SC	18	6	16
0	notFatigue	2	SC	SC	16	5	
4	notFatigue	3	SC	SC	17	6	10
0	notFatigue	2	SC	SC	11	0	9
2	notFatigue	1	SC	SC	14	2	14

1	notFatigue	1	SC	SC	15	1	8
1	notFatigue	0	SC	SC	9	0	6
0	notFatigue	0	SC	SC	11	0	5
0	notFatigue	0	SC	SC	11	0	5
0	notFatigue	0	SC	SC	12	0	9
0	notFatigue	0	SC	SC	11	0	8
0	notFatigue	0	SC	SC	11	0	9
0	notFatigue	0	SC	SC	11	0	4
0	notFatigue	0	SC	SC	16	4	
2	notFatigue	2	SC	SC	22	6	
2	notFatigue	1	SC	SC	28	6	
0	notFatigue	0	SC	SC	12	0	
0	notFatigue	0	SC	SC	12	0	
0	notFatigue	0	SC	SC	11	0	
0	notFatigue	0	SC	SC	8	0	
0	notFatigue	0	SC	SC	5	0	
0	notFatigue	1	SC	SC	11	0	
0	notFatigue	0	SC	SC	5	0	
0	notFatigue	0	SC	SC	13	3	
0	notFatigue	0	SC	SC	11	2	
0	notFatigue	0	SC	SC	11	0	6
0	notFatigue	0	SC	SC	11	0	
0	notFatigue	0	SC	SC	11	0	
0	notFatigue	0	SC	SC	11	0	7
0	notFatigue	0	SC	SC	11	0	4
0	notFatigue	0	SC	SC	11	0	5
0	notFatigue	0	SC	SC	8	0	
0	notFatigue	0	SC	SC		2	9
1	notFatigue	2	SC	SC	14	3	14
0	notFatigue	0	SC	SC	9	0	9
0	notFatigue	0	SC	SC		0	4
0	notFatigue	0	SC	SC	9	0	
0	notFatigue	0	SC	SC	0	0	4
0	notFatigue	0	SC	SC	11	0	
0	notFatigue	0	SC	SC	11	0	
3	notFatigue	2	SC	SC	16	2	8
1	notFatigue	1	SC	SC	19	5	18
0	notFatigue	2	SC	SC	13	3	
2	notFatigue	3	SC	SC	27	6	18
0	notFatigue	2	SC	SC	12	4	9
0	notFatigue	0	SC	SC	1	0	5
0	notFatigue	0	SC	SC	10	0	10
0	notFatigue	0	SC	SC	12	1	10

12 2

MDFI Physi	MDFI Redu	MDFI Redu	MDFI Menta	MDFI ΣDom	Epworth	Widesprea	Tender Poir	Tender Poir
16	20	10	17	83		1	1	16.5
16	20	15	16	87		1	1	15
18	19	18	20	93		1	0	4
20	17	5	19	77		0	0	0
18	18	15	19	90		0	0	0
16	15	9	9	64		1	0	9.5
20	15	13	11	78		1	1	17
18	17	13	18	86		1	1	18
19	20	15	20	94		1	0	1
13	14	8	20	75		1	0	2
12	14	13	10	66		0	0	10.5
13	20	19	13	85		1	1	17
20	19	16	17	91		1	1	16
18	15	15	20	88		1	1	17
20	20	14	13	87		1	0	8
20	10	11	14	75		1	0	6.5
19	16	8	12	75		0	0	5
16	17	13	14	76		1	1	13
20	17	16	13	85		1	1	9
10	17	7	19	67		1	1	15.5
							1	18
							1	18
20	20	20	20	100		0	0	8.5
12	17	15	15	75		0	0	
						1	0	6
					7	0	0	6.7
					10	1	1	11.3
						1	0	16.0
					3	1	1	13.7
					11	1	1	12.5
					7	0	0	9.7
					0	0	0	0
					11	1	1	15.0
					7	1	0	6.3
					9	1	0	5.3
					21	1	0	3.0
					17	1	1	0.7
					13	0	1	15
					10	1	1	10.3
					4	0	0	7
19	17	5	11	70		0	0	
					5	0	0	2.25
15	20	14	19	84		0	1	14

					5	1	1	14
12	17	11	16	76		1	1	18
					7	1	0	4.0
20	20	12	12	84		1	0	0
					18	1	1	17.0
						1	1	10.0
					13	1	1	12
16	17	14	20	86		0	1	15
								17
13	18	16	16	83		0	0	0
15	14	10	9	63		0	1	11.5
					4	0	0	7.5
18	20	10	15	82		1	1	17.5
20	20	17	17	94		1	1	13.7
					1	1	1	6.0
					8	0	0	1.67
17	12	13	18	78		0	0	0
10	12	4	14	56			0	
15	15	11	9	68		1	1	11.5
19	17	7	10	72		0	0	4
17	10	10	14	71		1	1	15.5
20	19	10	4	68		1	0	9
					6	1	1	13.0
					6	0	0	9.3
					1	0	0	
					7	0	0	0
					11	1	0	8.3
					9	0	0	1.3
					10	0	1	13.5
					5	0	0	0.3
					15	1	0	7
							1	7.5
					7	0	0	0.3
20	20	19	14	93		0	0	2
					6	0	0	6
							1	13.5
							1	14
							0	2
							0	1
					19	1	1	15.5
					15	1	1	18
14	11	14	14	67		1	0	4
					12	1	1	4.33
9	16	17	15	71		1	0	

					4	0	0	0
15	16	12	15	77		1	0	4
16	18	15	15	82		1	0	0.666667
4	4	6	7	26		0	0	6
					6	0	0	0
					7	1	0	3.33
					8	1	0	1.0
					12	1	1	12
					9	1	0	4
12	13	13	14	69		1	0	0
14	15	17	13	77		1	1	16
					7	1	1	10.33
11	14	15	17	73		1	0	9
18	16	15	10	77		0	0	6
12	13	13	14	69			0	0
20	20	12	16	88		1	1	13
15	15	18	11	76		0	0	6
14	14	14	10	70		1	1	13
					15	1	1	18
					6	1	1	16.5
					1	1	0	9
					13	0	0	1.75
					16	1	0	6
					21	1	1	18
					24	1	0	4
					11	1	1	18
					18	1	1	17.5
					12	0	1	13.25
					17	1	1	9.33
					7	1	1	16.5
20	15	15	20	90		1	0	8
					21	1	1	16
					7	1	0	3.5
					3	1	1	8
					9	0	1	10
20	20	12	20	92		1	1	17
20	16	17	19	92		1	1	18
18	20	17	19	94		0	1	13.25
20	19	12	20	91		1	1	15.5
17	18	12	19	85		1	0	0.5
12	11	9	16	62		1	1	18
16	16	8	14	74		1	1	16.25
19	15	10	18	82		1	1	
					11	1	1	10

11	14	9	8	54		0	0	12.25
20	20	16	20	95		0	0	2
16	9	6	9	60		0	1	16
17	16	15	12	78		1	1	17.5
13	9	9	13	62		1	1	16.25
18	17	14	15	84		1	1	14.25
16	17	15	16	82		1	1	16
16	20	18	13	87		1	0	3.25
13	15	10	4	61		1	1	14
13	19	7	17	75		0	0	
14	13	12	12	60		1	0	14.25
					9	0	0	1
					15	1	1	18
					10	0	0	0
					9	1	1	14
					16	1	0	18
					21	1	1	17
					6	0	1	17.75
					19	1	1	12
					8	1	1	12
					23	1	0	5.5
					9	1	0	1.0
					16	1	0	10.0
					19	1	1	12.3
					14	1	1	15.0
					2	1	0	10.0
13	16	12	14	70		1	1	17
					12	1	0	7.0
16	19	15	15	84			1	16
20	20	20	14	94		1	0	1
							0	0
15	18	16	17	85		0	1	12.75
16	19	16	20	91			1	17.75
					15	1	1	14
					14	1	0	8.7
16	15	12	15	73		1	1	15
14	13	10	16	70		1	0	7.25
13	20	11	18	80		1	1	15.75
					19	1	1	11.7
					14	1	0	6.5
17	18	13	15	83			1	12
17	18	13	15	83		1	1	14.5
17	18	15	17	86		1	1	14
					22	1	0	4

					15	1	1	16.3
					13	0	0	2
16	15	14	19	81		1	1	12.66667
20	18	18	19	95		1	1	18
20	12	7	16	73		1	1	
15	13	13	14	66		1	0	10.5
19	18	14	18	87		1	1	16.75
							0	4.75
12	11	14	14	63			1	16.75
17	18	11	11	75		1	1	16
					7	1	1	18
					14	1	0	0
7	7	9	14	52		0		
18	20	16	16	90		1	1	16
20	20	20	20	100		1	1	18
14	16	10	12	68		1	1	18
20	20	20	20	100		1	1	17
16	18	16	20	90		1	1	13
19	20	12	20	91		1	1	18
13	14	14	20	79		1	0	1
					3	1	0	0
20	18	18	16	91		1	1	3
20	20	15	15	90		1	1	17.5
13	15	14	16	76		0	1	13
13	15	14	16	76		1	1	13
19	20	15	20	94		1	1	16
14	13	17	16	76		1	0	3
					2	1	0	3
							1	15
20	20	20	20	100			1	18
16	20	20	16	92		1	1	18
19	20	11	13	81		1	1	15
18	12	14	12	72			1	17
15	6	10	14	65		1	1	17.75
20	20	20	20	100		1	1	16
							0	4
19	20	15	20	94			1	13
20	20	20	20	100		1	1	18
17	18	12	19	85		0	0	0
12	17	11	18	76		1	1	16
10	9	12	13	64		1	1	16
					19	1	0	7
20	20	20	20	100		1	1	16
13	9	6	10	50			1	18

17	18	18	11	84		1	1	18
15	15	12	15	76		0	1	15
14	14	10	14	68		1	0	7
16	19	15	15	84		1	0	13.25
16	18	13	14	81		1	0	5
18	20	16	16	90			1	14
17	18	11	11	75			1	16
14	13	10	16	70			0	9
6	7	10	12	43		0	0	9
16	17	15	16	82			1	18
14	13	12	12	60			0	4
16	19	16	20	91			1	18
18	14	16	16	82		1	0	7
16	20	18	13	87			0	4
16	16	8	14	74			1	11
							1	13
11	9	7	10	51		1	0	8
20	19	14	10	82		1	1	15
18	16	15	10	77			0	9
							1	18
							1	18
							1	18
							1	18
							0	2
							1	18
							0	3
							1	14
							0	4
							0	1
							0	5
14	8	6	16	63		1	1	18
7	5	10	4	34		0	0	9
15	16	13	13	73		1		
14	15	5	10	62		0	1	13.5
4	9	12	4	36		0	0	0
					5	1	0	3.0
					5	0	0	4.0
					8	0	0	1.0
					11	1		
11	16	14	16	73		1	0	9
					2	0	1	7
18	11	9	10	58		1	0	10
7	4	5	5	30		1	0	0
14	12	10	9	59		0	0	0

6	5	6	5	30		0	0	1
6	6	7	10	35		0	0	0
6	4	4	8	27		0	0	0
4	4	4	4	21		0	0	0
6	6	9	14	44		0	0	0
6	7	10	12	43			0	0
5	8	9	5	36		0	0	1
4	4	6	11	29		0	0	0
					15	0	0	0
					6	0	0	0.3
					20	0	0	0
					3	0	0	0
					3	0	0	6.7
					9	0	0	1.3
					4	0	0	5.0
					10	0	0	0
					1	0	0	1.0
					2	0	0	3
					15	0	0	0
					7	0	0	0
8	5	7	6	32		0	0	2.333333
					11	0	0	0.7
					8	0	0	1.7
5	5	6	4	27		0	0	7
4	4	4	8	24		0	0	4.5
5	4	5	4	23			1	11
					7	0	0	3.5
11	10	6	14	50		0	0	0
11	9	10	11	55		0	0	10
4	4	11	8	36		0	0	
4	4	4	4	20		0	0	0
					6	0	0	0
4	6	7	7	28		0	0	0.5
					2	0	0	0
					8	0	0	1.5
20	8	8	8	52		1	1	12
15	15	15	18	81		1	0	10
					2	0	0	0
18	17	11	18	82		0	0	0
6	4	4	13	36		0	0	2.5
5	6	7	6	29		0	0	0
5	6	4	8	33			0	0.333333
4	4	4	7	29		0	0	0
							1	18

1	0	0	0
		0	0
	0	0	2.7
	0	0	0.5
		0	0

Dolorimetr	FM_1990	FM_2010	FM_2011	McGill Tota	McGill Sens	McGill Affe	sf36_pf	sf36_rp
0.66	1			17	13	4	35	0
1.27	1			27	24	3	10	0
1.39	0			33	24	9	20	0
2.88	0			4	4	0	30	0
2.90	0			21	16	5	0	0
1.49	0			15	11	4	30	0
1.67	1			27	25	2	10	0
1.97	0			20	17	3	50	0
2.32	0			12	6	6	55	0
2.44	0			39	28	11	35	0
4.22	0			6	6	0	60	0
4.44	1			11	9	2	25	0
1.19	1			29	23	6	50	0
1.27	1			21	15	6	30	0
1.76	0			26	14	12	35	0
1.93	0			13	11	2	65	0
2.10	0			16	16	0	45	0
2.80	1			27	23	4	55	25
2.82	1			16	13	3	50	25
3.09	1			19	15	4	55	0
0.81	1							
1.48	1							
2.11	0			8	8	0	50	25
4.66	0			12	8	4	70	0
5.50	0							
4.19	0	0	0	14	11	3	75	0
3.28	0	0	0	29	25	4	75	0
1.20	0	1	1	30	22	8	0	0
1.73	1		0	15	13	2	85	50
2.24	1	1	1				25	0
3.13	0	1	1	2	2	0	30	0
5.25	0		0	3	2	1	30	0
2.06	1	1	1	20	18	2	20	0
4.38	0	1	1	30	27	3	45	0
3.57	0	1	1	15	11	4	65	0
4.37	0	1	1	13	11	2	55	0
5.65	0	1	1	12	10	2	40	0
2.03	1	1	1	38	28	10	25	0
3.40	1	1	1	19	17	2	65	0
4.42	0	0	0	4	3	1	55	0
4.75	0			3	3	0	20	0
4.05	0	0	0	2	2	0	60	0
2.45	0			19	16	3	35	0

3.19	0	1	1	13	10	3	75	50
0.80	1			21	12	9	25	0
4.03	0	1	1	16	13	3	25	0
3.04	0			29	23	6	10	0
1.99	1	1	1	21	18	3	25	0
2.12	1	1	1				15	0
0.53	1	1	1	27	23	4	0	0
2.53	1			15	12	3	50	100
2.35	1							
5.45	0			15	10	5	50	0
2.09	1			12	10	2	65	0
2.91	0	0	0	12	12	0	25	0
3.23	1			22	19	3	0	50
3.63	1			17	14	3	25	0
1.96	1	1	1	19	11	8	20	0
4.00	0	0	0	11	8	3	95	25
2.74	0			7	5	2	70	25
3.23	0			0	0	0	85	0
1.47	0			22	16	6	60	0
3.36	0			5	5	0	30	0
3.76	0			20	18	2	40	0
2.93	0			6	3	3	45	0
2.59	1	1	1	38	27	11	20	0
2.80	0	0	0	12	11	1	55	0
4.45	0	1		5	3	2	35	0
4.92	0	0	0	0	0	0	40	0
3.50	0	1	1	16	13	3	80	0
3.54	0	0	0	0	0	0	40	0
2.00	0	0	0				50	0
7.49	0	0	0	2	0	2	35	0
4.68	0	1	1	17	14	3	15	0
5.30	0	1	1				85	100
5.75	0	0	0	3	1	2	65	0
4.89	0			19	12	7	30	0
6.00	0	0	0	0	0	0	70	25
1.80	1							
2.23	1							
7.41	0							
7.86	0	1	1					
2.45	1	1	1	9	9	0	80	75
0.95	1	1	1	22	18	4	40	0
7.89	0	1		23	20	3	40	25
4.84	0	1	0	20	20	0	50	50
4.29	0			10	9	1	85	0

8.01	0	0	0	5	4	1	95	25
5.65	0			10	7	3	50	0
5.71	0			10	6	4	80	50
6.48	0			0	0	0	100	50
5.58	0	0	0	9	8	1	100	100
5.88	0	1	0	7	4	3	50	25
5.04	0	0	0	5	4	1	45	0
2.67	0	1	1	19	16	3	50	25
3.80	0	1	1	9	8	1	5	0
6.91	0			21	14	7	90	50
3.72	1			20	17	3	65	25
2.20	0	1	1	20	19	1	50	0
6.98				16	16	0	90	50
5.91	0			11	10	1	80	50
7.57				21	14	7	90	50
4.19	1						20	0
4.41				8	7	1	85	50
5.08				16	14	2	85	50
0.90	1	1	1	25	18	7	30	0
1.71	1	1	1	20	17	3	35	0
2.06	1	1	1	35	23	12	65	0
5.09	0	0	0	24	17	7	30	0
6.46	0	1	1	23	19	4	40	0
0.86	1	1	1	19	18	1	70	100
5.74	0	1	1	26	18	8	80	0
1.01	1	1	1	34	28	6	35	0
1.07	1	1	1	37	28	9	90	0
3.78	1	1	1	24	19	5	40	0
6.06	0	1	1	23	18	5	65	0
2.05	1	1	1	42	30	12	30	0
4.51				25	17	8	45	0
1.05	1	1	1	30	23	7	35	25
5.76	0	1	1	26	23	3	55	0
3.72	1	1	1	25	19	6	30	0
3.24	0	1	0				35	0
0.19	1			38	28	10	10	0
0.46	1			34	28	6	5	0
3.71	0			10	4	6	40	0
3.98				24	17	7	35	0
8.39	0			17	11	6	40	0
0.83	1			17	13	4	25	0
1.47	1			30	24	6	55	75
2.90	1			35	27	8	65	0
3.36	0	1	1	34	24	10	60	0

4.33	0			20	12	8	40	0
7.44	0			20	17	3	20	25
1.16	0			22	17	5	35	25
2.48	1			21	15	6	20	0
2.55	1			20	17	3	60	0
3.08	1			22	18	4	25	0
3.29	1			22	17	5	75	0
6.74	0			29	19	10	70	0
1.17	1			17	13	4	45	0
2.00	0			6	6	0	65	25
2.41	0			22	19	3	40	0
6.68	0	0	0	6	6	0	95	50
0.69	1	1	1	38	29	9	15	0
6.60	0	1	1	23	18	5	50	0
2.95	1	1	1	22	15	7	45	0
4.46	0	1	1	33	22	11	35	0
2.50	1	1	1	35	29	6	60	0
0.73	1	1	1	19	13	6	50	0
2.78	1	1	1	23	17	6	70	0
2.77	1	1	1	23	17	6	15	0
4.43	0	1	0	9	6	3	40	0
5.55	0	1	0	27	20	7	30	0
1.87	0	1	0	37	28	9	30	0
2.27	0			24	17	7	25	25
2.80	1	1	1	27	18	9	5	0
3.87	0	1	0	20	17	3	20	0
2.29	1			19	16	3	35	0
2.61	0	1	1	20	13	7	30	0
2.52				31	24	7	30	0
6.87				36	24	12	60	0
8.24				19	16	3	45	0
3.82	0			26	17	9	95	0
3.73	0			21	17	4	20	0
4.31	1	1	1	20	16	4	70	0
4.80	0	1	1	27	22	5	30	0
2.05				24	17	7	20	0
3.90	0			23	20	3	80	0
1.21	1			31	23	8	50	0
3.20	1	1	1	33	26	7	5	0
5.01	0	0	0	27	22	5	50	0
1.70				27	21	6	20	0
1.75	1			27	21	6	20	0
3.23				24	19	5	10	0
4.62	0	1	1	28	22	6	30	0

1.07	1	1	0	26	18	8	60	0
2.70	0	1	1	25	22	3	60	0
2.75	1			26	18	8	55	100
1.83				39	28	11	65	0
2.09	1			24	19	5	65	0
2.19	0			27	22	5	35	0
2.54	0			37	25	12	40	0
4.86	0							
3.17				22	18	4	20	0
3.82	1			32	24	8	70	0
0.92	1	1	1	28	21	7	55	0
4.63	0	1	1	37	25	12	10	0
5.21	0			0	0	0	90	75
2.93	1			31	21	10	10	0
0.47	1			29	22	7	5	0
0.76	1			24	18	6	15	0
2.80	1			17	14	3		
3.37	1			41	29	12	65	0
1.11	1			23	15	8	10	0
7.28	0			25	20	5	75	0
9.58	0	1	1	19	14	5	35	0
5.68	0			24	13	11	20	0
2.30	1			27	21	6	15	0
3.84	0			10	7	3	55	0
5.26				10	7	3	55	0
0.88	1			38	28	10	30	0
7.29				23	16	7	75	25
3.50	0	1	1	10	8	2	70	0
2.03	1							
0.74				29	22	7	5	0
0.74				42	31	11	5	0
1.14	1			22	17	5	15	0
1.39				24	18	6	15	0
2.04	1			41	29	12	35	0
4.11				33	24	9	5	0
6.93	0						15	0
1.26				38	28	10	30	0
1.40	1			36	24	12	0	0
8.60				17	11	6	40	0
0.91	1			45	33	12	30	0
1.30	1			18	15	3	30	50
2.23	0	1	1	29	21	8	55	0
2.39				30	21	9	0	0
2.49				35	27	8	65	0

2.57				18	14	4	45	25
2.76	0			35	27	8	60	0
3.18				9	7	2	80	0
3.30	0			31	24	7	30	0
3.63				13	11	2	35	25
3.64				31	21	10	10	0
4.67				32	24	8	70	0
5.10				23	20	3	80	0
5.77				20	12	8	40	0
1.76				22	17	5	75	0
2.11				22	19	3	40	0
3.73				21	17	4	20	0
3.78	0			30	24	6	30	0
7.52				29	19	10	70	0
1.39				30	24	6	55	75
2.09				31.5	23.5	8	75	0
2.77				14	12	2	90	50
3.23				26	17	9	65	0
7.68				11	10	1	80	50
0.12								
1.04								
1.12								
2.53								
2.84								
2.92								
3.20	0	1	1					
3.48								
4.13								
7.93								
8.47								
0.65	1			23	17	6	80	25
1.72	0			3	2	1	95	100
2.07				19	14	5	30	0
4.84	0			0	0	0	55	0
2.29	0			0	0	0	95	100
3.48	0	0	0	9	9	0	50	0
6.48	0	0	0	0	0	0	75	0
7.34	0	0	0	16	13	3	90	0
4.60	0	0	0	6	6	0	90	25
3.64	0			18	17	1	85	0
6.10	0	0	0	0	0	0	85	100
2.07	0			17	15	2	55	0
2.08	0			17	15	2	85	0
8.42	0			3	3	0	100	100

9.29	0			4	4	0	95	75
2.93	0			1	0	1	100	100
3.59	0			1	1	0	95	100
4.87	0			0	0	0	100	100
6.20	0			0	0	0	100	100
8.67	0			9	9	0	85	100
9.00	0			3	2	1	100	100
9.17	0			0	0	0	100	100
6.52	0	0	0	0	0	0	15	100
4.56	0	0	0	11	10	1	75	0
3.52	0	0	0	0	0	0	85	0
3.04	0	0	0	0	0	0	100	100
5.22	0	0	0	0	0	0	100	100
6.20	0	0	0	0	0	0	100	100
6.73	0	0	0	8	8	0	50	100
8.29	0	0	0	1	1	0	100	100
8.26	0	0	0	0	0	0	90	100
4.80	0	0	0	0	0	0	100	100
8.08	0	0	0	0	0	0	100	100
8.80	0	0	0	3	2	1	100	100
4.56	0			2	2	0	20	100
6.97	0	0	0	7	5	2	90	100
5.39	0	0	0	0	0	0	95	100
3.29	0			0	0	0	90	100
4.23	0			0	0	0	100	100
4.32				0	0	0	100	100
7.35	0	0	0	0	0	0	100	100
8.38	0			2	2	0	100	75
5.03	0			11	10	1	70	50
8.83	0			0	0	0	100	100
7.29	0			0	0	0	100	100
3.82	0	0	0	0	0	0	100	100
8.36	0			0	0	0	100	100
6.79	0	0	0	2	2	0	100	100
7.20	0	0	0	2	2	0	100	100
2.43	0			26	22	4	25	0
5.86				13	11	2	85	100
7.52	0	0	0	7	6	1	95	100
5.59	0			8	8	0	95	0
7.95	0			1	1	0	85	75
3.70	0			1	1	0	100	100
8.32	0			0	0	0	100	100
8.40	0			0	0	0	100	100
3.22								

5.18	0	0	0	3	3	0	100	100
6.95	0							
7.30	0	0	0					
8.19	0	0	0					
8.40	0							

sf36_bp	sf36_gh	sf36_v	sf36_sf	sf36_re	sf36_mh	SF10	SF V RP SF	CMSI Rheu
22.5	15	0	12.5	100	88	2.5	4	23
10	30	0	0	100	60	0	0	31
12.5	55	10	25	100	72	7	12	29
45	70	0	37.5	100	92	7.5	13	18
0	10	10	12.5	0	40	4.5	8	19
32.5	50	30	62.5	66.66667	72	20.5	31	
22.5	15	0	0	100	92	0	0	21
22.5	45	20	37.5	0	60	18.5	19	15
0	15	0	0	100	68	5	0	19
22.5	45	10	25	0	36	9	12	24
45	50	20	50	100	60	23	23	11
45	65	5	12.5	0	48	4.5	6	17
22.5	10	0	25	0	36	5	8	22
45	30	10	25	33.33333	44	14	12	20
20	15	15	12.5	0	52	6.5	9	24
57.5	15	5	25	100	60	12	10	13
32.5	10	0	0	100	76	0	0	28
22.5	55	30	25	100	88	26	27	16
57.5	30	0	0	66.66667	48	10	8	
22.5	30	35	37.5	33.33333	48	17.5	24	19
80	65	35	12.5	100	84	24.5	24	15
67.5	65	20	37.5	33.33333	48	23.5	19	29
45	25	5	25	0	36	17	10	13
45	50	55	62.5	33.33333	60	35.5	39	16
0	10	5	0	100	92	2	2	29
57.5	60	30	62.5	100	84	52.5	48	12
20	35	10	25	0	68	7	12	17
45	95	20	25	100	92	16	15	14
77.5	20	15	12.5	100	68	6.5	9	11
32.5	35	0	12.5	100	88	2.5	4	0
20	5	20	0	0	36	6	7	19
32.5	40	35	75	100	56	30	37	11
67.5	35	25	0	0	52	13	8	23
67.5	35	0	25	100	76	5	8	19
10	25	5	0	0	36	2	2	35
45	30	25	50	100	68	23	25	15
80	45	45	62.5	100	92	28.5	36	6
45	10	15	12.5	100	92	4.5	9	12
42.5	30	5	0	0	40	7	2	7
32.5	35	0	75	100	84	20	25	22

57.5	5	30	75	100	52	53	52	15
22.5	20	15	25	0	68	11	13	27
45	15	0	0	100	80	0	0	11
0	20	0	0	100	56	0	0	31
22.5	15	5	25	0	52	7	10	21
10	10	0	12.5	100	80	2.5	4	34
0	10	0	0	66.66667	64	0	0	17
22.5	40	30	37.5	100	40	62.5	56	16
22.5	30	20	25	0	52	13	15	21
45	30	15	37.5	100	68	18.5	18	24
45	35	45	25	66.66667	68	19	23	20
57.5	35	20	62.5	33.33333	68	38.5	44	31
10	5	0	0	0	76	0	0	
32.5	10	30	25	100	84	15	18	10
32.5	45	15	62.5	100	64	32.5	34	12
70	15	0	0	100	64	15	8	4
22.5	45	0	25	100	100	10	8	9
35	75	35	50	0	68	27	28	10
67.5	30	15	0	33.33333	44	4	5	9
45	30	10	25	0	48	12	12	13
32.5	35	20	25	100	60	13	15	19
45	55	30	12.5	100	76	12.5	14	18
57.5	30	20	37.5	100	64	13.5	19	22
67.5	20	0	25	100	84	5	8	18
100	75	25	25	100	80	13	17	7
45	80	35	50	0	76	25	28	9
90	35	10	25	100	76	14	12	11
45	40	20	37.5	100	80	13.5	19	11
90	15	30	37.5	100	60	17.5	23	10
22.5	20	0	25	100	88	5	8	21
100	15	45	100	100	48	82	82	
67.5	20	15	25	0	52	16	13	13
20	40	10	25	0	52	9	12	14
100	35	20	62.5	100	60	33.5	36	8
45	35	45	75	33.33333	60	69	65	17
32.5	25	30	37.5	0	52	29.5	23	
22.5	25	30	50	66.66667	68	35	35	26
35	20	55	75	0	84	58	60	19
67.5	35	35	25	0	36	22	20	23

67.5	60	35	62.5	100	84	46.5	41	13
22.5	25	10	37.5	0	40	16.5	16	18
45	20	25	37.5	33.33333	60	40.5	38	12
80	75	50	75	100	72	61	58	7
80	75	50	87.5	100	92	81.5	79	13
45	45	35	62.5	0	88	35.5	41	12
67.5	35	15	50	0	60	16	22	8
45	55	40	37.5	33.33333	64	34.5	34	13
45	0	0	0	66.66667	84	0	0	14
57.5	40	15	75	33.33333	44	49	47	16
35	35	15	0	33.33333	32	21	13	26
45	35	35	75	33.33333	80	32	37	11
35	15	10	62.5	0	52	44.5	41	0
57.5	35	0	25	100	68	35	25	12
57.5	40	15	75	33.33333	44	49	47	16
100	45	30	62.5	0	76	27.5	31	0
35	30	25	25	66.66667	48	36	33	13
57.5	40	5	25	66.66667	44	35	27	16
22.5	10	35	37.5	33.33333	84	22.5	24	33
20	5	5	12.5	0	44	4.5	6	20
10	15	15	0	0	32	9	5	18
45	15	0	25	100	52	10	8	18
32.5	35	10	0	0	60	2	3	20
35	40	0	25	33.33333	40	50	42	28
22.5	50	15	50	0	52	19	22	20
22.5	15	25	25	33.33333	68	13	17	20
12.5	10	30	25	33.33333	20	19	18	37
22.5	10	0	25	0	52	10	8	21
57.5	25	10	62.5	0	52	21.5	24	28
0	5	5	0	0	12	5	2	26
12.5	0	0	0	0	56	0	0	33
32.5	35	20	75	33.33333	36	36	40	26
45	20	25	50	100	80	25	25	23
10	25	0	12.5	100	60	2.5	4	12
67.5	55	40	62.5	66.66667	68	29.5	34	19
12.5	0	45	12.5	0	60	18.5	19	36
22.5	20	10	12.5	0	32	4.5	8	35
45	35	35	25	0	40	24	20	14
22.5	10	10	12.5	0	48	11.5	8	17
22.5	15	35	37.5	0	36	24.5	24	18
45	30	30	50	100	80	18	27	23
22.5	50	10	12.5	100	80	39.5	33	22
20	20	20	62.5	0	68	23.5	28	22
22.5	10	5	0	0	20	7	2	22

67.5	5	0	12.5	0	40	2.5	4	26
10	20	0	0	0	12	10	8	13
32.5	10	20	37.5	100	68	26.5	28	15
10	5	20	25	0	60	11	15	18
45	30	5	12.5	33.33333	72	9.5	6	19
0	15	5	0	33.33333	60	7	2	20
45	10	15	37.5	33.33333	44	21.5	18	16
0	10	0	12.5	33.33333	48	7.5	4	27
22.5	55	15	25	33.33333	68	16	13	13
67.5	45	25	50	66.66667	76	33	33	6
32.5	70	40	50	0	88	20	30	17
80	70	55	100	100	64	66	68	14
22.5	20	10	12.5	0	24	4.5	8	22
22.5	5	5	0	0	0	7	2	35
45	25	0	0	0	28	5	0	20
32.5	10	5	12.5	0	32	7.5	6	32
10	10	30	12.5	0	52	20.5	14	38
35	25	5	12.5	0	24	9.5	6	22
10	20	20	50	100	60	21	23	26
22.5	10	5	37.5	0	64	9.5	14	34
32.5	20	0	37.5	0	96	17.5	13	25
0	35	10	12.5	33.33333	60	6.5	8	31
0	10	0	0	33.33333	48	0	0	32
32.5	55	30	62.5	66.66667	56	26.5	39	21
45	10	25	12.5	0	56	8.5	13	35
22.5	25	15	12.5	100	88	8.5	9	14
32.5	30	25	25	100	68	13	17	19
22.5	25	10	37.5	66.66667	68	16.5	16	18
22.5	25	10	25	0	68	7	12	17
0	15	0	50	33.33333	40	10	17	24
22.5	20	15	25	0	8	14	13	14
0	25	15	12.5	0	12	14.5	9	29
45	0	0	0	0	20	0	0	25
45	65	15	62.5	66.66667	80	21.5	26	22
22.5	30	30	37.5	0	44	20.5	23	24
0	65	30	12.5	0	68	10.5	14	18
45	40	0	25	100	96	10	8	20
45	50	15	50	0	40	19	22	23
0	0	0	25	33.33333	40	5	8	26
45	45	40	62.5	0	52	27.5	34	29
0	20	0	25	0	40	5	8	18
0	20	0	25	0	40	5	8	18
32.5	25	15	25	0	24	9	13	16
22.5	10	5	37.5	0	28	9.5	14	20

22.5	15	25	12.5	0	48	17.5	13	32
32.5	25	5	25	100	40	12	10	16
57.5	20	40	50	100	44	62	63	25
0	30	20	25	0	52	16	15	25
35	45	35	87.5	100	88	37.5	41	18
22.5	25	25	25	0	36	13	17	23
10	35	5	12.5	0	16	9.5	6	23
35	15	5	25	33.33333	52	7	10	18
22.5	20	10	25	66.66667	68	14	12	16
32.5	30	10	62.5	0	68	19.5	24	21
0	10	10	0	0	20	4	3	27
100	55	40	37.5	100	76	61.5	51	5
10	15	5	0	0	44	2	2	35
22.5	0	5	25	0	48	7	10	29
22.5	45	20	25	100	80	11	15	34
22.5	15	0	0	33.33333	8	0	0	24
35	35	15	37.5	0	60	13.5	18	30
35	25	5	25	0	44	17	10	20
35	20	0	37.5	100	84	12.5	13	16
22.5	15	15	12.5	0	24	6.5	9	27
32.5	10	35	12.5	0	48	6.5	16	23
32.5	60	25	50	0	60	21	25	23
32.5	60	25	50	0	60	21	25	23
22.5	30	25	25	0	56	11	17	29
45	20	15	25	33.33333	60	29	22	22
45	20	10	37.5	100	68	21.5	16	17
22.5	0	5	25	0	48	7	10	29
0	0	0	12.5	0	4	2.5	4	32
10	25	10	37.5	100	64	9.5	16	36
22.5	35	20	25	100	80	11	15	34
0	0	10	0	0	76	7	3	22
22.5	5	0	0	0	16	0	0	37
0	25	0	0	100	84	0	0	
22.5	30	25	25	0	56	11	17	29
0	0	25	0	0	20	10	8	44
22.5	15	35	37.5	0	36	24.5	24	18
0	20	15	0	0	36	6	5	42
45	25	20	37.5	100	56	36.5	36	18
32.5	35	5	37.5	33.33333	60	12.5	14	22
0	10	0	0	0	24	0	0	38
20	20	20	62.5	0	68	23.5	28	22

32.5	30	40	75	0	16	46	47	13
22.5	50	20	25	0	52	16	15	22
45	45	0	12.5	100	92	7.5	4	16
22.5	25	10	25	0	68	7	12	17
45	35	0	50	66.66667	76	25	25	15
10	15	5	0	0	44	2	2	35
22.5	20	10	25	66.66667	68	14	12	16
45	40	0	25	100	96	10	8	20
67.5	5	0	12.5	0	40	2.5	4	26
45	10	15	37.5	33.33333	44	21.5	18	16
32.5	70	40	50	0	88	20	30	17
45	0	0	0	0	20	0	0	25
45	15	35	25	0	48	20	20	
0	10	0	12.5	33.33333	48	7.5	4	27
22.5	50	10	12.5	100	80	39.5	33	22
22.5	30	10	25	100	68	12	12	19
67.5	35	60	87.5	33.33333	64	65.5	66	5
35	20	0	37.5	33.33333	72	12.5	13	21
57.5	35	0	25	100	68	35	25	12

57.5	55	25	75	100	80	38	42	15
80	60	60	75	100	76	85	78	0
0	30	0	12.5	0	64	7.5	4	22
100	65	0	25	100	76	10	8	7
100	55	55	100	100	80	88	85	0
57.5	55	30	50	66.66667	80	20	27	15
100	20	35	37.5	100	64	24.5	24	8
22.5	45	40	75	100	92	37	38	23
67.5	30	15	75	100	76	39	38	8
67.5	60	20	62.5	33.33333	56	28.5	28	24
100	45	30	37.5	100	76	67.5	56	14
22.5	60	30	75	100	64	32	35	20
67.5	55	60	75	33.33333	68	44	45	6
80	70	45	62.5	100	52	74.5	69	3

90	80	55	100	66.66667	88	78	77	0
100	90	70	100	100	84	92	90	3
90	65	100	100	100	88	100	100	0
100	85	85	100	100	96	96	95	0
100	80	50	100	100	76	86	83	0
67.5	80	60	87.5	100	60	87.5	83	6
90	70	75	100	100	76	92	92	3
100	100	100	100	100	100	100	100	0
100	45	35	100	100	64	75	78	0
57.5	40	15	25	33.33333	80	14	13	15
100	20	25	12.5	0	28	16.5	13	11
90	60	55	62.5	0	44	80.5	73	4
100	95	70	100	100	64	90	90	1
100	90	60	87.5	100	72	83.5	83	0
90	95	80	100	100	88	89	93	7
90	85	80	100	100	88	94	93	4
100	80	85	100	100	88	96	95	3
100	85	75	100	100	92	94	92	1
90	70	60	75	100	72	83	78	1
100	65	50	87.5	100	72	81.5	79	4
90	75	80	100	100	92	84	93	2
90	70	75	100	100	92	92	92	7
100	75	90	100	100	92	98	97	0
100	95	80	100	100	92	94	93	2
90	90	80	100	100	72	94	93	0
80	100	85	100	100	92	96	95	2
90	95	65	100	100	72	90	88	1
100	60	75	100	0	64	82	83	
35	50	50	62.5	100	64	53.5	54	8
100	85	75	100	100	84	92	92	0
100	75	75	100	100	88	94	92	
100	100	85	100	100	88	96	95	4
100	95	85	100	100	92	96	95	0
90	75	80	100	100	84	94	93	2
77.5	80	60	100	100	80	88	87	2
22.5	45	50	25	100	68	21	25	22
77.5	35	5	62.5	100	44	59.5	56	12
90	80	60	75	33.33333	40	83	78	4
90	40	20	87.5	100	84	33.5	36	9
67.5	85	65	87.5	33.33333	84	74.5	76	2
100	90	70	100	100	88	94	90	0
100	95	75	100	100	76	92	92	0
100	85	75	100	100	80	92	92	0

90	95	70	75	100	80	87	82	3

CMSI Dysp	CMSI Cardi	CMSI Head	CMSI Neur	CMSI Ear&	CMSI GI	CMSI Blad	CMSI_172_	CMSI_NoPa
10	2	4	8	2	3	2	54	31
20	8	0	15	3	8	4	89	58
0	6	0	13	10	7	13	78	49
14	2	0	10	0	0	3	68	50
20	8	3	10	6	15	3	84	65
15	3	0	12	4	10	11	76	55
0	0	3	2	10	0	0	30	15
5	5	2	12	0	8	4	55	36
10	11	5	9	5	19	11	94	70
2	0	0	0	0	0	0	13	2
0	0	0	7	3	17	6	50	33
8	4	6	5	2	0	11	58	36
7	2	3	8	4	18	9	71	51
5	0	7	6	6	0	2	50	26
3	0	0	7	0	6	2	31	18
18	3	4	5	3	0	0	61	33
8	0	3	11	14	29	9	90	74
0	0	0	5	1	11	0	36	17
8	2	0	8	10	15	0	58	43
10	7	0	9	3	20	9	87	58
8	2	5	7	8	5	4	52	39
5	2	6	4	3	5	0	41	25
5	3	4	9	2	17	14	83	54
1	2	4	2	0	1	1	23	11
0	1	1	7	1	7	1	35	18
5	0	3	9	1	0	2	34	20
10	5	3	7	3	3	3	45	34
0	0	0	0	0	0	0	0	0
5	2	4	7	8	12	5	62	43
3	1	0	8	0	6	5	34	23
11	7	1	6	2	15	0	65	42
3	7	3	6	2	5	2	47	28
12	10	7	13	12	19	10	118	83
3	1	2	9	0	15	0	45	30
6	0	0	1	2	0	0	15	9
11	3	6	5	2	8	0	47	35
0	2	0	1	0	9	0	19	12
13	5	3	8	7	5	0	63	41

8	4	5	9	7	14	9	71	56
5	3	4	11	12	16	6	84	57
4	7	4	5	2	8	5	46	35
11	6	7	14	8	20	0	97	66
14	0	2	6	1	5	3	52	31
18	12	4	15	4	25	12	124	90
15	2	0	7	7	2	7	57	40
10	0	0	0	0	0	2	28	12
2	0	6	11	10	16	0	66	45
2	2	5	5	5	7	1	51	27
12	2	7	12	2	0	0	55	35
8	7	5	12	6	18	8	95	64
3	0	2	3	1	3	0	22	12
11	0	3	1	1	0	0	28	16
0	0	0	6	3	0	0	13	9
0	0	4	5	4	0	0	22	13
0	1	1	5	0	0	0	17	7
0	1	0	5	0	9	0	24	15
5	2	0	3	1	6	2	32	19
12	6	2	1	6	0	3	49	30
5	4	0	7	1	9	1	45	27
6	9	2	7	5	15	8	74	52
8	3	0	10	2	12	0	53	35
7	1	0	4	4	0	0	23	16
0	0	4	3	8	13	1	38	29
12	0	0	5	4	3	0	35	24
8	3	3	5	0	5	0	35	24
2	2	0	6	4	3	0	27	17
13	2	0	5	4	1	4	50	29
3	4	0	7	6	3	0	36	23
2	0	5	5	1	26	4	57	43
0	1	3	3	1	9	2	27	19
1	1	2	3	11	14	11	60	43
1	6	3	10	0	14	2	62	36
12	3	0	6	7	0	1	48	29
1	0	2	8	10	11	3	58	35

1	0	1	6	5	9	1	36	23
14	3	0	5	4	10	3	57	39
0	1	3	6	5	10	1	38	26
1	0	2	1	1	0	0	12	5
0	0	3	5	3	8	0	32	19
0	2	0	8	4	3	0	29	17
14	5	0	2	9	12	0	50	42
8	6	0	9	2	2	2	42	29
11	2	1	6	11	24	11	80	66
5	2	2	5	7	9	3	49	33
6	9	5	9	11	18	10	94	68
2	1	1	5	11	19	3	53	42
0	0	0	0	0	0	0	0	0
2	0	6	6	6	4	0	36	24
5	2	2	5	7	9	3	49	33
0	0	0	0	0	0	0	0	0
2	0	6	7	7	7	0	42	29
2	1	6	7	5	15	0	52	36
16	4	8	13	8	14	1	97	64
13	3	3	8	9	15	0	71	51
2	0	3	10	3	23	4	63	45
13	7	4	8	4	6	0	60	42
9	11	1	6	8	17	0	72	52
5	0	3	6	8	23	4	77	49
9	2	2	7	10	22	1	73	53
2	1	3	7	13	26	7	79	59
17	4	7	10	16	29	5	125	88
6	6	5	10	15	18	0	81	60
7	7	6	7	9	19	11	94	66
15	7	8	11	10	21	7	105	79
12	4	4	12	8	28	5	106	73
8	2	4	8	5	22	4	79	53
13	2	0	6	5	12	4	65	42
16	4	8	11	3	17	3	74	62
10	0	1	5	2	9	5	51	32
16	12	7	14	11	24	9	129	93
16	12	4	12	11	21	10	121	86
6	1	2	8	3	3	0	37	23
11	0	4	8	1	12	0	53	36
12	6	1	7	8	15	4	71	53
10	8	2	11	2	3	3	62	39
11	8	6	11	11	14	8	91	69
4	7	8	13	16	21	10	101	79
8	9	8	10	11	17	4	89	67

6	4	4	11	10	29	3	93	67
2	9	0	8	6	20	4	62	49
15	6	5	3	2	15	1	62	47
8	4	4	7	6	9	1	57	39
1	0	0	10	6	3	0	39	20
5	1	8	9	6	29	5	83	63
0	0	3	7	8	18	0	52	36
1	3	2	9	6	3	2	53	26
4	0	0	0	0	0	0	17	4
0	0	0	3	2	0	0	11	5
5	0	4	6	6	18	0	56	39
3	4	5	6	0	0	0	32	18
11	4	6	11	12	22	8	96	74
18	13	8	14	13	30	8	139	104
8	3	3	8	3	19	6	70	50
18	12	8	16	13	19	10	128	96
11	1	8	16	20	30	8	132	94
7	2	3	9	7	23	2	75	53
2	0	0	13	8	20	3	72	46
5	11	5	15	12	22	5	109	75
5	3	0	16	20	24	4	97	72
11	1	6	12	12	18	3	94	63
11	9	6	11	9	28	12	118	86
16	7	5	13	8	23	8	101	80
11	15	0	12	13	21	13	120	85
3	3	6	10	4	21	4	65	51
10	2	4	10	9	18	3	75	56
10	1	1	7	1	14	1	53	35
9	4	4	10	8	24	0	76	59
6	4	4	10	7	3	0	58	34
5	1	0	7	4	13	3	47	33
9	4	7	5	3	28	11	96	67
15	9	7	13	4	12	5	90	65
13	6	6	6	8	17	5	83	61
8	1	5	12	8	24	5	87	63
15	1	5	13	10	18	5	85	67
0	0	8	7	3	10	9	57	37
8	6	5	11	10	25	5	93	70
20	8	6	13	11	25	8	117	91
6	0	5	14	1	12	8	75	46
5	2	2	10	3	8	1	49	31
5	2	2	10	3	8	1	49	31
1	0	2	6	3	17	0	45	29
20	2	4	14	3	17	1	81	61

13	4	7	8	8	22	6	100	68
7	5	6	11	9	19	5	78	62
7	7	5	8	5	8	4	69	44
8	8	7	11.5	10	13.5	7.5	90.5	65.5
3	1	6	7	9	17	6	67	49
5	6	7	6	10	20	4	81	58
20	4	6	4	8	25	15	105	82
15	8	2	10	3	27	7	90	72
4	5	1	6	3	17	1	53	37
9	5	6	10	12	19	10	92	71
14	10	1	14	6	20	6	98	71
3	0	0	4	1	5	0	18	13
20	5	8	11	13	31	9	132	97
19	0	8	11	19	28	4	118	89
8	2	4	14	5	13	7	87	53
9	6	8	11	10	31	8	107	83
11	7	4	9	10	20	3	94	64
5	2	3	9	7	4	1	51	31
0	0	8	4	5	0	6	39	23
11	2	3	7	7	26	9	92	65
12	12	3	11	8	19	5	93	70
8	0	2	6	5	8	5	57	34
8	0	2	6	5	8	5	57	34
18	5	6	15	15	19	12	119	90
5	4	1	5	8	3	1	49	27
5	0	6	5	3	12	10	58	41
19	0	8	11	19	28	4	118	89
15	5	8	8	12	19	5	104	72
13	7	6	14	10	17	5	108	72
8	2	4	14	5	13	7	87	53
9	5	4	8	6	18	7	79	57
20	9	8	13	15	30	13	145	108
18	5	6	15	15	19	12	119	90
20	16	8	16	20	32	16	172	128
12	6	1	7	8	15	4	71	53
15	5	8	12	15	30	12	139	97
18	9	6	5	3	16	0	75	57
9	2	6	6	7	10	6	68	46
16	6	7	12	15	30	9	133	95
4	7	8	13	16	21	10	101	79

11	0	3	3	3	20	0	53	40
4	7	8	13	16	21	10	101	79
1	0	3	4	0	4	2	30	14
9	4	4	10	8	24	0	76	59
10	1	4	4	4	19	0	57	42
20	5	8	11	13	31	9	132	97
4	5	1	6	3	17	1	53	37
0	0	8	7	3	10	9	57	37
6	4	4	11	10	29	3	93	67
0	0	3	7	8	18	0	52	36
5	0	4	6	6	18	0	56	39
15	9	7	13	4	12	5	90	65
1	3	2	9	6	3	2	53	26
11	8	6	11	11	14	8	91	69
5	5	2	6	3	19	1	60	41
0	0	2	3	3	15	0	28	23
7	3	0	5	3	16	0	55	34
2	0	6	6	6	4	0	36	24
2	0	6	2	2	12	9	48	33
0	0	0	0	0	0	0	0	0
0	0	4	14	0	13	10	63	41
10	2	0	11	11	3	5	49	42
0	0	0	0	0	0	0	0	0
2	1	2	6	9	4	2	41	26
4	0	0	2	3	1	0	18	10
0	0	0	3	0	1	3	30	7
0	2	1	6	5	0	0	22	14
6	5	4	8	9	13	9	78	54
3	0	3	3	0	0	0	23	9
5	6	4	4	5	8	5	57	37
0	0	0	0	0	3	0	9	3
4	0	0	0	3	0	0	10	7

0	0	0	0	0	0	0	0	0
2	0	0	0	0	0	0	5	2
0	0	0	0	0	2	0	2	2
0	0	0	0	0	0	0	0	0
0	0	0	0	0	0	0	0	0
1	0	1	2	5	3	0	18	12
0	0	0	0	6	0	0	9	6
0	0	0	0	0	0	0	0	0
0	0	0	0	0	0	0	0	0
4	6	6	6	5	2	2	46	31
1	0	2	8	0	0	0	22	11
1	0	4	1	0	4	1	15	11
0	1	0	0	0	2	1	5	4
0	0	0	0	0	0	0	0	0
1	0	0	1	3	3	2	17	10
0	0	0	0	0	0	0	4	0
1	0	0	1	0	0	0	5	2
2	0	0	0	1	0	1	5	4
0	0	0	0	0	0	0	1	0
5	1	0	2	0	9	0	21	17
2	1	1	1	1	0	0	8	6
0	0	1	1	1	1	3	14	7
2	1	0	1	1	0	0	5	5
1	0	0	1	0	0	0	4	2
0	0	0	0	0	0	0	0	0
1	0	0	1	0	0	0	4	2
1	1	2	0	0	0	0	5	4
0	0	6	3	2	0	0	19	11
0	0	0	0	0	0	0	0	0
1	0	0	0	0	0	0	5	1
0	0	0	0	1	0	0	1	1
1	0	0	1	0	0	0	4	2
3	0	0	0	0	0	0	5	3
9	3	0	8	1	19	3	65	43
4	2	4	8	9	24	0	63	51
0	0	6	1	1	14	0	26	22
5	1	3	2	5	3	7	35	26
0	0	0	1	4	0	0	7	5
0	0	0	0	0	0	0	0	0
0	0	0	0	0	0	0	0	0
0	0	0	0	0	0	0	0	0

0	0	0	1	1	1	0	6	3

C:chemica	C:other_ex	C:symptom	C:masking_	C:impact_c	RhSc Rhinit	RhSc Chest	RhSc Sum	IRS Conges
51	23	84	3	59	16	7	23	3
57	54	72	2	50	17	9	26	21
65	23	60	4	59	17	7	24	14
52	24	39	3	21	4	6	10	0
57	10	56	3	42	13	8	21	3
72	32	33	0	60				
67	26	56	2	24	15	8	23	13
					7	1	8	12
31	75	58	2	67	13	8	21	19
96	62	91	2	100	13	15	28	20
					7	2	9	0
68	36	74	2	27	14	0	14	3
33	31	56	7	13	14	4	18	4
16	15	75	6	8	7	6	13	6
78	35	64	4	90	0	0	0	8
24	0	23	5	0	6	5	11	0
50	45	47	5	62	21	18	39	21
0	2	15	5	0	5	0	5	4
14	18	35	4	10				
					12	2	14	4
31	11	45	5	4	7	4	11	13
51	17	33	3	21	6	1	7	13
17	19	10	4	5	6	6	12	11
59	31	34	6	18	17	5	22	9
45	44	56	3	80	0	0	0	0
61	37	31	5	12	6	3	9	6
35	32	44	3	60	4	0	4	13
84	39	45	2	43	12	9	21	6
29	30	29	2	62	11	1	12	16
68	36	29	5	37	7	2	9	10
69	45	80	3	89	28	9	37	10
30	40	25	3	31	9	0	9	7
					20	11	31	27
16	14	10	2	8	6	0	6	8
100	80	87	3	95	25	22	47	23
53	58	51	1	74	5	0	5	18
2	0	1	4	0	0	0	0	0
9	0	5	1	5	17	6	23	0
18	11	24	2	22	8	4	12	12
35	2	42	2	0	7	5	12	13

29	36	36	2	25	27	9	36	17
28	20	51	3	8	8	2	10	15
26	22	25	6	9	8	3	11	7
80	63	65	2	73	40	14	54	20
8	3	6	4	4	6	2	8	0
58	27	32	1	55	1	4	5	2
40	7	55	4	30	16	27	43	7
16	13	34	5	15	14	0	14	6
20	9	35	5	14	9	0	9	13
31	4	12	3	0	22	0	22	3
					22	3	25	16
15	17	6	3	2	11	4	15	10
8	9	23	0	0	15	0	15	4
0	15	42	3	1	17	0	17	3
3	0	24	2	18	13	0	13	2
24	0	23	5	0	0	0	0	0
10	14	13	2	9	1	0	1	8
					11	2	13	8
0	16	13	1	0	0	5	5	0
					3	9	12	0
64	51	66	3	69	10	9	19	24
73	23	47	2	59	10	2	12	0
19	12	10	4	2				13
71	27	38	4	59	18	0	18	19
0	0	0	7	0	5	3	8	2
10	10	6	1	0	10	0	10	9
29	21	21	2	9	14	4	18	14
20	24	38	4	50	24	16	40	16
7	6	34	3	2	19	5	24	9
					8	1	9	0
11	39	27	2	2	5	0	5	1
18	12	43	7	49	3	0	3	
16	2	49	8	20	6	2	8	0
71	18	51	4	33	17	3	20	23
59	29	48	4	46	16	15	31	21
33	32	30	4	30	16	1	17	14

5	10	26	3	13	3	0	3	1
26	22	73	5	8	5	22	27	6
25	20	50	3	35	5	0	5	2
0	0	1	4	0	13	1	14	4
8	3	30	4	0	20	17	37	6
0	0	19	2	26	0	0	0	0
11	16	23	3	40	6	4	10	0
45	28	19	5	45	16	7	23	19
25	29	60	3	5	33	27	60	10
43	12	41	9	6	10	13	23	11
4	39	7	5	0	22	8	30	13
14	30	35	5	13	6	0	6	6
55	14	74	7	37				
52	26	20	4	18	4	2	6	7
43	12	41	9	6	10	13	23	11
58	34	25	4	37	4	0	4	2
46	21	19	4	11	6	0	6	0
64	22	60	4	33	16	25	41	14
80	46	72	2	83	20	16	36	18
18	24	31	4	30	1	0	1	
50	42	68	2	66	22	13	35	16
47	34	62	3	66	22	12	34	10
41	19	68	5	0	7	8	15	9
44	10	46	2	13	12	15	27	13
60	45	56	4	44	28	20	48	23
89	67	79	3	88	35	34	69	
57	24	79	5	63	9	2	11	10
41	27	61	3	24	23	10	33	19
58	44	81	8	48	21	20	41	11
40	24	53	3	20	25	17	42	25
59	13	70	2	55	13	11	24	12
14	4	35	3	0	3	4	7	0
5	19	74	4	58	12	10	22	3
30	28	30	5	29	10	12	22	
81	16	74	4	71	31	15	46	17
82	46	70	3	72	24	21	45	24
14	0	38	5	8	0	0	0	0
38	32	52	5	39	15	14	29	19
38	1	46	5	80	9	8	17	5
50		54	4	43	10	4	14	14
0	0	0	3	0	16	10	26	12
69	26	79	5	61	25	7	32	17
36	42	58	3	58	20	1	21	16

26	17	74	3	10	29	26	55	26
9	5	45	4	54	7	4	11	2
36	24	41	5	28	23	25	48	14
41	18	33	2	26	12	11	23	14
15	20	21	3	0	21	5	26	4
8	6	64	2	4	6	0	6	2
57	49	53	3	53				13
16	7	50	2	1	10	6	16	1
46	34	25	4	3	23	13	36	8
26	16	43	3	0	14	5	19	5
76	40	48	5	60	10	10	20	14
31	15	40	3	16	26	9	35	12
29	17	39	4	16	18	13	31	16
43	0	68	6	0	40	36	76	24
19	17	38	4	19	6	0	6	8
73	28	89	4	75	31	18	49	22
66	38	62	4	67	35	34	69	24
56	21	71	2	49	21	20	41	
47	25	54	7	63	10	12	22	15
20	29	77	7	45	14	8	22	8
58	25	40	4	28	28	9	37	16
61	23	63	6	68	0	0	0	14
54	47	71	3	91	11	2	13	17
57	17	78	4	61	26	20	46	25
81	62	72	1	87	22	17	39	22
11	12	60	2	40	10	3	13	6
82	63	83	3	67	15	5	20	17
11	33	22	2	9	1	1	2	3
55	29	61	6	45	14	9	23	10
24	12	55	2	13	13	4	17	3
13	0	30	4	3	0	1	1	2
18	40	47	3	75	29	4	33	20
24	0	72	3	0	16	6	22	11
51	16	61	2	27	23	10	33	22
56	45	71	4	60	17	10	27	21
68	39	55	5	41	27	22	49	26
6	9	4	5	2	6	0	6	2
46	47	63	5	59	14	0	14	20
57	52	81	4	75	25	12	37	7
21	52	75	9	50	15	1	16	16
42	28	9	5	18	16	0	16	14
42	28	9	5	18	16	0	16	14
34	15	21	4	37	12	3	15	7
41	21	63	4	64	21	19	40	

70	37	67	4	67	20	10	30	28
11	8	27	2	0	14	1	15	7
52	26	55	4	34	19	12	31	18
47	0	0	4	0	16	9	25	16
83	0	52	4	66	20	5	25	20
53	26	61	3	57	11	8	19	12
54	10	80	3	73	31	33	64	22

64	33	73	1	87	4	6	10	5
9	7	30	2	23	26	13	39	6
70	25	59	4	35				15
71	33	32	4	46	8	14	22	12
0	2	14	4	0	20	2	22	0
90	41	88	3	76	28	19	47	19
79	29	76	2	61	32	23	55	18
69	54	60	4	65	13	5	18	0
95	0	90	2	28				
62	65	73	3	85	16	13	29	12
25	42	68	3	57	21	7	28	18
54	11	69	8	13	16	9	25	13
16	26	55	5	8	40	36	76	
49	5	70	3	36	21	22	43	8
71	0	80	2	90	5	6	11	14
19	24	54	2	23	6	1	7	5
19	24	54	2	23	6	1	7	5
59	44	94	6	61	19	12	31	23
48	22	73	7	28	12	6	18	12
13	27	31	3	28	13	2	15	11

79	29	76	2	61	32	23	55	18
50	4	32	1	26	30	24	54	21
83	62	69	8	73	24	20	44	25
69	54	60	4	65	13	5	18	0
43	24	83	5	27	23	9	32	18
87	26	83	1	77	30	29	59	27

59	44	94	6	61	19	12	31	23
90	57	99	3	90	40	36	76	25
38	1	46	5	80	9	8	17	5
					30	21	51	19
35	0	49	7	5	23	14	37	17
46	34	50	4	35	24	9	33	23
76	23	78	4	72	27	26	53	26
69	26	79	5	61	25	7	32	17

74	15	50	5	37	2	8	10	25
60	31	44	5	20	25	7	32	17
0	19	17	6	0	0	0	0	0
55	29	61	6	45	14	9	23	10
64	12	50	4	43	10	4	14	21.66667
90	41	88	3	76	28	19	47	19
9	7	30	2	23	26	13	39	6
6	9	4	5	2	6	0	6	2
26	17	74	3	10	29	26	55	26
57	49	53	3	53	25	1	26	13
76	40	48	5	60	10	10	20	14
24	0	72	3	0	16	6	22	11
49	10	51	7	18				
16	7	50	2	1	10	6	16	1
0	0	0	3	0	16	10	26	12
					18.5	9	27.5	6
29	37	35	4	38	21	0	21	12
27	15	25	3	17	13	5	18	6
52	26	20	4	18	4	2	6	7

64	48	38	6	24	6	2	8	11
11	9	20	7	0	3	0	3	9
					6	1	7	2
					12	7	19	16
5	0	1	2	0	0	0	0	0
26	34	38	2	13	18	0	18	3
8	1	12	4	0	1	1	2	1
0	16	14	5	17	8	4	12	2
26	14	19	3	10	7	1	8	9
36	18	66	4	15	19	5	24	6
0	0	0	4	0	1	0	1	1
					5	0	5	2
13	12	19	2	0	6	0	6	4
0	5	15	4	5	5	0	5	6

46	7	9	1	0	5	4	9	4
40	21	14	2	13	4	0	4	13
3	0	2	4	2	2	0	2	1
3	0	0	4	0	0	0	0	0
1	0	1	5	0	0	0	0	0
5	12	20	5	4	6	0	6	2
9	0	0	5	0	0	0	0	3
2	0	1	5	0	0	0	0	0
0	0	0	1	0	0	1	1	8
45	16	32	1	26	8	5	13	9
23	12	24	4	11	3	0	3	0
3	13	8	3	16	4	0	4	0
6	4	8	4	0	0	1	1	2
2	0	0	3	0	0	0	0	5
25	10	13	5	0	10	1	11	9
3	1	0	4	0	2	0	2	0
0	0	0	3	0	0	0	0	0
0	5	2	2	0	21	3	24	1
0	0	0	6	0	0	0	0	1
12	14	9	5	1	2	2	4	0
14	13	7	5	1	12	9	21	1
51	16	24	4	9	7	0	7	13
8	3	3	5	0	2	0	2	5
0	0	0	3	0	0	0	0	0
8	0	0	1	0	8	2	10	0
					0	0	0	0
8	1	3	5	7	1	0	1	
2	0	0	2	0				
16	1	16	5	4	8	2	10	
0	0	6	2	0	2	0	2	3
0	0	0	3	0				
2	0	0	6	1	0	0	0	1
26	0	0	3	1	2	0	2	1
5	2	9	5	0	0	1	1	3
3	3	0	5	1	2	0	2	0
					12	5	17	10
78	43	57	3	49	6	1	7	5
29	7	31	4	3	8	0	8	14
6	13	42	4	13	5	2	7	7
18	2	0	4	0	10	0	10	9
2	4	4	3	1	1	0	1	0
5	1	2	2	0	7	0	7	2
10	4	10	2	0	20	0	20	6

2 0 0 5 8 7 1 8 0

IRS Rhinorr	IRS sum	USCD Dysp	∑Urinary_A	COMPASS	COMPASS	COMPASS	COMPASS	COMPASS
12	15	54	19					
15	36	81	9					
8	22	64	26					
0	0	27	7					
2	5	93	3					
			6					
6	19	38	22					
9	21	5	11					
4	23	16	7					
15	35	58	16					
6	6	3	10					
8	11	59	6					
1	5	33	11					
6	12	25	17					
0	8	5	6					
0	0	8	8					
13	34	81	7					
0	4	8	27					
			10					
8	12	1	12					
0	13	39	1					
12	25	6	28					
9	20		4	32.0	2.5	0.0	6.2	0.0
8	17		14	0.0	0.0	6.4	7.1	2.2
0	0		24					
3	9		0	12.0	0.0	10.7	7.1	0.0
0	13		17	20.0	0.0	0.0	10.7	1.1
4	10		12	24.0	1.7	4.3	6.2	0.0
0	16		15	24.0	0.0	6.4	6.2	0.0
6	16		20	24.0	1.7	4.3	13.4	4.4
10	20		18	24.0	0.0	8.6	14.3	1.1
5	12		9	16.0	1.7	0.0	2.7	2.2
26	53		11	12.0	2.5	8.6	13.4	1.1
2	10		20	24	0	6.428571	9.821428	2.222222
23	46		19	16.0	0.0	8.6	10.7	4.4
9	27		3	12.0	0.0	6.4	12.5	0.0
1	1		1	4.0	0.0	0.0	0.0	0.0
0	0	47	4					
9	21		0	0.0	0.0	2.1	0.0	0.0
8	21	37	8					

15	32		13	24.0	0.0	4.3	16.1	2.2
3	18	33	18					
3	10		7	24.0	1.7	0.0	9.8	0.0
20	40	17	5					
0	0		16	12.0	1.7	10.7	4.5	1.1
3	5		18	4.0	0.0	0.0	2.7	3.3
0	7	32	16					
11	17	15	0					
9	22	19	11					
5	8		6	20.0	0.0	0.0	0.0	1.1
11	27	5	22					
5	15		6	0	0	2.142857	4.464286	0
4	8		0	0.0	0.0	0.0	6.2	0.0
3	6	2	0					
0	2	0	6					
3	3	10	6					
14	22	9	2					
9	17	43	15					
0	0	63	8					
0	0		4	12.0	1.7	2.1	10.7	2.2
0	24		13	16.0	1.7	10.7	10.7	1.1
1	1		3	20.0	0.0	6.4	8.9	0.0
12	25		6	20.0	1.7	0.0	6.2	2.2
14	33		5	16.0	0.0	0.0	6.2	0.0
0	2		1	0.0	1.7	4.3	5.4	0.0
11	20		1	24.0	0.0	0.0	5.4	0.0
10	24		9	16	0	0	11.60714	0
14	30		26	8.0	0.0	8.6	10.7	4.4
9	18		0	16.0	0.0	2.1	5.4	0.0
0	0	2	5					
0	1		7	20.0	0.0	0.0	8.9	0.0
			8					
			28	20.0	0.0	4.3	13.4	7.8
4	4		15	20.0	0.0	2.1	9.8	2.2
14	37	11	15					
13	34		5	12.0	0.8	8.6	7.1	1.1
5	19	4	6					

4	5		1	0.0	2.5	4.3	10.7	0.0
0	6	38	27					
3	5	5	21					
3	7	7	3					
6	12		4	8.0	0.0	2.1	7.1	0.0
0	0		1	24.0	0.0	0.0	3.6	0.0
11	11		13	20.0	1.7	6.4	8.9	2.2
19	38		5	8.0	0.0	6.4	8.9	1.1
9	19		17	24.0	3.3	6.4	16.1	3.3
8	19	9	9					
6	19	31	26					
1	7			8.0	0.0	0.0	9.8	0.0
			11					
7	14	7	1					
8	19	9	9					
			1					
7	9	7	4					
9	9	7	4					
11	25		11	28.0	1.7	8.6	17.0	0.0
8	26		13	8.0	0.0	8.6	10.7	2.2
			11	8.0	0.0	2.1	12.5	2.2
11	27		1	12.0	0.0	4.3	6.2	0.0
10	20		19	16.0	0.0	4.3	11.6	2.2
5	14		21	8.0	0.8	6.4	13.4	2.2
13	26		21	4.0	0.0	0.0	14.3	2.2
25	48		15	20.0	0.0	6.4	15.2	0.0
			11	32.0	0.0	8.6	17.9	0.0
12	22		16	28.0	0.0	4.3	9.8	2.2
16	35		25	12.0	0.0	6.4	12.5	4.4
6	17		31	12.0	3.3	8.6	16.1	6.7
20.5	45.5	42	18					
16	28		25	24.0	2.5	0.0	11.6	3.3
0	0		21	20.0	1.7	4.3	9.8	2.2
2	5		17	24.0	0.0	0.0	8.9	2.2
			23	8.0	0.8	8.6	8.9	6.7
17	34	86	24					
24	48	74	28					
0	0	6	13					
17	36	40	9					
5	10	41	4					
14	28	20	5					
16	28	7	26					
16	33	35	10					
13	29		7	24.0	0.0	2.1	8.0	0.0

24	50	21	18					
0	2	23	11					
16	30	63	7					
14	28	22	21					
0	4	11	13					
6	8	34	9					
17	30	4	10					
1	2	16	6					
8	16	38	10					
7	12	28	3					
12	26	21	6					
0	12		6	20.0	0.0	4.3	2.7	0.0
10	26		24	28.0	0.0	8.6	12.5	2.2
24	48		14	36.0	0.0	15.0	11.6	0.0
9	17		25	12.0	1.7	8.6	15.2	4.4
18	40		18	28.0	1.7	8.6	15.2	2.2
25	49		28	32.0	2.5	6.4	15.2	3.3
			2	8.0	0.0	10.7	14.3	0.0
18	33		21	28.0	0.0	8.6	17.0	2.2
8	16		26	16.0	0.0	6.4	15.2	6.7
16	32		5	28.0	0.0	8.6	11.6	1.1
13	27		13	24.0	2.5	10.7	10.7	0.0
15	32		27	28.0	2.5	10.7	15.2	3.3
7	32		24	24.0	0.0	10.7	12.5	6.7
23	45		27	32.0	3.3	12.9	12.5	4.4
0	6		12	0.0	0.0	2.1	13.4	1.1
13	30	52	15					
1	4		1	16.0	0.0	0.0	12.5	0.0
6	16	25	20					
2	5	10	8					
2	4	22	8					
20	40	7	24					
13	24	81	3					
15	37		22	12.0	0.0	4.3	11.6	1.1
20	41		13	0.0	1.7	6.4	16.1	6.7
28	54	74	4					
4	6	7	4					
17	37	34	10					
5	12		28	32.0	0.0	10.7	18.7	3.3
14	30		31	28.0	0.0	10.7	16.1	7.8
15	29	11	8					
15	29	11	8					
7	14	6	3					
			13	24.0	0.0	6.4	11.6	1.1

23	51		18	0.0	1.7	8.6	17.0	2.2
6	13		4	20.0	0.0	0.0	13.4	0.0
12	30	44	13					
17	33	4	26					
8	28	21	24					
13	25	19	14					
20	42	107	28					
5	10	19	19					
1	7	12	11					
15	30		24	16.0	0.0	6.4	15.2	4.4
15	27		22	32.0	0.0	8.6	16.1	2.2
4	4	14	4					
18	37	64	20					
16	34	102	3					
13	13	43	22					
			11					
22	34	33	12					
12	30	74	1					
15	28	9	13					
			25	16.0	0.0	2.1	8.9	2.2
8	16	77	21					
13	27	33	14					
1	6	30	17					
1	6	30	17					
21	44	65	19					
9	21	10	8					
2	13		25	20.0	0.0	8.6	11.6	6.7
16	34	88	3					
22	43	86	3					
25	50	80	26					
13	13	43	22					
18	36	36	17					
26	53	105	30					
21	44	65	19					
23	48	112	32					
5	10	41	4					
12	31	39	30					
10	27	67	6					
16	39		19	16.0	1.7	6.4	13.4	3.3
25	51	91	24					
16	33	35	10					

21	46	46	0
16	33	35	6
3	3	1	10
6	16	25	20
20.33333	42	62	0
18	37	64	20
1	7	12	11
4	6	7	4
24	50	21	18
17	30	4	10
12	26	21	6
13	24	81	3
			6
1	2	16	6
16	28	2	26
2	8	23	
12	24	2	3
6	12	29	9
7	14	6	1

17	28	18	3					
1	10	3	21					
3	5	5	23					
11	27	48	15					
0	0	4	1					
3	6		5	8.0	0.0	4.3	5.4	1.1
2	3		2	12.0	0.0	4.3	4.5	0.0
2	4		9	12.0	0.0	6.4	3.6	1.1
2	11		13	16.0	0.0	0.0	0.0	4.4
0	6	21	19					
0	1		8	0.0	0.0	4.3	3.6	1.1
0	2	8	14					
0	4	0	6					
3	9	12	4					

10	14	4	3					
13	26	9	4					
1	2	2	2					
2	2	0	7					
0	0	2	4					
3	5	3	10					
2	5	6	2					
0	0	0	0					
9	17		0	0.0	0.0	2.1	1.8	0.0
4	13		12	16.0	0.0	6.4	7.1	2.2
0	0		0	36.0	0.0	0.0	4.5	0.0
0	0		5	16.0	0.0	0.0	10.7	0.0
1	3		3	16.0	0.0	0.0	3.6	0.0
5	10		0	16.0	0.0	2.1	1.8	0.0
9	18		11	0.0	0.0	0.0	0.0	0.0
0	0		3	0.0	0.0	0.0	0.0	0.0
0	0		1	12.0	0.0	0.0	0.0	0.0
0	1		4	4.0	0.0	0.0	1.8	0.0
0	1		1	0.0	0.0	0.0	3.6	0.0
0	0		7	16.0	0.0	2.1	8.0	0.0
2	3	10	2					
2	15		11	12.0	0.0	2.1	5.4	1.1
0	5		4	12.0	0.0	4.3	0.9	2.2
0	0	1	4					
3	3	2	8					
0	0	2	5					
			0	0.0	0.0	0.0	0.0	0.0
			0					
		10	9					
3	6	4	9					
			6					
0	1		4	0.0	0.0	4.3	4.5	0.0
1	2	0	2					
0	3		2	0.0	0.0	0.0	0.0	0.0
2	2		2	0.0	0.0	0.0	0.0	0.0
10	20	35	5					
6	11	9	4					
5	19		2	0.0	0.0	0.0	13.4	0.0
0	7	7	11					
0	9	3	1					
0	0	3	0					
3	5	1	5					
2	8	0	4					

0	0		2	12.0	0.0	0.0	1.8	0.0

COMPASS I	COMPASS S	TOY Sum	Migraine_IH	MA_IHS	MO_IHS	Multiple Ch	IBS Dx
		37	1	0	1		
		39	1	1	0		0
		37	1	0	1		1
		27	0	0	0		0
		35	1	0	1		
		31	0	0	0		1
		35	1	1	0		0
		37	1	0	1		
		29	1	1	0		1
		32	1	1	0	1	1
		24					0
		27	1	0	1		1
		37	1	0	1		0
		38	1	0	1		1
		43	1	0	1		0
		23	0	0	0		0
		30	1	0	1		1
		30	1	0	1		
		26	1	0	1		
		40	0	0	0		
			1	0	1		0
			1	0	1		1
		33	1	0	1		1
		32	1	0	1		0
			1	1	0		0
2.7	43.4		1	1	0	0	0
1.3	17.1		1	0	1	0	0
			0	0	0	1	1
1.3	31.2		1	1	0	0	0
2.0	33.8		0	0	0	0	0
1.7	37.9		1	1	0	1	0
2.7	39.3		1	0	1	0	0
2.7	50.5		0	0	0	0	1
3.0	51.0		1	0	1	0	1
2.3	24.9		0	0	0	0	0
3.3	40.9		1	1	0	0	1
3.999996	46.47222		0	0	0	0	1
2.7	42.4		1	0	1	1	1
2.0	32.9		0	0	0	0	0
1.7	5.7		1	1	0	0	0
		30	1	1	0		1
2.0	4.1		0	0	0	0	0
		27	1	1	0		0

3.0	49.6		1	0	1	0	1
		26	1	0	1		1
3.0	38.5		1	0	1	1	0
		37	1	0	1		
3.3	33.3		0	0	0	0	0
			1	0	1	0	1
3.3	13.3		1	0	1	0	0
		45	1	0	1		0
						1	
		27	1	1	0		0
		26	1	0	1		1
3.7	24.8		1	0	1	0	0
		28	1	1	0		1
			0	0	0		1
1.666665	8.273808		1	0	1	0	0
2.0	8.2		0	0	0	0	0
		18	1	0	1		
		14	1	0	1		
		25	1	0	1		0
		24	1	0	1		1
		41	0	0	0		1
		16	1	0	1		0
1.3	30.1		0	0	0	0	1
1.7	41.9		1	0	1	0	0
2.7	38.0		0	0	0	0	0
2.7	32.8		0	0	0	0	0
4.0	26.2		1	1	1	1	1
3.0	14.3		0	0	0	0	0
1.0	30.4		0	0	0	0	0
1.666665	29.27381		0	0	0	0	0
2.7	34.4		0	0	0	0	0
			0	0	0	0	0
2.7	26.2		0	0	0	0	0
		26	1	0	1		1
1.3	30.3		0	0	0	0	0
			1	0	1		
			1	0	1		0
			1	1	0		1
			0	0	0	0	0
3.0	48.5		0	0	0	1	1
2.0	36.2		0	0	0	0	1
		32	1	0	1	0	0
3.7	33.3		1	1	0	0	0
		30	0	0	0		1

1.7	19.2		0	0	0	0	1
		25	0	0	0		1
		31	1	0	1		
		17	0	0	0		
1.0	18.3		0	0	0	0	0
1.0	28.6		0	0	0	0	0
1.7	40.9		0	0	0	0	1
3.0	27.5					0	1
2.3	55.5		1	0	1	0	1
		32	0	0	0		
		30	1	0	1		0
2.3	20.2		1	0	1	0	1
		29					
		33	1	1	0		
		32					
		27					
		25					
4.0	59.2		1	1	0	0	1
2.3	31.8		1	0	1	1	0
2.7	27.5		1	1	0	0	1
2.3	24.9		1	0	1	0	0
2.0	36.1		1	1	0	0	1
3.0	33.9		0	0	0	0	1
3.3	23.8		1	0	1	0	1
3.0	44.6		0	0	0	1	1
4.7	63.1		1	1	0	1	1
2.7	47.0		1	0	1	1	1
2.0	37.4		1	0	1	0	1
4.7	51.3		1	0	1	0	1
		30					
2.0	43.4		0	0	0	0	1
2.7	40.7		0	0	0	0	1
2.0	37.2		1	0	1	0	1
1.7	34.7		0	0	0	0	1
		38	1	0	1		1
		44	1	1	0		1
		25	0	0	0		
		32	0	0	0		
		17	0	0	0		0
		27	0	0	0		
		26					
		29	1	1	1		1
3.3	37.5		1	1	0	1	1

		42	0	0	0		1
		36	0	0	0		1
		34	1	0	1		1
		29					
		27					
		20	1	0	1		
		38	0	0	0		
		33	1	0	1		
		36	1	0	1		1
		25	1	0	1		0
		35	1	0	1		
2.3	29.3		1	0	1	0	0
2.3	53.6		1	0	1	0	1
5.0	67.6		1	1	0	1	1
3.0	44.9		1	0	1	0	1
4.7	60.3		1	1	0	1	0
4.0	63.4		1	1	0	1	1
3.0	36.0		1	1	0	1	1
3.3	59.1		0	0	0	0	1
3.3	47.6		1	1	0	0	1
2.7	52.0		0	0	0	1	1
3.7	51.6		1	1	0	0	0
4.3	64.1		1	0	1	1	1
4.3	58.2		0	0	0	0	0
2.7	67.8		0	0	0	1	1
2.0	18.6		1	1	0	0	1
		37					
1.0	29.5		0	0	0	0	0
		33					
		33					
		21					
		28					
		32	1	0	1		1
2.0	31.0		1	1	0	1	1
4.0	34.8		1	0	1	1	1
		36					
		30	1	0	1		
		40	1	0	1		
4.3	69.1		1	0	1	1	1
4.0	66.6		1	1	0	1	1
		38					
		38	1	0	1		
		35					
3.3	46.5		0	0	0	0	1

3.7	33.1		1	0	1	1	1
2.0	35.4		1	0	1	0	1
		23	1	0	1		
		32					
		33	1	0	1		
		39	1	0	1		
		36	1	0	1		
			1	1	0		
		3					
		27	0	0	0		
4.0	46.1		0	0	0	0	1
2.3	61.2		0	0	0	1	0
		18	0	0	0		0
		42	1	0	1		
		39	1	1	0		
		28	1	0	1		1
			0	0	0		1
		44	1	1	1		1
		26	1	0	1		1
		28					
1.0	30.3		1	0	1	0	0
		37	1	0	1		1
		35	0	0	0		1
		38	0	0	0		0
		38					
		32	0	0	0		
		28					
2.7	49.5		1	1	0	0	1
			1	0	1		1
		39					
		43					
		28					
		28					
		44	0	0	0		
		47					
							1
		32					
		50	1	0	1		
		17					
		42					1
		32					
3.0	43.8		1	1	0	0	1
		36					
		29					

		28					
		26					
		29					
		33	1	1	0		
		38					
		42					
		27					
		30					
		42					
		38					
		35					
		32					
		32	1	0	1		1
		33					
		26					
		32					
		26					
		33					
			0	0	0	0	0
		42	1	0	1		1
		33	0	0	0		1
		24	0	0	0		
		26	1	1	0		1
		18	0	0	0		0
1.7	20.4		0	0	0	0	0
1.7	22.4		0	0	0	0	0
2.0	25.1		0	0	0	0	0
1.7	22.1		0	0	0	0	0
		29	0	0	0		0
0.3	9.3		0	0	0	0	0
		26	1	0	1		
		30	0	0	0		0
		24	0	0	0		0

		23	0	0	0		0
		26	0	0	0		
		13	0	0	0		0
		15	0	0	0		
		12	0	0	0		0
		18	0	0	0		0
		23	0	0	0		0
		15	0	0	0		0
0.7	4.6		0	0	0	0	0
2.3	34.1		1	0	1	0	0
0.0	40.5		0	0	0	0	0
1.7	28.4		1	0	1	0	0
0.7	20.2		0	0	0	0	0
0.7	20.6		0	0	0	0	0
1.0	1.0		0	0	0	0	0
0.0	0.0		0	0	0	0	0
0.0	12.0		0	0	0	0	0
1.0	6.8		0	0	0	0	0
1.7	5.2		0	0	0	0	0
0.7	26.8		0	0	0	0	0
		24	0	0	0	0	0
1.7	22.3		0	0	0	0	0
1.0	20.4		0	0	0	0	0
		15					
		14	0	0	0		0
		18					
0.3	0.3		0	0	0	0	1
			0	0	0		0
		27	1	1	1		
		21	0	0	0		0
		18	0	0	0		0
1.3	10.1		0	0	0	0	0
		18	0	0	0		0
1.0	1.0		0	0	0	0	0
0.7	0.7		0	0	0	0	0
		33	0	0	0		1
		31					
1.7	15.1		1	0	1	0	0
		33	0	0	0		0
		22	0	0	0		0
		20	0	0	0		
		10	0	0	0		
		11	0	0	0		

0.0	13.8	0	0	0	0	0
		0	0	0	0	0
		0	0	0	0	0

IBS diarrhe	IBS constip	IBS mixed	IBS Rome I	IBS Rome II	IBS Rome III		GAD_ICD1	Depr_DSM
0	0	0						
0	1	0						
0	0	0						
0	0	1						
0	0	0						1
0	1	0						
0	0	1						
0	0	0						
1	0	0						
0	0	0						
0	0	1						
0	0	0						
0	0	0						
0	1	0						
0	0	0						
1	0	0						
0	1	0						
0	0	0						
0	0	0						
0	0	0	0	0	0			
0	0	0	0	0	0		1	1
0	0	0	1	0	0			
0	0	0	0	0	0			
0	0	0	0	0	0			
0	0	0	0	0	0			
0	0	0	0	0	0			
1	0	0	1	1	1			
0	0	1	1	1	1			
0	0	0	0	0	0			
0	1	0	1	1	1			
1	0	0	1	1	1			
0	0	1	1	1	1		1	0
0	0	0	0	0	0			
0	0	0	0	0	0		0	0
0	0	1						
0	0	0	0	0	0		0	0
0	0	0						

1	0	0	1	1	1		
0	0	1					
0	0	0	0	0	0		
0	0	0	0	0	0		
0	1	0	1	1	1		
0	0	0	0	0	0	0	0
0	0	0					
0	0	0					
0	0	1					
0	0	0	0	0	0		
0	0	1					
0	0	1					
0	0	0	0	0	0		
0	0	0	0	0	0	0	0
0	0	0					
0	0	1					
0	1	0					
0	0	0					
0	0	1	0	1	1		
0	0	0	0	0	0		
0	0	0	0	0	0		
0	0	0	0	0	0		
1	1	1	1	1	1		
0	0	0	0	0	0		
0	0	0	0	0	0		
0	0	0	0	0	0		
0	0	0	0	0	0	0	0
0	0	0	0	0	0	0	0
0	0	0	0	0	0		
0	0	1					
0	0	0	0	0	0	0	0
0	0	0					
0	1	0					
0	0	0	0	0	0	0	0
0	1	0	1	1	1	1	1
1	0	0	1	1	1	0	0
0	0	0	0	0	0		
0	0	0	0	0	0	1	0
0	0	1					

1	0	0	1	1	1	0	0
1	0	0					
0	0	0	0	0	0	0	0
0	0	0	0	0	0	1	0
0	0	1	1	1	1		
1	0	0	1	1	1		
1	0	0	1	1	1	0	0
0	0	0					
1	0	0	1	1	1	0	0
1	0	0	1	1	1	1	0
0	0	0	0	0	0	0	0
0	0	1	1	1	1	1	0
0	0	0	0	0	0	0	1
0	1	0	1	1	1	0	0
0	0	1	1	1	1	1	0
0	0	1	1	0	0	0	0
0	0	1	1	1	1	0	0
0	0	1	1	1	1	1	0
1	0	0	1	1	1	0	0
0	1	0	1	1	1	1	0
0	0	1	1	1	1	1	1
0	1	0	1	1	1	0	0
1	0	0	1	0	0	1	0
1	0	0	1	1	1	0	0
0	0	1	1	1	1	0	0
1	0	0					
0	0	1					
0	0	0					
1	0	0					
0	1	0	1	1	0	1	0

0	0	1
1	0	0
0	0	1

0	0	1
0	0	0

0	0	0	0	0	0	0	0
1	0	0	1	1	1	1	0
0	0	1	1	1	1	1	1
1	0	0	1	1	1	1	0
0	0	0	0	0	0	1	1
1	1	1	1	1	1	1	0
0	0	1	1	1	1	0	0
1	0	0	1	1	1	0	0
0	0	1	1	1	1	0	0
0	0	1	0	1	0		
0	0	0	0	0	0		
0	0	1	1	1	1		
0	0	0	0	0	0		
0	0	1	1	1	1		
0	0	1	1	1	1		

0	0	0	0	0	0		

0	1	0					
0	0	1	1	1	1	0	0
0	0	1	1	1	1		

0	0	1	1	1	1		
0	0	1	1	1	1	1	1

1	0	0	1	0	0	1	1

0	0	1	1	1	1		
0	0	1	1	1	1		
1	0	0	1	1	1	0	0
0	0	0	0	0	0	1	1
0	0	0					
1	0	0					
0	0	1					
1	0	0					
1	0	0					
0	0	0	0	0	0	0	0
0	0	1					
0	1	0					
0	0	0					
0	0	1	1	0	1	0	0
0	0	1					
0	1	0					
1	0	0					
1	0	0	1	1	1		

0	0	1						
0	0	0	0	0	0		1	0
0	0	1						
1	0	0						
0	0	1						
0	0	0						
0	0	0	0	0	0			
0	0	0	0	0	0			
0	0	0	0	0	0			
0	0	0	0	0	0			
0	0	0						
0	0	0	0	0	0		0	0
0	0	0						
0	0	0						

0	0	0					
0	0	0					
0	0	0					
0	0	0					
0	0	0					
0	0	0					
0	0	0	0	0	0		
0	0	0	0	0	0		
0	0	0	0	0	0		
0	0	0	0	0	0		
0	0	0	0	0	0		
0	0	0	0	0	0		
0	0	0	0	0	0		
0	0	0	0	0	0		
0	0	0	0	0	0		
0	0	0	0	0	0	0	0
0	0	0	0	0	0		
0	0	0	0	0	0		
0	0	0	0	0	0	0	0
0	0	0	0	0	0		
0	0	0	0	0	0		
0	0	0					
1	0	0	1	1	1	0	0
0	0	0					
0	0	0					
0	0	0					
0	0	0	0	0	0		
0	0	0					
0	0	0	0	0	0		
0	0	0	0	0	0		
0	0	1					
0	0	0	0	0	0		
0	0	0					
0	0	0					

0	0	0	0	0	0
0	0	0	0	0	0
0	0	0	0	0	0

GAD_DSM	PTSD	MPTSD	PCL-C	∑GAD7	GAD7≥10	STAI	MASQ Gen
						25	
				0	0	29	
				12	1	47	
				0	0	24	
				6	0	65	
		55		0	0	24	
						49	
						59	
						43	
						49	
		78		11	1	58	
				17	1	41	
				14	1	69	
				2	0	49	
				8	0	64	
						27	
				0	0	29	
				2	0	56	
						29	
				17	1		
				10	1		
				1	0	26	
				14	1	52	
	0	74	29	8	0		30
1	1	83	44	7	0		18
	0	61	27	0	0		12
	0		26	2	0		12
	1	79	44	7	0		25
	0		22	0	0		16
	0		26	2	0		16
	0	66	24	1	0		12
	1	87	62	15	1		35
	1		43	8	0		34
	1		53	7	0		23
	0	67	41	4	0		26
	1	79	57	18	1		36
	0	67	29	2	0		18
0	0	44	18	0	0		12
				0	0	30	
0	0	114	68	10	1		37
		76		4	0	33	

	0	77	24	5	0		15
				5	0	45	
	0		28	2	0		25
				9	0	71	
	0	83	43	6	0		27
	0						
0	0	64	27	2	0		17
				7	0	62	
				19	1	63	
				4	0	33	
	1	76	37	5	0		14
						34	
	0	71	24	1	0		19
0	0	64	19	1	0		13
				9	0	43	
				0	0	20	
				5	0	43	
				9	0	36	
						55	
				1	0	43	
			24	8	0		24
	0	69	32	0	0		13
	0		23	1	0		16
	0		31	7	0		21
	0		28	4	0		16
	0		19	0	0		13
	0		25	3	0		20
	0	78	25	4	0		24
0	0	54	23	4	0		15
0	0						
	0	71	28	5	0		15
						52	
0	0	54	17	2	0		19
0	0						
1	1	104	53	9	0		20
0	1	116	50	6	0		22
		67		1	0	49	
1	1	117	51	8	0		17
		104		4	0	62	

0	0	46	18	0	0		13
		85		1	0	40	
		103		12	1	23	
		56		1	0	20	
0	0	49	19	0	0		11
0	0	63	28	1	0		12
	0		29	5	0		23
	1	97	40	8	0		27
0	1	73	25	3	0		15
		79		16	1	66	
		83		8	0	60	
0	0	58	22	6	0		19
		95		9	0	60	
		77		2	0	48	
		79		16	1	66	
		80		0	0	53	
		75		2	0	40	
1	1	116	47	5	0		19
0	1	102	50	5	0		28
0	1	117	44	8	0		28
0	0	97	49	17	1		28
0	0	75	43	5	0		27
1	1	97	73	7	0		26
0	0	99	55	11	1		30
0	1	61	41	8	0		20
0	1	102	76	21	1		42
0	1	124	73	7	0		31
1	0	93	53	9	0		27
1	1	109	70	21	1		37
		90		13	1	59	
0	0	116	63	14	1		35
1	0	70	23	5	0		14
0	0	68	28	9	0		20
0	1	84	38	9	0		12
		122		10	1	54	
		103		13	1	38	
		106		12	1	48	
		135		15	1	65	
		126		16	1	61	
		67		3	0	38	
		67		0	0	20	
		71		14	1	50	
1	1	101	51	21	1		26

		88		12	1	68	
		120		21	1	68	
		66		2	0	40	
		89		14	1	49	
		98		4	0	26	
		98		2	0	47	
		95		15	1	68	
		71		7	0	64	
				3	0	20	
		63		1	0	38	
		72		7	0	41	
0	0	86	47	4	0		36
1	1	119	65	18	1		32
1	1	168	85	21	1		31
1	1	98	64	7	0		29
1	1	140	74	21	1		41
1	1	116	65	10	1		21
0	1	106	68	15	1		25
0	0	100	62	12	1		39
0	0	93	44	0	0		19
	1		52	6	0		13
	0		33	4	0		18
	1		59	8	0		26
	1		63	13	1		33
	1	115	54	13	1		30
			29	1	0		16
		80		7	0	36	
	0		38	2	0		16
		100		10	1	54	
		75		10	1	63	
		87		19	1	49	
		79		12	1	69	
		108		17	1	68	
0	1	74	47	5	0		15
	1		72	12	1		38
		90		14	1	58	
		60		0	0	23	
		81		10	1	70	
	0	77	37	6	0		28
1	1	115	60	20	1		29
		95		15	1	58	
		95		15	1	58	
		120		16	1	63	
0	1	117	64	18	1		40

	0		59	16	1		27
	0	96	38	13	1		24
		108		9	0	58	
		78		7	0	50	
				1	0	29	
		129		17	1	60	
		112		11	1	53	
		89		7	0	37	
		68		5	0	43	
0	1	91	51	9	0		27
1	1	116	61	16	1		34
		55		5	0	34	
		144		13	1	58	
		124		13	1	58	
				2	0	35	
1							
		156		21	1	80	
		87		13	1	43	
		93		16	1	63	
0	1	104	47	0	0		13
		123		15	1	80	
		125		11	1	46	
		92		9	0	42	
		92		9	0	42	
		122		15	1		
		98		11	1	53	
0	0	63	28	5	0		16
				2	0		
		124		13	1	58	
		120		20	1	58	
		60		2	0	37	
				2	0	35	
		127		16	1	23	
		154		21	1	76	
		122		15	1		
		168		21	1	75	
		126		16	1	61	
						21	
		73		6	0	49	
	1	85	35	2	0		21
		124		14	1	59	
		71		14	1	50	

		93		16	1	44	
		57		8	0	53	
		55		0	0	20	
		100		10	1	54	
		75		16	1	49	
		144		13	1	58	
		68		5	0	43	
		60		0	0	23	
		88		12	1	58	
		95		15	1	68	
		72		7	0	41	
		108		17	1	68	
						55	
		71		7	0	64	
		67		0	0	31	
		86		9	0	41	
		55		4	0	42	
		77		2	0	43	
0	0						
				4	0	36	
				1	0	41	
						45	
						35	
				1	0	29	
	0		22	0	0		13
	0		23	1	0		12
	0		21	0	0		13
	0	61	19	0	0		11
				14	1	52	
0	0	66	30	1	0		11
						39	
						32	
		78		10	1	48	

		57		3	0	20	
				1	0	31	
		44		0	0	20	
		50		0	0	24	
				0	0	37	
		91		11	1	57	
		63		0	0	35	
		56		0	0	25	
	0	71	17	0	0		10
	0		29	4	0		18
	1		76	21	1		41
	0	70	29	3	0		25
	0		17	4	0		20
	0		19	0	0		10
	0		20	0	0		10
	0		26	0	0		14
	0	59	17	0	0		10
0	0	55	17	0	0		11
	0	68	21	2	0		20
	0	73	32	10	1		22
0	0	56		1	0	20	
	0		19	0	0		13
	0	61	18	0	0		10
				0	0	23	
		55		1	0	25	
		48		0	0	20	
0	0	55	22	3	0		14
		70		11	1	43	
		68		0	0	45	
		71		2	0	22	
				0	0	20	
	0		17	3	0		10
		73		0	0	25	
	0	53	17	0	0		16
	0	49	18	0	0		14
						33	
		78		3	0	47	
	0	61	29	11	1		24
		49		0	0	36	
		79		0	0	23	
				0	0	23	
		49		2	0	24	
				6	0	30	

0	20	1	0	16
0				
0				

MASQ Anhe	MASQ Anxious Arousal	CESD Sum	CESD Soma	CESD Depr	CESD Anhe	CESD Inter	CESD≥16=
		14	12	1	1	0	0
		23	15	3	5	0	1
		24	14	3	7	0	1
		8	8	0	0	0	0
		40	16	13	10	1	1
		5	4	0	1	0	0
		30	14	10	4	2	1
		19	12	4	3	0	1
		26	9	7	8	2	1
		13	3	3	7	0	0
		31	10	12	7	2	1
		40	14	16	9	1	1
		36	13	16	7	0	1
		25	15	3	7	0	1
		21	11	4	5	1	1
		17	12	5	0	0	1
		14	7	1	6	0	0
		26	7	10	9	0	1
		27	13	8	6	0	1
		17	10	1	5	1	1
		37	17	10	6	4	1
38	21	27	10	8	8	1	1
36	18	23	7	6	8	2	1
20	25	15	11	0	4	0	0
28	16	5	3	0	2	0	0
29	17	19	11	3	5	0	1
20	24	13	7	3	0	3	0
26	26	20	13	3	4	0	1
19	25	12	9	1	2	0	0
43	20	40	13	15	9	3	1
45	20	26	10	7	9	0	1
25	21	32	18	6	7	1	1
34	26	19	13	4	2	0	1
37	34	50	18	18	10	4	1
29	20	21	15	1	5	0	1
27	19	0	0	0	0	0	0
		14	7	1	6	0	0
48	19	32	12	7	10	3	1
		26	12	3	11	0	1

31	31	20	6	8	6	0	1
		14	10	2	2	0	0
37	34	22	15	3	4	0	1
		22	12	3	6	1	1
45	24	30	15	7	7	1	1
		21	13	5	3	0	1
42	24	22	13	4	5	0	1
		31	13	13	5	0	1
		25	11	8	0	6	1
		10	5	2	3	0	0
40	17	17	10	5	2	0	1
		10	10	0	0	0	0
		19	10	4	4	1	1
34	14	14	7	4	3	0	0
32	18	16	8	2	6	0	1
		14	11	0	2	1	0
		6	6	0	0	0	0
		20	9	6	4	1	1
		24	8	11	5	0	1
		23	11	6	6	0	1
		19	11	3	5	0	1
29	26	6	2	2	1	1	0
32	23	22	9	7	5	1	1
26	22	9	7	0	2	0	0
38	17	11	8	3	0	0	0
19	19	10	8	2	0	0	0
19	15	4	4	0	0	0	0
32	19	5	3	1	1	0	0
37	15	10	5	0	5	0	0
30	22	12	8	0	4	0	0
		17	4	2	7	4	1
39	22	15	10	3	2	0	0
		20	13	2	5	0	1
35	19	8	5	0	3	0	0
39	17	23	13	4	4	2	1
47	18	20	9	5	6	0	1
		18	8	2	7	1	1
10	27	11	10	0	1	0	0
		32	12	9	9	2	1

24	13	5	4	1	0	0	0
		22	10	6	6	0	1
		27	12	5	6	4	1
		1	1	0	0	0	0
11	11	3	3	0	0	0	0
25	19	9	2	2	5	0	0
41	23	18	6	5	7	0	1
33	25	22	5	7	9	1	1
42	19	16	10	0	6	0	1
		25	10	8	6	1	1
		38	14	10	12	2	1
35	18	13	9	1	3	0	0
		30	15	7	6	2	1
		23	7	8	5	3	1
		25	10	8	6	1	1
		18	2	5	11	0	1
		24	10	9	4	1	1
26	29	12	9	1	2	0	0
42	27	32	14	9	8	1	1
43	28	33	13	9	11	0	1
41	23	24	14	3	7	0	1
28	22	8	8	0	0	0	0
38	24	26	10	7	8	1	1
34	23	28	13	8	7	0	1
20	20	24	12	4	8	0	1
49	36	47	16	16	11	4	1
40	23	38	15	9	11	3	1
31	27	25	13	5	5	2	1
41	31	33	11	9	10	3	1
		25	16	5	4	0	1
34	27	27	10	6	8	3	1
42	22	8	7	0	1	0	0
37	29	24	13	2	9	0	1
31	16	15	6	2	5	2	0
		28	12	10	4	2	1
		33	14	9	8	2	1
		34	15	11	8	0	1
		35	12	9	9	5	1
		31	10	10	9	2	1
		12	11	1	0	0	0
		14	7	1	5	1	0
		20	9	6	5	0	1
48	29	34	16	9	7	2	1

		21	14	3	4	0	1
		52	20	16	12	4	1
		17	10	4	3	0	1
		18	8	1	7	2	1
		13	10	0	3	0	0
		29	14	7	8	0	1
		37	13	11	9	4	1
		27	14	7	4	2	1
		15	8	4	3	0	0
		14	8	2	2	2	0
		18	12	4	1	1	1
43	23	21	9	4	7	1	1
41	29	43	19	13	8	3	1
38	30	54	20	19	9	6	1
38	24	34	12	13	7	2	1
46	39	48	17	15	11	5	1
42	22	37	20	7	5	5	1
42	27	32	14	6	12	0	1
41	26	27	16	4	7	0	1
36	32	26	16	4	6	0	1
17	25	13	9	0	3	1	0
28	31	26	15	7	3	1	1
42	29	29	14	6	8	1	1
18	38	29	14	10	1	4	1
33	27	28	15	6	6	1	1
39	19	12	9	1	2	0	0
		19	11	4	4	0	1
20	27	24	15	3	5	1	1
		39	17	10	8	4	1
		23	13	5	3	2	1
		35	12	12	9	2	1
		42	15	13	10	4	1
		49	19	19	7	4	1
25	23	12	11	1	0	0	0
38	31	37	13	12	9	3	1
		33	12	12	5	4	1
		5	4	0	1	0	0
		29	12	5	12	0	1
33	24	34	18	5	9	2	1
32	26	22	10	6	6	0	1
		45	17	15	11	2	1
		45	17	15	11	2	1
		36	13	12	10	1	1
44	38	43	15	13	11	4	1

42	27	34	15	8	11	0	1
47	26	26	12	5	9	0	1
		36	14	10	8	4	1
		23	10	4	8	1	1
		23	9	7	4	3	1
		24	10	9	3	2	1
		34	15	11	6	2	1
		15	8	3	4	0	0
		23	8	5	8	2	1
30	26	25	10	6	7	2	1
43	39	46	18	14	10	4	1
		17	9	0	8	0	1
		30	14	7	7	2	1
		37	17	9	9	2	1
		14	7	5	2	0	0
		19	14	2	2	1	1
		57	20	21	12	4	1
		30	15	8	5	2	1
		31	15	8	7	1	1
27	14	15	9	1	1	4	0
		47	14	16	12	5	1
		27	14	5	4	4	1
		18	13	3	1	1	1
		18	13	3	1	1	1
		35	15	9	9	2	1
		30	15	7	6	2	1
39	20	16	12	0	4	0	1
		37	17	9	9	2	1
		44	19	14	9	2	1
		14	10	3	1	0	0
		12	7	5	0	0	0
		27	15	5	3	4	1
		46	17	16	9	4	1
		12	10	0	2	0	0
		35	15	9	9	2	1
		57	21	21	9	6	1
		31	10	10	9	2	1
		29	17	6	6	0	1
		25	12	7	5	1	1
32	18	25	15	5	4	1	1
		35	15	12	8	0	1
		20	9	6	5	0	1

		30	7	8	9	6	1
		20	9	6	5	0	1
		9	6	0	3	0	0
		39	17	10	8	4	1
		18	8	0	10	0	1
		30	14	7	7	2	1
		23	8	5	8	2	1
		5	4	0	1	0	0
		21	14	3	4	0	1
		37	13	11	9	4	1
		18	12	4	1	1	1
		49	19	19	7	4	1
		28	11	8	6	3	1
		27	14	7	4	2	1
		14	7	1	5	1	0
		20	13	4	3	0	1
		12	5	5	0	2	0
		12	9	1	2	0	0
		23	7	8	5	3	1
		46	19	14	11	2	1
		22	10	9	3	0	1
		7	7	0	0	0	0
		5	0	0	5	0	0
		24	14	5	5	0	1
		10	7	2	1	0	0
		13	7	0	3	3	0
25	13	11	6	3	1	1	0
29	14	21	11	4	6	0	1
16	15	11	8	0	0	3	0
26	12	12	7	0	5	0	0
		23	10	6	4	3	1
30	12	8	4	2	2	0	0
		13	8	4	1	0	0
		6	2	4	0	0	0
		18	6	6	6	0	1

		3	3	0	0	0	0
		3	1	2	0	0	0
		4	1	0	3	0	0
		0	0	0	0	0	0
		5	1	2	2	0	0
		10	3	2	5	0	0
		6	2	0	4	0	0
		2	0	0	2	0	0
50	14	12	7	1	2	2	0
30	20	11	7	3	1	0	0
48	20	48	16	16	10	6	1
26	18	25	4	10	8	3	1
40	15	9	0	2	4	3	0
20	14	9	3	0	4	2	0
38	15	4	0	0	4	0	0
23	11	1	1	0	0	0	0
27	11	1	1	0	0	0	0
30	11	4	1	0	3	0	0
38	13	9	3	2	4	0	0
15	15	6	2	3	0	1	0
		0	0	0	0	0	0
18	12	1	1	0	0	0	0
28	14	4	0	1	3	0	0
		8	1	0	7	0	0
		1	1	0	0	0	0
		8	1	0	7	0	0
14	16	6	0	2	4	0	0
		14	6	3	4	1	0
		18	6	5	5	2	1
		1	1	0	0	0	0
10	10	0	0	0	0	0	1
		0	0	0	0	0	0
17	11	0	0	0	0	0	0
21	17	4	3	1	0	0	0
		13	1	0	12	0	0
		25	8	8	9	0	1
31	14	14	5	4	5	0	0
		22	13	5	4	0	1
		6	3	0	3	0	0
		1	0	0	1	0	0
		7	4	0	3	0	0
		4	1	0	3	0	0

22	13		29	14	10	1	4	1

BDI_Score	BDI_grade	BDI_Negati	BDI_Anhed	BDI_Somatic_3	PBPI Myste	PBPI Perma	PBPI Const
12	Mild	3	8	1	1.50	1.40	-0.75
3	Minimal	0	2	1	-1.75	-0.40	2
20	Moderate	5	13	2	1.25	0.80	2
3	Minimal	0	3	0	-0.50	-0.20	0
5	Minimal	0	2	3	-0.75	1.60	1
					0.00	-0.80	0
4	Minimal	0	3	1	-1.00	0.00	1.75
25	Moderate	12	11	2	2.00	1.20	2
17	Mild	4	11	2	2.00	0.00	0.5
13	Mild	4	9	0	1.75	-0.60	1
9	Minimal	4	4	1	0.75	1.40	1.25
2	Minimal	0	1	1	-1.50	1.20	2
31	Severe	15	14	2	0.75	1.00	2
13	Mild	3	8	2	1.00	2.00	1.75
27	Moderate	13	12	2	1.50	1.60	-0.25
11	Mild	7	4	0	2.00	1.60	-1.25
8	Minimal	2	6	0	0.00	-1.20	-0.75
14	Mild	0	10	4	0.25	1.80	0
					1.75	1.00	-1
5	Minimal	2	3	0	-1.75	-0.40	0.25
12	Mild	1	9	2	1.50	0.60	-0.75
20	Moderate	9	9	2	1.75	1.00	0
5	Minimal	0	4	1	-2.00	-1.80	-1.75
22	Moderate	9	12	1	1.25	-0.20	0

17	Mild	4	8	5	1.00	0.80	-0.5
10	Mild	0	7	3	1.50	1.00	-1.5
31	Severe	17	13	1	1.25	-0.6	0.25
17	Mild	4	8	5	1.00	1.20	1.75
17	Mild	6	10	1	1.50	0.80	
14	Mild	2	9	3	-0.50	1.00	1.5
3	Minimal	0	3	0	0.75	-0.80	1
5	Minimal	4	1	0			
1	Minimal	1	0	0	0.00	0.60	-0.5
13	Mild	5	8	0	-1.75	-1.60	-2
19	Moderate	6	12	1	2.00	0.60	1.5
14	Mild	2	9	3	1.75	1.4	-1
11	Mild	3	4	4	0.00	0.20	2
13	Mild	8	3	2	0.50	-0.20	0.5
48	Severe	30	15	3	1.00	1.20	1

16	Mild	5	9	2	1.00	1.40	2
17	Mild	6	6	5	1.75	1.2	1.75
4	Minimal	0	0	4	-2	-2	-2
22	Moderate	12	9	1	2	1.6	1.25
30	Severe	14	9	7	-0.25	1.40	1.75
					1	1.2	1.5
10	Mild	1	6	3	-0.25	1.2	2
22	Moderate	12	9	1	2	1.6	1.25
17	Mild	5	8	4	-0.25	1.4	2
12	Mild	3	8	1	0.75	1.2	2
17	Mild	5	11	1	-0.5	-0.2	1.25
28	Moderate	12	13	3	0.25	2.00	2
22	Moderate	11	6	5	0.00	0.20	2
12	Mild	2	7	3	2	1	1
32	Severe	15	13	4	-2.00	-1.80	1.75
32	Severe	13	13	6	1	-0.6	1
12	Mild	0	7	5	1	-1	0
32	Severe	14	14	4	-1	1.2	1.75
11	Mild	4	3	4	0.25	-1.8	-0.25

19	Moderate	4	10	5	0.75	1	-1
34	Severe	14	18	2	0.25	1.00	2
3	Minimal	0	0	3	-1.00	1.00	1.25
16	Mild	6	7	3	1.25	0.8	1.25
12	Mild	4	7	1	0.75	1.8	2
30	Severe	10	13	7	1.25	2	1.75
25	Moderate	12	9	4	-2.00	-1.80	1.25
19	Moderate	5	12	2	1	1	2
7	Minimal	3	3	1	-2.00	-1.40	-1.75
14	Mild	2	10	2		-1.00	-2
					0	-0.4	0.5

12	Mild	3	7	2	0	0	-1
23	Moderate	10	10	3	1.5	0.8	2
24	Moderate	9	13	2	1	1	2
29	Moderate	13	12	4	1	0.4	1.75
27	Moderate	16	10	1	1.75	2	0.25
38	Severe	17	14	7	0	2	1.5
16	Mild	7	8	1	0.75	0.2	1
7	Minimal	1	5	1	0	1	0.5
24	Moderate	11	8	5	2	1.2	0.75
32	Severe	14	14	4	1	1.8	2
0	Minimal	0	0	0	1	1.8	2
30	Severe	14	14	2	0.5	1.2	1.75

24	Moderate	13	9	2	1	1	1
14	Mild	3	11	0	-1	1.2	1
13	Mild	6	5	2	0.50	1.00	2
25	Moderate	12	9	4	1.5	-0.2	0.5
15	Mild	2	10	3	1.75	0.6	2
12	Mild	3	8	1			
13	Mild	5	6	2	1	-2	1.25
10	Mild	1	5	4	-2.00	0.40	-2
26	Moderate	8	14	4	1.75	2	2
20	Moderate	6	11	3	0.75	0	2
7	Minimal	1	4	2	0.75	-0.20	1.5
0	Minimal	0	0	0	1.75	1.80	1.25
24	Moderate	9	10	5	0.25	1.00	1.5
30	Severe	14	11	5	1	1.2	1.25
25	Moderate	8	14	3	1.50	1.20	1.5
15	Mild	2	12	1	-0.50	1.40	1.25
15	Mild	2	7	6	1.00	-0.60	0.5
15	Mild	2	7	6	1	-0.6	0.5
23	Moderate	11	11	1	1.75	2	2
30	Severe	15	10	5	1	1.8	1.75
20	Moderate	6	11	3	0.75	0	2
43	Severe	25	16	2	0	2	2
14	Mild	3	8	3			
7	Minimal	1	4	2	0.75	-0.2	1.5
11	Mild	4	6	1	0.75	2	2
40	Severe	18	17	5	0.75	2	1.75
23	Moderate	11	11	1	1.75	2	2
46	Severe	23	17	6	1.25	1.2	2
32	Severe	13	13	6	1	-0.6	1
12	Mild	2	6	4	0.25	-1.00	2
13	Mild	5	5	3	0.75	1.6	1.75
33	Severe	13	15	5	0.5	1.8	1.75
11	Mild	4	3	4			

12	Mild	2	9	1	2	0.4	1
11	Mild	4	3	4	-1.25	1.4	-0.75
4	Minimal	0	3	1	1.5	0.6	1
23	Moderate	10	10	3	1.5	0.8	2
10	Mild	2	8	0	1	1.6	1.25
26	Moderate	8	14	4	1.75	2	2
13	Mild	5	6	2	1	-2	1.25
5	Minimal	0	4	1	0	1	0.5
19	Moderate	4	10	5	0.75	1	-1
25	Moderate	12	9	4	1.5	0.2	1.25
13	Mild	4	8	1	0	-0.4	0.5
38	Severe	17	14	7	0	2	1.5
					1.75	2	1.25
19	Moderate	5	12	2	1	1	2
10	Mild	1	6	3	-1	1.2	1.75
12	Mild	3	7	3			
5	Minimal	3	2	0	-1	0.6	-0.5
15	Mild	3	8	4	1	-1.4	2
11	Mild	4	6	1	-0.25	1.2	2
5	Minimal	0	4	1	-0.50	0.20	0.5
31	Severe	15	14	2	1.00	-0.80	-1.75
18	Mild	3	10	5	1.50	0.80	1
9	Minimal	4	4	1	-1.00	0.20	-0.5
14	Mild	3	9	2	-2.00	-1.20	-2
14	Mild	5	9	0	1.50	-1.00	1.25
10	Mild	3	5	2	-1.25	0.60	1.5
0	Minimal	0	0	0	-1.25	-0.20	-1
17	Mild	8	8	1	-1.25	1.00	-0.5

0	Minimal	0	0	0	-1.00	-1.20	-2
1	Minimal	1	0	0			
0	Minimal	0	0	0	-2.00	-2.00	-2
5	Minimal	0	4	1	-2	-1.2	-2
0	Minimal	0	0	0	-2.00	-1.80	-2
8	Minimal	3	4	1	0.5	-0.2	1.25
2	Minimal	0	1	1	-2	-1.8	-2
0	Minimal	0	0	0	-2.00	0.40	-1
2	Minimal	1	1	0	-2.00	-1.80	-1.75
1	Minimal	0	1	0	-2	-0.8	-1.75
0	Minimal	0	0	0	-2.00	-2.00	-2
1	Minimal	0	1	0			
					-1.75	-1.20	-1.5
					1	1	1
0	Minimal	0	0	0	-2.00	0.40	-2
					-2	-2	-2
3	Minimal	0	0	3			
16	Mild	4	12	0	1.75	-0.20	0.5
13	Mild	5	6	2	1.5	1.6	1
2	Minimal	1	1	0	-1.00	-1.40	-1.75
1	Minimal	0	1	0	-1.00	0.20	0.5
0	Minimal	0	0	0	-2.00	-2.00	-2
3	Minimal	1	2	0	-1.75	-1.4	-2
25	Moderate	11	11	3	-2.00	-1.60	-2

PBPI Selfblame	BCP Interna	BCP Power	BCP Chance	Happenin	CPSSsum	Adult_Conf
-2	12	7	13		64	2
-2	10	4	3		63	3
-1	12	13	14		69	13
-1	8	13	13		45	0
-0.33333	11	12	20		74	19
-2	5	12	13			7
-2	8	16	14		43	0
-2	14	12	10		94	4
-2	6	15	8		74	12
-2	11	11	11		77	2
-2	9	12	12		58	10
-2	11	14	10		68	13
-1.33333	8	14	8		84	15
-2	8	24	21		79	16
-2	10	10	9		60	6
-2	13	8	13		39	8
-2	7	11	8		58	5
-2	12	12	13		63	2
-2	9	13	13		64	16
-2	11	17	5		52	16
-1	12	12	12		47	2
-1.33333	10	19	14		70	19
-2	6	15	7		23	0
-2	10	14	8		37	3

-2	10	13	16	58	7
1	22	14	14	89	10
-1.33333	14	20	15	60	0
-2	7	9	17	57	10
-0.33333	14	7	10	64	5
-2	10	8	15	45	3
-2	12	13	16	76	0
					0
-1.33333	10	8	8	49	5
1	21	6	9	31	6
-2	16	14	11	78	10
-1.66667	11	12	6	75	12
-2	6	17	10	74	7
-2	11	14	4	50	10
-1.33333	11	13	13	55	8

-2	6	10	6	58	0
-2	7	17	18	21	13
-2	19	5	10	27	1
-2	11	12	12	63	14
-1.33333	15	15	12	73	12
-1	13	16	10	63	5
-1.66667	14	10	15	48	20
-2	11	12	12	63	14
					5
-1	16	14	16	52	16
-0.33333	17	4	10	34	20
-1.66667	12	19	12	54	1
-2	5	17	8	71	6
-1	18	13	10	77	0
-1.66667	8	18	12	65	4
-2	5	13	16	74	3
-1	9	16	8	73	14
-1	12	14	9	46	0
-2	9	7	10	58	4
-2	6	9	5	52	7

-2				73	10
-2	7	19	9	62	13
-1	14	18	12	53	4
-1	8	13	15	69	9
-1	9	10	9	55	1
-2	7	14	17	93	10
-2	18	10	9	71	6
-2	8	10	11	78	5
-2	15	4	4	51	9
-1.66667	14	9	14	48	9
-1.66667	13	6	6	88	12
-1.66667	15	8	9	76	7
-1.66667	14	20	13	66	1
-1	9	16	9	80	9
-1	6	12	7	82	4
1.666667	26	14	21	95	18
-2	10	11	9	99	11
-1	13	7	11	95	3
-1	14	10	11	58	2
-0.33333	14	6	9	84	13
-2	10	19	18	73	4
-2	10	19	18	73	4
-1	12	16	16	72	13

-1	12	10	13	68	10
-2	6	4	12	72	3
-2	19	19	6	75	8
-2	11	15	13	72	13
-1.66667				55	7
	6	4	10	45	
-2	15	20	12	37	5
-2	22	5	7	25	3
2	11	17	12	99	18
-2	6	19	14	85	11
-2	10	10	5	65	18
0.666667	22	12	14	101	18
-1.66667	11	16	15	87	12
-1	11	12	11	64	3
-1.66667	5	16	9	88	19
-2	5	5	9	58	13
-1.33333	11	18	12	47	2
-1.33333	11	18	12	47	2
-1.66667	7	19	11	48	11
-2	8	14	9	64	10
-2	6	19	14	85	11
-1.66667	7	16	19	85	0
	10	9	6	64	12
-2	10	10	5	65	18
-2	6	21	19		0
2	19	22	18	105	16
-1.66667	7	19	11		11
2	10	15	12	97	11
-1	9	16	8	73	14
-2	14	14	14	72	0
-2	17	12	16		17
0.666667	21	14	20	88	16
				52	7

-2	6	13	16	69	7
-2	9	7	5	37	1
-1.66667	9	13	9	67	0
-1.66667	14	20	13	66	1
-2	6	18	11	69	5
2	11	17	12	99	18
-2	15	20	12	37	5
-1	14	10	11	58	2
-2	14	14	10	73	10
-2	18	10	9	71	6
-1.66667	13	6	6	88	12
-2	10	11	9	99	11
0.333333	11	11	23	75	11
-2	8	10	11	78	5
-2	9	7	10	62	4
-0.33333	16	14	9	54	4
-2	9	14	9	48	4
-1.66667	14	10	15	52	20
-2	16	12	11	61	3
-2	13	10	12	37	6
-2	10	12	12	62	3
-1	20	5	7	0	5
-2	6	0	0	21	10
-0.33333	19	12	13	73	14
-2	7	16	11	47	0
-2	11	6	19	45	0
-1.33333	14	14	18	21	19

-2	15	10	8	34	7
	24	12	12	36	1
-2	20	8	16	21	0
-2	15	18	14	21	0
0	25	7	6	21	9
-1	14	14	10	39	0
1.666667	22	8	7	25	6
-2	19	14	13	21	0
-0.33333	24	12	7	79	2
-2				21	2
-2	15	5	12	25	0
	16	18	14	28	0
-1.66667	12	11	5	21	
-1.33333	11	19	16	62	8
-2	10	8	18	28	0
-2	17	8	9		1
	19	11	10	21	4
-2	9	21	14	57	1
-0.33333	10	9	11	49	16
-2	10	5	4	21	3
-2	11	8	13	28	0
-2	22	5	9	23	1
-2	14	4	4	21	2
1	22	5	4	21	4

Adult_Affec	Child_Conf	Child_Affective	PCS Rumin	PCS Magni	PCS Helplessness	PMD Major
1	1	0	4	4	5	0
0	5	2	4	4	8	0
8	19	8	11	6	17	1
1	0	0	0	0	0	0
10	13	11	12	4	6	1
6	18	9	3	3	4	0
0	4	2	3	0	5	0
0	16	9	16	10	17	
5	11	5	5	4	0	0
3	7	4	12	5	15	0
7	11	6	2	0	4	
8	13	1	8	4	19	1
7	11	5	6	3	11	1
7	17	10	16	7	21	0
2	0	0	16	7	24	0
4	3	1	5	1	6	0
3	15	11	2	1	4	0
2	0	0	2	5	7	0
7	4	2	2	2	0	0
5	2	1	6	3	3	
1	3	0	10	6	16	0
9	20	10	1	2	4	0
			12	6	14	1
			8	8	11	0
			6	2	5	0
			0	0	1	
			3	3	2	0
			0	0	0	
			4	0	3	
			0	0	4	0
			12	6	14	1
			4	0	6	
			2	1	5	
			10	3	6	0
			12	9	18	1
			0	1	1	0
			0	0	0	0
0	0	0	0	0	0	0
			0	3	3	1
0	19	9	0	0	0	0

			6	5	6	0
2	0	0	9	3	15	0
			5	0	6	
6	17	7	9	2	8	0
			8	7	7	0
			8	4	10	0
0	0	0	12	0	8	0
1	15	4	7	6	12	1
2	10	7	4	0	1	0
			0	1	1	0
2	0	3	3	0	6	
			3	1	12	0
			3	0	0	0
0	0	2	4	1	2	0
0	0	0	0	0	0	0
3	5	3	5	1	6	0
2	3	0	4	2	0	0
9	11	6	12	5	14	
8	14	6	6	4	2	0
			5	2	10	
			1	0	3	0
			6	5	2	
			0	1	2	
			0	1	3	
			0	0	0	
			6	2	1	
			3	0	0	0
			8	7	10	0
			0	0	0	0
4	8	3	11	1	12	
			1	4	2	0
			8	3	14	0
			11	7	15	0
6	10	6	12	7	17	1
			0	0	0	0
2	2	0	0	0	2	0

			0	0	1	0
2	15	11	12	3	12	0
5	7	4	13	4	18	1
2	0	0	0	0	0	0
			5	2	3	0
			4	3	6	0
			0	1	2	
			4	2	3	0
			2	4	6	0
5	9	3	9	8	12	1
8	18	9	13	6	11	0
			9	8	13	0
6	7	3	5	5	10	1
12	20	10	3	1	4	1
5	9	3	9	8	12	1
4	17	6	0	0	0	
12	20	12	4	1	9	0
12	20	10	4	2	2	0
			10	10	6	0
			8	2	5	1
			15	6	22	1
			5	4	6	0
			1	1	1	0
			15	5	16	0
			7	8	12	1
			14	8	13	0
			13	9	21	1
			5	7	7	1
			5	6	6	0
			16	12	24	1
0	0	0	7	2	6	1
			7	10	14	1
			0	0	0	0
			4	1	6	1
			9	6	8	0
2	13	7	6	5	11	1
0	6	7	6	4	10	1
2	0	0	0	3	7	0
2	3	2	13	6	19	1
5	1	0	12	9	5	1
0	3	2	1	0	1	0
0	20	12	3	2	10	0
5	13	3	10	8	3	0
			16	11	21	1

2	0	0	10	10	13	0
7	0	0	10	7	19	1
3	10	5	7	1	6	0
5	10	6	9	8	13	1
0	15	3	2	0	5	1
4	1	0	7	5	7	1
6	9	0	8	6	11	1
2	0	0	8	4	18	0
5	17	9	1	2	5	0
5	10	8	6	5	8	0
8	0	0	6	3	11	0
			0	0	0	0
			16	9	19	0
			16	7	13	1
			5	6	7	1
			6	5	13	1
			14	3	15	1
			6	6	11	1
			12	6	17	1
			2	1	6	0
			1	2	5	
			4	1	1	
			8	5	11	
			4	1	7	
			11	9	11	0
			2	2	3	
4	7	3	9	2	10	0
			10	4	10	
0	3	3	8	2	9	0
6	1	0	10	3	18	0
2	20	12	10	8	14	1
12	2	0	16	6	18	1
9	9	7	14	8	19	1
			0	0	0	1
			3	6	10	
0	0	0	6	5	4	0
1	5	1	4	0	2	0
7	11	10	5	6	8	0
			12	8	15	0
			6	5	10	1
0	3	6	11	6	17	1
0	3	6	11	6	17	1
3	13	6	10	7	14	1
			11	6	13	1

			8	10	17	
			7	1	8	0
6	10	6	8	5	11	1
0	20	12	12	7	19	0
5	5	2	8	2	5	0
5	3	2	11	8	12	1
8	12	8	10	8	9	0
			0	1	3	1
1	7	1	4	1	5	0
			11	7	16	0
			14	8	20	1
2	1	4	0	0	0	0
9	1	1	13	10	19	
6	5	4	13	11	20	1
8	16	11	4	2	3	0
10	20	11	16	12	22	1
7	15	7	4	4	9	1
2	7	2	8	8	14	1
			2	2	6	0
12	0	0	15	9	18	1
6	0	0	1	2	11	0
1	13	6	11	5	9	0
1	13	6	11	5	9	0
7	7	4	16	10	20	1
9	7	1	5	7	8	0
			12	4	10	0
6	5	4	13	11	20	1
0	0	0	13	7	21	1
5	12	9	2	2	4	0
8	16	11	4	2	3	0
0	12	0	16	8	24	1
9	0	0	16	12	22	1
7	7	4	16	10	20	1
7	20	12	16	12	24	1
5	1	0	12	9	5	1
0	0	0	8	7	14	
11	10	6	7	2	7	0
			4	2	6	0
8	0	0	14	9	17	1
5	13	3	10	8	3	0

3	0	0	9	3	12	0
2	5	2	1	1	1	0
1	5	1	0	1	0	0
0	3	3	8	2	9	0
0	3	0	8	6	8	0
9	1	1	13	10	19	
1	7	1	4	1	5	0
1	5	1	4	0	2	0
2	0	0	10	10	13	0
6	9	0	8	6	11	1
8	0	0	6	3	11	0
9	9	7	14	8	19	1
9	19	11				
2	0	0	8	4	18	0
0	20	12	3	2	10	0
6	14	2	3	1	6	0
0	7	2	5	3	7	0
12	20	10	3	1	4	1

0	2	0	12	5	11	0
3	0	0	0	0	1	0
0	7	5	10	10	17	
6	5	3	0	0	0	
5	10	6	0	0	0	0
			3	4	3	
			0	0	0	
			11	0	10	
			0	2	0	0
7	15	7	4	5	7	1
			0	0	0	0
0	0	0	10	4	10	
0	2	2	2	1	5	0
10	19	9	0	1	2	0

2	11	3	0	1	1	0
1	16	6				0
0	0	0	0	0	0	0
0	0	0	0	0	0	0
2	4	0	0	0	0	0
0	2	1	4	0	2	0
4	16	8	0	0	0	0
0	0	0	2	0	3	0
			0	0	0	0
			5	0	2	
			8	6	12	
			3	0	1	0
			0	0	0	
			0	0	0	
			2	1	2	
			0	1	0	
			0	0	0	0
			2	0	0	0
			0	0	0	0
			2	0	2	0
0	9	3	0	0	0	0
			3	0	0	
			1	0	1	0
1	0	0	0	0	0	0
1	1	0	0	0	0	0
0	1	1	0	0	2	0
			4	2	5	0
			0	0	0	
5	9	5	6	4	7	0
0	3	0	8	7	8	0
1	8	2	1	1	2	0
			0	0	0	
2	1	0	15	5	11	0
			1	0	1	0
			0	0	0	0
0	1	0	14	10	18	
10	20	12	7	4	6	0
			0	0	0	0
0	0	0	4	1	1	0
0	0	0	0	0	3	0
0	7	4	0	0	0	0
0	1	0	0	0	0	0
1	3	0	0	0	0	0
						0

0	1	0

PMD Other	PMD Panic	PMD Difficulty Score	TIQ	TIQ How of	TIQ How much	B5 Extraver
0	0	3				
0	0	3				
0	0	2				
0	0	2				
0	0	3				
0	0	1				
0	0	0	27	14	13	19
0	0	1				
1	0	2				
0	0	3	66	32	34	7
0	0	3				
0	0	1				
0	0	3				
0	0	2				
0	0	3				
0	0	0				
0	0	3	27	14	13	14
0	0	2				
0	0	1				
0	0	1	40	21	19	2
0	0	0	38	19	19	16
0	0	3	39	19	20	31
			20	9	11	
0	0	2	44	21	23	14
			20	10	10	
			43	20	23	
0	0	3	34	17	17	17
0	0	3	76	34	42	17
			63	27	36	
			56	25	31	
0	0	2	50	24	26	26
0	0	3	53	27	26	18
0	0	2	35	18	17	3
0	0	0	20	10	10	16
0	0	3	28	14	14	20
0	0	3	62	28	34	2
1	0	1	43	20	23	18

0	0	1	32	16	16	18
0	0	1				
			50	25	25	
0	0	3				
1	0	3	74	35	39	4
0	0	2	42	21	21	23
0	0	1	90	42	48	13
0	0	1				
0	0	0				
0	0	1	46	23	23	15
0	0	2	40	21	19	14
0	0	0	21	10	11	3
0	0	2				
0	0	0				
0	0	1				
1	0	2				
0	0	3				
			64	32	32	
0	0	1	36	18	18	9
			37	18	19	
			50	24	26	
			28	14	14	
			29	13	16	
			48	20	28	
0	0	2	45	23	22	21
0	0	3	46	20	26	25
0	0	2	42	21	21	6
0	0	1	37	16	21	13
1	0	2	73	37	36	5
1	0	2	72	32	40	
0	0	2	43	18	25	21
0	0	3	61	28	33	11
1	0	1	53	25	28	9

0	0	0		22	11	11		20
0	0	2		55	23	32		15
0	0	3		77	37	40		8
0	0	0		20	10	10		18
0	0	0		20	10	10		29
0	0	0		30	15	15		25
				49	22	27		
0	0	1		61	31	30		22
1	0	3		37	19	18		11
0	0	2		47	22	25		14
1	0	1		57	26	31		23
0	0	3		49	25	24		
0	0	1		46	23	23		10
0	0	1		49	25	24		3
0	0	2		48	22	26		14
1	0	0		39	20	19		3
1	0	2		42	21	21		3
0	0	2		48	24	24		9
0	0	3		40	19	21		
0	0	3		58	29	29		
0	0	3		82	44	38		13
0	0	2						
0	0	1		38	19	19		8
0	0	2		53	26	27		12
0	0	1		49	24	25		11
0	0	2		72	36	36		13
0	0	3		62	31	31		10
0	0	2		55	27	28		9
0	0	2		92	46	46		11
0	0	3		45	23	22		14
0	0	3		59	30	29		10
0	0	1		30	14	16		14
0	0	3		34	16	18		14
0	0	1		52	25	27		18
0	0	3		72	36	36		15
0	0	3		56	28	28		16
1	0	0		54	27	27		9
0	0	3		82	42	40		9
0	0	3		37	18	19		14
0	0	1		33	17	16		18
0	0	1		19	9	10		24
0	0	2		53	24	29		23
0	0	3		66	32	34		19

0	0	1	54	27	27	16
0	0	3	64	29	35	5
0	0	2	33	18	15	22
0	0	2	61	31	30	10
0	0	2	40	19	21	7
0	0	3	55	27	28	9
0	0	2	94	48	46	6
1	0	3	65	30	35	18
0	0	1				
0	0	0	47	21	26	12
0	0	3	63	31	32	29
0	0	2	49	24	25	4
0	0	2	87	42	45	5
0	0	3	102	51	51	6
0	0	3	67	35	32	16
0	0	3	93	45	48	13
0	0	3	54	29	25	9
0	0	3	61	31	30	10
0	0	2	65	32	33	8
1	0	3	44	19	25	14
			41	20	21	
			45	23	22	
			82	37	45	
			61	29	32	
0	0	3	55	27	28	21
			41	20	21	
1	0	1	49	23	26	22
			41	21	20	
1	0	3	71	36	35	25
1	0	3	79	33	46	17
0	0	2	35	15	20	12
0	0	2	69	33	36	17
0	0	2	73	37	36	12
0	0	1	39	18	21	11
			86	43	43	
1	0	3	65	34	31	24
0	0	2	28	14	14	17
1	0	1	41	20	21	9
0	0	2	60	30	30	11
0	0	2	46	24	22	5
0	0	3	69	32	37	16
0	0	3	68	32	36	16
0	0	1	83	38	45	11
0	0	3	88	42	46	

			55	28	27	
0	0	3	53	25	28	4
0	0	1				20
0	0	0	37	17	20	19
0	0	0	36	17	19	24
0	0	2	74	36	38	16
1	0	3	64	30	34	15
0	0	3	57	24	33	14
0	0	3	49	24	25	18
0	0	1	43	20	23	18
0	0	2	80	40	40	17
1	0	1	60	27	33	20
			92	46	46	1
0	0	3	56	28	28	13
0	0	2	46	23	23	21
0	0	3	112	55	57	9
0	0	2	60	28	32	18
0	0	2	50	25	25	11
0	0	1	40	20	20	7
0	0	3	97	47	50	23
1	0	3	70	33	37	7
1	0	2	73	36	37	13
1	0	2	42	21	21	13
0	0	2	91	45	46	6
1	0	1	51	26	25	12
0	0	0	51	25	26	16
0	0	3	55	28	27	13
0	0	3	92	46	46	12
0	0	0	41	21	20	23
0	0	2	47	23	24	21
0	0	3	56	29	27	13
0	0	3	78	39	39	1
0	0	2	90	45	45	6
0	0	3	111	55	56	8
0	0	3	42	21	21	14
0	0	1	51	26	25	18
0	0	2	56	25	31	12
0	0	3	79	38	41	8
0	0	2	56	24	32	23

0	0	2	54	26	28		12
0	0	1	44	23	21		26
0	0	1	18	9	9		20
1	0	3	74	36	38		25
0	0	0	44	23	21		18
			91	46	45		1
0	0	3	52	24	28		18
0	0	2	31	14	17		17
0	0	1	51	27	24		16
0	0	2	95	48	47		6
0	0	3	64	31	33		29
0	0	2	73	37	36		12
							17
1	0	3	66	30	36		18
0	0	1	22	9	13		24
0	0	1	74	36	38		2
0	0	0	44	22	22		
0	0	1	50	25	25		3
0	0	0					
0	0	0					
0	0	0					
			22	12	10		
			28	14	14		
			35	15	20		
0	0	0	31	14	17		14
0	0	1					
0	0	0	21	10	11		9
0	0	1					
0	0	1	57	27	30		17

0	0	0	37	18	19	12
0	0	1				
0	0	0	25	13	12	13
0	0	0	24	12	12	17
0	0	0				
0	0	0	50	27	23	11
0	0	0	38	19	19	11
0	0	0	31	15	16	25
0	0	0	42	16	26	9
			28	14	14	
			102	51	51	
0	0	1	43	18	25	14
			41	21	20	
			39	19	20	
			30	15	15	
			18	9	9	
0	0	0	26	13	13	7
0	0	0	18	9	9	18
0	0	0	40	19	21	17
0	0	0	33	14	19	18
0	0	0	41	20	21	20
0	0	1	20	10	10	16
0	0	0	15	8	7	23
0	0	0	40	18	22	32
0	0	0	22	11	11	17
0	0	0	26	10	16	17
			62	30	32	
0	0	1	26	13	13	14
0	0	0	15	10	5	22
0	0	0	41	20	21	16
			28	14	14	
0	0	0	53	25	28	22
0	0	0	22	11	11	30
0	0	0	27	13	14	14
1	0	1	62	30	32	2
1	0	2	39	20	19	21
0	0	1	32	16	16	6
0	0	0	34	16	18	27
0	0	0				
0	0	0	21	9	12	13
0	0	0				
0	0	0				

22	10	12

B5 Agreeab	B5 Conscie	B5 Neurotic	B5 Openness	McGill throl	McGill shoo	McGill stab	McGill shar
				2	0	0	2
				2	2	2	2
				2	3	3	3
				0	0	0	0
				1	0	0	0
				1	0	0	1
30	28	9	32	2	3	3	3
				2	1	3	3
				0	0	0	0
				2	3	2	3
				0	1	1	2
26	28	28	35	0	0	0	2
				3	2	2	2
				0	3	0	0
				1	0	1	1
				0	0	0	0
				2	2	0	2
				3	2	2	2
26	31	18	35	2	0	0	1
				2	0	0	0
				1	2	2	1
				2	0	0	2
29	30	20	38	2	0	0	0
18	18	16	20	2	3	3	3
33	34	3	35	3	1	1	3
				1	0	1	1
28	25	21	31				
				0	0	0	0
				0	0	0	0
29	28	8	35	0	3	3	3
22	28	26	39	3	1	2	3
				0	0	1	1
				1	3	0	0
33	26	22	16	1	1	1	1
29	27	16	26	3	2	2	2
35	33	14	38	0	3	0	2
28	33	5	39	1	0	0	0
26	30	7	25	0	0	0	0
14	0	25	21	0	0	0	0
30	25	14	35	1	2	2	3

24	27	11	28	2	2	0	0
				0	0	0	0
				2	1	2	2
				2	2	2	2
21	21	22	28	0	1	1	1
27	23	6	29	3	2	3	3
20	23	28	26	3	2	0	0
				0	0	0	2
				2	0	0	0
20	18	16	26	3	3	3	0
				3	2	0	3
				0	1	1	2
25	27	9	30	2	1	1	1
24	17	8	7	2	0	0	0
				1	0	0	0
				0	0	0	0
				2	2	2	0
				0	0	1	0
				1	1	1	3
				0	0	0	0
				1	3	3	3
27	26	11	24	1	1	1	1
				1	0	0	0
				0	0	0	0
				1	1	1	1
				0	0	0	0
25	19	19	28	0	0	0	0
33	26	5	36	1	0	0	1
30	13	9	28	0	0	0	0
				1	2	0	3
29	29	5	35	0	0	0	0
19	25	20	12	0	0	0	1
				1	3	3	3
35	25	9	32	3	3	2	3
28	30	11	39	2	3	3	3
30	19	26	26	0	1	1	1

32	27	4	37	0	0	0	0
24	25	25	16	0	1	0	0
26	23	17	30	0	2	2	1
23	25	9	23	0	0	0	0
34	32	0	33	1	2	0	0
34	27	8	30	2	0	0	0
				0	0	0	1
13	12	21	25	2	2	2	2
21	29	19	14	0	0	0	0
26	20	13	28	1	3	1	2
18	26	14	36	2	2	2	1
				2	3	3	1
24	16	13	31	1	2	2	3
30	25	16	22	0	0	2	1
26	20	13	28	1	3	1	2
28	24	21	22	2	0	1	1
27	26	18	18	3	1	1	3
27	24	16	37	1	1	1	1
				1	1	0	2
				3	2	2	2
16	25	20	23	3	2	2	2
				1	3	3	3
24	28	22	18	2	2	1	2
29	25	17	36	1	1	1	2
22	22	18	18	2	2	3	3
22	19	24	30	3	1	1	3
9	4	26	18	1	2	2	3
20	25	24	16	2	1	1	2
22	21	28	13	2	3	3	3
29	15	23	19	1	1	2	2
14	17	21	29	2	3	3	3
26	27	8	24	2	2	2	2
30	29	12	15	3	3	1	3
23	32	11	31				
23	21	18	23	3	3	3	3
33	20	24	22	3	3	2	3
20	22	18	18	2	0	0	0
9	12	28	14	3	2	0	2
31	32	14	30	0	1	1	2
28	23	10	29	2	1	0	1
36	35	0	38	3	3	3	3
35	18	16	38	2	3	3	2
27	31	20	19	3	2	2	2

28	16	22	25	1	0	2	2
19	8	30	13	2	2	3	3
32	33	8	39	3	0	0	2
25	21	18	20	2	2	1	2
18	25	7	31	2	1	1	2
25	9	19	22	1	2	2	1
22	19	25	21	2	2	2	2
25	31	15	14	2	0	0	1
				2	0	0	2
27	30	16	26	0	0	0	2
26	30	16	39	2	2	2	2
28	17	26	29	0	0	0	0
13	16	27	21	2	3	3	3
15	19	23	26	3	3	1	1
24	17	22	23	2	1	1	2
24	20	28	27	2	3	2	1
27	27	10	25	2	2	3	3
21	18	15	12	1	2	2	2
22	24	15	23	2	3	2	2
23	20	7	33	1	1	1	1
				0	1	0	0
				2	1	1	1
				2	3	3	3
				3	2	1	2
25	28	13	34	2	2	2	2
				2	2	2	2
35	34	10	37	0	2	2	2
				1	2	0	1
21	28	24	40	2	3	3	3
24	29	16	12	3	1	1	1
28	29	17	26	1	2	2	3
27	24	24	29	3	3	3	3
28	34	22	38	1	2	2	2
33	27	11	23	1	1	2	2
				2	2	3	2
19	25	23	36	1	2	2	3
27	28	14	18	0	0	0	3
24	10	18	36	2	3	3	3
23	23	17	29	1	3	3	3
27	17	25	22	2	2	2	2
32	23	25	29	3	1	2	1
32	23	25	29	3	1	2	1
24	14	26	24	2	1	2	2
				2	3	2	3

				1	2	1	2
25	13	18	12	1	3	3	2
24	21	20	18	2	2	2	2
34	36	2	39	3	3	3	3
31	23	9	28	2	1	3	1
30	24	27	22	2	3	3	3
21	12	15	13	2	2	3	3
27	24	18	17	1	3	3	3
24	24	16	40	3	3	2	3
32	29	7	27	2	2	2	1
25	22	13	20	2	3	3	3
21	24	13	28	0	0	0	0
17	13	32	11	2	2	2	2
32	18	21	10	3	3	1	3
28	20	12	36	2	1	1	1
				3	0	1	1
23	8	32	21	2	3	3	3
25	19	14	29	2	1	0	1
27	18	13	28	1	3	2	3
17	36	5	10	2	2	2	3
19	22	28	18	1	1	1	1
22	23	17	27	2	3	3	3
24	24	17	26	2	2	1	0
24	24	17	26	2	2	1	0
24	20	16	20	3	2	3	3
28	23	16	32	1	3	3	3
29	28	11	32	0	0	0	0
32	18	21	10	3	3	1	3
14	18	23	19	3	3	3	3
32	26	16	35	0	0	0	1
28	20	12	36	2	1	1	1
27	32	16	27	3	3	3	3
12	8	30	5	2	2	1	2
24	20	16	20	3	2	3	3
19	22	21	25	3	3	3	3
31	32	14	30	0	1	1	2
				3	3	3	3
33	33	10	31	2	1	0	2
30	21	17	34	2	1	1	1
14	13	28	3	2	1	2	2
35	18	16	38	2	3	3	2

24	20	13	16	0	0	0	4
32	24	12	35	2	3	3	2
24	32	10	14	0	1	0	1
21	28	24	40	2	3	3	3
20	23	11	12	3	1	1	2
17	13	32	11	2	2	2	2
24	24	16	40	3	3	2	3
27	28	14	18	0	0	0	3
28	16	22	25	1	0	2	2
22	19	25	21	2	2	2	2
26	30	16	39	2	2	2	2
28	34	22	38	1	2	2	2
24	16	18	19	3	2	2	2
25	31	15	14	2	0	0	1
36	35	0	38	3	3	3	3
				2	3	2	3
26	19	26	15	2	1	2	2
				1	1	1	1
30	25	16	22	0	0	2	1

				3	0	0	0
				0	0	1	1
				2	1	1	1
				0	0	0	0
				0	0	0	0
				0	2	0	2
				0	0	0	0
				3	0	0	0
18	31	5	37	0	0	2	2
				1	3	3	2
30	29	7	28	0	0	0	0
				2	1	1	1
				2	0	2	2
22	23	22	24	1	0	0	0

31	23	8	27	1	1	0	0
				0	0	0	0
23	24	16	30	0	0	0	0
27	32	1	26	0	0	0	0
				0	0	0	0
21	29	26	20	1	2	2	2
17	29	12	23	0	0	0	0
23	23	10	34	0	0	0	0
31	24	5	23	0	0	0	0
				2	1	1	2
				0	0	0	0
29	27	16	31	0	0	0	0
				0	0	0	0
				0	0	0	0
				3	1	0	0
				0	0	0	0
35	30	11	32	0	0	0	0
34	34	1	24	0	0	0	0
23	26	16	21	0	0	0	0
21	21	16	29	0	0	0	0
28	30	8	34	0	0	0	0
				1	1	1	1
26	13	3	22	0	0	0	0
26	32	3	35	0	0	0	0
27	32	19	22	0	0	0	0
27	34	8	28	0	0	0	0
29	26	11	29	0	0	0	0
				0	0	0	1
24	23	13	20	1	2	3	2
29	21	13	27	0	0	0	0
28	29	4	29	0	0	0	0
				0	0	0	0
22	22	12	32	0	0	0	0
27	35	5	27	1	0	0	0
32	28	11	24	0	1	0	0
				2	2	2	2
28	18	16	17	2	1	1	1
27	31	20	25	0	1	0	1
32	23	11	19	2	0	0	2
35	30	4	21	0	0	0	0
				0	0	0	0
28	28	14	38	0	0	0	0
				0	0	0	0

0	0	0	2

McGill cran	McGill gnav	McGill hot_	McGill achi	McGill heav	McGill tend	McGill split	McGill tiring	McGill sick
0	0	2	3	2	1	1	3	1
1	3	2	3	3	3	1	3	0
1	1	2	3	1	3	2	3	2
0	0	2	2	0	0	0	0	0
3	1	2	2	2	3	2	3	2
1	2	1	2	0	2	1	3	0
2	0	3	3	3	3	0	2	0
0	0	1	3	2	1	1	2	1
0	0	0	2	2	2	0	3	0
3	3	2	3	2	2	3	3	3
0	0	0	2	0	0	0	0	0
0	0	0	3	2	2	0	2	0
1	2	3	3	2	3	0	3	1
0	3	3	3	1	2	0	2	0
0	2	0	2	2	2	3	3	3
2	2	3	2	2	0	0	2	0
2	0	0	2	2	2	2	0	0
0	3	3	3	2	2	1	3	1
0	1	0	3	1	3	2	3	0
2	1	1	3	3	3	0	2	2
0	0	2	0	0	0	0	0	0
0	0	2	0	0	2	0	2	0
2	0	0	3	2	2	0	3	0
2	2	2	2	2	3	1	1	1
3	2	0	3	3	2	1	3	3
2	2	0	2	2	2	0	2	0
0	0	0	2	0	0	0	0	0
0	0	0	1	0	1	0	1	0
3	0	0	2	2	2	0	2	0
1	3	3	3	3	3	2	3	0
0	0	0	3	3	3	0	3	0
3	0	0	2	0	2	0	2	0
0	0	0	3	3	0	0	2	0
2	3	3	3	3	3	2	3	3
0	2	3	3	1	2	1	1	1
1	0	0	1	0	0	0	1	0
0	0	0	2	0	1	0	0	0
0	0	0	2	0	0	0	0	0
2	0	0	3	1	2	0	3	0

2	0	0	2	0	2	0	2	1
2	3	0	3	2	0	2	3	3
1	2	0	1	0	1	1	2	0
3	0	3	3	3	0	3	3	3
2	1	3	3	3	2	1	3	0
0	0	2	3	3	3	1	3	1
0	0	1	3	0	3	0	3	0
1	0	0	2	2	3	0	3	2
0	0	2	2	1	1	2	2	0
1	0	0	1	0	1	0	0	0
2	1	2	3	0	3	0	2	1
1	0	0	3	3	3	0	3	0
0	0	1	3	1	0	1	3	2
0	0	2	2	2	0	0	3	0
0	0	0	2	2	0	0	2	0
0	0	0	0	0	0	0	0	0
2	2	0	2	0	2	2	2	2
2	0	0	2	0	0	0	0	0
2	2	2	2	2	2	0	0	1
0	0	2	1	0	0	0	3	0
1	1	3	3	3	3	3	3	3
0	1	1	2	1	2	0	1	0
0	0	0	2	0	0	0	2	0
0	0	0	0	0	0	0	0	0
1	2	1	2	1	2	0	2	1
0	0	0	0	0	0	0	0	0
0	0	0	0	0	0	0	2	0
2	1	1	3	3	2	0	3	0
0	0	0	0	1	0	0	2	0
0	0	0	1	3	0	2	3	2
0	0	0	0	0	0	0	0	0
2	0	0	3	2	1	0	0	0
1	0	0	2	2	2	1	2	0
1	1	1	3	1	1	1	1	0
2	1	2	2	0	2	0	0	0
1	0	1	2	0	2	0	0	0

2	0	1	0	1	0	0	1	0
3	0	0	3	0	0	0	3	0
0	0	0	1	0	0	0	2	0
0	0	0	0	0	0	0	0	0
2	0	0	1	0	0	2	1	0
0	0	0	2	0	0	0	2	0
0	1	0	2	0	0	0	1	0
1	0	3	2	0	1	1	2	1
2	0	0	3	0	3	0	1	0
0	3	0	3	1	0	0	3	3
3	2	2	3	0	0	0	3	0
0	3	3	3	0	1	0	1	0
0	2	2	3	0	1	0	0	0
0	0	2	2	0	1	2	1	0
0	3	0	3	1	0	0	3	3

1	0	0	2	0	0	0	1	0
0	0	1	1	0	1	3	2	0
1	2	2	3	3	2	1	3	2
3	1	1	3	1	3	1	2	1
1	2	3	3	2	2	1	3	3
1	1	0	2	0	1	3	3	2
3	1	2	1	0	1	1	3	1
3	1	1	3	1	1	1	0	1
3	3	0	3	3	0	1	3	1
3	2	2	3	2	3	3	3	1
2	3	3	3	3	3	3	3	3
1	1	1	2	2	1	3	3	2
2	2	1	3	1	1	2	1	2
2	3	3	3	3	2	3	3	3
1	1	3	2	0	2	2	3	2
2	1	2	2	2	2	1	2	1
2	2	2	3	2	2	2	3	0
1	0	0	3	1	1	3	3	3

3	2	3	3	2	3	0	3	2
2	3	2	3	2	3	2	3	1
0	0	0	2	0	0	0	2	2
1	0	0	3	2	3	1	3	1
2	0	0	3	1	1	0	3	0
1	1	1	2	1	2	1	2	1
0	0	3	3	1	2	3	3	0
3	3	1	3	2	2	3	3	2
2	2	2	3	2	2	2	3	2

0	0	2	2	0	1	2	3	1
1	1	3	2	0	0	0	2	1
3	0	2	3	2	2	0	3	2
0	0	1	2	2	2	1	3	2
3	2	0	2	3	1	0	3	0
0	0	0	3	3	3	3	3	1
1	1	0	3	0	2	2	2	2
3	3	2	3	3	1	1	3	2
1	0	0	3	2	3	0	3	1
2	0	0	2	0	0	0	0	0
2	2	1	1	2	2	1	2	1
2	2	0	2	0	0	0	0	0
3	2	2	3	3	3	2	2	2
2	1	2	2	1	1	1	2	1
2	0	0	3	1	2	1	3	1
3	2	2	3	1	2	1	3	3
3	1	3	3	3	3	3	3	3
1	1	0	2	1	0	1	2	1
1	2	0	3	0	2	0	3	2
2	3	2	3	2	1	0	3	1
2	0	3	0	0	0	0	3	0
1	3	2	3	3	2	1	3	1
3	2	0	3	3	3	3	3	2
1	1	1	3	1	1	1	3	1
2	1	1	2	1	2	1	3	2
2	0	1	3	0	3	0	3	0
3	2	2	3	0	0	0	3	0
0	0	0	3	3	3	0	3	2
1	3	1	3	2	1	2	3	3
3	3	3	3	3	0	3	3	3
2	1	0	3	0	2	0	3	0
3	0	0	0	0	2	0	3	3
2	1	0	2	2	2	1	3	0
1	1	2	2	1	2	1	3	1
2	1	3	2	1	2	2	2	1
2	1	2	1	1	2	0	3	1
2	2	3	3	2	2	3	3	0
1	2	1	2	2	3	1	3	2
2	2	1	3	3	2	3	3	2
3	1	2	2	2	2	2	2	1
1	1	1	3	3	3	2	3	0
1	1	1	3	3	3	2	3	0
1	0	1	3	3	3	1	3	0
1	1	3	2	1	3	1	3	1

3	0	1	3	2	3	0	3	2
2	3	2	3	1	0	2	3	0
1	1	1	2	2	2	1	2	2
0	1	3	3	3	3	3	3	3
1	1	1	3	2	3	1	3	2
2	1	1	2	2	1	2	2	1
2	3	1	2	3	2	2	3	3
2	2	1	2	0	1	0	3	1
3	1	1	2	1	2	3	3	3
2	2	2	2	2	2	2	2	2
2	1	2	3	2	1	3	3	3
0	0	0	0	0	0	0	0	0
2	1	2	3	3	2	0	3	2
1	1	3	3	1	2	1	3	2
3	3	1	1	2	2	1	3	2
0	0	3	3	3	0	0	3	0
3	2	3	3	1	3	3	3	3
1	0	1	2	3	3	1	3	3
2	2	2	2	2	1	0	2	2
0	0	0	3	0	2	0	3	2
2	0	2	3	0	2	0	3	2
0	0	0	2	3	2	3	3	3
0	0	0	1	0	1	0	2	0
0	0	0	1	0	1	0	2	0
2	3	3	3	2	2	2	3	3
1	2	1	2	0	0	0	2	3
0	2	0	2	2	0	2	2	0
1	1	3	3	1	2	1	3	2
2	2	3	3	3	3	3	3	3
3	3	2	2	2	3	1	3	2
3	3	1	1	2	2	1	3	2
3	2	0	3	3	3	3	3	3
3	2	3	2	2	2	3	3	2
2	3	3	3	2	2	2	3	3
3	3	3	3	0	0	0	3	3
2	0	0	3	1	1	0	3	0
3	3	3	3	3	3	3	3	3
2	0	2	2	0	2	2	2	1
3	3	2	3	2	3	0	3	3
3	1	2	2	2	2	2	3	2
3	3	1	3	2	2	3	3	2

3	0	0	4	3	0	0	4	0
3	3	1	3	2	2	3	3	2
0	0	0	2	1	2	0	2	0
1	3	1	3	2	1	2	3	3
0	0	2	2	0	0	0	2	0
2	1	2	3	3	2	0	3	2
3	1	1	2	1	2	3	3	3
2	2	3	3	2	2	3	3	0
0	0	2	2	0	1	2	3	1
1	1	0	3	0	2	2	2	2
2	2	1	1	2	2	1	2	1
2	1	0	2	2	2	1	3	0
3	1	3	3	0	3	2	2	2
3	3	2	3	3	1	1	3	2
0	0	3	3	1	2	3	3	0
2.5	1.5	2	2	1.5	1.5	2.5	3	2.5
2	0	0	1	0	2	0	1	1
2	2	1	3	1	2	2	3	3
0	0	2	2	0	1	2	1	0

2	2	0	3	3	3	1	3	1
0	0	0	0	0	0	0	0	1
2	0	0	1	2	3	1	3	1
0	0	0	0	0	0	0	0	0
0	0	0	0	0	0	0	0	0
1	0	0	2	0	2	0	0	0
0	0	0	0	0	0	0	0	0
0	0	1	3	3	3	0	3	0
0	0	1	1	0	0	0	0	0
1	1	3	0	2	1	0	0	1
0	0	0	0	0	0	0	0	0
1	2	0	3	2	2	0	2	0
3	0	0	2	0	2	2	2	0
1	0	0	1	0	0	0	0	0

1	0	0	1	0	0	0	0	0
0	0	0	0	0	0	0	1	0
0	0	0	1	0	0	0	0	0
0	0	0	0	0	0	0	0	0
0	0	0	0	0	0	0	0	0
1	0	0	1	0	0	0	0	0
1	0	0	1	0	0	0	1	0
0	0	0	0	0	0	0	0	0
0	0	0	0	0	0	0	0	0
0	0	0	2	0	2	0	1	0
0	0	0	0	0	0	0	0	0
0	0	0	0	0	0	0	0	0
0	0	0	0	0	0	0	0	0
0	0	0	0	0	0	0	0	0
2	0	0	1	0	1	0	0	0
0	0	0	1	0	0	0	0	0
0	0	0	0	0	0	0	0	0
0	0	0	0	0	0	0	0	0
0	0	0	0	0	0	0	0	0
0	0	0	1	0	1	0	0	0
0	0	0	1	0	1	0	0	0
0	0	0	0	1	0	0	1	1
0	0	0	0	0	0	0	0	0
0	0	0	0	0	0	0	0	0
0	0	0	0	0	0	0	0	0
0	0	0	0	0	0	0	0	0
0	0	0	0	0	0	0	0	0
0	0	0	1	0	0	0	0	0
0	0	0	1	0	0	1	1	0
0	0	0	0	0	0	0	0	0
0	0	0	0	0	0	0	0	0
0	0	0	0	0	0	0	0	0
0	0	0	0	0	0	0	0	0
0	0	0	1	0	0	0	0	0
0	0	0	1	0	0	0	0	0
3	2	0	3	3	1	2	1	1
0	0	2	2	0	0	2	2	0
2	0	0	1	0	0	1	1	0
1	0	0	2	0	0	1	0	0
0	0	0	1	0	0	0	0	0
0	0	0	1	0	0	0	0	0
0	0	0	0	0	0	0	0	0
0	0	0	0	0	0	0	0	0

0	0	0	0	0	1	0	0	0

McGill fear	McGill puni	McGill Tota	McGill Sens	McGill Affective=last4=	CMSI fatigu	CMSI morn	CMSI musc
0	0	17	13	4	4	3	3
0	0	27	24	3	4	4	4
2	2	33	24	9	4	3	4
0	0	4	4	0	4	2	3
0	0	21	16	5	4	4	3
0	1	15	11	4			
0	0	27	25	2	4	4	3
0	0	20	17	3	3	3	4
3	0	12	6	6	4	1	3
2	3	39	28	11	4	4	4
0	0	6	6	0	3	0	3
0	0	11	9	2	4	3	3
1	1	29	23	6	4	2	3
2	2	21	15	6	3	4	3
3	3	26	14	12	4	4	2
0	0	13	11	2	4	3	4
0	0	16	16	0	4	4	3
0	0	27	23	4	3	4	3
0	0	16	13	3			
0	0	19	15	4	3	3	2
0	0	8	8	0	4	0	3
2	0	12	8	4	4	4	3
0	0	14	11	3	4	3	3
1	1	29	25	4	2	2	3
0	2	30	22	8	4	4	4
0	0	15	13	2	2	3	3
					4	3	4
0	0	2	2	0	4	4	3
0	0	3	2	1	3	2	1
0	0	20	18	2	0	0	0
0	0	30	27	3	4	2	3
0	1	15	11	4	4	2	1
0	0	13	11	2	3	4	3
0	0	12	10	2	4	1	1
2	2	38	28	10	4	4	4
0	0	19	17	2	4	4	3
0	0	4	3	1	3	0	1
0	0	3	3	0	4	0	1
0	0	2	2	0	4	1	1
0	0	19	16	3	4	3	3

0	0	13	10	3	3	2	3
0	3	21	12	9	4	4	4
0	1	16	13	3	4	2	3
0	0	29	23	6	4	4	4
0	0	21	18	3	4	4	3
					4	4	4
0	0	27	23	4	4	4	4
0	0	15	12	3	3	3	4
0	0	15	10	5	4	3	3
0	0	12	10	2	3	2	2
0	0	12	12	0	4	3	3
0	0	22	19	3	4	3	3
0	0	17	14	3			
0	3	19	11	8	3	3	2
0	0	11	8	3	3	3	3
0	0	7	5	2	4	0	0
0	0	0	0	0	4	0	0
2	0	22	16	6	3	2	3
0	0	5	5	0	4	3	2
1	0	20	18	2	4	2	2
0	0	6	3	3	4	0	4
2	3	38	27	11	4	2	4
0	0	12	11	1	3	3	2
0	0	5	3	2	3	3	3
0	0	0	0	0	3	1	1
0	0	16	13	3	3	2	2
0	0	0	0	0	4	2	3
					3	1	2
0	0	2	0	2	4	3	0
0	0	17	14	3	4	4	4
0	0	3	1	2	4	1	1
0	2	19	12	7	4	2	0
0	0	0	0	0	4	0	0
0	0	9	9	0	2	3	4
0	2	22	18	4			
0	2	23	20	3	3	3	4
0	0	20	20	0	1	4	4
1	0	10	9	1	3	2	3

0	0	5	4	1	3	2	2
0	0	10	7	3	4	4	4
0	2	10	6	4	3	2	1
0	0	0	0	0	2	0	0
0	0	9	8	1	3	2	2
1	0	7	4	3	3	3	3
0	0	5	4	1	3	1	1
0	0	19	16	3	2	3	3
0	0	9	8	1	4	3	2
1	0	21	14	7	3	3	3
0	0	20	17	3	4	3	4
0	0	20	19	1	2	3	3
0	0	16	16	0			
0	0	11	10	1	3	1	3
1	0	21	14	7	3	3	3
0	0	8	7	1	2	0	1
0	0	16	14	2	3	0	0
0	2	25	18	7	3	4	4
0	0	20	17	3	4	3	3
3	3	35	23	12	4	4	4
1	1	24	17	7	4	3	3
0	0	23	19	4	4	3	3
0	0	19	18	1	4	4	4
1	3	26	18	8	4	4	4
1	1	34	28	6	3	3	3
0	3	37	28	9	4	3	4
0	0	24	19	5	4	4	4
1	1	23	18	5	3	3	3
3	3	42	30	12	4	4	4
1	2	25	17	8	4	3	3
2	2	30	23	7	3	4	3
0	0	26	23	3	3	4	3
0	0	25	19	6	4	4	3
					2	3	3
2	3	38	28	10	4	4	4
1	1	34	28	6	4	4	4
2	0	10	4	6	3	3	3
2	1	24	17	7	4	4	4
3	0	17	11	6	3	4	4
1	0	17	13	4	3	3	3
0	3	30	24	6	4	3	4
2	1	35	27	8	0	3	4
3	2	34	24	10	3	3	4

2	2	20	12	8	4	2	1
0	0	20	17	3	4	3	3
0	0	22	17	5	3	3	3
1	0	21	15	6	3	4	3
0	0	20	17	3	4	4	4
0	0	22	18	4	4	2	4
1	0	22	17	5	3	3	3
2	3	29	19	10	4	4	4
0	0	17	13	4	3	2	3
0	0	6	6	0	3	0	0
0	0	22	19	3	4	3	3
0	0	6	6	0	3	2	2
2	3	38	29	9	3	4	4
1	1	23	18	5	4	4	4
1	2	22	15	7	4	3	2
3	2	33	22	11	3	4	4
0	0	35	29	6	4	4	4
2	1	19	13	6	4	4	3
0	1	23	17	6	4	4	3
0	2	23	17	6	4	4	4
0	0	9	6	3	4	2	4
0	3	27	20	7	4	4	4
1	3	37	28	9	4	4	4
1	2	24	17	7	4	1	3
2	2	27	18	9	4	4	3
0	0	20	17	3	4	2	4
0	0	19	16	3	3	2	3
1	1	20	13	7	4	3	4
0	1	31	24	7	4	3	1
3	3	36	24	12	4	4	4
0	0	19	16	3	3	3	3
0	3	26	17	9	4	3	3
0	1	21	17	4	4	4	4
0	0	20	16	4	4	3	3
1	1	27	22	5	4	3	3
2	1	24	17	7	4	4	3
0	0	23	20	3	4	3	3
1	2	31	23	8	3	3	3
0	2	33	26	7	4	3	4
1	1	27	22	5	3	3	3
0	3	27	21	6	4	4	4
0	3	27	21	6	4	4	4
0	2	24	19	5	3	4	4
1	1	28	22	6	4	3	3

2	1	26	18	8	4	4	4
0	0	25	22	3	4	4	3
2	2	26	18	8	3	4	4
2	3	39	28	11	4	3.5	4
0	0	24	19	5	4	3	3
1	1	27	22	5	3	3	4
3	3	37	25	12	4	4	4
0	0	22	18	4	4	4	4
2	0	32	24	8	4	2	2
1	2	28	21	7	3	4	3
3	3	37	25	12	4	4	4
0	0	0	0	0	3	0	1
2	3	31	21	10	4	4	4
1	1	29	22	7	4	4	4
1	0	24	18	6	4	4	4
0	0	17	14	3			
3	3	41	29	12	4	1	4
1	1	23	15	8	4	3	3
1	0	25	20	5	4	3	4
0	0	19	14	5	4	4	4
3	3	24	13	11	4	4	4
0	0	27	21	6	4	3	4
1	0	10	7	3	3	2	3
1	0	10	7	3	3	2	3
2	2	38	28	10	3	3	3
1	1	23	16	7	4	3	3
0	0	10	8	2	4	4	3
1	1	29	22	7	4	4	4
2	3	42	31	11	4	4	4
0	0	22	17	5	3	4	3
1	0	24	18	6	4	4	4
3	3	41	29	12	4	4	4
2	2	33	24	9	4	4	4
2	2	38	28	10	3	3	3
3	3	36	24	12	4	4	4
3	0	17	11	6	3	4	4
3	3	45	33	12	4	4	4
0	0	18	15	3	3	3	3
1	1	29	21	8	4	3	3
2	2	30	21	9	4	4	4
2	1	35	27	8	0	3	4

0	0	18	14	4	3	1	3
2	1	35	27	8	0	3	4
0	0	9	7	2	3	3	3
0	1	31	24	7	4	3	1
0	0	13	11	2	3	3	3
2	3	31	21	10	4	4	4
2	0	32	24	8	4	2	2
0	0	23	20	3	4	3	3
2	2	20	12	8	4	2	1
1	0	22	17	5	3	3	3
0	0	22	19	3	4	3	3
0	1	21	17	4	4	4	4
1	1	30	24	6			
2	3	29	19	10	4	4	4
0	3	30	24	6	4	3	4
1.5	1	31.5	23.5	8	4	2	3
0	0	14	12	2	2	0	2
1	2	26	17	9	4	2	3
0	0	11	10	1	3	1	3

1	1	23	17	6	2	3	3
0	0	3	2	1	0	0	0
1	0	19	14	5	4	3	3
0	0	0	0	0	4	1	0
0	0	0	0	0	0	0	0
0	0	9	9	0	3	2	2
0	0	0	0	0	3	2	1
0	0	16	13	3	4	4	4
0	0	6	6	0	3	1	2
0	0	18	17	1	2	3	2
0	0	0	0	0	3	0	0
0	0	17	15	2	3	4	3
0	0	17	15	2	0	0	3
0	0	3	3	0	0	1	0

0	0	4	4	0	0	0	0
0	0	1	0	1	0	0	0
0	0	1	1	0	0	0	0
0	0	0	0	0	0	0	0
0	0	0	0	0	0	0	0
0	0	9	9	0	1	1	1
0	0	3	2	1	0	1	1
0	0	0	0	0	0	0	0
0	0	0	0	0	0	0	0
0	0	11	10	1	2	0	1
0	0	0	0	0	4	4	0
0	0	0	0	0	1	0	0
0	0	0	0	0	0	0	0
0	0	0	0	0	0	0	0
0	0	8	8	0	1	0	1
0	0	1	1	0	1	2	1
0	0	0	0	0	1	1	1
0	0	0	0	0	0	1	0
0	0	0	0	0	0	0	1
1	0	3	2	1	2	0	0
0	0	2	2	0	0	1	1
0	0	7	5	2	1	1	1
0	0	0	0	0	0	0	0
0	0	0	0	0	1	1	0
0	0	0	0	0	0	0	0
0	0	0	0	0	1	1	0
0	0	0	0	0	1	0	0
0	0	2	2	0			
0	0	11	10	1	2	3	2
0	0	0	0	0	0	0	0
0	0	0	0	0			
0	0	0	0	0	0	1	0
0	0	0	0	0	0	0	0
0	0	2	2	0	0	1	0
0	0	2	2	0	0	2	0
1	1	26	22	4	2	3	4
0	0	13	11	2	3	0	0
0	0	7	6	1	1	2	1
0	0	8	8	0	3	1	2
0	0	1	1	0	1	0	1
0	0	1	1	0	0	0	0
0	0	0	0	0	0	0	0
0	0	0	0	0	0	0	0

0	0	3	3	0		1	1	1

CMSI musc	CMSI recur	CMSI dry_e	CMSI dry_	CMSI finge	CMSI finge	CMSI swoll	CMSI swoll	CMSI SOB_
2	0	4	0	3	2	2	0	3
4	3	2	0	0	4	3	3	4
3	0	4	0	2	3	3	3	0
0	0	3	0	0	3	3	0	3
3	0	2	3	0	0	0	0	4
3	1	0	0	0	2	1	3	3
2	0	0	0	1	2	0	0	0
2	0	0	3	0	4	0	2	2
3	0	2	3	0	3	1	0	2
0	0	0	2	0	3	0	0	2
0	0	3	1	0	0	0	3	0
3	3	1	1	1	2	2	0	2
2	0	1	1	3	3	0	0	1
1	4	2	3	0	2	1	1	1
0	0	2	0	0	0	0	0	3
2	1	0	0	4	4	3	3	4
3	3	0	0	0	0	0	0	0
0	1	2	3	0	3	0	2	0
2	0	0	4	0	0	2	0	3
3	0	2	2	2	4	1	4	3
2	0	0	0	1	0	0	0	2
3	2	1	1	0	1	0	1	1
4	1	3	3	1	3	2	0	1
0	0	2	2	0	0	0	0	0
2	0	1	1	0	2	0	0	0
0	0	1	0	0	1	1	0	1
0	0	2	2	0	0	0	1	1
0	0	0	0	0	0	0	0	0
2	0	1	4	0	0	2	1	1
0	0	0	0	1	3	0	0	1
4	0	4	3	1	1	0	0	3
2	2	3	2	0	2	2	0	1
4	2	3	3	3	3	3	2	3
0	0	2	2	0	0	0	0	1
0	0	0	0	0	0	2	0	0
1	3	0	0	0	0	3	0	3
1	0	0	0	0	0	0	0	0
3	0	0	0	2	2	2	3	3

3	0	0	0	0	1	0	3	2
3	2	2	2	0	3	3	0	1
1	1	0	0	0	0	0	0	0
0	4	4	0	0	4	3	4	3
1	0	4	3	0	2	0	0	2
1	2	4	4	4	4	2	1	4
4	0	0	0	0	0	1	0	3
3	0	0	0	0	0	0	3	3
2	0	2	2	0	2	0	3	0
1	3	4	2	0	2	2	3	1
1	3	0	1	0	1	1	3	2
4	2	4	3	2	0	3	3	3
0	0	1	1	0	0	0	0	1
0	1	0	0	0	1	0	1	2
0	0	0	0	0	0	0	0	0
0	0	0	0	3	2	0	0	0
2	0	0	0	0	0	0	0	0
0	0	0	0	0	0	0	0	0
0	0	0	0	2	2	1	0	1
1	0	3	2	2	3	0	0	2
2	0	2	1	0	3	0	0	1
1	2	3	2	1	1	2	2	2
1	0	3	1	0	0	1	3	1
2	0	0	0	0	0	0	0	1
1	0	0	0	0	1	0	0	0
0	0	2	0	0	0	0	0	3
2	0	0	2	0	1	0	0	2
0	0	0	0	0	0	2	1	1
2	1	1	3	1	1	0	0	2
1	0	0	1	0	0	2	3	1
0	0	0	0	4	4	0	0	0
0	0	0	2	0	0	2	0	0
2	0	0	1	0	2	1	2	0
3	1	0	4	0	2	4	2	0
4	0	3	1	0	1	0	1	3
4	1	3	1	2	2	1	1	0

1	0	3	0	0	0	1	1	0
3	0	3	0	0	0	0	0	3
0	2	2	2	0	0	0	0	0
0	0	0	2	0	0	3	0	0
3	0	0	1	0	2	0	0	0
3	0	0	0	0	0	0	0	0
1	0	1	1	0	0	0	0	3
2	0	2	1	0	0	0	0	2
0	1	1	0	0	0	0	3	2
1	0	0	1	1	2	0	2	1
2	2	2	1	2	2	2	2	2
1	0	0	0	0	0	1	1	0
1	0	3	1	0	0	0	0	0
1	0	0	1	1	2	0	2	1
0	0	4	4	0	2	0	0	0
2	0	4	4	0	3	0	0	0
3	2	3	4	3	3	2	2	4
2	0	2	3	0	0	2	1	3
2	0	0	0	0	2	0	2	1
3	0	1	0	0	0	2	2	3
2	0	2	2	0	2	0	2	1
3	0	4	3	1	4	0	1	1
2	0	0	0	1	1	2	2	2
2	0	1	2	0	2	2	2	1
3	3	3	3	2	4	4	4	4
2	1	3	1	0	0	1	1	1
2	2	3	2	2	3	2	3	2
3	2	2	2	0	0	2	3	3
3	3	2	3	3	3	3	3	3
2	3	1	1	1	4	3	1	1
3	0	4	1	1	1	1	2	3
1	0	0	0	0	0	0	0	4
3	0	2	2	1	2	0	1	2
4	2	2	3	3	3	3	4	3
4	1	3	3	2	3	3	4	3
3	0	1	1	0	0	0	0	3
2	0	0	1	0	0	0	2	2
2	1	0	0	0	0	2	2	3
3	2	1	1	1	1	2	3	2
2	1	2	2	1	1	1	1	2
3	2	3	2	2	2	0	1	0
4	1	2	1	0	0	2	2	2

2	3	1	3	3	3	2	2	2
3	0	0	0	0	0	0	0	0
2	2	0	0	0	0	2	0	3
2	1	0	0	1	1	0	3	2
2	0	2	3	0	0	0	0	0
2	1	2	0	1	2	2	0	1
2	0	2	2	0	0	1	0	0
2	0	1	4	1	4	1	2	0
0	0	3	2	0	0	0	0	2
0	0	3	0	0	0	0	0	0
3	4	0	0	0	0	0	0	1
0	0	1	1	0	3	2	0	1
2	0	0	3	0	3	0	3	3
4	2	3	3	3	3	2	3	3
2	1	2	3	0	1	0	2	2
3	0	4	4	2	2	3	3	4
4	2	1	4	4	4	3	4	2
2	1	0	2	3	3	0	0	2
3	2	0	2	2	3	0	3	1
3	2	3	3	3	3	3	2	2
2	0	2	2	0	1	4	4	1
4	2	3	4	0	0	2	4	2
0	4	4	4	4	2	0	2	2
2	0	4	4	1	1	0	1	4
3	2	2	2	4	4	4	3	3
3	0	0	0	0	0	1	0	0
3	0	2	2	1	3	0	0	1
1	1	0	0	0	1	1	3	0
2	0	0	3	0	0	0	4	1
3	0	1	3	1	3	0	1	1
1	0	0	0	0	1	2	1	1
3	2	4	4	1	3	1	1	3
3	0	3	4	0	3	0	0	3
1	1	3	2	0	1	3	1	3
3	1	3	3	1	1	0	2	2
2	3	0	0	0	2	0	0	3
0	1	0	1	3	3	0	2	0
3	2	2	2	1	1	1	2	1
3	3	1	1	0	3	2	2	4
4	0	3	4	4	4	1	0	0
3	0	0	0	0	1	0	2	0
3	0	0	0	0	1	0	2	0
2	0	0	0	0	1	1	1	0
2	2	1	3	0	0	0	2	4

3	2	3	3	1	3	1	4	3
1	1	0	0	0	1	1	1	1
4	2	2	1	0	1	1	3	2
2	1.5	2	2.5	1.5	1	1	2	1.5
2	1	1	1	0	1	2	0	1
3	1	1	2	1	3	0	2	2
3	1	1	1	0	1	2	2	4

1	0	3	1	0	0	1	0	3
3	1	1	1	0	0	1	1	1
3	1	1	1	0	3	0	2	2
4	0	1	3	3	3	0	1	3
0	0	0	1	0	0	0	0	2
3	3	3	3	3	3	1	4	4
3	4	0	0	0	2	4	4	4
4	3	3	3	2	2	3	2	1

4	3	0	4	0	0	0	4	3
3	3	2	2	2	2	2	4	2
2	2	1	0	0	3	0	1	2
0	0	0	0	0	1	0	3	0
2	0	0	0	2	3	4	4	3
2	2	4	0	0	1	3	0	3
1	0	4	3	2	2	2	1	1
1	0	4	3	2	2	2	1	1
3	1	4	4	2	4	1	1	4
2	2	1	0	0	3	1	3	1
0	0	3	3	0	0	0	0	0

3	4	0	0	0	2	4	4	4
4	3	2	2	1	2	3	3	3
4	3	3	3	3	3	3	4	2
4	3	3	3	2	2	3	2	1
2	0	0	1	2	3	2	0	2
4	3	3	3	3	3	2	4	4

3	1	4	4	2	4	1	1	4
4	4	4	4	4	4	4	4	4
2	1	0	0	0	0	2	2	3
4	3	4	4	4	4	3	4	3
3	0	0	0	0	3	3	0	4
1	1	1	3	0	3	2	1	2
4	3	3	3	3	3	3	4	3
3	2	3	2	2	2	0	1	0

2	1	0	1	0	2	0	0	4
3	2	3	2	2	2	0	1	0
0	0	1	0	3	3	0	0	0
2	0	0	3	0	0	0	4	1
2	1	0	3	0	0	0	0	3
3	3	3	3	3	3	1	4	4
3	1	1	1	0	0	1	1	1
0	1	0	1	3	3	0	2	0
2	3	1	3	3	3	2	2	2
2	0	2	2	0	0	1	0	0
3	4	0	0	0	0	0	0	1
3	0	3	4	0	3	0	0	3
2	0	1	4	1	4	1	2	0
2	1	2	2	1	1	1	1	2
3	1	1	1	0	1	2	1	1
0	0	0	0	0	0	0	1	0
3	2	1	1	0	1	2	2	2
1	0	3	1	0	0	0	0	0

0	0	3	0	0	2	2	0	0
0	0	0	0	0	0	0	0	0
4	2	0	0	0	0	3	3	0
0	0	0	0	0	0	2	0	2
0	0	0	0	0	0	0	0	0
2	0	4	1	0	0	1	0	1
0	0	2	0	0	0	0	0	1
0	0	3	3	0	3	2	0	0
2	0	0	0	0	0	0	0	0
2	1	3	3	3	2	2	1	1
0	0	4	4	0	3	0	0	3
3	1	1	1	1	1	1	1	1
3	0	0	0	0	0	0	0	0
0	0	0	2	0	0	0	0	2

0	0	0	0	0	0	0	0	0
0	0	3	0	0	0	0	0	2
0	0	0	0	0	0	0	0	0
0	0	0	0	0	0	0	0	0
0	0	0	0	0	0	0	0	0
0	0	1	1	0	0	0	1	1
0	0	0	0	0	0	0	1	0
0	0	0	0	0	0	0	0	0
0	0	0	0	0	0	0	0	0
3	0	0	2	3	3	1	0	1
3	0	0	0	0	0	0	0	0
0	0	0	1	0	0	2	0	0
0	0	1	0	0	0	0	0	0
0	0	0	0	0	0	0	0	0
1	0	0	2	1	1	0	0	0
0	0	0	0	0	0	0	0	0
0	0	0	0	0	0	0	0	0
0	0	0	0	0	0	0	0	0
0	0	0	0	0	0	0	0	0
1	0	0	1	0	0	0	0	1
0	0	0	0	0	0	0	0	0
0	0	1	1	0	0	0	2	0
0	0	0	0	0	0	0	0	0
0	0	0	0	0	0	0	0	0
0	0	0	0	0	0	0	0	0
0	0	0	0	0	0	0	0	0
0	0	0	0	0	0	0	0	0
1	0	0	0	0	0	0	0	0
0	0	0	0	0	0	0	0	0
0	0	2	0	0	1	0	0	0
0	0	0	0	0	0	0	0	0
0	0	0	0	0	0	0	1	0
0	0	0	0	0	0	0	0	0
4	0	2	0	4	3	0	0	0
0	0	4	3	0	2	0	0	0
0	0	0	0	0	0	0	0	0
0	0	1	1	0	0	1	0	1
0	0	0	0	0	0	0	0	0
0	0	0	0	0	0	0	0	0
0	0	0	0	0	0	0	0	0
0	0	0	0	0	0	0	0	0

0	0	0	0	0	0	0	0	0

CMSI SOB_	CMSI SOB_	CMSI SOB_	CMSI SOB_	CMSI rapid_	CMSI chest	CMSI irregu	CMSI palpit	CMSI migra
3	2	1	1	0	2	0	0	4
4	4	4	4	2	2	2	2	0
0	0	0	0	0	2	2	2	0
4	3	2	2	0	0	0	23	0
4	4	4	4	4	4	0	0	0
4	3	3	2	1	2	0	0	0
0	0	0	0	0	0	0	0	2
1	2	0	0	4	0	1	0	0
3	3	0	2	4	3	2	2	3
0	0	0	0	0	0	0	0	0
0	0	0	0	0	0	0	0	0
3	2	1	0	1	0	1	2	3
3	2	1	0	0	0	1	1	1
2	2	0	0	0	0	0	0	4
0	0	0	0	0	0	0	0	0
4	4	3	3	1	0	1	1	2
3	3	2	0	0	0	0	0	0
0	0	0	0	0	0	0	0	0
2	0	0	3	0	0	0	2	0
3	2	2	0	3	0	1	3	0
3	2	1	0	0	2	0	0	3
1	1	1	1	1	1	0	0	3
1	2	0	1	3	0	0	0	0
1	0	0	0	2	0	0	0	2
0	0	0	0	1	0	0	0	0
3	1	0	0	0	0	0	0	3
3	3	2	1	3	0	0	2	3
0	0	0	0	0	0	0	0	0
1	1	1	1	0	1	0	1	2
2	0	0	0	1	0	0	0	0
3	2	2	1	1	2	2	2	1
2	0	0	0	3	0	0	4	0
3	2	2	2	2	3	2	3	4
1	1	0	0	0	0	0	1	0
2	2	2	0	0	0	0	0	0
3	3	1	1	1	1	0	1	3
0	0	0	0	2	0	0	0	0
3	3	2	2	2	0	0	3	2

2	2	2	0	1	1	1	1	4
1	1	1	1	1	0	1	1	4
2	1	1	0	2	1	2	2	4
3	3	2	0	3	3	0	0	4
3	3	3	3	0	0	0	0	0
4	4	2	4	3	3	3	3	4
3	3	3	3	2	0	0	0	0
3	2	2	0	0	0	0	0	0

2	0	0	0	0	0	0	0	3
1	0	0	0	1	0	0	1	3
3	3	2	2	0	0	1	1	4
3	0	0	2	2	2	1	2	3

1	0	0	1	0	0	0	0	2
2	2	2	3	0	0	0	0	1
0	0	0	0	0	0	0	0	0
0	0	0	0	0	0	0	0	0
0	0	0	0	0	0	0	1	0
0	0	0	0	0	0	0	1	0
1	1	1	1	1	1	0	0	0
2	3	3	2	3	0	0	3	2
1	1	1	1	2	0	0	2	0
1	1	0	2	3	2	2	2	1
2	2	2	1	2	0	1	0	0
2	2	1	1	1	0	0	0	0
0	0	0	0	0	0	0	0	2
3	3	2	1	0	0	0	0	0
3	0	0	3	2	0	0	1	0
1	0	0	0	1	0	0	1	0
4	3	2	2	1	1	0	0	0

1	1	0	0	3	1	0	0	0
2	0	0	0	0	0	0	0	4
0	0	0	0	0	0	0	1	0

1	0	0	0	1	0	0	0	1
1	0	0	0	0	3	0	3	2
3	2	2	2	3	0	0	0	0
1	0	0	0	0	0	0	0	0

1	0	0	0	0	0	0	0	0
3	3	3	2	1	0	1	1	0
0	0	0	0	1	0	0	0	1
1	0	0	0	0	0	0	0	0
0	0	0	0	0	0	0	0	3
0	0	0	0	1	0	0	1	0
3	3	2	3	2	1	1	1	0
2	2	1	1	2	2	0	2	0
3	2	2	2	1	0	0	1	0
1	1	1	1	1	1	0	0	1
2	2	0	0	3	2	2	2	3
1	1	0	0	0	0	0	1	0
2	0	0	0	0	0	0	0	3
1	1	1	1	1	1	0	0	1
2	0	0	0	0	0	0	0	3
1	1	0	0	1	0	0	0	3
4	3	3	2	3	0	0	1	4
3	3	2	2	1	1	0	1	0
1	0	0	0	0	0	0	0	1
3	3	2	2	3	0	2	2	2
3	2	2	1	3	0	4	4	1
1	1	1	1	0	0	0	0	1
2	2	2	1	0	1	0	1	2
1	0	0	0	0	1	0	0	0
4	3	3	3	3	0	0	1	3
2	2	1	0	2	2	0	2	3
2	1	1	1	2	2	1	2	3
3	4	3	2	3	3	0	1	4
3	2	3	1	2	2	0	0	1
3	1	2	1	1	1	0	0	2
3	3	3	1	1	1	0	0	0
4	3	2	3	1	3	0	0	4
3	2	2	1	0	0	0	0	0
3	3	3	4	3	4	2	3	4
4	4	3	2	3	3	3	3	2
2	1	0	0	1	0	0	0	0
3	3	2	1	0	0	0	0	2
3	3	2	1	1	2	1	2	0
2	2	2	2	2	2	2	2	0
3	2	2	2	3	2	1	2	4
2	0	0	2	2	3	1	1	4
3	1	1	1	2	3	1	3	4

2	1	1	0	0	2	0	2	0
0	0	2	0	2	2	2	3	0
3	3	3	3	2	2	0	2	3
2	2	2	0	0	0	2	2	2
1	0	0	0	0	0	0	0	0
1	1	1	1	1	0	0	0	4
0	0	0	0	0	0	0	0	0
1	0	0	0	2	0	0	1	1
2	0	0	0	0	0	0	0	0
0	0	0	0	0	0	0	0	0
1	1	1	1	0	0	0	0	2
1	1	0	0	2	0	0	2	3
2	2	2	2	3	0	0	1	3
4	4	4	3	4	2	3	4	4
2	3	1	0	1	1	0	1	1
4	4	3	3	4	3	2	3	4
3	3	2	1	1	0	0	0	4
2	1	1	1	2	0	0	0	1
1	0	0	0	0	0	0	0	0
2	1	0	0	3	0	4	4	3
2	2	0	0	3	0	0	0	0
3	3	2	1	0	1	0	0	4
2	1	3	3	1	2	2	4	4
4	4	4	0	2	2	1	2	1
3	2	2	1	4	3	4	4	0
1	1	1	0	0	3	0	0	3
3	3	2	1	0	0	1	1	2
3	3	2	2	1	0	0	0	1
3	3	2	0	1	1	0	2	3
2	1	1	1	1	1	1	1	1
1	1	1	1	0	0	0	1	0
3	1	1	1	1	1	1	1	4
3	3	3	3	4	2	0	3	3
3	2	3	2	1	2	1	2	3
2	2	1	1	0	0	0	1	4
3	3	3	3	1	0	0	0	2
0	0	0	0	0	0	0	0	4
2	3	1	1	2	2	1	1	2
4	4	4	4	3	0	2	3	4
3	2	0	1	0	0	0	0	3
1	3	1	0	0	0	0	2	1
1	3	1	0	0	0	0	2	1
0	1	0	0	0	0	0	0	0
4	4	4	4	1	0	1	0	2

3	3	3	1	3	1	0	0	4
2	2	2	0	1	1	1	2	3
2	1	1	1	2	2	1	2	3
2	1.5	1.5	1.5	2.5	2	1.5	2	4
1	1	0	0	0	1	0	0	4
1	1	1	0	2	1	1	2	4
4	4	4	4	2	2	0	0	3
3	3	3	3	3	1	2	2	2
1	1	0	1	1	2	1	1	0
2	2	2	1	1	2	1	1	3
4	3	2	2	3	3	1	3	0
1	0	0	0	0	0	0	0	0
4	4	4	4	3	2	0	0	4
4	4	4	3	0	0	0	0	4
3	1	1	2	2	0	0	0	3
2	2	2	0	2	2	0	2	4
3	2	2	2	3	2	0	2	2
1	1	1	0	1	1	0	0	1
0	0	0	0	0	0	0	0	4
2	2	2	2	0	2	0	0	0
3	3	3	0	3	3	3	3	3
3	2	1	1	0	0	0	0	0
3	2	1	1	0	0	0	0	0
4	4	4	2	2	1	1	1	3
1	1	1	1	1	2	0	1	0
3	2	0	0	0	0	0	0	3
4	4	4	3	0	0	0	0	4
3	3	3	3	3	0	0	2	4
3	3	3	2	2	2	2	1	3
3	1	1	2	2	0	0	0	3
4	1	1	1	2	2	1	0	2
4	4	4	4	2	3	1	3	4
4	4	4	2	2	1	1	1	3
4	4	4	4	4	4	4	4	4
3	3	2	1	1	2	1	2	0
3	3	3	3	3	0	1	1	4
4	4	3	3	3	3	0	3	3
3	2	1	1	1	0	0	1	3
4	3	3	3	2	2	1	1	4
2	0	0	2	2	3	1	1	4

3	2	2	0	0	0	0	0	3
2	0	0	2	2	3	1	1	4
1	0	0	0	0	0	0	0	2
3	3	2	0	1	1	0	2	3
3	3	0	1	1	0	0	0	4
4	4	4	4	3	2	0	0	4
1	1	0	1	1	2	1	1	0
0	0	0	0	0	0	0	0	4
2	1	1	0	0	2	0	2	0
0	0	0	0	0	0	0	0	0
1	1	1	1	0	0	0	0	2
3	3	3	3	4	2	0	3	3
1	0	0	0	2	0	0	1	1
3	2	2	2	3	2	1	2	4
1	1	1	1	1	2	1	1	0
0	0	0	0	0	0	0	0	0
2	1	1	1	1	1	0	1	0
2	0	0	0	0	0	0	0	3
2	0	0	0	0	0	0	0	3
0	0	0	0	0	0	0	0	0
0	0	0	0	0	0	0	0	4
2	2	2	2	1	0	0	1	0
0	0	0	0	0	0	0	0	0
1	0	0	0	1	0	0	0	0
1	1	1	0	0	0	0	0	0
0	0	0	0	0	0	0	0	0
0	0	0	0	0	2	0	0	0
2	1	1	1	2	1	1	1	1
0	0	0	0	0	0	0	0	0
1	1	1	1	1	3	1	1	1
0	0	0	0	0	0	0	0	0
2	0	0	0	0	0	0	0	0

0	0	0	0	0	0	0	0	0
0	0	0	0	0	0	0	0	0
0	0	0	0	0	0	0	0	0
0	0	0	0	0	0	0	0	0
0	0	0	0	0	0	0	0	0
0	0	0	0	0	0	0	0	0
0	0	0	0	0	0	0	0	0
0	0	0	0	0	0	0	0	0
0	0	0	0	0	0	0	0	0
2	1	0	0	2	1	1	2	3
1	0	0	0	0	0	0	0	0
1	0	0	0	0	0	0	0	2
0	0	0	0	0	0	0	1	0
0	0	0	0	0	0	0	0	0
1	0	0	0	0	0	0	0	0
0	0	0	0	0	0	0	0	0
1	0	0	0	0	0	0	0	0
1	1	0	0	0	0	0	0	0
0	0	0	0	0	0	0	0	0
1	1	1	1	0	1	0	0	0
1	1	0	0	0	0	0	1	0
0	0	0	0	0	0	0	0	0
1	1	0	0	0	0	0	1	0
1	0	0	0	0	0	0	0	0
0	0	0	0	0	0	0	0	0
1	0	0	0	0	0	0	0	0
1	0	0	0	0	0	0	1	1
0	0	0	0	0	0	0	0	3
0	0	0	0	0	0	0	0	0
1	0	0	0	0	0	0	0	0
0	0	0	0	0	0	0	0	0
1	0	0	0	0	0	0	0	0
1	1	1	0	0	0	0	0	0
4	3	2	0	1	0	1	1	0
2	2	0	0	2	0	0	0	2
0	0	0	0	0	0	0	0	3
2	2	0	0	1	0	0	0	0
0	0	0	0	0	0	0	0	0
0	0	0	0	0	0	0	0	0
0	0	0	0	0	0	0	0	0
0	0	0	0	0	0	0	0	0

0	0	0	0	0	0	0	0	0

CMSI tensi	CMSI numb	CMSI mem	CMSI dizzir	CMSI balan	CMSI ear_p	CMSI ringin	CMSI heari	CMSI ear_b
0	3	3	2	0	0	2	0	0
0	4	3	4	4	0	0	0	0
0	4	3	3	3	4	4	2	0
0	4	3	3	0	0	0	0	0
3	0	4	3	3	3	2	0	1
0	2	3	4	3	0	1	0	3
1	0	1	1	0	4	2	0	4
2	4	4	3	1	0	0	0	0
2	3	3	1	2	0	3	2	0
0	0	0	0	0	0	0	0	0
0	3	3	0	1	0	0	0	0
3	2	0	1	2	1	0	0	1
2	1	4	1	2	0	0	1	1
3	1	3	1	1	0	1	1	0
0	0	3	2	2	0	0	0	0
2	0	2	2	1	1	0	0	0
3	3	3	2	3	4	2	3	3
0	3	2	0	0	0	0	0	0
0	0	4	2	2	2	3	2	0
0	4	4	1	0	0	1	0	0
2	0	2	3	2	2	2	0	2
3	2	2	0	0	0	1	0	0
4	3	4	2	0	2	0	0	0
2	1	1	0	0	0	0	0	0
1	1	3	1	2	0	0	0	1
0	0	3	3	3	0	0	1	0
0	0	3	3	1	0	2	0	0
0	0	0	0	0	0	0	0	0
2	1	2	2	2	2	3	3	0
0	3	3	1	1	0	0	0	0
0	1	4	1	0	0	0	0	1
3	0	3	1	2	1	0	0	1
3	3	4	3	3	3	4	0	2
2	2	3	2	2	0	0	0	0
0	0	1	0	0	0	2	0	0
3	0	3	1	1	1	1	0	0
0	0	1	0	0	0	0	0	0
1	1	4	2	1	0	4	3	0

1	3	2	2	2	2	2	0	1
0	3	4	1	3	4	1	0	4
0	1	1	2	1	0	1	0	0
3	3	4	4	3	0	4	0	0
2	1	2	2	1	0	0	0	1
0	4	4	4	3	1	1	1	1
0	2	2	1	2	0	4	0	3
0	0	0	0	0	0	0	0	0
3	2	3	3	3	3	2	0	3
2	2	1	1	1	1	1	0	1
3	0	4	4	4	0	0	0	0
2	3	3	3	3	2	1	0	1
0	1	1	1	0	0	0	0	0
2	0	1	0	0	0	1	0	0
0	0	4	2	0	0	3	0	0
4	0	3	2	0	0	0	0	0
1	3	1	1	0	0	0	0	0
0	2	0	3	0	0	0	0	0
0	0	0	2	1	0	0	0	0
0	0	0	1	0	3	3	0	0
0	0	3	2	2	0	0	0	1
1	2	2	2	1	2	1	0	1
0	2	4	2	2	1	0	0	0
0	1	1	1	1	0	1	2	1
2	1	1	1	0	3	2	0	0
0	0	3	0	2	0	4	0	0
3	0	2	3	0	0	0	0	0
0	1	3	2	0	0	1	0	0
0	2	1	0	2	0	2	1	0
0	3	3	0	1	3	0	0	1
1	2	1	1	1	1	0	0	0
3	0	1	1	1	0	1	0	0
1	1	2	0	0	0	4	4	1
1	3	4	2	1	0	0	0	0
0	3	3	0	0	0	4	0	3
2	0	3	2	3	0	4	3	2

1	3	1	1	1	2	0	2	0
0	1	2	1	1	1	1	1	1
2	2	2	1	1	1	3	1	0
2	0	1	0	0	0	0	0	1
0	4	1	0	0	0	0	0	0
0	1	1	3	3	0	1	3	0
0	0	2	0	0	2	2	1	2
0	3	3	1	2	0	0	1	0
1	2	2	2	0	3	2	0	2
1	1	2	1	1	0	2	1	2
2	2	2	3	2	2	2	2	2
1	1	2	2	0	2	2	0	4
3	3	0	1	2	0	3	3	0
1	1	2	1	1	0	2	1	2
3	4	1	1	1	0	3	4	0
3	3	2	1	1	0	3	2	0
4	3	4	3	3	1	4	2	0
3	1	3	2	2	2	2	1	3
2	4	3	2	1	0	0	0	0
2	1	3	2	2	0	4	0	0
0	0	3	1	2	0	3	2	2
2	2	3	1	0	0	4	1	1
0	1	4	1	1	1	4	3	0
3	3	2	1	1	1	4	3	1
4	3	4	2	1	3	4	3	4
2	3	3	2	2	3	4	4	2
3	2	2	2	1	1	2	1	1
4	3	4	2	2	1	3	3	1
3	3	3	3	3	0	3	1	1
2	3	3	1	1	0	1	0	1
0	2	2	1	1	1	1	0	2
4	3	2	3	3	0	2	0	0
1	2	1	1	1	0	2	0	0
3	4	4	2	4	2	3	0	2
2	3	3	3	3	2	2	2	3
2	2	3	2	1	0	3	0	0
2	1	4	2	1	0	0	0	0
1	1	4	1	1	1	2	2	3
2	2	3	3	3	0	2	0	0
2	4	3	2	2	2	4	2	2
4	3	2	4	4	3	4	2	3
4	3	3	2	2	2	4	2	2

4	2	3	3	3	1	3	3	1
0	4	4	0	0	0	3	3	0
2	0	1	1	1	0	0	0	0
2	2	2	1	2	3	2	0	1
0	3	4	2	1	0	3	3	0
4	2	3	2	2	0	3	3	0
3	3	3	1	0	0	2	2	1
1	4	3	1	1	0	4	2	0
0	0	0	0	0	0	0	0	0
0	0	3	0	0	2	0	0	0
2	2	2	1	1	0	4	1	0
2	0	3	2	1	0	0	0	0
3	3	3	2	3	3	3	1	2
4	4	4	3	3	1	3	3	3
2	2	3	2	1	0	1	1	0
4	4	4	4	4	2	3	2	3
4	4	4	4	4	4	4	4	4
2	0	4	3	2	1	4	1	1
0	3	4	3	3	0	3	3	0
2	4	4	4	3	2	4	4	2
0	4	4	4	4	4	4	4	4
2	3	4	2	3	4	1	0	4
2	4	2	2	3	3	0	3	0
4	2	4	4	3	1	2	0	1
0	3	3	3	3	3	3	3	3
3	3	3	2	2	0	1	0	1
2	3	3	2	2	0	4	3	2
0	1	3	2	1	0	1	0	0
1	2	3	2	3	0	1	4	1
3	4	3	2	1	0	4	2	1
0	1	3	1	2	0	2	1	1
3	1	2	1	1	1	0	0	0
4	3	3	4	3	0	3	1	0
3	2	2	1	1	2	1	0	2
1	3	3	3	3	0	4	3	0
3	3	4	3	3	1	4	1	2
4	0	4	1	2	3	0	0	0
3	3	2	3	3	2	3	1	2
2	2	4	3	4	2	2	1	3
2	2	4	4	4	0	0	0	0
1	2	3	2	3	1	0	0	1
1	2	3	2	3	1	0	0	1
2	1	2	1	2	0	0	0	1
2	3	4	4	3	0	0	1	2

3	3	3	1	1	1	1	0	3
3	2	4	3	2	0	4	3	0
2	2	2	2	2	1	1	1	1
3	4	3.5	2	2	1.5	4	2	1.5
2	2	3	1	1	2	3	0	2
3	3	2	1	0	0	2	3	3
3	0	4	0	0	1	4	0	1

0	4	3	1	2	1	1	0	1
1	2	2	1	1	0	1	0	1
3	3	3	2	2	2	4	2	2
1	4	4	3	3	4	1	1	0
0	0	2	1	1	0	0	0	1
4	1	4	3	3	3	4	4	1
4	2	4	3	2	4	4	4	3
1	3	3	4	4	1	2	0	2

4	3	4	2	2	2	1	1	3
2	3	3	1	2	2	2	1	2
2	1	4	2	2	1	2	1	1
4	0	3	0	1	0	2	2	1
3	3	2	1	1	3	0	0	1
0	2	3	3	3	2	2	2	2
2	1	3	1	1	0	2	1	2
2	1	3	1	1	0	2	1	2
3	3	4	4	4	3	3	3	3
1	2	2	1	0	1	2	1	2
3	0	2	2	1	0	0	0	0

4	2	4	3	2	4	4	4	3
4	0	4	1	3	2	2	2	2
3	3	3	4	4	2	3	2	2
1	3	3	4	4	1	2	0	2
2	3	4	1	0	2	2	0	0
4	3	4	3	3	3	4	3	3

3	3	4	4	4	3	3	3	3
4	4	4	4	4	4	4	4	4
1	1	4	1	1	1	2	2	3
4	3	3	3	3	3	3	3	3
3	2	2	1	0	0	0	0	0
3	3	2	1	0	2	0	1	1
3	2	4	3	3	3	4	3	3
4	3	2	4	4	3	4	2	3

0	0	3	0	0	0	3	0	0
4	3	2	4	4	3	4	2	3
1	0	3	0	1	0	0	0	0
1	2	3	2	3	0	1	4	1
0	0	4	0	0	0	3	1	0
4	1	4	3	3	3	4	4	1
1	2	2	1	1	0	1	0	1
4	0	4	1	2	3	0	0	0
4	2	3	3	3	1	3	3	1
3	3	3	1	0	0	2	2	1
2	2	2	1	1	0	4	1	0
4	3	3	4	3	0	3	1	0
1	4	3	1	1	0	4	2	0
2	4	3	2	2	2	4	2	2
2	1	1	2	2	0	1	0	1
2	1	2	0	0	0	1	1	0
0	1	2	1	1	1	1	0	0
3	3	0	1	2	0	3	3	0
3	0	2	0	0	0	0	0	0
0	0	0	0	0	0	0	0	0
0	4	3	3	4	0	0	0	0
0	3	2	3	3	2	2	0	3
0	0	0	0	0	0	0	0	0
2	0	3	2	1	2	1	0	3
0	0	2	0	0	0	3	0	0
0	0	2	1	0	0	0	0	0
1	1	2	2	1	0	3	2	0
3	2	3	2	1	2	4	1	1
3	0	3	0	0	0	0	0	0
3	1	1	1	1	1	1	1	1
0	0	0	0	0	0	0	0	0
0	0	0	0	0	0	0	2	0

0	0	0	0	0	0	0	0	0
0	0	0	0	0	0	0	0	0
0	0	0	0	0	0	0	0	0
0	0	0	0	0	0	0	0	0
0	0	0	0	0	0	0	0	0
1	1	1	0	0	0	2	2	0
0	0	0	0	0	0	3	2	1
0	0	0	0	0	0	0	0	0
0	0	0	0	0	0	0	0	0
3	1	2	2	1	0	2	0	2
2	0	2	3	3	0	0	0	0
2	0	0	0	1	0	0	0	0
0	0	0	0	0	0	0	0	0
0	0	0	0	0	0	0	0	0
0	1	0	0	0	1	0	0	2
0	0	0	0	0	0	0	0	0
0	0	1	0	0	0	0	0	0
0	0	0	0	0	0	0	0	0
0	0	0	0	0	0	0	0	0
0	0	1	0	1	0	0	0	0
1	0	1	0	0	0	1	0	0
1	0	0	1	0	0	0	0	0
0	0	0	0	1	1	0	0	0
0	0	1	0	0	0	0	0	0
0	0	0	0	0	0	0	0	0
0	0	1	0	0	0	0	0	0
1	0	0	0	0	0	0	0	0
3	1	1	1	0	0	1	1	0
0	0	0	0	0	0	0	0	0
0	0	0	0	0	0	0	0	0
0	0	0	0	0	0	1	0	0
0	0	1	0	0	0	0	0	0
0	0	0	0	0	0	0	0	0
0	4	0	0	4	0	0	0	0
2	2	3	0	3	3	3	3	0
3	0	1	0	0	0	0	0	0
3	0	1	1	0	1	1	0	1
0	0	1	0	0	0	3	0	0
0	0	0	0	0	0	0	0	0
0	0	0	0	0	0	0	0	0
0	0	0	0	0	0	0	0	0

0	1	0	0	0	0	0	0	1

CMSI sinus	CMSI abpai	CMSI abpai	09-change_	CMSI chang	CMSI chang	CMSI bloati	CMSI mucu	CMSI nause
0	0	0	0	0	0	0	0	3
3	0	0	0	0	0	3	2	3
0	0	0	2	2	0	0	2	1
0	0	0	0	0	0	0	0	0
0	2	3	3	2	2	1	0	2
0	0	0	2	2	2	2	0	2
0	0	0	0	0	0	0	0	0
0	0	0	4	4	0	0	0	0
0	2	2	3	3	2	3	2	2
0	0	0	0	0	0	0	0	0
3	1	4	4	0	3	2	0	3
0	0	0	0	0	0	0	0	0
2	2	3	3	3	3	3	1	0
4	0	0	0	0	0	0	0	0
0	2	2	0	0	2	0	0	0
2	0	0	0	0	0	0	0	0
2	4	4	4	4	4	3	4	2
1	0	0	2	2	2	3	0	2
3	3	3	3	3	0	3	0	0
2	4	3	3	3	3	4	0	0
2	1	0	0	0	0	3	0	1
2	1	0	1	1	1	1	0	0
0	4	4	0	4	0	4	0	1
0	0	0	0	0	0	1	0	0
0	2	2	0	0	0	2	1	0
0	0	0	0	0	0	0	0	0
1	0	0	0	1	1	0	0	1
0	0	0	0	0	0	0	0	0
0	1	1	4	4	1	1	0	0
0	1	0	1	1	0	1	2	0
1	1	1	2	3	3	4	1	0
0	0	0	0	2	0	1	0	2
3	3	3	2	2	3	3	1	2
0	3	3	1	1	1	3	0	3
0	0	0	0	0	0	0	0	0
0	2	1	1	1	0	0	1	2
0	2	2	1	1	1	1	0	1
0	0	0	2	2	0	1	0	0

2	2	2	2	2	2	2	0	2
3	2	2	0	2	3	3	3	1
1	1	1	0	0	2	2	0	2
4	0	0	3	3	3	3	4	4
0	2	0	0	0	1	0	0	2
0	4	4	3	2	3	4	2	3
0	2	0	0	0	0	0	0	0
0	0	0	0	0	0	0	0	0
2	3	3	3	2	2	3	0	0
2	2	0	0	0	0	1	2	2
2	0	0	0	0	0	0	0	0
2	1	1	2	3	2	3	2	4
1	1	0	0	0	0	1	0	1
0	0	0	0	0	0	0	0	0
0	0	0	0	0	0	0	0	0
4	0	0	0	0	0	0	0	0
0	0	0	0	0	0	0	0	0
0	0	0	3	3	0	3	0	0
1	1	1	0	0	0	2	0	2
0	0	0	0	0	0	0	0	0
0	1	2	2	1	1	1	0	1
1	2	2	1	1	1	2	2	4
1	2	0	2	2	2	2	0	2
0	0	0	0	0	0	0	0	0
3	2	2	2	2	2	2	1	0
0	0	0	0	0	0	0	0	3
0	2	0	0	0	0	3	0	0
3	1	1	1	0	0	0	0	0
1	0	0	0	0	0	1	0	0
2	0	0	1	0	0	0	2	0
0	2	3	4	4	4	4	1	4
0	0	2	2	2	0	1	0	2
2	2	1	3	4	3	0	1	0
0	2	2	2	2	2	2	2	0
0	0	0	0	0	0	0	0	0
1	0	0	3	3	1	3	1	0

1	3	1	1	1	1	2	0	0
0	2	1	1	1	1	4	0	0
0	2	2	2	2	1	1	0	0
0	0	0	0	0	0	0	0	0
3	2	1	1	1	1	2	0	0
0	0	0	1	2	0	0	0	0
2	0	2	2	2	2	2	2	0
1	0	2	0	0	0	0	0	0
4	3	3	3	2	2	3	4	4
2	1	0	1	1	1	2	1	2
3	2	2	3	3	2	2	2	2
3	3	3	3	3	2	4	0	1
0	0	0	2	2	0	0	0	0
2	1	0	1	1	1	2	1	2
0	2	4	0	0	0	0	0	1
0	3	3	3	0	0	3	0	3
1	2	2	2	3	2	1	1	1
1	1	1	2	2	2	2	2	3
3	3	3	3	3	3	3	2	3
0	0	0	0	0	0	3	3	0
1	2	2	3	3	3	3	1	0
2	1	4	4	4	4	4	2	0
2	4	4	4	4	3	3	0	0
4	4	4	4	4	4	4	2	0
2	4	4	4	4	4	4	3	2
2	4	3	3	3	3	2	0	0
4	3	3	3	3	3	3	0	1
2	3	2	3	3	3	3	2	2
3	4	4	4	4	4	4	4	0
3	3	3	4	4	4	4	0	0
1	1	2	4	4	0	1	0	0
1	2	1	2	2	2	3	1	4
0	2	1	1	1	1	1	1	1
4	3	3	4	3	3	2	3	3
2	3	3	2	2	3	3	3	2
0	0	0	0	0	0	3	0	0
1	3	3	1	1	1	3	0	0
0	1	1	3	3	3	4	0	0
0	0	0	0	0	0	2	0	1
1	1	2	2	2	2	2	2	1
4	3	3	2	2	2	4	2	3
1	2	2	2	2	2	2	2	3

2	4	4	4	4	4	4	4	1
0	3	3	3	3	3	3	2	0
2	2	2	2	2	0	3	2	2
0	0	1	1	2	2	2	0	1
0	0	0	0	0	0	0	3	0
0	4	4	4	4	4	4	4	1
3	3	3	3	3	3	2	0	1
0	0	0	0	0	0	2	0	1
0	0	0	0	0	0	0	0	0
0	0	0	0	0	0	0	0	0
1	2	3	3	3	3	3	0	1
0	0	0	0	0	0	0	0	0
3	4	4	4	4	0	4	1	1
3	4	4	4	4	4	4	3	3
1	3	3	3	3	2	2	2	1
3	1	3	3	3	3	3	2	1
4	4	4	4	4	4	4	4	2
0	4	4	4	4	4	3	0	0
2	3	4	3	3	3	2	0	2
0	3	3	4	4	4	2	2	0
4	4	4	4	4	4	4	0	0
3	1	1	3	3	3	2	2	3
3	4	4	4	4	4	4	2	2
4	3	1	4	4	3	4	1	3
1	3	3	3	3	3	3	2	1
2	3	3	3	3	3	3	2	1
0	2	3	3	3	4	3	0	0
0	1	2	1	0	3	3	0	4
2	3	2	3	4	2	3	3	4
0	0	0	0	0	0	2	0	1
0	0	1	3	3	3	3	0	0
2	4	4	3	4	4	4	4	1
0	0	0	1	2	3	3	0	3
3	3	3	2	2	2	2	2	1
1	3	3	3	3	3	3	3	3
2	3	3	2	2	2	3	0	3
0	3	3	0	0	0	4	0	0
2	3	4	4	4	3	2	3	2
3	3	3	3	3	3	4	3	3
1	1	2	2	2	2	2	0	1
1	1	1	1	1	1	1	2	0
1	1	1	1	1	1	1	2	0
2	2	2	2	2	2	2	2	3
0	3	3	3	3	3	2	0	0

3	4	4	2	4	3	3	1	1
2	2	2	3	3	3	2	2	2
1	1	1	1	1	1	1	1	1
1	1	1.5	2.5	2.5	2	2	1.5	0.5
2	2	4	3	4	1	1	1	1
2	3	3	3	3	3	1	1	3
2	4	4	4	3	3	3	3	1
0	3	3	4	4	4	4	2	3
1	2	2	3	3	3	2	1	1
2	2	3	2	2	2	2	3	3
0	3	3	3	3	3	2	0	3
0	0	0	2	2	0	1	0	0
1	4	4	4	4	4	4	3	4
4	0	4	4	4	4	4	4	4
0	0	2	2	2	2	4	0	1
3	4	4	4	4	4	4	4	3
3	3	3	3	3	3	2	0	3
2	0	0	0	0	0	1	0	3
0	0	0	0	0	0	0	0	0
3	4	4	4	4	4	4	0	2
0	4	0	1	4	3	3	1	3
0	2	0	2	2	1	1	0	0
0	2	0	2	2	1	1	0	0
3	2	2	3	3	3	2	1	3
2	0	0	0	0	0	0	0	3
3	2	2	2	0	2	2	1	1
4	0	4	4	4	4	4	4	4
4	4	2	2	2	2	3	2	2
1	2	3	1	2	4	4	0	1
0	0	2	2	2	2	4	0	1
2	2	2	3	3	2	3	3	0
2	4	4	4	4	4	4	3	3
3	2	2	3	3	3	2	1	3
4	4	4	4	4	4	4	4	4
0	1	1	3	3	3	4	0	0
3	4	4	4	4	4	3	3	4
3	3	3	2	2	1	3	0	2
3	1	1	1	1	0	2	2	2
2	4	4	4	4	4	4	3	3
4	3	3	2	2	2	4	2	3

0	3	3	3	3	3	3	2	0
4	3	3	2	2	2	4	2	3
0	1	0	0	0	0	1	2	0
2	3	2	3	4	2	3	3	4
0	3	2	2	2	3	3	3	1
1	4	4	4	4	4	4	3	4
1	2	2	3	3	3	2	1	1
0	3	3	0	0	0	4	0	0
2	4	4	4	4	4	4	4	1
3	3	3	3	3	3	2	0	1
1	2	3	3	3	3	3	0	1
0	0	0	1	2	3	3	0	3

0	0	0	0	0	0	2	0	1
1	1	2	2	2	2	2	2	1
1	3	3	3	3	3	3	0	1
1	3	3	2	3	3	1	0	0
1	2	2	3	3	2	2	1	1
0	0	0	2	2	0	0	0	0

2	2	2	2	2	2	2	0	0
0	0	0	0	0	0	0	0	0
0	0	0	4	4	3	0	0	2
4	0	0	1	1	1	0	0	0
0	0	0	0	0	0	0	0	0
3	1	0	0	0	0	1	2	0
0	0	0	1	0	0	0	0	0
0	0	0	0	0	0	0	1	0
0	0	0	0	0	0	0	0	0
1	2	2	2	3	2	1	0	1
0	0	0	0	0	0	0	0	0
1	1	1	1	1	1	1	1	1
0	0	0	0	0	0	3	0	0
1	0	0	0	0	0	0	0	0

0	0	0	0	0	0	0	0	0
0	0	0	0	0	0	0	0	0
0	0	0	1	1	0	0	0	0
0	0	0	0	0	0	0	0	0
0	0	0	0	0	0	0	0	0
1	1	0	0	0	0	1	1	0
0	0	0	0	0	0	0	0	0
0	0	0	0	0	0	0	0	0
0	0	0	0	0	0	0	0	0
1	0	0	0	0	0	0	0	2
0	0	0	0	0	0	0	0	0
0	1	1	0	1	0	0	0	1
0	2	0	0	0	0	0	0	0
0	0	0	0	0	0	0	0	0
0	1	0	1	1	0	0	0	0
0	0	0	0	0	0	0	0	0
0	0	0	0	0	0	0	0	0
1	0	0	0	0	0	0	0	0
0	0	0	0	0	0	0	0	0
0	0	0	3	3	0	1	1	1
0	0	0	0	0	0	0	0	0
1	0	0	0	0	0	0	1	0
0	0	0	0	0	0	0	0	0
0	0	0	0	0	0	0	0	0
0	0	0	0	0	0	0	0	0
0	0	0	0	0	0	0	0	0
0	0	0	0	0	0	0	0	0
0	0	0	0	0	0	0	0	0
0	0	0	0	0	0	0	0	0
0	0	0	0	0	0	0	0	0
0	0	0	0	0	0	0	0	0
0	0	0	0	0	0	0	0	0
0	0	0	0	0	0	0	0	0
1	3	2	2	3	2	4	1	2
0	4	3	3	4	4	3	0	3
1	2	2	2	2	2	3	1	0
2	2	0	0	0	0	0	0	1
1	0	0	0	0	0	0	0	0
0	0	0	0	0	0	0	0	0
0	0	0	0	0	0	0	0	0
0	0	0	0	0	0	0	0	0

0	0	0	0	0	0	0	1	0

CMSI bladd	CMSI urina	CMSI pelvic	CMSI burni	CMSI Rheu	CMSI Dyspi	CMSI Cardi	CMSI Head	CMSI Neuro
0	2	0	0	23	10	2	4	8
0	0	4	0	31	20	8	0	15
4	4	3	2	29	0	6	0	13
0	3	0	0	18	14	2	0	10
0	0	3	0	19	20	8	3	10
4	4	3	0	21	15	3	0	12
0	0	0	0	15	0	0	3	2
0	0	0	4	19	5	5	2	12
2	3	3	3	24	10	11	5	9
0	0	0	0	11	2	0	0	0
0	3	0	3	17	0	0	0	7
3	1	4	3	22	8	4	6	5
3	3	3	0	20	7	2	3	8
0	0	2	0	24	5	0	7	6
0	2	0	0	13	3	0	0	7
0	0	0	0	28	18	3	4	5
3	3	3	0	16	8	0	3	11
0	0	0	0	19	0	0	0	5
0	0	0	0	15	8	2	0	8
3	3	2	1	29	10	7	0	9
0	2	2	0	13	8	2	5	7
0	0	0	0	16	5	2	6	4
4	4	4	2	29	5	3	4	9
0	0	1	0	12	1	2	4	2
1	0	0	0	17	0	1	1	7
1	1	0	0	14	5	0	3	9
0	3	0	0	11	10	5	3	7
0	0	0	0	0	0	0	0	0
0	4	1	0	19	5	2	4	7
2	2	1	0	11	3	1	0	8
0	0	0	0	23	11	7	1	6
2	0	0	0	19	3	7	3	6
2	4	3	1	35	12	10	7	13
0	0	0	0	15	3	1	2	9
0	0	0	0	6	6	0	0	1
0	0	0	0	12	11	3	6	5
0	0	0	0	7	0	2	0	1
0	0	0	0	22	13	5	3	8

3	3	3	0	15	8	4	5	9
2	2	2	0	27	5	3	4	11
0	2	3	0	11	4	7	4	5
0	0	0	0	31	11	6	7	14
1	2	0	0	21	14	0	2	6
4	4	2	2	34	18	12	4	15
3	3	0	1	17	15	2	0	7
0	2	0	0	16	10	0	0	0
0	0	0	0	21	2	0	6	11
1	0	0	0	24	2	2	5	5
0	0	0	0	20	12	2	7	12
3	4	0	1	31	8	7	5	12
0	0	0	0	10	3	0	2	3
0	0	0	0	12	11	0	3	1
0	0	0	0	4	0	0	0	6
0	0	0	0	9	0	0	4	5
0	0	0	0	10	0	1	1	5
0	0	0	0	9	0	1	0	5
0	2	0	0	13	5	2	0	3
0	3	0	0	19	12	6	2	1
0	1	0	0	18	5	4	0	7
3	3	2	0	22	6	9	2	7
0	0	0	0	18	8	3	0	10
0	0	0	0	7	7	1	0	4
0	0	1	0	9	0	0	4	3
0	0	0	0	11	12	0	0	5
0	0	0	0	11	8	3	3	5
0	0	0	0	10	2	2	0	6
2	2	0	0	21	13	2	0	5
0	0	0	0	13	3	4	0	7
0	0	4	0	14	2	0	5	5
0	2	0	0	8	0	1	3	3
4	4	3	0	17	1	1	2	3
0	1	1	0	26	1	6	3	10
1	0	0	0	19	12	3	0	6
0	0	3	0	23	1	0	2	8

0	0	1	0	13	1	0	1	6
2	1	0	0	18	14	3	0	5
1	0	0	0	12	0	1	3	6
0	0	0	0	7	1	0	2	1
0	0	0	0	13	0	0	3	5
0	0	0	0	12	0	2	0	8
0	0	0	0	8	14	5	0	2
0	1	1	0	13	8	6	0	9
4	4	3	0	14	11	2	1	6
0	1	2	0	16	5	2	2	5
3	3	2	2	26	6	9	5	9
2	0	1	0	11	2	1	1	5
				0	0	0	0	0
0	0	0	0	12	2	0	6	6
0	1	2	0	16	5	2	2	5
				0	0	0	0	0
0	0	0	0	13	2	0	6	7
0	0	0	0	16	2	1	6	7
1	0	0	0	33	16	4	8	13
0	0	0	0	20	13	3	3	8
1	1	2	0	18	2	0	3	10
0	0	0	0	18	13	7	4	8
0	0	0	0	20	9	11	1	6
2	2	0	0	28	5	0	3	6
0	1	0	0	20	9	2	2	7
3	2	1	1	20	2	1	3	7
2	3	0	0	37	17	4	7	10
0	0	0	0	21	6	6	5	10
2	3	3	3	28	7	7	6	7
3	3	1	0	26	15	7	8	11
1	1	3	0	33	12	4	4	12
0	0	3	1	26	8	2	4	8
1	1	2	0	23	13	2	0	6
0	0	3	0	12	16	4	8	11
2	2	0	1	19	10	0	1	5
3	4	2	0	36	16	12	7	14
3	3	3	1	35	16	12	4	12
0	0	0	0	14	6	1	2	8
0	0	0	0	17	11	0	4	8
0	0	4	0	18	12	6	1	7
1	1	0	1	23	10	8	2	11
3	3	1	1	22	11	8	6	11
2	3	2	3	22	4	7	8	13
1	1	2	0	22	8	9	8	10

1	1	1	0	26	6	4	4	11
0	0	4	0	13	2	9	0	8
0	0	1	0	15	15	6	5	3
1	0	0	0	18	8	4	4	7
0	0	0	0	19	1	0	0	10
1	1	3	0	20	5	1	8	9
0	0	0	0	16	0	0	3	7
0	1	1	0	27	1	3	2	9
0	0	0	0	13	4	0	0	0
0	0	0	0	6	0	0	0	3
0	0	0	0	17	5	0	4	6
0	0	0	0	14	3	4	5	6
4	4	0	0	22	11	4	6	11
2	3	2	1	35	18	13	8	14
3	3	0	0	20	8	3	3	8
2	2	3	3	32	18	12	8	16
2	2	4	0	38	11	1	8	16
1	0	0	1	22	7	2	3	9
1	1	1	0	26	2	0	0	13
1	0	4	0	34	5	11	5	15
0	4	0	0	25	5	3	0	16
2	0	1	0	31	11	1	6	12
4	4	4	0	32	11	9	6	11
3	4	1	0	21	16	7	5	13
3	3	4	3	35	11	15	0	12
0	4	0	0	14	3	3	6	10
0	3	0	0	19	10	2	4	10
0	0	1	0	18	10	1	1	7
0	0	0	0	17	9	4	4	10
0	0	0	0	24	6	4	4	10
0	0	3	0	14	5	1	0	7
4	3	4	0	29	9	4	7	5
0	1	3	1	25	15	9	7	13
2	2	1	0	22	13	6	6	6
1	1	3	0	24	8	1	5	12
1	1	3	0	18	15	1	5	13
3	3	3	0	20	0	0	8	7
1	1	2	1	23	8	6	5	11
2	2	3	1	26	20	8	6	13
4	4	0	0	29	6	0	5	14
0	0	1	0	18	5	2	2	10
0	0	1	0	18	5	2	2	10
0	0	0	0	16	1	0	2	6
0	1	0	0	20	20	2	4	14

2	3	1	0	32	13	4	7	8
3	2	0	0	16	7	5	6	11
1	1	1	1	25	7	7	5	8
2.5	3	1.5	0.5	25	8	8	7	11.5
1	3	1	1	18	3	1	6	7
3	1	0	0	23	5	6	7	6
4	3	4	4	23	20	4	6	4
0	3	4	0	18	15	8	2	10
0	0	1	0	16	4	5	1	6
3	2	2	3	21	9	5	6	10
1	3	0	2	27	14	10	1	14
0	0	0	0	5	3	0	0	4
3	3	3	0	35	20	5	8	11
0	0	4	0	29	19	0	8	11
3	0	4	0	34	8	2	4	14
1	1	4	2	24	9	6	8	11
1	1	1	0	30	11	7	4	9
0	1	0	0	20	5	2	3	9
1	3	0	2	16	0	0	8	4
3	4	0	2	27	11	2	3	7
0	4	1	0	23	12	12	3	11
0	1	1	3	23	8	0	2	6
0	1	1	3	23	8	0	2	6
3	4	4	1	29	18	5	6	15
1	0	0	0	22	5	4	1	5
3	3	2	2	17	5	0	6	5
0	0	4	0	29	19	0	8	11
2	0	3	0	32	15	5	8	8
0	0	4	1	36	13	7	6	14
3	0	4	0	34	8	2	4	14
2	2	3	0	22	9	5	4	8
4	4	4	1	37	20	9	8	13
3	4	4	1	29	18	5	6	15
4	4	4	4	44	20	16	8	16
0	0	4	0	18	12	6	1	7
3	3	3	3	42	15	5	8	12
0	0	0	0	18	18	9	6	5
2	2	2	0	22	9	2	6	6
3	3	3	0	38	16	6	7	12
2	3	2	3	22	4	7	8	13

0	0	0	0	13	11	0	3	3
2	3	2	3	22	4	7	8	13
1	1	0	0	16	1	0	3	4
0	0	0	0	17	9	4	4	10
0	0	0	0	15	10	1	4	4
3	3	3	0	35	20	5	8	11
0	0	1	0	16	4	5	1	6
3	3	3	0	20	0	0	8	7
1	1	1	0	26	6	4	4	11
0	0	0	0	16	0	0	3	7
0	0	0	0	17	5	0	4	6
0	1	3	1	25	15	9	7	13
0	1	1	0	27	1	3	2	9
3	3	1	1	22	11	8	6	11
0	0	1	0	19	5	5	2	6
0	0	0	0	5	0	0	2	3
0	0	0	0	21	7	3	0	5
0	0	0	0	12	2	0	6	6

0	3	3	3	15	2	0	6	2
0	0	0	0	0	0	0	0	0
3	4	1	2	22	0	0	4	14
0	0	3	2	7	10	2	0	11
0	0	0	0	0	0	0	0	0
0	2	0	0	15	2	1	2	6
0	0	0	0	8	4	0	0	2
1	2	0	0	23	0	0	0	3
0	0	0	0	8	0	2	1	6
2	2	1	4	24	6	5	4	8
0	0	0	0	14	3	0	3	3
1	2	1	1	20	5	6	4	4
0	0	0	0	6	0	0	0	0
0	0	0	0	3	4	0	0	0

0	0	0	0	0	0	0	0	0
0	0	0	0	3	2	0	0	0
0	0	0	0	0	0	0	0	0
0	0	0	0	0	0	0	0	0
0	0	0	0	0	0	0	0	0
0	0	0	0	6	1	0	1	2
0	0	0	0	3	0	0	0	0
0	0	0	0	0	0	0	0	0
0	0	0	0	0	0	0	0	0
0	1	1	0	15	4	6	6	6
0	0	0	0	11	1	0	2	8
0	0	0	1	4	1	0	4	1
0	0	1	0	1	0	1	0	0
0	0	0	0	0	0	0	0	0
0	2	0	0	7	1	0	0	1
0	0	0	0	4	0	0	0	0
0	0	0	0	3	1	0	0	1
0	1	0	0	1	2	0	0	0
0	0	0	0	1	0	0	0	0
0	0	0	0	4	5	1	0	2
0	0	0	0	2	2	1	1	1
2	1	0	0	7	0	0	1	1
0	0	0	0	0	2	1	0	1
0	0	0	0	2	1	0	0	1
0	0	0	0	0	0	0	0	0
0	0	0	0	2	1	0	0	1
0	0	0	0	1	1	1	2	0
0	0	0	0	8	0	0	6	3
0	0	0	0	0	0	0	0	0
0	0	0	0	4	1	0	0	0
0	0	0	0	0	0	0	0	0
0	0	0	0	2	1	0	0	1
0	0	0	0	2	3	0	0	0
1	1	1	0	22	9	3	0	8
0	0	0	0	12	4	2	4	8
0	0	0	0	4	0	0	6	1
3	4	0	0	9	5	1	3	2
0	0	0	0	2	0	0	0	1
0	0	0	0	0	0	0	0	0
0	0	0	0	0	0	0	0	0
0	0	0	0	0	0	0	0	0

0	0	0	0	3	0	0	0	1

CMSI Ear&S	CMSI GI	CMSI Bladc	CMSI172	CMSI no PAIN		GAD feelin	GAD contrc	GAD worryi
2	3	2	54	31				
3	8	4	89	58		0	0	0
10	7	13	78	49		2	1	1
0	0	3	68	50		0	0	0
6	15	3	84	65		1	1	1
4	10	11	76	55		0	0	0
10	0	0	30	15				
0	8	4	55	36				
5	19	11	94	70				
0	0	0	13	2				
3	17	6	50	33		2	2	2
2	0	11	58	36		3	2	3
4	18	9	71	51		1	3	3
6	0	2	50	26		0	0	0
0	6	2	31	18		3	0	0
3	0	0	61	33				
14	29	9	90	74		0	0	0
						1	0	0
1	11	0	36	17				
						3	3	3
						1	1	1
10	15	0	58	43		0	0	0
3	20	9	87	58		2	2	2
8	5	4	52	39		2	2	2
3	5	0	41	25		1	1	1
2	17	14	83	54		0	0	0
0	1	1	23	11		0	0	0
1	7	1	35	18		2	2	2
1	0	2	34	20		0	0	0
3	3	3	45	34		1	0	0
0	0	0	0	0		0	0	0
8	12	5	62	43		2	3	3
0	6	5	34	23		1	1	1
2	15	0	65	42		1	2	1
2	5	2	47	28		1	0	1
12	19	10	118	83		3	3	3
0	15	0	45	30		1	0	1
2	0	0	15	9		0	0	0
2	8	0	47	35		0	0	0
0	9	0	19	12		1	1	1
7	5	0	63	41		0	1	2

7	14	9	71	56	1	0	1
12	16	6	84	57	1	1	1
2	8	5	46	35	0	0	0
8	20	0	97	66	2	1	1
1	5	3	52	31	1	1	1
4	25	12	124	90			
7	2	7	57	40	0	0	0
0	0	2	28	12	1	1	1
10	16	0	66	45	3	3	3
5	7	1	51	27	0	0	1
2	0	0	55	35	1	1	0
6	18	8	95	64			
1	3	0	22	12	0	0	0
1	0	0	28	16	0	0	0
3	0	0	13	9	3	0	0
4	0	0	22	13	0	0	0
0	0	0	17	7	1	1	1
0	9	0	24	15	1	1	1
1	6	2	32	19			
6	0	3	49	30	1	0	0
1	9	1	45	27	1	1	1
5	15	8	74	52	0	0	0
2	12	0	53	35	0	0	0
4	0	0	23	16	1	1	1
8	13	1	38	29	2	0	0
4	3	0	35	24	0	0	0
0	5	0	35	24	1	1	1
4	3	0	27	17	1	0	1
4	1	4	50	29	0	0	1
6	3	0	36	23	1	0	1
1	26	4	57	43			
1	9	2	27	19	0	0	1
11	14	11	60	43	1	2	2
					1	0	0
0	14	2	62	36	0	0	0
7	0	1	48	29	1	1	1
10	11	3	58	35	1	0	0

5	9	1	36	23	0	0	0
4	10	3	57	39	0	0	0
5	10	1	38	26	2	1	1
1	0	0	12	5	0	0	0
3	8	0	32	19	0	0	0
4	3	0	29	17	0	0	1
9	12	0	50	42	1	0	0
2	2	2	42	29	1	1	1
11	24	11	80	66	0	0	0
7	9	3	49	33	3	3	3
11	18	10	94	68	1	1	0
11	19	3	53	42	1	1	1
0	0	0	0	0	3	1	1
6	4	0	36	24	0	0	0
7	9	3	49	33	3	3	3
0	0	0	0	0			
7	7	0	42	29	0	0	0
5	15	0	52	36	0	0	0
8	14	1	97	64	1	0	1
9	15	0	71	51	1	1	1
3	23	4	63	45	3	1	1
4	6	0	60	42	1	2	3
8	17	0	72	52	0	0	1
8	23	4	77	49	1	1	1
10	22	1	73	53	1	1	2
13	26	7	79	59	1	1	1
16	29	5	125	88	3	3	3
15	18	0	81	60	3	0	1
9	19	11	94	66	3	1	1
10	21	7	105	79	3	3	3
8	28	5	106	73	2	2	2
5	22	4	79	53	1	2	3
5	12	4	65	42	0	0	0
3	17	3	74	62	3	0	0
2	9	5	51	32	3	1	2
11	24	9	129	93	1	1	1
11	21	10	121	86	2	2	2
3	3	0	37	23	0	2	2
1	12	0	53	36	2	2	3
8	15	4	71	53	3	3	3
2	3	3	62	39	1	0	0
11	14	8	91	69	0	0	0
16	21	10	101	79	3	1	3
11	17	4	89	67	3	3	3

10	29	3	93	67	2	2	2
6	20	4	62	49	3	3	3
2	15	1	62	47	0	0	0
6	9	1	57	39	1	2	2
6	3	0	39	20	0	0	0
6	29	5	83	63	0	0	1
8	18	0	52	36	2	1	2
6	3	2	53	26	1	0	1
0	0	0	17	4	1	0	0
2	0	0	11	5	0	0	1
6	18	0	56	39	2	1	1
0	0	0	32	18	1	0	1
12	22	8	96	74	3	2	2
13	30	8	139	104	3	3	3
3	19	6	70	50	1	1	1
13	19	10	128	96	3	3	3
20	30	8	132	94	2	0	0
7	23	2	75	53	2	2	2
8	20	3	72	46	1	1	2
12	22	5	109	75	0	0	0
20	24	4	97	72	1	1	0
12	18	3	94	63	0	0	0
9	28	12	118	86	1	0	0
8	23	8	101	80	3	1	2
13	21	13	120	85	2	2	2
4	21	4	65	51	0	0	0
9	18	3	75	56	2	0	2
1	14	1	53	35	0	0	0
8	24	0	76	59	2	1	1
7	3	0	58	34	2	0	1
4	13	3	47	33	3	3	3
3	28	11	96	67	3	1	1
4	12	5	90	65	2	3	3
8	17	5	83	61	1	0	0
8	24	5	87	63	2	2	2
10	18	5	85	67	2	2	2
3	10	9	57	37	0	0	0
10	25	5	93	70	3	1	1
11	25	8	117	91	0	1	1
1	12	8	75	46	3	3	3
3	8	1	49	31	1	2	2
3	8	1	49	31	1	2	2
3	17	0	45	29	1	2	2
3	17	1	81	61	3	3	3

8	22	6	100	68	2	2	3
9	19	5	78	62	2	2	1
5	8	4	69	44	2	1	1
10	13.5	7.5	90.5	65.5	2	1	1
9	17	6	67	49	0	0	1
10	20	4	81	58	2	3	3
8	25	15	105	82	2	2	1
3	27	7	90	72	1	0	1
3	17	1	53	37	1	0	0
12	19	10	92	71	1	1	1
6	20	6	98	71	2	2	3
1	5	0	18	13	0	0	0
13	31	9	132	97	2	1	1
19	28	4	118	89	1	2	2
5	13	7	87	53	0	0	0
10	31	8	107	83	3	3	3
10	20	3	94	64	2	2	2
7	4	1	51	31	2	3	3
5	0	6	39	23	0	0	0
7	26	9	92	65	3	2	2
8	19	5	93	70	3	3	1
5	8	5	57	34	2	1	1
5	8	5	57	34	2	1	1
15	19	12	119	90	1	3	3
8	3	1	49	27	2	1	1
3	12	10	58	41	1	0	0
					1	0	0
19	28	4	118	89	1	2	2
12	19	5	104	72	3	3	3
10	17	5	108	72	0	0	1
5	13	7	87	53	0	0	0
6	18	7	79	57	3	2	2
15	30	13	145	108	3	3	3
15	19	12	119	90	1	3	3
20	32	16	172	128	3	3	3
8	15	4	71	53	3	3	3
15	30	12	139	97			
3	16	0	75	57	1	0	1
7	10	6	68	46	1	0	0
15	30	9	133	95	2	2	2
16	21	10	101	79	3	1	3

3	20	0	53	40	3	3	3
16	21	10	101	79	1	1	1
0	4	2	30	14	0	0	0
8	24	0	76	59	2	1	1
4	19	0	57	42	3	3	3
13	31	9	132	97	2	1	1
3	17	1	53	37	1	0	0
3	10	9	57	37	0	0	0
10	29	3	93	67	2	2	2
8	18	0	52	36	2	1	2
6	18	0	56	39	2	1	1
4	12	5	90	65	2	3	3
6	3	2	53	26	1	0	1
11	14	8	91	69	0	0	0
3	19	1	60	41			
3	15	0	28	23	2	2	1
3	16	0	55	34	1	0	0
6	4	0	36	24	0	0	0

2	12	9	48	33	0	0	0
0	0	0	0	0	1	0	0
0	13	10	63	41			
11	3	5	49	42			
0	0	0	0	0	0	0	0
9	4	2	41	26	0	0	0
3	1	0	18	10	0	0	0
0	1	3	30	7	0	0	0
5	0	0	22	14	0	0	0
9	13	9	78	54	3	2	2
0	0	0	23	9	0	0	0
5	8	5	57	37			
0	3	0	9	3			
3	0	0	10	7	2	1	1

0	0	0	0	0	0	0	1
0	0	0	5	2	0	0	1
0	2	0	2	2	0	0	0
0	0	0	0	0	0	0	0
0	0	0	0	0	0	0	0
5	3	0	18	12	3	1	1
6	0	0	9	6	0	0	0
0	0	0	0	0	0	0	0
0	0	0	0	0	0	0	0
5	2	2	46	31	2	0	0
0	0	0	22	11	3	3	3
0	4	1	15	11	1	0	0
0	2	1	5	4	2	0	0
0	0	0	0	0	0	0	0
3	3	2	17	10	0	0	0
0	0	0	4	0	0	0	0
0	0	0	5	2	0	0	0
1	0	1	5	4	0	0	0
0	0	0	1	0	0	0	0
0	9	0	21	17	3	1	3
1	0	0	8	6	0	0	0
1	1	3	14	7	0	0	0
1	0	0	5	5	0	0	0
0	0	0	4	2	0	0	0
0	0	0	0	0	0	0	1
0	0	0	4	2	0	0	0
0	0	0	5	4	0	1	1
					2	2	2
2	0	0	19	11	0	0	0
0	0	0	0	0	0	0	0
					0	0	0
0	0	0	5	1	0	0	0
1	0	0	1	1	0	0	0
0	0	0	4	2	0	0	0
0	0	0	5	3	0	0	0
1	19	3	65	43			
9	24	0	63	51	1	0	1
1	14	0	26	22	2	2	2
5	3	7	35	26	0	0	0
4	0	0	7	5	0	0	0
0	0	0	0	0	0	0	0
0	0	0	0	0	1	0	0
0	0	0	0	0	1	1	1

1	1	0	6	3		0	0	0

GAD troubl	GAD hard_t	GAD easily_	GAD feeling	∑GAD7	GAD7≥10		ESR	CRP mg/L
							46	1.5
0	0	0	0	0	0			1.5
2	3	2	1	12	1		18	1.5
0	0	0	0	0	0		17	1.5
1	1	1	0	6	0		17	1.5
							3	1.5
0	0	0	0	0	0		5	1.5
							4	1.5
							5	1.5
							8	1.5
							5	1.5
2	1	1	1	11	1		27	1.5
3	1	3	2	17	1			1.5
2	1	2	2	14	1		3	1.5
2	0	0	0	2	0		9	1.5
3	0	2	0	8	0		2	1.5
							5	1.5
0	0	0	0	0	0		5	1.5
1	0	0	0	2	0		1	1.5
							3	1.5
0	3	2	3	17	1		22	1.5
3	0	3	1	10	1		32	1.5
0	0	1	0	1	0			1.5
2	2	2	2	14	1		4	1.5
							21	1.5
1	0	1	0	8	0		10	2
1	1	1	1	7	0			2.4
0	0	0	0	0	0		15	2.9
1	0	1	0	2	0		19	2.9
0	0	1	0	7	0		14	2.9
0	0	0	0	0	0		15	2.9
1	0	0	0	2	0		5	2.9
0	0	1	0	1	0		10	3
2	1	2	2	15	1		14	3
2	0	3	0	8	0		15	3.16
0	0	1	2	7	0		20	4.72
1	1	0	0	4	0		17	5.4
2	1	3	3	18	1		1	6
0	0	0	0	2	0		2	6
0	0	0	0	0	0		4	6
0	0	0	0	0	0		11	6
2	2	3	0	10	1			6.2
0	0	1	0	4	0		70	8

1	0	1	1	5	0	8	8.2
1	0	1	0	5	0		9
1	0	1	0	2	0	13	9.76
3	0	1	1	9	0	20	12
1	0	1	1	6	0	9	17.1
						19	23
1	1	0	0	2	0	12	46.9
1	1	1	1	7	0	73	164
3	2	2	3	19	1		
1	1	1	0	4	0		
1	0	1	1	5	0	6	
0	0	1	0	1	0	12	18
0	1	0	0	1	0		0.2
3	0	3	0	9	0		1.5
0	0	0	0	0	0		1.5
0	0	1	1	5	0	2	1.5
2	1	0	3	9	0	2	1.5
						15	1.5
0	0	0	0	1	0	28	1.5
1	1	2	1	8	0	2	2.9
0	0	0	0	0	0	45	2.9
0	0	1	0	1	0	50	2.9
1	1	1	1	7	0	2	2.9
1	0	1	0	4	0	2	2.9
0	0	0	0	0	0	12	2.9
0	0	0	0	3	0	10	3
1	0	1	0	4	0	2	4
1	1	1	0	4	0		6.1
							6.6
2	0	1	0	5	0	2	7
						12	
0	0	1	0	2	0		
						8	1.5
						8	1.5
							0.8
1	1	1	1	9	0		0.9
2	1	1	1	6	0		1.4
0	0	1	0	1	0	3	1.5
1	2	2	0	8	0		2.6
1	0	1	1	4	0	4	0

0	0	0	0	0	0		0.6
1	0	0	0	1	0	2	1.5
3	2	3	0	12	1	4	1.5
0	0	1	0	1	0	5	1.5
0	0	0	0	0	0		2.7
0	0	0	0	1	0		2.8
2	1	1	0	5	0	5	2.9
1	1	2	1	8	0		3.1
0	0	3	0	3	0		5.2
2	2	1	2	16	1	5	7
2	1	2	1	8	0	5	10
0	0	2	1	6	0		10.8
2	0	1	1	9	0	6	17
2	0	0	0	2	0	18	25
2	2	1	2	16	1		
0	0	0	0	0	0		
1	0	1	0	2	0	15	
0	0	2	1	5	0		0.2
0	0	1	1	5	0		0.2
1	0	2	0	8	0		0.2
3	3	3	2	17	1		0.2
2	0	2	0	5	0		0.2
1	1	1	1	7	0		0.3
2	2	1	2	11	1		0.3
1	1	2	1	8	0		0.4
3	3	3	3	21	1		0.4
0	0	3	0	7	0		0.5
2	1	1	0	9	0		0.7
3	3	3	3	21	1		0.9
2	0	3	2	13	1	8	1
3	1	2	2	14	1		1.1
3	1	1	0	5	0		1.1
2	1	3	0	9	0		1.3
1	1	0	1	9	0		1.3
3	1	3	0	10	1	20	1.5
2	2	2	1	13	1	2	1.5
2	2	2	2	12	1	3	1.5
3	2	2	1	15	1	9	1.5
3	2	0	2	16	1	3	1.5
1	0	1	0	3	0	9	1.5
0	0	0	0	0	0	1	1.5
3	2	1	1	14	1	36	1.5
3	3	3	3	21	1		1.5

2	0	2	2	12	1	3	1.5
3	3	3	3	21	1	2	1.5
1	1	0	0	2	0	1	1.5
3	3	2	1	14	1	10	1.5
1	2	1	0	4	0	1	1.5
1	0	0	0	2	0	5	1.5
3	2	3	2	15	1	5	1.5
2	0	3	0	7	0	5	1.5
1	0	1	0	3	0	14	1.5
0	0	0	0	1	0		1.5
1	1	1	0	7	0	2	1.5
0	1	1	0	4	0		1.5
3	3	3	2	18	1		1.7
3	3	3	3	21	1		2
1	0	2	1	7	0		2.2
3	3	3	3	21	1		2.4
2	1	3	2	10	1		2.4
2	2	3	2	15	1		2.4
2	2	3	1	12	1		2.5
0	0	0	0	0	0		2.7
1	0	3	0	6	0	3	2.9
1	0	3	0	4	0	2	2.9
2	1	3	1	8	0	20	2.9
2	3	2	0	13	1	15	2.9
2	2	2	1	13	1	9	2.9
0	0	1	0	1	0	15	2.9
1	1	1	0	7	0	7	2.9
1	0	1	0	2	0	15	2.9
2	2	2	0	10	1	4	3
3	1	3	0	10	1	3	3
3	3	2	2	19	1	9	3
2	1	3	1	12	1	10	3.1
3	2	3	1	17	1		3.1
1	1	2	0	5	0		4.2
1	1	3	1	12	1	3	4.81
2	2	2	2	14	1	2.2	5
0	0	0	0	0	0	6	5
2	2	1	0	10	1	3	5
0	1	2	1	6	0	3	5.41
3	3	3	2	20	1		5.6
3	2	2	3	15	1	10	6
3	2	2	3	15	1	0.1	6
2	3	3	3	16	1	6	6
3	1	3	2	18	1		6.4

3	2	3	1	16	1	34	6.67
2	2	1	3	13	1	13	6.9
2	1	2	0	9	0	4	7
1	0	1	1	7	0	1	8
0	0	0	0	1	0	20	8
2	2	2	3	17	1	5	8
2	2	1	1	11	1	5	8
						1	8
2	0	3	0	7	0	42	8
1	1	1	1	5	0		8.1
2	2	1	1	9	0		8.3
3	2	2	2	16	1		8.5
2	0	3	0	5	0	7	8.5
2	2	2	3	13	1	7	9
2	3	1	2	13	1	0	10
1	1	0	0	2	0	20	10
						2	10
3	3	3	3	21	1	24	10
2	2	2	1	13	1	20	11
2	1	2	3	16	1	7	11
0	0	0	0	0	0		11
2	2	3	1	15	1	18	12
1	0	3	0	11	1	30	14
1	2	2	0	9	0	19	15.5
1	2	2	0	9	0	19	15.5
1	1	3	3	15	1	35	16
1	1	2	3	11	1	17	24
1	0	3	0	5	0	2	31
0	0	1	0	2	0	30	68
2	3	1	2	13	1		
3	3	3	2	20	1	3	
0	0	1	0	2	0		
1	1	0	0	2	0		
3	3	3	0	16	1		
3	3	3	3	21	1		
1	1	3	3	15	1		
3	3	3	3	21	1		
3	2	0	2	16	1	3	
3	1	0	0	6	0		
0	0	1	0	2	0	20	
2	2	2	2	14	1		
3	2	1	1	14	1		

3	1	3	0	16	1	8	
1	2	1	1	8	0		
0	0	0	0	0	0	7	
2	2	2	0	10	1		
3	1	3	0	16	1		
2	2	2	3	13	1		
1	1	1	1	5	0		
0	0	0	0	0	0		
2	0	2	2	12	1	3	
3	2	3	2	15	1	5	
1	1	1	0	7	0		
3	2	3	1	17	1		
2	0	3	0	7	0		
0	0	0	0	0	0		
1	1	1	1	9	0	5	
1	0	1	1	4	0	4	
2	0	0	0	2	0		
						2	
						14	12
						3	6
						10	3
							1.9
						2	18
2	1	1	0	4	0	7	1.5
0	0	0	0	1	0	0	9
						3	1.5
						10	1.5
0	0	1	0	1	0	5	1.5
0	0	0	0	0	0	17	2.9
0	0	1	0	1	0	1	2.9
0	0	0	0	0	0	10	2.9
0	0	0	0	0	0		
2	1	3	1	14	1	4	1.5
0	0	1	0	1	0		0.5
						7	1.5
						6	1.5
3	1	2	0	10	1	4	1.5

1	1	0	0	3	0	4	1.5
0	0	0	0	1	0	5	1.5
0	0	0	0	0	0	12	1.5
0	0	0	0	0	0	7	1.5
0	0	0	0	0	0	3	1.5
2	2	1	1	11	1	1	1.5
0	0	0	0	0	0	0	1.5
0	0	0	0	0	0	4	1.5
0	0	0	0	0	0	1	2
1	0	1	0	4	0	2	2.9
3	3	3	3	21	1	5	2.9
0	0	1	1	3	0	3	2.9
0	0	1	1	4	0	4	2.9
0	0	0	0	0	0	4	2.9
0	0	0	0	0	0	20	2.9
0	0	0	0	0	0	2	2.9
0	0	0	0	0	0	7	3
0	0	0	0	0	0		3
1	0	1	0	2	0	5	3
0	1	1	1	10	1	1	3
0	0	1	0	1	0	3	3.1
0	0	0	0	0	0	15	3.1
0	0	0	0	0	0	6	3.83
0	0	0	0	0	0	12	5
0	0	0	0	1	0	5	5
0	0	0	0	0	0	32	5
0	0	1	0	3	0	13	6
2	1	2	0	11	1	5	6
0	0	0	0	0	0	16	6.6
0	1	1	0	2	0	2	7
0	0	0	0	0	0	1	8
0	3	0	0	3	0	37	8.19
0	0	0	0	0	0	3	9
0	0	0	0	0	0	7	11
0	0	0	0	0	0	33	16.4
						19	19
1	0	0	0	3	0	10	21.4
2	2	1	0	11	1	30	30.8
0	0	0	0	0	0	1	
0	0	0	0	0	0	1	
0	0	0	0	0	0		
0	0	1	0	2	0		
1	0	1	1	6	0	6	
						27	5

0	0	1	0	1	0		0	2
							3	2.9
							5	16
								1.5

GWI CCC S	CIF Quad
CCC	CFS
CCC	CFS
CCC	CFS
CCC	CFS
CCC	CFS
CCC	CFS
CCC	CFS
CCC	CFS
CCC	CFS
CCC	CFS
CCC	CFS
CCC	CFS
CCC	CFS
CCC	CFS
CCC	CFS
CCC	CFS
CCC	CFS
CCC	CFS
CCC	CFS
CCC	CFS
CCC	CFS
CCC	CFS
CCC	CFS
CCC	CFS
CCC	CFS
CCC	CFS
CCC	CFS
CCC	CFS
CCC	CFS
CCC	CFS
CCC	CFS
CCC	CFS
CCC	CFS
CCC	CFS
CCC	CFS
CCC	CFS
CCC	CFS
CCC	CFS
CCC	CFS
CCC	CFS
CCC	CFS
CCC	CFS
CCC	CFS

CCC	CFS
CCC	CFS
CCC	CFS
CCC	CFS
CCC	CFS
CCC	CFS
CCC	CFS
CCC	CFS
CCC	CFS
CCC	CFS
CCC	CFS
CCC	CFS
CCC	CFS
CCC	CFS
CCC	CFSLWIFS
CCC	CIF (CFS)
CCC	CIF (CFS)
CCC	CIF (CFS)
CCC	CIF (CFS)
CCC	CIF (CFS)
CCC	CIF (CFS)
CCC	CIF (CFS)
CCC	CIF (CFS)
CCC	CIF (CFS)
CCC	CIF (CFS)
CCC	CIF (CFS)
CCC	CIF (CFS)
CCC	CIF (CFS)
CCC	CIF (CFS)
CCC	CIF (CFS)
CCC	CIF (CFS)
CCC	CIF (CFS)
CCC	CIF (CFS)
CCC	CIF (CFS)
CCC	CIF (CFS)
CCC	
CCC	
CCC	
CCC	
GWI	CFSLWIFS
GWI	CFSLWIFS
GWI	CFSLWIFS
GWI	CFSLWIFS
GWI	CIF (GWI)

GWI	CIF (GWI)
GWI	CIF (GWI)
GWI	CIF (GWI)
GWI	CIF (GWI)
GWI	CIF (GWI)
GWI	CIF (GWI)
GWI	CIF (GWI)
GWI	CIF (GWI)
GWI	CIF (GWI)
GWI	CIF (GWI)
GWI	CIF (GWI)
GWI	CIF (GWI)
GWI	CIF (GWI)
GWI	CIF (GWI)
GWI	CIF (GWI)
GWI	CIF (GWI)
GWI	CIF (GWI)
GWI	CIF (GWI)
GWI	GWI
GWI	GWI
GWI	GWI
GWI	GWI
GWI	GWI
GWI	GWI
GWI	GWI
GWI	GWI
GWI	GWI
GWI	GWI
GWI	GWI
GWI	GWI
GWI	GWI
GWI	GWI
GWI	GWI
GWI	GWI
GWI	GWI
GWI	GWI
GWI	GWI
GWI	GWI
GWI	GWI
GWI	GWI
GWI	GWI
GWI	GWI
GWI	GWI
GWI	GWI

GWI	GWI
GWI	GWI
GWI	GWI
GWI	GWI
GWI	GWI
GWI	GWI
GWI	GWI
GWI	GWI
GWI	GWI
GWI	GWI
GWI	GWI
GWI	GWI
GWI	GWI
GWI	GWI
GWI	GWI
GWI	GWI
GWI	GWI
GWI	GWI
GWI	GWI
GWI	GWI
GWI	GWI
GWI	GWI
GWI	GWI
GWI	GWI
GWI	GWI
GWI	GWI
GWI	GWI
GWI	GWI
GWI	GWI
GWI	GWI
GWI	GWI
GWI	GWI
GWI	GWI
GWI	GWI
GWI	GWI
GWI	GWI
GWI	GWI
GWI	GWI
GWI	GWI
GWI	GWI
GWI	GWI
GWI	GWI
GWI	GWI
GWI	GWI

GWI	GWI
GWI	GWI
GWI	GWI
GWI	GWI
GWI	GWI
GWI	GWI
GWI	GWI
GWI	GWI
GWI	GWI
GWI	GWI
GWI	GWI
GWI	GWI
GWI	GWI
GWI	GWI
GWI	GWI
GWI	GWI
GWI	GWI
GWI	GWI
GWI	GWI
GWI	GWI
GWI	GWI
GWI	GWI
GWI	GWI
GWI	GWI
GWI	GWI
GWI	GWI
GWI	GWI
GWI	GWI
GWI	GWI
GWI	GWI
GWI	GWI
GWI	GWI
GWI	GWI
GWI	GWI
GWI	GWI
GWI	GWI
GWI	GWI
GWI	GWI
GWI	GWI
GWI	GWI
GWI	GWI
GWI	GWI
GWI	GWI
GWI	GWI

GWI	GWI
GWI	GWI
GWI	GWI
GWI	GWI
GWI	GWI
GWI	GWI
GWI	GWI
GWI	GWI
GWI	GWI
GWI	GWI
GWI	GWI
GWI	GWI
GWI	GWI
GWI	GWI
GWI	GWI
GWI	GWI
GWI	GWI
GWI	GWI
GWI	GWI
GWI	
GWI	
GWI	
GWI	
GWI	
GWI	
GWI	
GWI	
GWI	
GWI	
GWI	
SC	CFSLWIFS
SC	CFSLWIFS
SC	CIF (SC)
SC	CIF (SC)
SC	CIF (SC)
SC	CIF (SC)
SC	CIF (SC)
SC	CIF (SC)
SC	CIF (SC)
SC	SC
SC	SC
SC	SC
SC	SC
SC	SC

SC	SC
SC	SC
SC	SC
SC	SC
SC	SC
SC	SC
SC	SC
SC	SC
SC	SC
SC	SC
SC	SC
SC	SC
SC	SC
SC	SC
SC	SC
SC	SC
SC	SC
SC	SC
SC	SC
SC	SC
SC	SC
SC	SC
SC	SC
SC	SC
SC	SC
SC	SC
SC	SC
SC	SC
SC	SC
SC	SC
SC	SC
SC	SC
SC	SC
SC	SC
SC	SC
SC	SC
SC	SC
SC	SC
SC	SC
SC	SC
SC	SC
SC	SC
SC	SC
SC	

SC
SC
SC
SC
SC
